# Synthesis of Derivatives of the Antibiotic Albicidin: The N‐Terminal Fragment as Key to Control Potency and Resistance Mediated by the Binding Protein AlbA

**DOI:** 10.1002/chem.202500162

**Published:** 2025-03-11

**Authors:** Marcel Kulike‐Koczula, Kay Hommernick, Leela B. Ghimire, Simone Kosol, Lieby Zborovsky, Maria Seidel, Nicole Sattler, Andi Mainz, John B. Weston, Dmitry Ghilarov, Roderich D. Süssmuth

**Affiliations:** ^1^ Institut für Organische Chemie Technische Universität Berlin Straße des 17. Juni 124 10623 Berlin Germany; ^2^ Department of Molecular Microbiology John Innes Centre Norwich Research Park NR4 7UH Norwich UK

**Keywords:** Antibiotics, Biological activity, Drug discovery, Antimicrobial resistance, Structure-activity relationships

## Abstract

The peptide albicidin represents a highly promising lead structure which is a first‐in‐class antibiotic with remarkable potency against gram‐negative bacteria. Past efforts in the synthesis of albicidin analogs focused on increasing hydrophilicity, broadening of the antibacterial profile and overcoming resistance. Herein, we present synthetic albicidin derivatives with variations in the N‐terminal building block and characterize their antibacterial activity and DNA gyrase inhibition. Furthermore, we show that the N‐terminus of albicidin greatly affects binding to the resistance factor AlbAL. This transcription regulator senses albicidin and triggers the biosynthesis of the binding protein AlbAS, thus reducing the free concentration of the antibiotic. Here we demonstrate uncoupling of the binding event from transcription activation for some derivatives, and even a few derivatives seemed insensitive to sequestration by AlbA. This approach could be a strategy to develop albicidin analogs escaping AlbA resistance.

## Graphical abstract

This manuscript gives a deep inside in the structure‐activity relationship of the N‐terminal fragment of the antibiotic albicidin. This study uncovered a new structure scaffold comprising bicyclic aromatics as replacement for albicidin's methyl coumaric acid. Such variations enable the control of the resistance factor AlbA while maintaining excellent antibacterial activity.

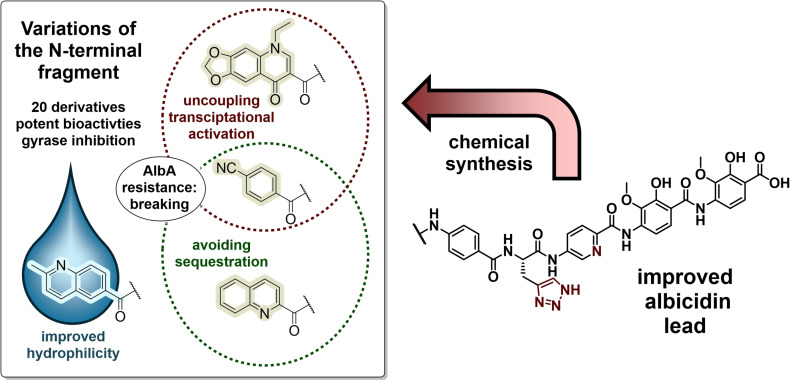

## Introduction

In 2016 the World Health Organization (WHO) declared antimicrobial resistance (AMR) as a major global public health threat facing humanity.^[1]^ The emergence of multidrug‐resistance (MDR) and the simultaneous lack of novel antibiotics prompted the Infectious Diseases Society of America (IDSA) to categorize the most concerning pathogens that are described by the acronym ESKAPE: ***E**nterococcus faecium*, ***S**taphylococcus aureus*, ***K**lebsiella pneumoniae*, ***A**cinetobacter baumannii, **P**seudomonas aeruginosa* and ***E**nterobacter* species.[^2^, ^3^] Without the development of effective counter‐measures against MDR pathogens from the ESKAPE panel, there are estimates of a dramatic increase of annual deaths caused by AMR from 1.27 million in 2019 to 10 million deaths attributable or associated to AMR in 2050.^[4]^

A candidate with potential as a future antibacterial drug is the non‐ribosomal acylpeptide (NRP) albicidin **1** produced by the plant pathogen *Xanthomonas albilineans*.^[5]^ This linear oligoarylamide natural product exhibits activity against gram–positive and particularly against gram–negative bacteria, including several ESKAPE organisms. Albicidin is active at nanomolar concentrations and represents a promising lead structure towards the development of a novel antibiotic.[^5^, ^6^, ^7^] Its mechanism of action involves the inhibition of bacterial DNA gyrase (a sub class of topoisomerase II)^[8]^ with a half maximum inhibitory concentration (IC_50_) of 40 nm which is in the same range as established gyrase inhibitors such as quinolones or coumarins.^[9]^ Albicidin occupies a site that is adjacent, but distinct from the quinolone binding site thus preventing cross resistance of fluoroquinolone‐resistant (FQR) isolates.^[8]^

**Figure 1:**
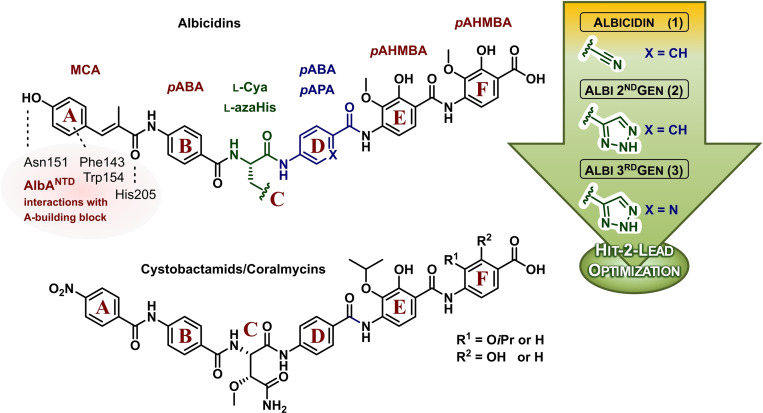
Chemical structures of albicidin **1**, 2^nd^ Gen albicidin **2**, 3^rd^ Gen albicidin **3** and structurally related cystobactamids/coralmycins. The letters A–F are assigned to the individual monomer building blocks. Interactions of the binding protein AlbA to the building block A of albicidin are indicated.

Albicidin's **1** chemical structure (Figure 1) can be divided into six building blocks assigned to the monomer building blocks A–F.^[5]^ The N‐terminal fragment AB consists of a methyl coumaric acid (MCA, building block A) acylated to a *para*‐aminobenzoic acid (*p*ABA, building block B).^[5]^ The center of the molecule is derived from the unusual amino acid β‐cyano‐l‐alanine (l‐Cya, building block C), which links the DNA binding N‐terminal fragment to the protein binding C‐terminal fragment. The tripeptidic fragment DEF is constituted by *p*ABA unit (building block D) and two *para*‐amino‐2‐hydroxy‐3‐methoxybenzoic acids (*p*AHMBA, building blocks E‐ and F)^[5]^ and wedges between two opposing α‐helices at the dimer interface of DNA gyrase.^[8]^ The structurally related cystobactamids and coralmycins are derived from benzoic acids instead of MCA in the building block A, 3‐methoxy‐l‐asparagine instead of Cya in the building block C and *iso‐*propoxy groups at the building block E‐ and/or F instead of methoxy groups (Figure 1).^[10]^ Generally, the highly aromatic character of this class of antibiotics causes them to be lipophilic and sparingly soluble in water, which makes reaching adequate plasma concentrations during treatment problematic.

The total synthesis^[11]^ of albicidin **1** was the path forward to conduct structure‐activity‐relationship (SAR) studies on oligoarylamide antibiotics, which led to new generations of albicidins with improved antibacterial activity and aqueous solubility. In brief, replacement of the central l‐Cya with l‐aza‐histidine (l‐azaHis) resulted in increased activity, increased plasma stability, increased aqueous solubility, and reduced plasma protein binding of 2^nd^ Gen albicidin **2**.[^6^, ^7^] Additional replacement of *p*ABA with *para*‐aminopicolinic acid (*p*APA) in the building block D increased the activity particularly against gram‐positive bacteria and enhanced the *in vitro* inhibition of DNA gyrase of *E. coli* yielding 3^rd^ Gen albicidin **3**.^[7]^ Additional SAR studies focused on variations of the building blocks A,[^12^, ^13^] C,^[14]^ EF^[6]^ and on amide bond isosteres countering enzymatic cleavage.[^6^, ^15^]

Mechanisms of resistance counteracting the antibacterial effects of albicidin include mutations in the *tsx* gene encoding for a gram‐negative outer membrane nucleoside transporter that facilitates cellular entry of albicidin,[^16^, ^17^] sequestration of albicidin by the binding proteins AlbA^[18]^ and STM3175^[19]^ as well as cleavage of the amide bond between the building blocks D and E by the peptidase AlbD.^[20]^ AlbA from *Klebsiella oxytoca* is of particular interest as homologues of this protein are also present in clinically relevant strains of *Klebsiella pneumoniae*.^[21]^ This protein is a MerR‐like transcriptional regulator in response to the intracellular concentration of albicidin to mitigate the effect of the gyrase poison.^[22]^ The full‐length protein (AlbAL) is a homodimer, which consists of a ligand binding domain (LBD), a coiled‐coil domain (dimerization interface) and a DNA binding domain (DBD). The crystal structure of the LBD revealed a repeat topology with an N‐terminal domain (NTD) and C‐terminal domain (CTD) which both form a hydrophobic tunnel to trap albicidin.[^18^, ^21^] Via the DBD AlbAL binds the promoter region (*pAlbA*) with micromolar affinity (*K*_d_=7.8 μM)^[22]^ repressing the promoter in absence of an inducer. Binding of albicidin to the LBD is hypothesized to allosterically mediate DNA bending via the pair of DBDs as described for other MerR‐like proteins.^[23]^ This would activate transcription of the downstream *albA* gene resulting in an upregulation of AlbAL and the truncated translational product AlbAS comprising only the LBD.^[22]^ The increased abundance of these gene products leads to protection of the bacterial strain.^[22]^

Recently, different albicidin derivatives were evaluated in terms of AlbA‐regulated transcription activation, whereby derivatives with a shortened or deleted building block A activated the promoter *pAlbA* considerably less. Since the NTD of AlbAS accommodates the N‐terminal arm of albicidin, this domain might be critical for an allosteric coupling of ligand binding and transcriptional response. This was supported by mutational studies showing that the hinge region around P209, which is in proximity to the bound building block A, is critical for signal transduction from the LBD to the DBD.^[22]^

**Figure 2:**
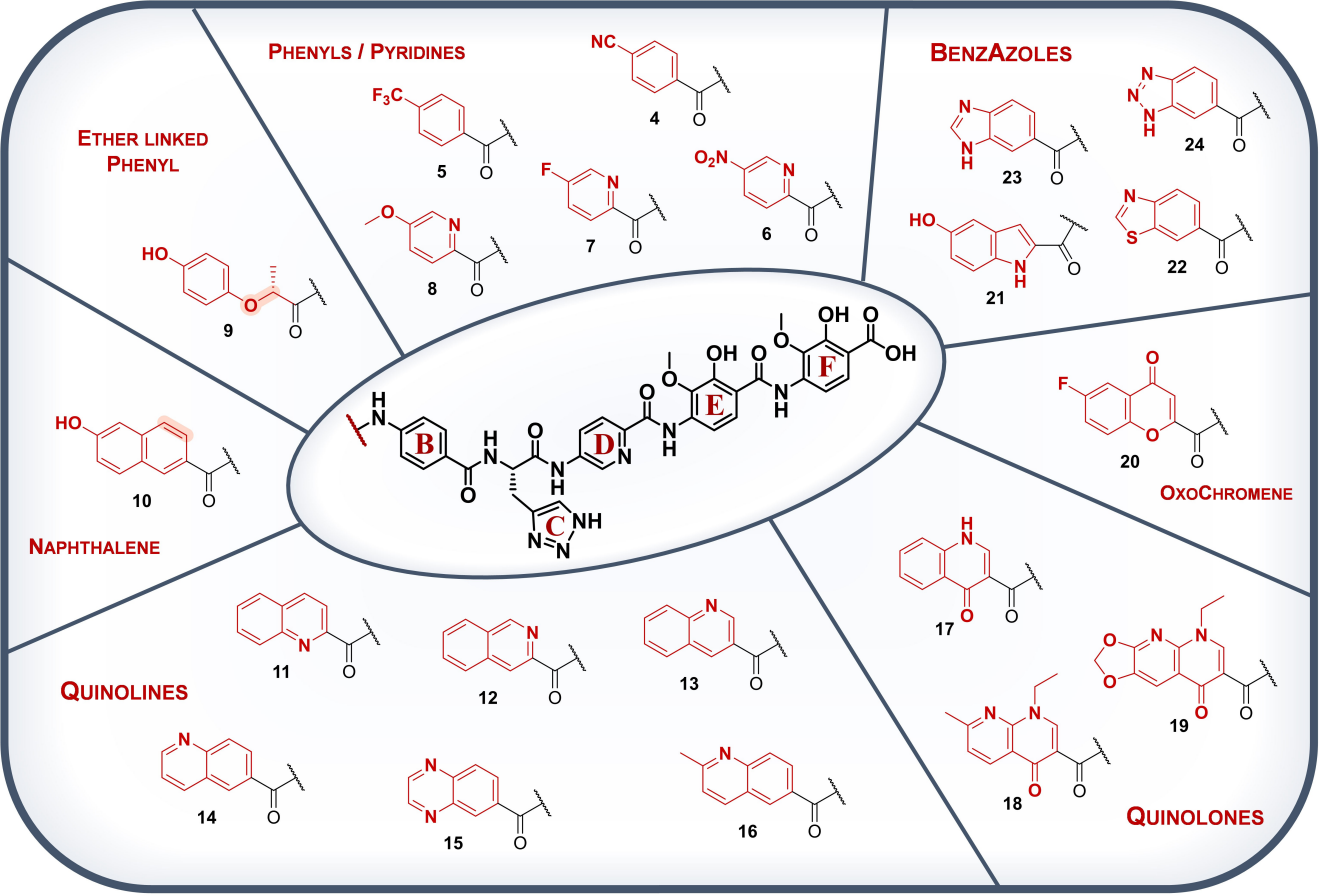
Chemical structures of A‐variations **4**–**24**. All derivatives feature the same BCDEF fragment as shown in the centre. The structures are grouped according to similarities of their scaffold and the structural changes are indicated in red. The shaded bonds in the isosteric ether **9** and naphthalene **10** highlight the altered atoms in comparison to methyl‐coumaric acid present in albicidins building block A.

The recently elucidated ternary structure of the *E. coli* gyrase:DNA:albicidin complex revealed the N‐terminal arm of albicidin as DNA intercalating residue.^[8]^ The putative promiscuity of DNA binding may in principle allow a broad scope of N‐terminally modified derivatives retaining their antibacterial activity. This would permit the fine‐tuning of physicochemical properties of derivatives and mitigating the neutralizing effect of resistance factors like AlbA. In this work, we report the synthesis of 20 derivatives with different hetero‐ or carbocyclic acyl groups replacing the N‐terminal building block A (Figure 2). Their antibacterial activity was characterized by determination of minimal inhibitory concentration (MIC) against a panel of gram‐positive and gram‐negative bacteria, as well as the inhibition of *E. coli* DNA gyrase. Additionally, the ability to escape sequestration and/or transcription regulation by resistance factor AlbA was assessed using a growth inhibition assay and a transcription activation assay.^[22]^

## Results and Discussion

The selected structural variations in the building block A were grouped into seven categories depending on their molecular scaffolds (Figure 2). The smallest scaffolds were *para*‐substituted benzoic acids and picolinic acids. Previous studies implied that electron‐accepting substituents in *para*–position of the phenyl‐ring of the building block A were beneficial for antibacterial activity.[^12^, ^13^] Thus, *para*‐cyano‐ and *para*‐trifluoromethyl benzoic acid as well as *para*‐nitro‐, *para*–fluoro‐ and *para*‐methoxy picolinic acid were chosen (**4**–**8**, Figure 2). The vinyl group of the photo‐labile coumaroyl residue was replaced with a methylene‐ether link in isosteric derivative **9**. Due to the newly introduced sp^3^‐center, this building block is more flexible and reports on the importance of the planarity of the linker between aromatic ring and amide bond. At the same time, the presence of an additional hydrogen‐bond (H‐bond) acceptor increases the polarity of derivative **9**. Another isosteric replacement in terms of length of the molecule is the substitution of the methyl coumaric acid with hydroxy‐naphthoic acid **10**, which keeps the planarity of the building block A and extends the size of the aromatic system. Moreover, various (iso)quinoline‐ and quinoxaline‐carboxylic acids **11**–**16** (Figure 2) were synthesized to assess the impact of 6,6‐fused‐heterocyclic aromatics featuring additional H‐bond acceptor(s) in different positions. Similar to pyridines, quinolines are electron‐deficient heterocycles containing a nitrogen atom in one of their rings. Thus, the ring system is electronically more polarized in comparison to isosteric naphthalenes. As a result, quinolines feature an increased basicity and can be ionized by protonation which leads to reduced lipophilicity and increased aqueous solubility.^[24]^ In addition, the ring nitrogen can form intermolecular and, depending on its position, intramolecular H‐bonds. The basicity and associated aqueous solubility of derivatives containing quinolines can further be increased by adding electron‐donating groups like the methyl group to the aromatic scaffold.^[25]^ Thereby, the electron density of an adjacent ring nitrogen atom is further increased which stabilizes the ionized protonated corresponding base.^[25]^ This reasoning led to the design of methylquinoline containing derivative **16** (Figure 2), which was reported alongside the cryoEM structure of the ternary gyrase:DNA:albicidin complex.^[8]^ The structurally related quinolones are well‐known intercalators of gyrase‐bound DNA represented by the class of quinolone antibiotics.^[26]^ Due to their bioisosterism to the coumaroyl residue of building block A, we were eager to test the impact of quinolone variants on the bioactivity of albicidin. Depending on their substitution pattern, quinolones exhibit two H‐bond acceptors and an additional H‐bond donor as embodied by derivative **17** (Figure 2), promising an increased hydrophilicity and water solubility. We also tested the potential of incorporation of the quinolone rings from the 1^st^ Gen quinolones^[27]^ nalidixic acid and oxolinic acid in albicidin derivatives **18** and **19**. We incorporated oxochromene derivative **20**, which could be useful as fluorescence label in future studies. Finally, we designed different albicidin derivatives containing 5,6‐heterocycles to assess the influence of an alternative ring size in the building block A. In contrast to quinolines, the indole scaffold of derivative **21** has an H‐bond donor in the heterocycle and features a hydroxy substituent which is also present in the coumaroyl residue of 1^st^ Gen albicidin **1**. Indoles are electron‐rich aromatic compounds with lower basicity compared to pyridines or quinolines due to the delocalization of the Lewis basic lone pair of the nitrogen atom within the aromatic ring.^[28]^ Protonation of indoles is unfavored because of the simultaneous dearomatization of the corresponding indolinium ion. In contrast to hydroxy‐indole **21**, the 5,6‐heterocyclic benzothiazole **22**, benzimidazole **23** and benzotriazole **24** are connected to the carboxy carbon of the amide bond via their 6‐membered phenyl ring (Figure 2). Due to the extended atom radius of the sulfur atom, benzothiazoles resemble from a steric perspective rather 6,6‐fused cycles such as naphthalenes or quinolines.^[29]^ The basicity of benzothiazoles is substantially lower than the basicity of benzimidazoles.^[30]^ The latter is expected to show significantly increased hydrophilicity (as in derivative **23**).

### Synthesis

**Scheme 1:**
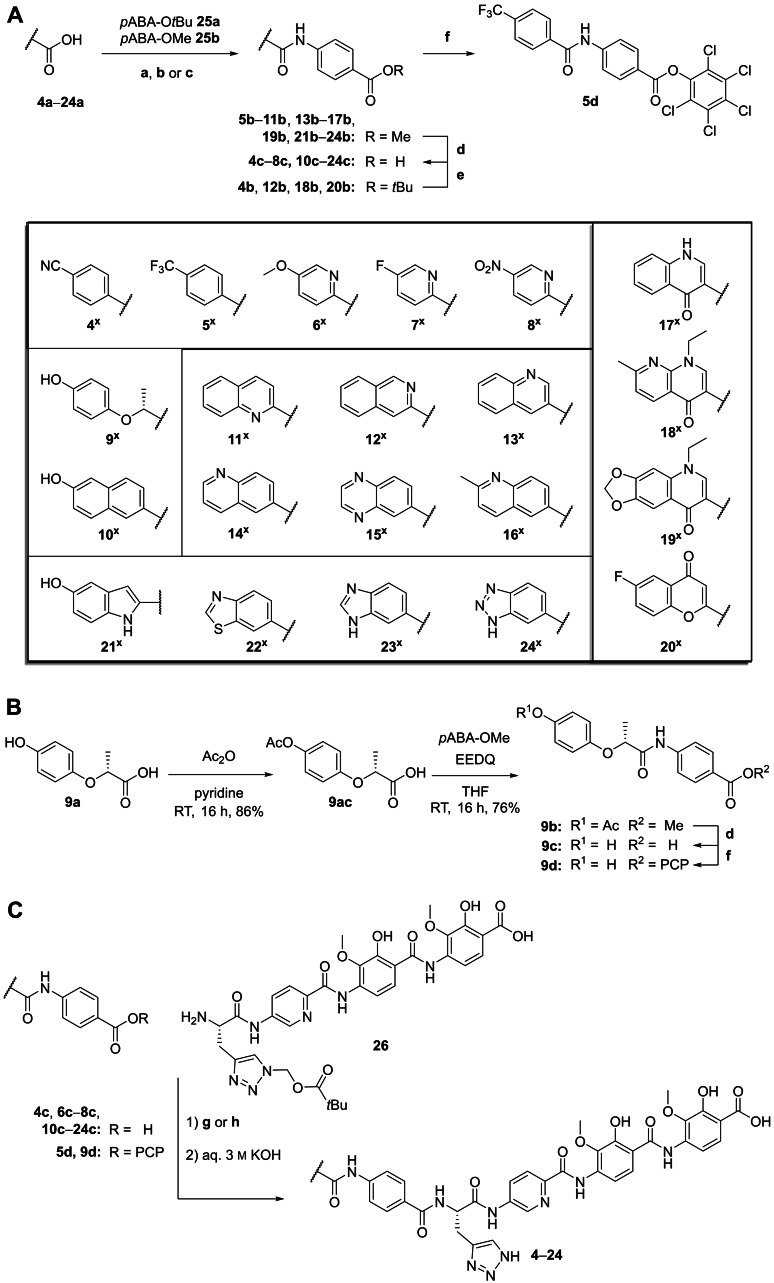
**A**) Synthesis of AB fragments **4 c**–**8 c**, **10 c**–**24 c** and PCP ester **5 d**. a) HATU, DIPEA, DMF, RT, 16 h. b) thionyl chloride, toluene, 3 h, 80 °C, then TEA, *p*ABA‐OMe, THF, RT, 16 h. c) oxalyl chloride, THF, then *p*ABA‐OMe, RT, 16 h. d) 5 m aq. KOH, MeOH/THF (1 : 1), RT, 4 h, quant. e) TFA/DCM or 4 m HCl in dioxane. f) pentachloro‐phenol, EDC, DIPEA, DMF, RT, 17 h, yield. **B**) Synthesis of PCP ester **9 d**. **C**) Final coupling of derivatives **4**–**24**. g) for compounds **4 c**, **6 c**–**8 c**, **10 c**–**24 c**: HATU, DIPEA, DMF, RT, 16 h. h) for compounds **5 d** and **9 d** DIPEA, DMF, RT, 16 h.

The synthesis of derivatives **4**–**24** makes use the pivaloyl‐oxymethyl (POM) protected CDEF‐tetrapeptide building block **26** (Scheme 1). Its assembly was previously published by Zborovsky *et al*.^[7]^ and used in this work without modification. The synthesis of AB‐fragments of the derivatives **4**–**8** and **10**–**24** was straightforward by coupling of the respective carboxylic acids **4 a**–**8 a** and **10 a**–**24 a** to *tert*‐butyl 4‐aminobenzoate (**25 a**, *p*ABA‐O*t*Bu) or methyl 4‐aminobenzoate (**25 b**, *p*ABA‐OMe) using either HATU/DIPEA, thionyl chloride/TEA or oxalyl chloride/DIPEA (Scheme 1, A). The hydroxy group of carboxylic acid **9 a** required protection by acylation with acetic anhydride in the presence of pyridine. The protected intermediate **9 ac** was subsequently coupled to *p*ABA‐OMe **25 b** using EEDQ (Scheme 1, B). The methyl esters **5 b**–**11 b**, **13 b**–**17 b**, **19 b**, **21 b**–**24 b** were saponified with aqueous KOH solution giving the corresponding carboxylic acids. The *tert*‐butyl esters **4 b**, **12 b**, **18 b**, **20 b** were deprotected in presence of TFA/DCM or HCl in dioxane. The carboxylic acids **4 c**–**24 c** were coupled to POM‐protected CDEF‐tetrapeptide **26** either by using HATU/DIPEA or by preactivation via the corresponding pentachlorophenol (PCP) esters **5 d** and **9 d** (Scheme 1, C). After deprotection of the POM‐protected triazole using an aqueous KOH solution, the final derivatives **4**–**24** were obtained after HPLC purification (for synthesis protocols and analytical data see Supporting Information).

As mentioned before, albicidin **1** is a rather hydrophobic compound and improving its aqueous solubility is desirable. The lipophilicity of drug molecules is commonly characterized by the octanol/water partition coefficient (log*P*). An alternative approach relies on the correlation between lipophilicity and the retention time (Rt) on a reverse‐phase HPLC column.^[31]^ This approach works well if structurally related compounds are compared as is the case for this SAR study. Rt was thus used to estimate the hydrophilicity of albicidin **1** and its derivatives **2**–**24** (Table 1).^[32]^

**Table 1:**
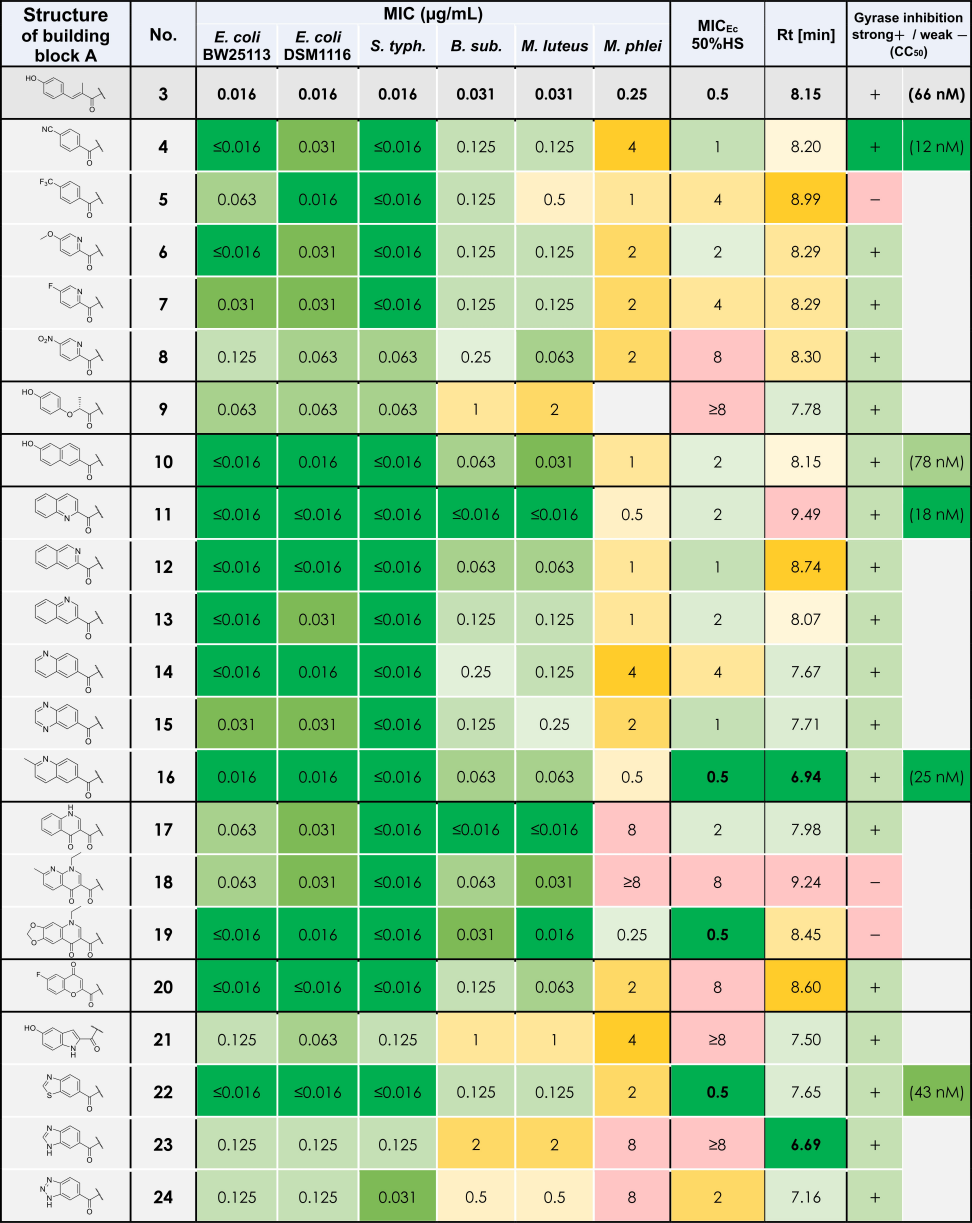
Minimal inhibitory concentration (MIC) values of derivatives **3**–**24** given in μg/mL. *E. coli*=*Escherichia coli*, *S. typh*.=*Salmonella typhimurium*, *B. sub*.=*Bacillus subtilis*, *M. luteus*=*Micrococcus luteus*, *M. phlei*=*Mycobacterium phlei*. Rt=retention time. Gyrase inhibition: qualitative DNA cleavage assay at constant concentrations (1 μM and 10 μM) of compounds **5**–**9**, **12**–**15**, **17**–**21**, **23** and **24**; symbols: + strong inhibition, − weak inhibition in comparison to compound **16**, for details see SI. Figures 1–3; CC_50_ values of compounds **3**, **4**, **10**, **11**, **16** and **22**, for details see SI. Figures 4–8. MIC and CC_50_ of compound **16** were already published elsewhere.^[8]^

### Antibacterial Activity of Synthetic Albicidin Variants

After completing the syntheses, the antibacterial activity of A‐variations **4**–**24** was assessed by determination of MIC values against a basic set of gram‐negative and gram‐positive bacteria (Table 1). The monocyclic derivatives **4**–**8** featuring a cyano‐, trifluoromethyl‐, methoxy‐ and fluoro‐ substituent in *para*‐position were very active against the gram–negative *E. coli* and *S. typhimurium* strains with MIC values in the same range as 3^rd^ Gen albicidin **3** or ciprofloxacin which is the gold standard for gyrase inhibitors. The nitro analog **8** exhibited somewhat lower activity against gram‐negatives with up to eight‐fold higher MIC values compared to lead structure **3**. These findings are in agreement with previous studies showing good activities for cyano, trifluoromethyl, methoxy and fluoro substituents in the *para*‐position of different aromatic rings in the building block A.[^12^, ^13^] The activity of the monocyclic derivatives **4**–**8** against the gram‐positive strains *B. subtilis* and *M. luteus* were similar but slightly less pronounced compared to the lead structure **3**. The retention time (Rt) as an indicator of the lipophilicity^[32]^ of compounds **4**–**8** was slightly increased which suggests a decreased water solubility, especially in the case of trifluoro‐methyl derivative **5**. The isosteric ether linked compound **9** had slightly decreased activities against gram‐negative bacteria suggesting a relatively low impact of the flexible linker unit on cell entry and target binding; however, the activity against gram‐positive bacteria is about at least one order of magnitude lower than that for the lead structure **3**. This compound also suffers from high plasma protein binding, as indicated by the poor MIC value against *E. coli* in presence of 50 % human serum (MIC_Ec_ 50 %HS), despite exhibiting a shorter Rt.

The activity of compound **10** with the naphthalene scaffold seems to match the bioactivity of the lead structure, proving bicyclic 6‐membered rings as a suitable isosteric replacement for the photolabile coumaroyl residue although the MIC_Ec_ 50 %HS is increased four‐fold. The quinoline series of compounds **11**–**16** further emphasized the potential of bicyclic building blocks with high activities against gram‐negative bacteria (MIC: 0.016–0.031 μg/mL). Derivatives **12**–**15** also showed good activities against the gram‐positive bacteria *B. subtilis* and *M. luteus* (MIC: 0.063–0.25 μg/mL), whereas quinoline **11** had outstanding activity with a twofold increase in activity compared to lead compound **3**. Despite being isomers, the Rt of quinolines **11**–**14** differed dramatically depending on the placement of the ring nitrogen and its basicity.^[24]^ The strongest increase of the Rt was observed for quinolines **11** and **12** which is likely due to the electron withdrawing effect of the adjacent carbonyl group destabilizing the positive charge of the corresponding quinolinium ion.^[33]^ Additionally, these nitrogen atoms are in close proximity to the adjacent proton of the A–B amide bond and will very likely form an intramolecular H‐bond (IMHB) which impairs protonation or H‐bonding of the quinoline nitrogen with hydrogen atoms of protic solvents like water. A similar formation of IMHB is described for the pyridine moiety of the building block D of 3^rd^ Gen albicidin **3**, where this hypothesis was supported by DFT calculations.^[7]^ In the latter case, increased antibacterial activity was believed to be caused by the planarization of the DEF fragment indicated by a decreased dihedral angle between the pyridine ring and the C‐terminal amide bond. Compared to 2^nd^ Gen albicidin **2**, which does not feature a pyridine ring as the D fragment, 3^rd^ Gen albicidin **3** and other pyridine containing derivatives forming an IMHD also show increased retention times.^[7]^ Analogously, a similar planarization effect is predicted for quinolines **11** and **12**, which might explain the increased bioactivity. This effect can be particularly observed by comparing quinoline **13**, which cannot form an IMHB with the adjacent amide proton, to quinolines **11** and **12**. The activity against gram‐positive strains of these derivatives is increasing in the same way as the retention times (Rt [min]: 8.07 (**13**), 8.74 (**12**), 9.49 (**11**); MIC [μg/mL] vs *B. subtilis* and *M. luteus*: 0.125 (**13**), 0.063 (**12**), ≤0.016 (**11**)). The reason for that could be more efficient permeation of gram‐positive bacterial membranes which in contrast to the outer membrane of gram‐negative bacteria are readily penetrated by lipophilic molecules via passive diffusion.^[34]^ Alternatively, the planarization of the AB‐fragment could also result in a more efficient intercalation into gyrase‐bound DNA resulting in a higher target affinity.

Compared to quinoline **13**, quinoline **14** and quinoxaline **15** are slightly less active against gram‐positive bacteria with up to four–fold higher MIC values. The heterocycles **14** and **15** are more hydrophilic because the ring nitrogens are located in the terminal 6‐membered ring of the bicycle. The basicity of the nitrogen atoms is less affected by the more distant carbonyl group of the amide bond in comparison to quinoline **13**, in which the base is located in the ring adjacent to the amide. A compound of particular interest is methylquinoline **16**. This compound is more basic compared to the desmethyl variant **14**, due to the electron donating effect of the methyl group adjacent to the ring nitrogen similar to methylated pyridine bases.^[25]^ Compound **16** is not only by far the most hydrophilic quinoline derivative (Rt: 6.94 min), but the incorporation of the methyl group led also to an up to four‐fold increase in activity against gram‐positive bacteria compared to the desmethyl variant **14**. Besides, among all tested derivatives, methylquinoline **16** performed the best in the presence of human serum and thus, may have the lowest plasma protein binding (MIC_Ec_ 50 %HS: 0.5 μg/mL).

The quinolones **17**–**19** had a broad spectrum of activity with MIC values between 0.016–0.063 μg/mL against all strains except *M. phlei* which was insensitive to quinolones **17**–**18**. Oxolinic acid derivative **19** had overall the best activity among the quinolones, outperforming the lead compound **3** with regard to antibacterial activity. In contrast to unsubstituted quinolone **17** and oxolinic acid **19**, nalidixic acid derivative **18** had poor MIC_EC_ 50 %HS indicating strong plasma protein binding likely due to its comparably high lipophilicity (Rt: 9.24 min).

The fluorescent fluoro‐oxochromene derivative **20** was also a successful modification of albicidin's building block A exhibiting comparable activities against gram‐negative bacteria as the lead compound **3**. This compound had slightly decreased activities against gram‐positive bacteria and suffered from poor MIC_Ec_ 50 %HS which indicates high plasma protein binding, probably due to its higher lipophilicity (Rt: 8.60 min).

Hydroxy‐indole containing derivative **21**, benzotriazole **24** and especially benzimidazole **23** showed short retention times and thus are rather more polar (Rt [min]: 7.50 (**21**), 6.69 (**23**), 7.16 (**24**)) but unfortunately had slightly decreased antibacterial activities against gram‐negative and gram‐positive bacteria. Intriguingly, benzothiazole **22** had similar activities to the lead structure **3** with increased hydrophilicity (Rt: 7.65 min) and comparably low plasma protein binding (MIC_Ec_ 50 %HS: 0.5 μg/mL). Since sulfur has a wider atom radius than carbon and nitrogen, thiazoles are isosteric to 6‐membered rings such as phenyl and pyridine, respectively.^[29]^ This might indicate that the optimal scaffold for albicidin's building block A is a bicyclic 6,6‐carbo‐ or 6,6‐heterocycle with the terminal cycle connecting to the *meta*‐ and *para*‐position. Other bicyclic ring configurations led to molecules with lowered activities.^[35]^

### Gyrase Activity of Synthetic Derivatives

**Figure 3:**
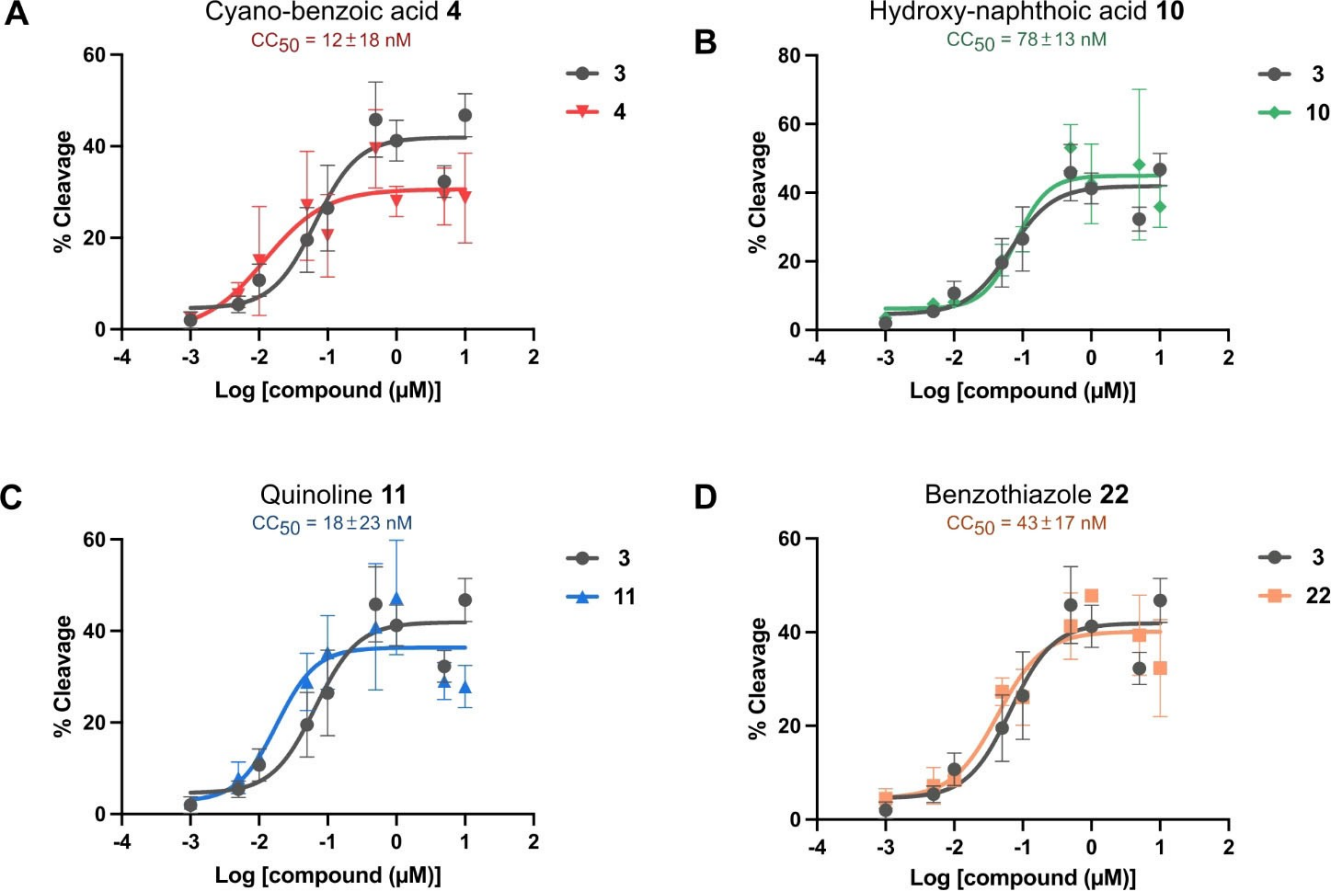
Susceptibility of *E. coli* gyrase to different albicidin derivatives in comparison to 3^rd^ Gen albicidin **3**. CC_50_ values were determined for A) cyano‐benzoic acid **4**, B) hydroxy‐naphthoic acid **10**, C) quinoline **11** and D) benzothiazole **22**. Error bars represent SD of triplicates.

The target activity of the synthesized derivatives was investigated using a DNA cleavage assay with purified and enzymatically active *E. coli* DNA gyrase^[8]^ (see SI). In this assay, albicidin‐stabilized complexes of gyrase and plasmid DNA are trapped, and subsequently denatured and deproteinated to reveal DNA linearized by gyrase. Initial qualitative screening experiments (see SI Figure 1–3) revealed that at a concentration of 10 μM all derivatives except for trifluoro‐benzoic acid derivative **5** and quinolone‐related derivatives **18** and **19** caused accumulation of cleaved DNA (Table 1). This is a surprising result as **5**, **18** and **19** had a pronounced antibacterial activity, which hints to the presence of a different target, probably *E. coli* topoisomerase IV which was previously identified as a second target of albicidin analogs.^[8]^ For the best performing compounds, which were able to fully stabilize the cleavage complex at a concentration of 1 μM, the concentrations at half‐maximum cleavage (CC_50_) were determined (Figure 3). Several A‐variations yielded lower CC_50_ values than the reference compound **3** (66 nM) indicating an improved target activity. For instance, benzothiazole **22** with a CC_50_ value of 43 nM was slightly more active on gyrase than the lead structure **3**. In particular cyano–benzoic acid **4** as well as quinoline **11** and methylquinoline **16** showed a significant improvement of cleavage‐complex stabilization with CC_50_ values of 12 nM, 18 nM and 25 nM respectively. Naphthoic acid derivative **10** was slightly less active on gyrase (CC_50_=78 nM) in comparison to the lead structure **3**.

Interestingly, target activity and hydrophobicity estimate (Rt) correlated with the MIC_Ec_ 50 %HS. As mentioned earlier, albicidins and cystobactamids suffer from plasma protein binding with up to 99 % binding.^[36]^ In general, less hydrophobic molecules tend to bind less to plasma proteins, which leads to a higher concentration of free drug molecules that can enter a bacterial cell.^[37]^ On the other hand, albicidins with higher target activity require less molecules for intracellular accumulation from the extracellular compartment to inhibit bacterial growth. A balanced combination of both properties embodied in compounds **16** and **22** translated well into promising MIC_Ec_ 50 %HS (Table 2). At the same time, compounds **4**, **10** and **11** with either severely increased Rt/hydrophobicity or reduced target activity had lower activity in presence of serum.

**Table 2:**
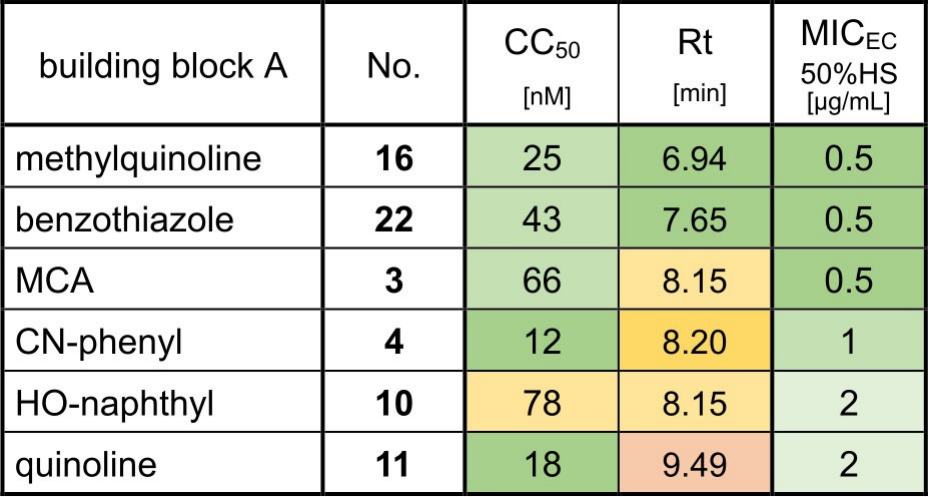
Synopsis of effects of target activity and hydrophobicity on antibacterial potency of albicidin derivatives. *E. coli* DNA gyrase activity (CC_50_) and HPLC‐retention times (Rt) as estimates of hydrophobicity are compiled with anti‐*E. coli* activity in presence of 50 % human serum (MIC_Ec_ 50 %HS) for selected albicidin analogs. Favorable, moderate or poor values are highlighted in green, yellow or red, respectively.

### Synthetic Albicidin Derivatives Evading AlbA Resistance

**Figure 4:**
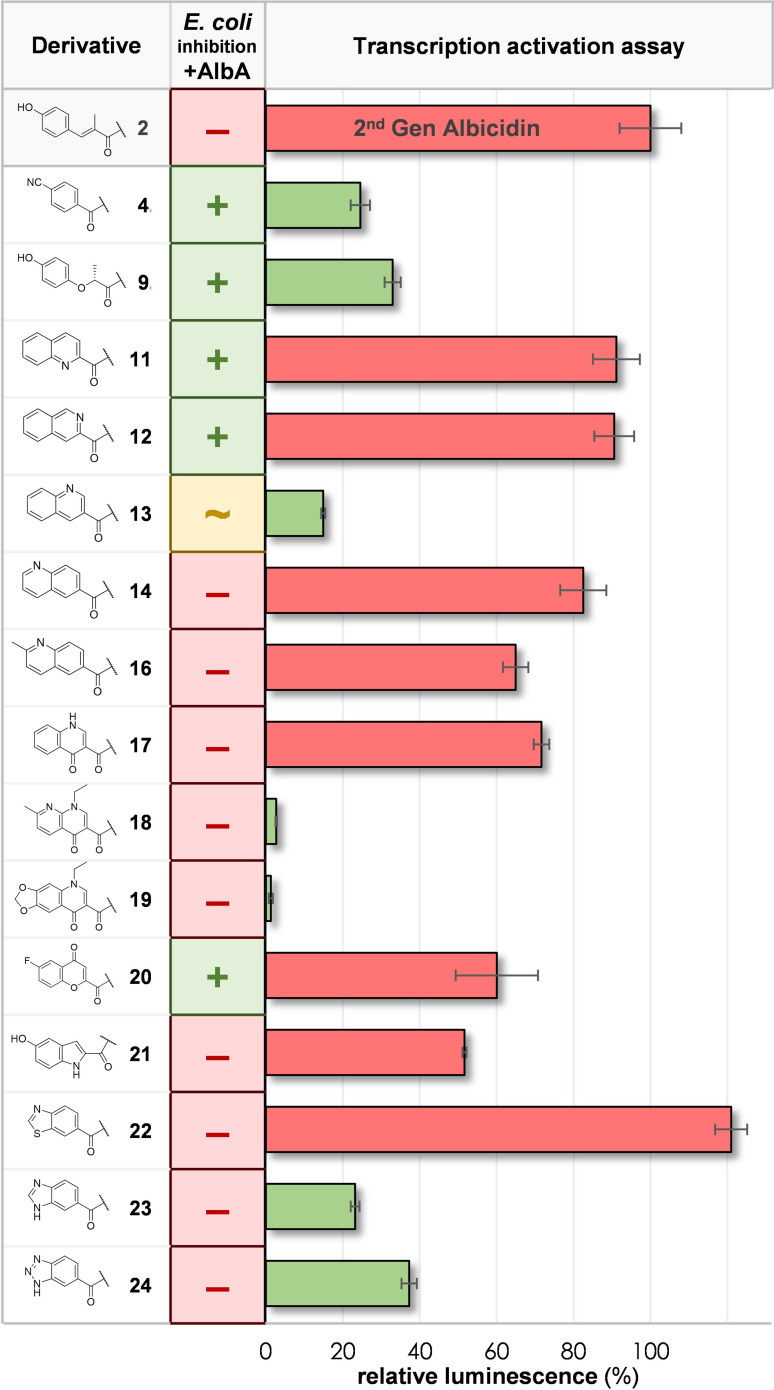
Characterization of AlbA resistance against derivatives **2**, **4**–**24**. GIA: Qualitative growth inhibition assay against *E. coli* DSM 1116. (−): Derivative unable to inhibit the growth of *E. coli* in presence of AlbAS. (+): Derivative inhibits growth of *E. coli* in presence of AlbAS. (~): Derivative partially inhibits growth of *E. coli* in presence of AlbAS. For details see SI Figure 9. Transcription activation (TA) assay: luminescence elicited by 2^nd^ Gen albicidin **2** from the TA assay was normalized to 100 % and the luminescence signal of tested derivatives provided: relative luminescence [%]=luminescence [derivative]**/**luminescence [**2**]×100 %. Green bars indicate low relative luminescence (<40 %) and red bars indicate high relative luminescence (>50 %). Error bars show the standard deviations of triplicate measurements.

Finally, we investigated if synthetic variations of building block A are able to evade the effects of the resistance protein AlbA. Therefore, we carried out a qualitative growth inhibition assay with derivatives **4**–**24** in presence of an equimolar amount of purified AlbAS. After incubation of the cultures overnight, the growth of the bacteria was evaluated. The ability of AlbAS to sufficiently sequester an albicidin derivative from the supernatant allowed *E. coli* cells to accumulate in a visible cell pellet. Second, we performed a quantitative transcription‐activation reporter assay^[22]^ to assess transcriptional regulation by AlbAL. The promoter sequence *pAlbA* was inserted into a reporter vector that encodes the *ilux* luminescence cassette.^[22]^ The luminescence signals elicited by ligand binding of synthetic variations to AlbAL was compared and normalized to the signal induced by 2^nd^ Gen albicidin **2** and are hereby referred to as relative transcription activation (TA^%^). The combination of both assays prompted us to categorize the experimental outcomes (Figure 4) in four groups:

**Group 1** (compounds **4**, **9** and **13**) has bioactivity in presence of AlbAS and desirably low TA^%^ (<50 %). The shortened fragment A of compound **4** might evade interactions with residues in the NTD, e. g. Asn151 and Phe143 (Figure 1). Curiously, a similar TA^%^ was reported previously for a truncated derivative lacking the fragment A.^[22]^ The loss of these contacts with the NTD may prevent proper bridging of the CTD and the NTD by the ligand, which appears to be relevant for efficient allosteric signal transduction within the multi‐domain regulator.^[22]^ A similar binding mode was proposed on basis of NMR experiments with a cystobactamid related nitro‐benzoyl derivative.^[18]^ The increased flexibility or absence of the sp^2^ character of the aliphatic ether in compound **9** may destabilize the ligand in the NTD as well. How quinoline **13** inhibits signal transduction however remains elusive.

**Group 2** (compounds **11**, **12**, **20**) is highly bioactive in presence of AlbAS but has undesirable high TA^%^ (>50 %). Although a binding event with the LBD of AlbAL is evident from the reporter assay, these compounds were not fully captured by AlbAS and thus inhibited the growth of *E. coli* cells. On the other hand, the residence times of these ligands in the AlbAL:*pAlbA* complex seem sufficient to induce the required conformational change for transcriptional response. As outlined before,^[22]^ trapping of albicidin by AlbAS does not necessarily correlate with induction of transcription activation. These findings emphasize that small changes in binding kinetics may have significant consequences for the overall cellular performance of compounds. Nevertheless, possible differences of cellular uptake rates for the compounds may impact the competition between AlbAS and influx through the Tsx channel. Thus, the uptake rate affects how dramatic the consequence of toxin dissociation from AlbAS/AL is. **Group 3** (compounds **18**, **19**, **23**, **24**) has low TA^%^ but representatives are deactivated in presence of AlbAS. Especially, the *N*‐alkylated quinolones **18** and **19** induced a remarkably low transcriptional response, despite the ability of AlbAS to capture these compounds. This suggests that the ligand binding is uncoupled from the allosteric conformational change of AlbAL required to activate the promoter *pAlbA*. Alternative binding modes with AlbAS distinct from the albicidin:AlbAS complex have also been described for other gyrase poisons, although the relationship with transcription activation remains elusive.^[38]^ The steric demand of the *N*‐ethyl group at the quinolone core of **18** and **19** might interfere with the typical binding mode of albicidins. Nevertheless, these results indicates that bacterial strains harbouring the *albA* gene might not be able to upregulate the expression of the binding protein when challenged with compounds **18** and **19**. Additionally, it is unclear whether basal levels of AlbAL are sufficient to protect the cell from the action of these poisons. Studies on *E. coli* mutants showed that simple overexpression of AlbA is not sufficient to protect bacteria against the natural product albicidin **1**.^[21]^ In general, this could imply that transcription activation is critical for AlbA mediated resistance rather than solely binding affinity of AlbA. Future MIC testing against resistant strains is necessary to assess the potential of compounds from **group 3** to escape from the action of AlbA.

Compounds of **Group 4** (compounds **2**, **14**, **16**, **17**, **21**, **22**) are deactivated in presence of AlbAS and induce strong TA^%^. These features are less desired towards the development of a resistance‐breaking oligoarylamide antibiotic. Typically, these compounds are isosteric to MCA like 6,6‐fused bicycles or benzothiazole as discussed before. An exception is hydroxy‐indole **21**, which induced only an intermediate TA^%^, probably due to the kink in the structure introduced by the 5‐membered ring.

## Conclusions

In summary, we have synthesized a comprehensive set of 20 new A‐variations of albicidin shedding light on the SAR and the structure‐resistance relationship (SRR) of albicidin's DNA binding epitope. This study revealed a wide scope of accepted structural motifs, as all synthesized compounds showed excellent activities against gram‐negative *E. coli* and *Salmonella* strains. The concept of replacing the vinyl group of albicidin's photo‐labile coumaroyl residue with an aromatic cycle fused to the terminal aromatic ring was demonstrated. Among the most potent derivatives were especially 6,6‐fused heterocycles with quinoline **11** and quinolone **19**, showing the strongest broad‐spectrum activity. The most interesting derivatives are methylquinoline **16** and benzothiazole **22** which represent the optimal compromise between hydrophilicity and high activities against gram‐negative and gram‐positive bacteria. In addition to their decent hydrophilicity, these derivatives are very potent gyrase poisons. Taken together, these traits translated into good antibacterial activities of **16** and **22** in presence of human serum.

Albicidin variants tested in the transcription activation assay of the drug‐binding protein AlbAL demonstrated that there are variants able to avoid up‐regulation of *albA* expression. Despite being able to activate transcription, some derivatives even could escape sequestration by AlbAS and thus inhibit the growth of *E. coli*. Thus, two mechanisms to break AlbA‐mediated resistance are conceivable and should be pursued in the future compound design: evading sequestration by the LBD of AlbA and/or uncoupling the transcriptional response from the sensor domain. The best candidates evading the action of the resistance protein were quinolines **11** and **12**, oxochromene **20** (avoiding sequestration, **group 2**), quinolones **18** and **19** (uncoupling transcription activation, **group 3**) and cyano‐benzoyl derivative **4** (both, **group 1**). Hence, this study provides strategies and structural features to avoid the action of transcription regulators like AlbA employing highly potent gyrase poisons, which will be substantial for future albicidin drugs.

## Conflict of Interests

The authors declare no conflict of interest.

1

## Supporting information

As a service to our authors and readers, this journal provides supporting information supplied by the authors. Such materials are peer reviewed and may be re‐organized for online delivery, but are not copy‐edited or typeset. Technical support issues arising from supporting information (other than missing files) should be addressed to the authors.

Supporting Information

## Data Availability

Experimental procedures and analytical data are available in the ESI (Supporting Information_Kulike‐Koczula_25.02.2025).

## References

[1] J. O'Neill , Tackling Drug-Resistant Infections Globally: Final Report and Recommendations, Government of the United Kingdom, 2016, 84 pp. https://amr-review.org/.

[2] J. N. Pendleton , S. P. Gorman , B. F. Gilmore , Expert Rev. Anti-Infect. Ther. 2013, 11, 297–308. 23458769 10.1586/eri.13.12

[3] L. B. Rice , J. Infect. Dis. 2008, 197, 1079–1081. 18419525 10.1086/533452

[4] M. Naghavi , S. E. Vollset , K. S. Ikuta , L. R. Swetschinski , A. P. Gray , E. E. Wool , et al., The Lancet 2024, 404, 1199–1226. 10.1016/S0140-6736(24)01867-1 PMC11718157 39299261

[5] S. Cociancich , A. Pesic , D. Petras , S. Uhlmann , J. Kretz , V. Schubert , L. Vieweg , S. Duplan , M. Marguerettaz , J. Noëll , I. Pieretti , M. Hügelland , S. Kemper , A. Mainz , P. Rott , M. Royer , R. D. Süssmuth , Nat. Chem. Biol. 2015, 11, 195–197. 25599532 10.1038/nchembio.1734

[6] I. Behroz , P. Durkin , S. Grätz , M. Seidel , L. Rostock , M. Spinczyk , J. B. Weston , R. D. Süssmuth , Chem. Eur. J. 2019, 25, 16538–16543. 31642561 10.1002/chem.201904752 PMC6972991

[7] L. Zborovsky , L. Kleebauer , M. Seidel , A. Kostenko , L. von Eckardstein , F. O. Gombert , J. Weston , R. D. Süssmuth , Chem. Sci. 2021, 12, 14606–14617. 34881013 10.1039/d1sc04019g PMC8580050

[8] E. Michalczyk , K. Hommernick , I. Behroz , M. Kulike , Z. Pakosz-Stępień , L. Mazurek , M. Seidel , M. Kunert , K. Santos , H. von Moeller , B. Loll , J. B. Weston , A. Mainz , J. G. Heddle , R. D. Süssmuth , D. Ghilarov , Nat. Catal. 2023, 6, 52–67. 36741192 10.1038/s41929-022-00904-1 PMC9886550

[9] S. M. Hashimi , M. K. Wall , A. B. Smith , A. Maxwell , R. G. Birch , Antimicrob. Agents Chemother. 2007, 51, 181–187. 17074789 10.1128/AAC.00918-06 PMC1797663

[10] S. Baumann , J. Herrmann , R. Raju , H. Steinmetz , K. I. Mohr , S. Hüttel , K. Harmrolfs , M. Stadler , R. Müller , Angew. Chem. Int. Ed. 2014, 53, 14605–14609. 10.1002/anie.201409964 25510965

[11] J. Kretz , D. Kerwat , V. Schubert , S. Grätz , A. Pesic , S. Semsary , S. Cociancich , M. Royer , R. D. Süssmuth , Angew. Chem. Int. Ed. 2015, 127, 1992–1996. 10.1002/anie.201409584 25504839

[12] D. Kerwat , S. Grätz , J. Kretz , M. Seidel , M. Kunert , J. B. Weston , R. D. Süssmuth , ChemMedChem 2016, 11, 1899–1903. 27439374 10.1002/cmdc.201600231

[13] I. Behroz , L. Kleebauer , K. Hommernick , M. Seidel , S. Grätz , A. Mainz , J. B. Weston , R. D. Süssmuth , Chem. Eur. J. 2021, 27, 9077–9086. 33769627 10.1002/chem.202100523 PMC8362182

[14] S. Grätz , D. Kerwat , J. Kretz , L. von Eckardstein , S. Semsary , M. Seidel , M. Kunert , J. B. Weston , R. D. Süssmuth , ChemMedChem 2016, 11, 1499–1502. 27245621 10.1002/cmdc.201600163

[15] L. Kleebauer , L. Zborovsky , K. Hommernick , M. Seidel , J. B. Weston , R. D. Süssmuth , Org. Lett. 2021, 23, 7023–7027. 34398605 10.1021/acs.orglett.1c02312

[16] R. G. Birch , J. M. Pemberton , W. V. S. Basnayake , Microbiology 1990, 136, 51–58. 10.1099/00221287-136-1-51 2191080

[17] H. Fsihi , B. Kottwitz , E. Bremer , J. Biol. Chem. 1993, 268, 17495–17503. 8349629

[18] L. Rostock , R. Driller , S. Grätz , D. Kerwat , L. von Eckardstein , D. Petras , M. Kunert , C. Alings , F.-J. Schmitt , T. Friedrich , M. C. Wahl , B. Loll , A. Mainz , R. D. Süssmuth , Nat. Commun. 2018, 9, 3095. 30082794 10.1038/s41467-018-05551-4 PMC6078987

[19] M. Saathoff , S. Kosol , T. Semmler , K. Tedin , N. Dimos , J. Kupke , M. Seidel , F. Ghazisaeedi , S. A. Wolf , B. Kuropka , W. Czyszczoń , D. Ghilarov , S. Grätz , J. G. Heddle , B. Loll , R. D. Süssmuth , M. Fulde , PLoS Biol. 2022, 21, e3002186. 10.1371/journal.pbio.3002186 PMC10414762 37561817

[20] L. Vieweg , J. Kretz , A. Pesic , D. Kerwat , S. Grätz , M. Royer , S. Cociancich , A. Mainz , R. D. Süssmuth , J. Am. Chem. Soc. 2015, 137, 7608–7611. 26057615 10.1021/jacs.5b04099

[21] A. Sikandar , K. Cirnski , G. Testolin , C. Volz , M. Brönstrup , O. V. Kalinina , R. Müller , J. Koehnke , J. Am. Chem. Soc. 2018, 140, 16641–16649. 30422653 10.1021/jacs.8b08895

[22] S. Kosol , L. Rostock , J. Barsig , T. Tabarelli , K. Hommernick , M. Kulike , T. F. Eulberg , M. Seidel , I. Behroz , L. Kleebauer , S. Grätz , A. Mainz , R. D. Süssmuth , Chem. Sci. 2023, 14, 5069–5078. 37206387 10.1039/d3sc00955f PMC10189885

[23] C. Fang , L. Li , Y. Zhao , X. Wu , S. J. Philips , L. You , M. Zhong , X. Shi , T. V. O'Halloran , Q. Li , Y. Zhang , Nat. Commun. 2020, 11, 6284. 33293519 10.1038/s41467-020-20134-y PMC7722741

[24] R. S. Hosmane , J. F. Liebman , Struct. Chem. 2009, 20, 693–697.

[25] A. Gero , J. J. Markham , J. Org. Chem. 1951, 16, 1835–1838.

[26] T. D. M. Pham , Z. M. Ziora , M. A. T. Blaskovich , MedChemComm 2019, 10, 1719–1739. 31803393 10.1039/c9md00120d PMC6836748

[27] C. M. Oliphant , G. M. Green , Am. Fam. Physician 2002, 65, 455–465. 11858629

[28] R. L. Hinman , J. Lang , J. Am. Chem. Soc. 1964, 86, 3796–3806.

[29] A. Flood , C. Trujillo , G. Sanchez-Sanz , B. Kelly , C. Muguruza , L. F. Callado , I. Rozas , Eur. J. Med. Chem. 2017, 138, 38–50. 28644987 10.1016/j.ejmech.2017.06.008

[30] R. Notario , M. Herreros , E. Ballesteros , M. Essefar , J.-L. M. Abboud , I. D. Sadekov , V. I. Minkin , J. Elguero , J. Chem. Soc. Perkin Trans. 2 1994, 0, 2341–2344.

[31] A. Carotti , I. Varfaj , I. Pruscini , G. W. A. Abualzulof , L. Mercolini , E. Bianconi , A. Macchiarulo , E. Camaioni , R. Sardella , J. Sep. Sci. 2023, 46, 2300346. 10.1002/jssc.202300346 37438993

[32] K. Valkó , C. Bevan , D. Reynolds , Anal. Chem. 1997, 69, 2022–2029. 21639241 10.1021/ac961242d

[33] G. J. Rowlands , R. J. Severinsen , J. K. Buchanan , K. J. Shaffer , H. T. Jameson , N. Thennakoon , I. Leito , M. Lõkov , A. Kütt , R. Vianello , I. Despotović , N. Radić , P. G. Plieger , J. Org. Chem. 2020, 85, 11297–11308. 32786648 10.1021/acs.joc.0c01428

[34] L. L. Silver , Clin. Microbiol. Rev. 2011, 24, 71–109. 21233508 10.1128/CMR.00030-10 PMC3021209

[35] G. Testolin , K. Cirnski , K. Rox , H. Prochnow , V. Fetz , C. Grandclaudon , T. Mollner , A. Baiyoumy , A. Ritter , C. Leitner , J. Krull , J. van den Heuvel , A. Vassort , S. Sordello , M. M. Hamed , W. A. M. Elgaher , J. Herrmann , R. W. Hartmann , R. Müller , M. Brönstrup , Chem. Sci. 2020, 11, 1316–1334. 10.1039/c9sc04769g PMC8148378 34123255

[36] H.-Y. Choi , B.-M. Kim , Y.-R. Kim , T. Yang , S. Ahn , D. Yong , J.-H. Kwak , W.-G. Kim , Antibiotics 2022, 11, 902. 35884156 10.3390/antibiotics11070902 PMC9311539

[37] M. L. Howard , J. J. Hill , G. R. Galluppi , M. A. McLean , Comb. Chem. High Throughput Screening 2010, 13, 170–187. 10.2174/138620710790596745 20053162

[38] Y. M. Surani, M. Wand, P. Picconi, M. D. Palma, R. Z. Chiozzi, M. M. Hasan, M. Maynard-Smith, R. Steiner, K. M. Rahman, C. Hind, M. Sutton, **2024**, Research Square preprint: DOI: 10.21203/rs.3.rs-4901630/v1, https://chemistry-europe.onlinelibrary.wiley.com/hub/journal/15213765/notice-to-authors.

