# Supplementary material for: Synthesis of Derivatives of the Antibiotic Albicidin: The N‐Terminal Fragment as Key to Control Potency and Resistance Mediated by the Binding Protein AlbA
Source: Chemistry. 2025 Mar 11;31(21):e202500162. doi: 10.1002/chem.202500162 (PMC11979685; doi:10.1002/chem.202500162)
Supplement: Supplementary file 1 — Supporting Information [file CHEM-31-e202500162-s001.pdf]

Chemistry–A European Journal

Supporting Information

Synthesis of Derivatives of the Antibiotic Albicidin: The N-Terminal Fragment as Key to Control Potency and Resistance Mediated by the Binding Protein AlbA

Marcel Kulike-Koczula, Kay Hommernick, Leela B. Ghimire, Simone Kosol, Lieby Zborovsky, Maria Seidel, Nicole Sattler, Andi Mainz, John B. Weston, Dmitry Ghilarov, and Roderich D. Süssmuth*

Supporting Information

Table of Contents

1. *In vitro* Experiments 2

1.1 Determination of Minimal inhibitory concentrations (MIC) 2

1.2 *Escherichia coli* Gyrase Cleavage Inhibition Assay……………………………………..3

1.3 Resistance assessment 9

2. Synthesis of final derivatives 12

1. *In vitro* Assays

1.1 Determination of Minimal Inhibitory Concentrations (MIC)

Minimal inhibitory concentration (MIC) against selected bacterial strains values were determined according to the ninth edition of the Approved Standard M07-A9.[1] 20 µL of cryo stocks of bacterial strains were suspended in 20 mL LB medium (Lysogeny broth: 10 g/L peptone, 5 g/L yeast extract, 5 g/L NaCl). Following incubation overnight at 37°C, 200 rpm, the test inoculum was adjusted by 0.5 McFarland Standard in cation adjusted Mueller-Hinton broth (BBLTM Mueller-Hinton Broth II, Becton, Dickinson and Company, New Jersey, USA) to approximately $1x10^6$ CFU mL^{-1}. 95 µL of the bacterial inoculum were applied per well of a 96-well plate. Albicidin and albicidin derivatives **3**—**24** were dissolved in DMSO (100%) to an initial concentration of 2560 µg/mL and serially diluted in DMSO (100%). Initial solution and serial dilutions of Ciprofloxacin were prepared in 0.1 N HCL, respectively. 5 µL of each antibiotic dilution were added to the microdilution tray to reach albicidin concentrations of 128 µg/mL to 0.016 µg/mL per well. One row of each well plate served as a growth control without antibiotic substances and another row of the microdilution tray served as sterility control (only MHB II-media). The antimicrobial effect of the solvent (DMSO) was tested by adding 5 µL DMSO to several wells. Purity check and cell titer control were performed according to International Standard M07-A9 on Mueller-Hinton II Agar (Mueller Hinton II Broth, 15 g/L agar-agar). Both microdilution trays and agar plates were incubated at 30—37°C for 16 h and subsequently analyzed for growth inhibition by naked eye. The following primary panel of microorganisms were purchased from German Collection of Microorganisms and Cell Cultures (DSMZ):

Escherichia coli DSM 1116

Bacillus subtilis DSM 10

Micrococcus luteus DSM 1790

Mycobacterium phlei DSM 750

Salmonella typhimurium TA100
was kindly provided by Prof. Dr. Vera Meyer (Technische Universität Berlin).

Escherichia coli BW25113
was purchased as part of the single gene knockout library of *E. coli* K12 BW25113 (Keio collection) from Horizon Discovery group plc (Cambridge, UK).

1.2 *Escherichia coli* Gyrase Cleavage Inhibition Assay

E. coli gyrase cleavage assays were executed by setting up a master mix including 0.25 µg relaxed plasmid (pBR322) in 35 mM Tris-HCl (pH 7.5), 24 mM KCl, 4 mM $MgCl_2$, 2 mM dithithreitol (DTT), 1.8 mM spermidine, 1 mM ATP, 6.5% (wt/vol) glycerol and 0.1 mg/ml bovine serum albumin per reaction. Of this stock solution, 13.2 µL was aliquoted for each reaction into 1.5 mL Eppendorf tubes and supplemented with 0.3 µL of the appropriate compound dilution or DMSO with a albicidin DMSO concentration of 2% per reaction. The reactions were started with 1.5 µL of 50 nM (pre-screening) or 40 nM (IC_{50} determinations) wt *E. coli* gyrase in dilution buffer (50 mM Tris-HCl (pH 7.5), 100 mM KCl, 2 mM DTT, 1 mM EDTA, and 50% (wt/vol) glycerol) for a albicidin volume of 15 µL. Reactions were incubated at 37°C for 30 minutes before addition of 1.5 µL 0.2% (wt/vol) SDS and 0.75 µL proteinase K (0.2 mg/mL) and further incubation at 37°C for 30 minutes to trap cleavage complexes. Reactions were stopped with 15 µL chloroform:isoamyl alcohol (24: 1 v/v) and 15 µL of STEB (40% (wt/vol) sucrose, 100 mL Tris-Cl pH 8, 10 mM EDTA, 0.5 mg/mL bromophenol blue). After brief vortexing and centrifugation at 2,300g for 5 min, 15 µL of the aqueous phase was loaded onto 1% (wt/vol) agarose in TAE (Tris-acetate 0.04 mM and EDTA 0.002 mM) gels supplemented with 10 µg/mL ethidium bromide. The gels were run at 85 V for 2 hours.

DNA was visualized (602/50, UV Trans, auto optimal exposure) with a ChemiDoc MP (Bio-Rad Laboratories Inc., equipped with ImageLab Touch Software v2.0.0.27) and the percentage of cleaved DNA relative to the total amount of DNA per lane was determined with ImageJ software.[2] IC_{50} curves were generated in GraphPad Prism (v10.0.0) using the non-linear regression curve fit variable slope with four parameters and least squares regression.

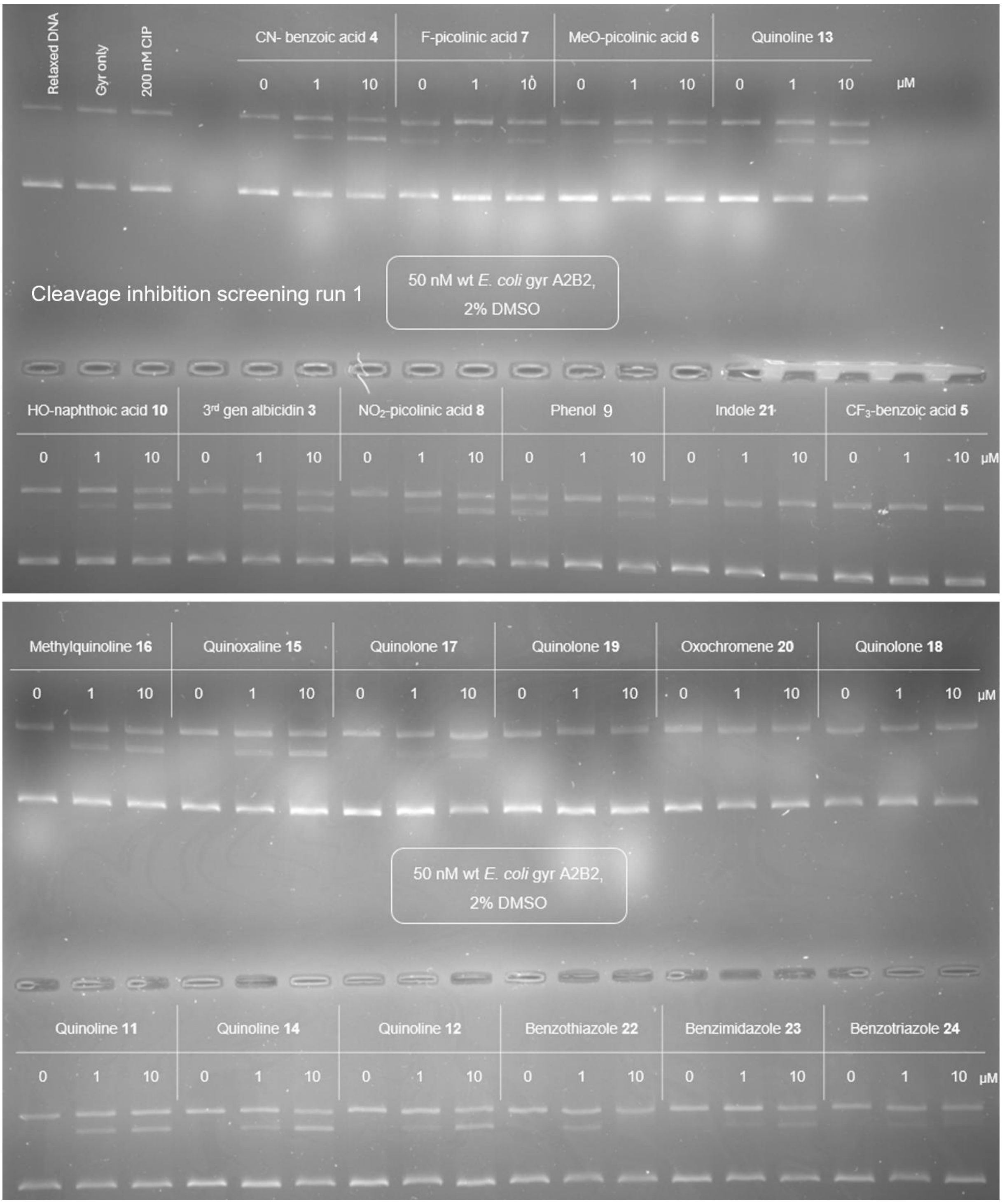

SI Figure 1: Screening run 1 for cleavage activity of WT *E. coli* gyrase in presence of various derivatives **3**–**24**. First three lanes (controls): relaxed DNA, WT *E. coli* gyrase and effect of ciprofloxacin (CIP, 200 µM) on WT gyrase activity, subsequent lanes: effect of increasing concentrations (0 µM, 1 µM and 10 µM) of derivatives **3**–**24** on WT gyrase activity. The upper band corresponds to nicked DNA, the middle band corresponds to linear DNA and the lower band corresponds to supercoiled DNA are indicated to the left of each gel.

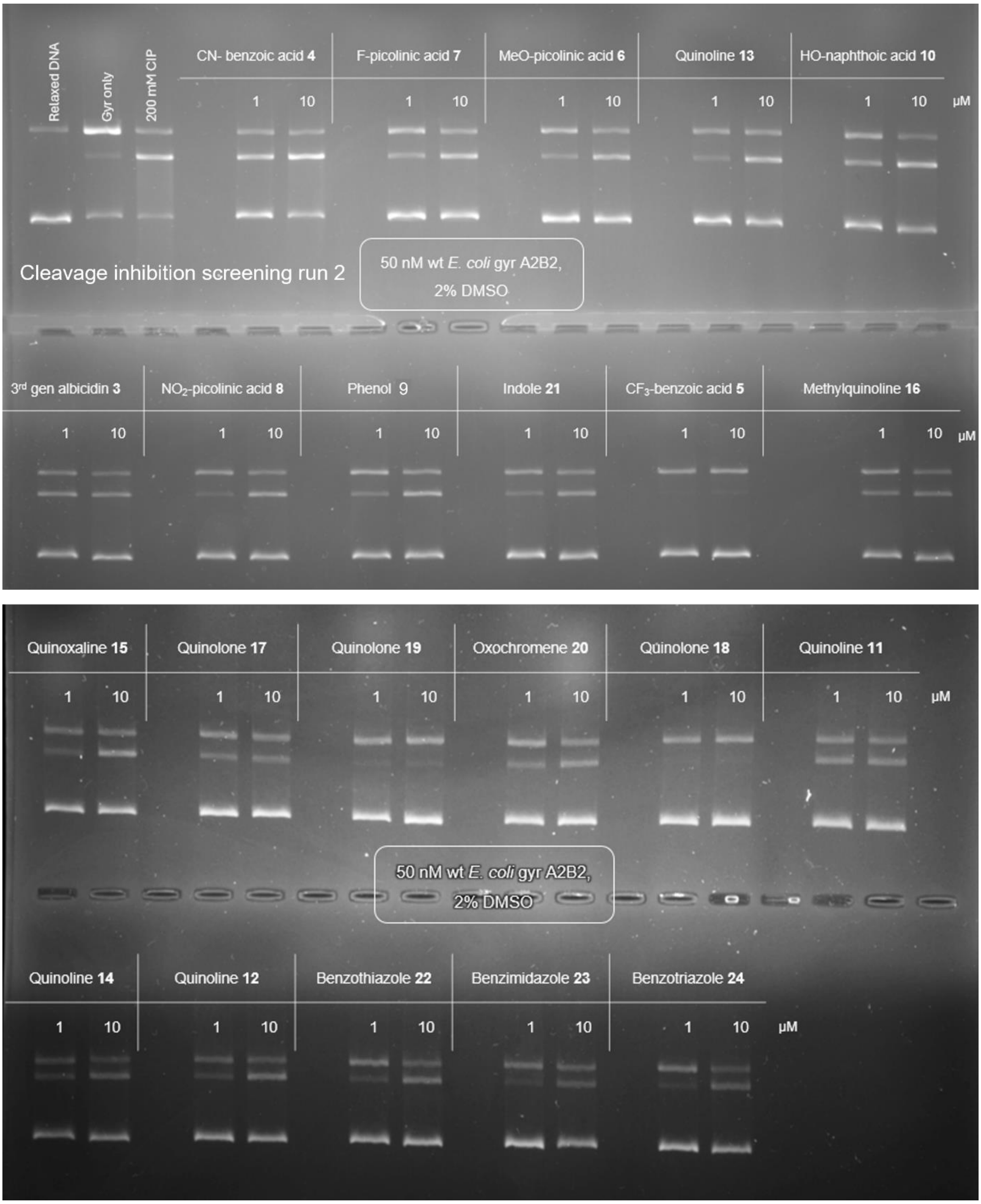

SI Figure 2: Screening run 2 for cleavage activity of WT *E. coli* gyrase in presence of various derivatives **3**—**24**. First three lanes (controls): relaxed DNA, WT *E. coli* gyrase and effect of ciprofloxacin (CIP, 200 µM) on WT gyrase activity, subsequent lanes: effect of increasing concentrations (0 µM, 1 µM and 10 µM) of derivatives **3**—**24** on WT gyrase activity. The upper band corresponds to nicked DNA, the middle band corresponds to linear DNA and the lower band corresponds to supercoiled DNA are indicated to the left of each gel.

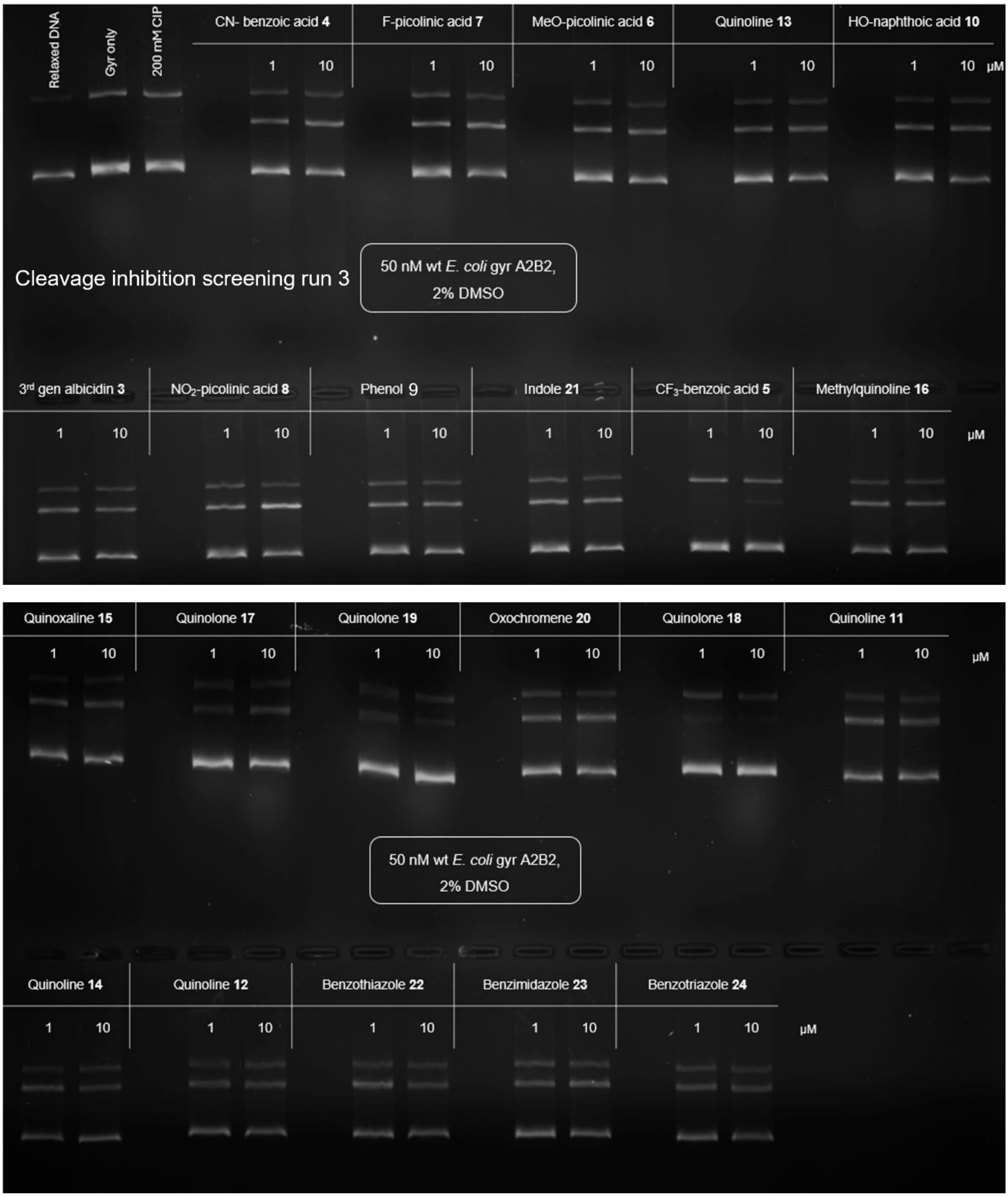

SI Figure 3: Screening run 3 for cleavage activity of WT *E. coli* gyrase in presence of various derivatives **3**−**24**. First three lanes (controls): relaxed DNA, WT *E. coli* gyrase and effect of ciprofloxacin (CIP, 200 µM) on WT gyrase activity, subsequent lanes: effect of increasing concentrations (0 µM, 1 µM and 10 µM) of derivatives **3**−**24** on WT gyrase activity. The upper band corresponds to nicked DNA, the middle band corresponds to linear DNA and the lower band corresponds to supercoiled DNA are indicated to the left of each gel.

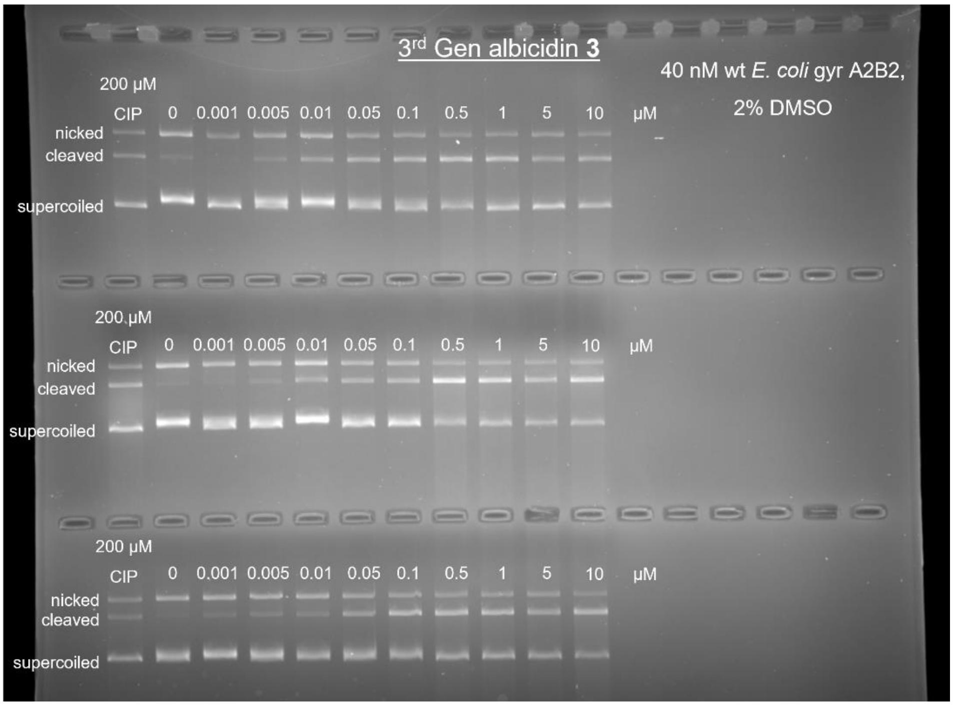

SI Figure 4: Cleavage activity of WT *E. coli* gyrase. First lane: effect of ciprofloxacin (CIP, 200 µM) on WT gyrase activity, subsequent lanes: effect of increasing concentrations (0.001 µM−10 µM) of 3rd Gen albicidin **3** on WT gyrase activity. Positions of nicked, linear and supercoiled DNA are indicated to the left of each gel.

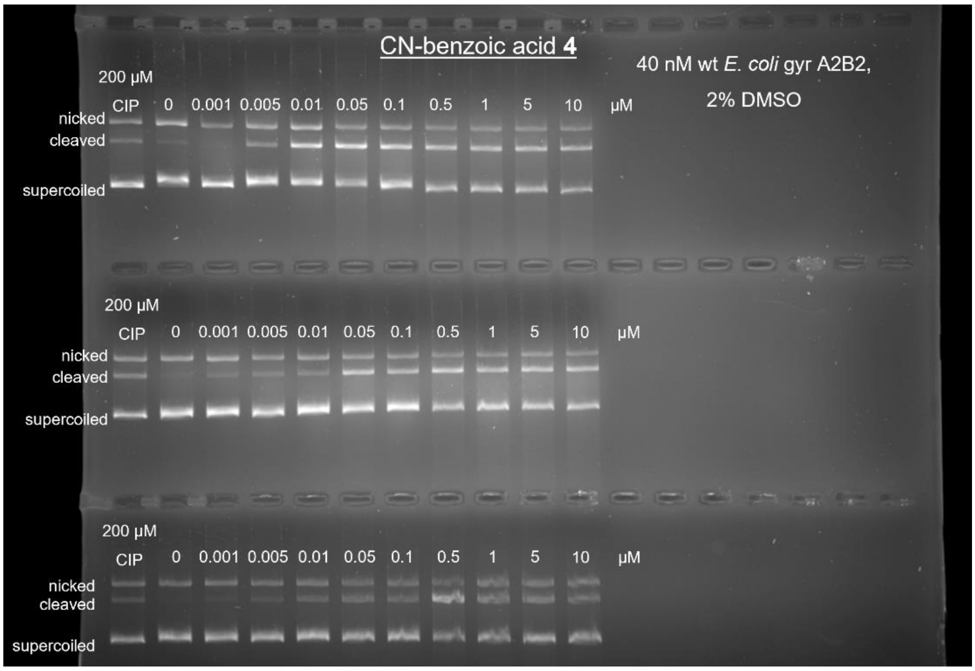

SI Figure 5: Cleavage activity of WT *E. coli* gyrase. First lane: effect of ciprofloxacin (CIP, 200 µM) on WT gyrase activity, subsequent lanes: effect of increasing concentrations (0.001 µM−10 µM) of cyano-benzoic acid **4** on WT gyrase activity. Positions of nicked, linear and supercoiled DNA are indicated to the left of each gel.

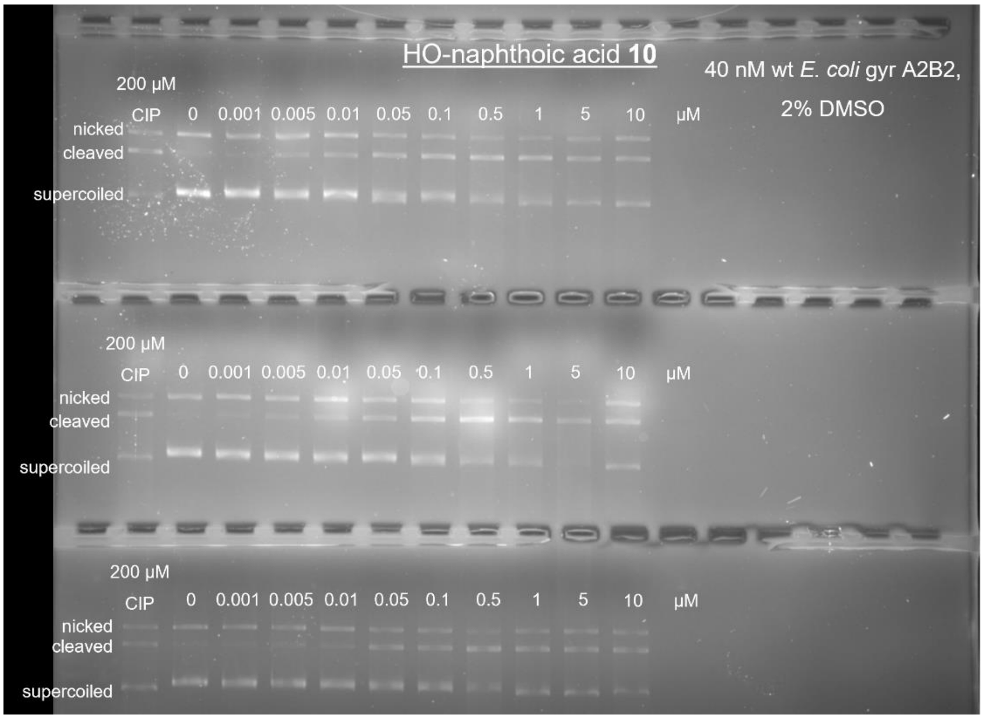

SI Figure 6: Cleavage activity of WT *E. coli* gyrase. First lane: effect of ciprofloxacin (CIP, 200 µM) on WT gyrase activity, subsequent lanes: effect of increasing concentrations (0.001 µM—10 µM) of hydroxy-naphthoic acid **10** on WT gyrase activity. Positions of nicked, linear and supercoiled DNA are indicated to the left of each gel.

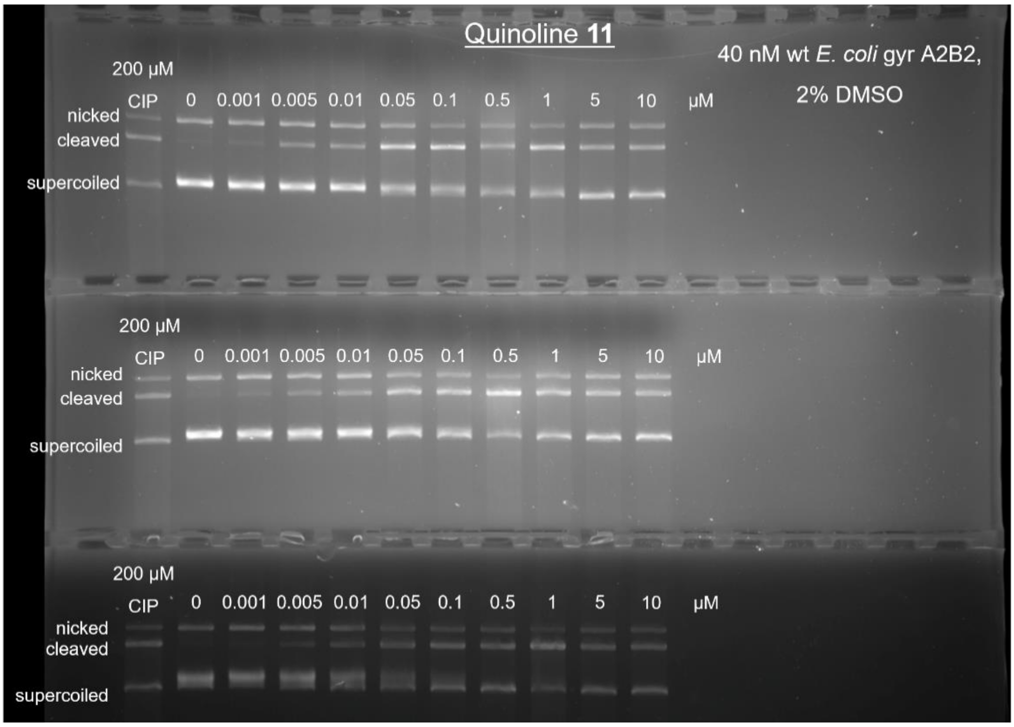

SI Figure 7: Cleavage activity of WT *E. coli* gyrase. First lane: effect of ciprofloxacin (CIP, 200 µM) on WT gyrase activity, subsequent lanes: effect of increasing concentrations (0.001 µM—10 µM) of quinoline **11** on WT gyrase activity. Positions of nicked, linear and supercoiled DNA are indicated to the left of each gel.

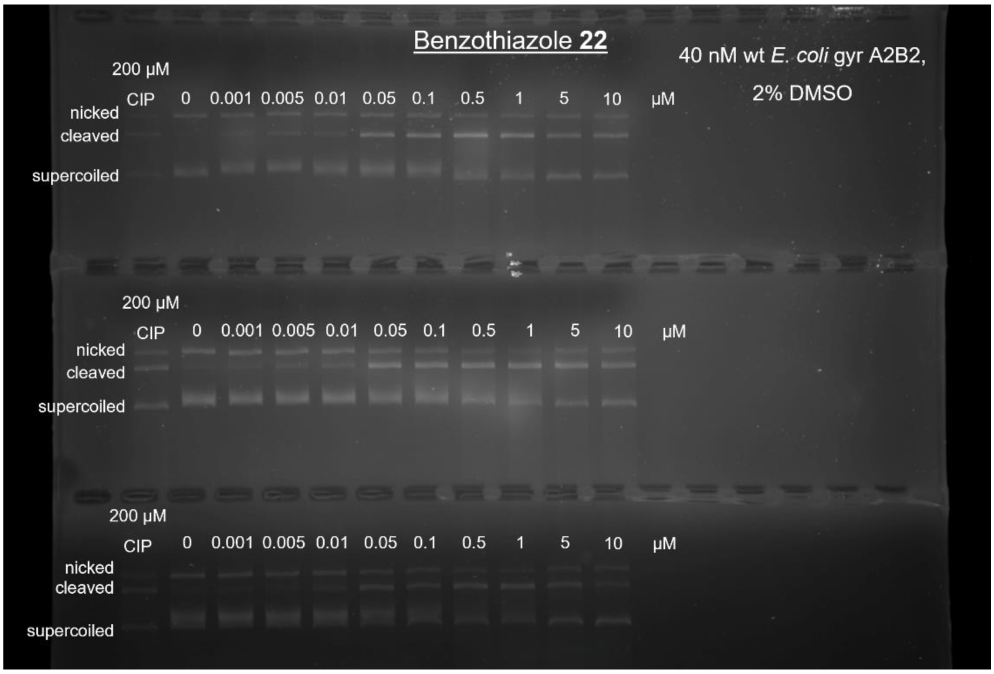

SI Figure 8: Cleavage activity of WT *E. coli* gyrase. First lane: effect of ciprofloxacin (CIP, 200 µM) on WT gyrase activity, subsequent lanes: effect of increasing concentrations (0.001 µM–10 µM) of benzothiazole **22** on WT gyrase activity. Positions of nicked, linear and supercoiled DNA are indicated to the left of each gel.

1.3 Resistance assessment against AlbA

E. coli strain DSM 1116 was used for all susceptibility testing. *E. coli* BL21 Star (DE3) cells (Invitrogen) were used for protein expression. LB medium (Lysogeny broth: 10 g/L peptone, 5 g/L yeast extract, 5 g/L NaCl) was used as broth or in agar plates for all experiments. Incubation steps took place at 37°C and 180 rpm shaking if not stated otherwise. Throughout the study, 2nd Gen albicidin **2** was used as albicidin standard due to its better stability, availability and similar activity compared to natural albicidin in presence of resistance factors.[3,4]

AlbA expression and purification

N-terminally His-tagged AlbA was expressed using a pET28a vector as described previously.[5] In brief, *E. coli* BL21(DE3) cells were transformed with pET28-AlbA plasmid and grown in LB medium with kanamycin. LB broth supplemented with 50 µg/mL kanamycin was inoculated from over-night cultures and grown at 37°C until an OD_{600} of 0.6 was reached. After induction with 0.2 mM isopropyl β-D-1-thiogalactopyranoside (IPTG) the proteins were expressed at 18°C for 20 h. The cells were harvested by centrifugation (4,000 x G, 20 min), resuspended in lysis buffer (50 mM Tris, pH 8.0, 500 mM NaCl, 10% glycerol) and processed in a cell disruptor (Constant Systems Ltd) at 25 kPsi before centrifugation at 50,000 x G, at 4°C for 45 min. The lysate was incubated with Pure Cube Ni-NTA agarose (Cube Biotech, Germany) at 4°C for 20 min while shaking. The slurry was transferred to empty gravity-flow columns (ThermoFisher) and after a washing step with lysis buffer, the protein was eluted in two steps with 250 mM imidazole and 500 mM imidazole in the lysis buffer, respectively. His_6-AlbA was buffer exchanged on PD10 Desalting Columns in assay buffer (50 mM sodium phosphate, pH 7.0, 100 mM

NaCl) and the protein concentration weas determined with a nano-photometer P330 (Implen, Munich, Germany) spectrophotometer using extinction coefficients calculated with the ProtParam tool[6] before storage at -80°C.

E. coli growth inhibition assay

To test the antibacterial activity of albicidin derivatives **2** and **4**–**24** in presence and absence of AlbA, susceptibility assays in liquid culture were conducted. An overnight culture of *E. coli* DSM 1116 was used to inoculate 50 mL LB which was grown to an OD ~1.0. The culture was diluted in LB medium to using 0.5 McFarland standard (1.5 x 10^8 cells/mL) and added to a clear round bottom 96-well plate (microdilution tray) with LB to obtain 1 x 10^6 cells/mL. Each well contained 85 µL inocolum to which either 10 µL AlbA (albicidin concentration 10 µM) or buffer (50 mM sodium phosphate pH 7.0, 100 mM NaCl) were added. The albicidin derivatives were dissolved in 100% DMSO and 5 µL were added to the wells to obtain a albicidin concentration of 10 µM compound and 5% (v/v) DMSO in a albicidin culture volume of 100 µL. All assays were set up in triplicates. The plates were incubated at 37°C in the dark without shaking for 20 h before documentation using a biostep scanner (Epson) with argusX.

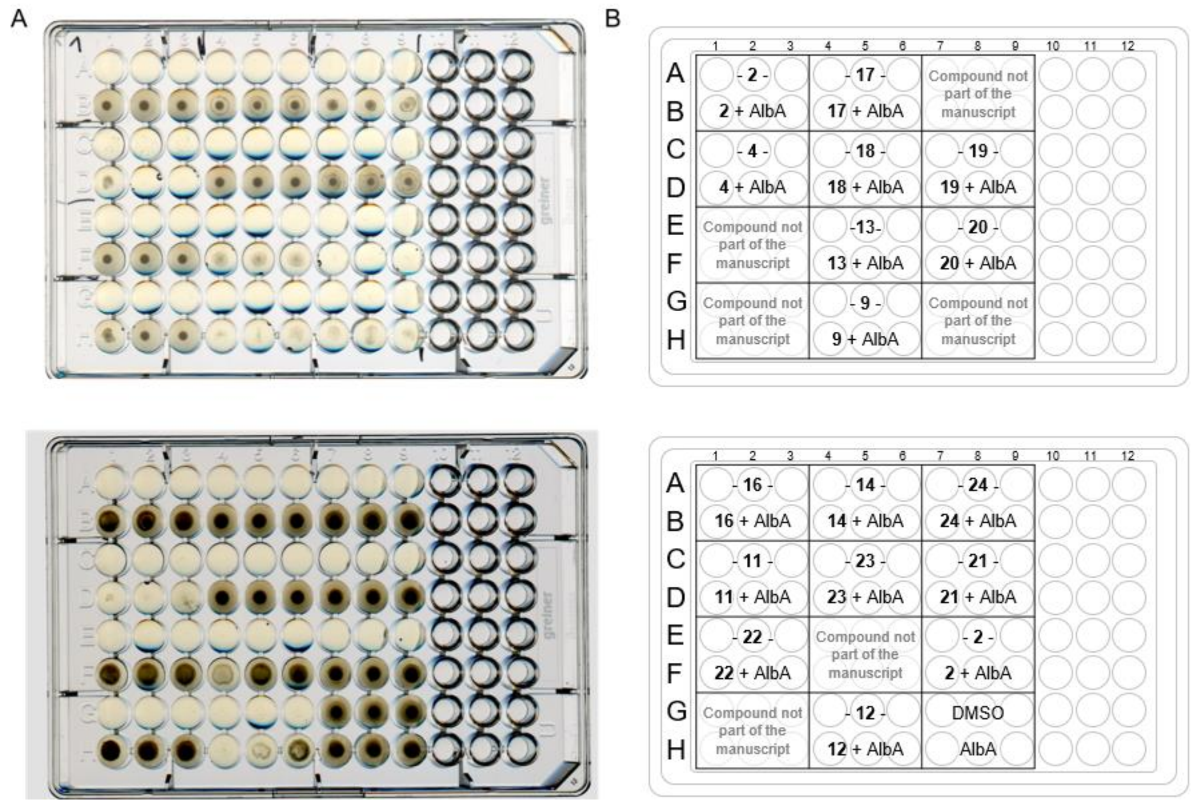

SI Figure 9: Growth inhibition assay with *E. coli* with albicidin derivatives in presence or absence of AlbA. A) Scan of liquid cultures of *E. coli* in 96-well plates and B) schematic assignment of the respective wells. All experiments were run in triplicates (columns 1–3, 4–6, 7–9). Upper rows contain 10 µM of the indicated derivative or DMSO without AlbA (rows A, C, E, G) and lower rows contain 10 µM of the indicated derivative with AlbA (rows B, D, F, H). Compounds that are not part of the manuscript are not specified.

Transcription activation (TA) assay

Transcription reporter assays were conducted as described previously.[4] In short, *E. coli* BL21(DE3) were transformed with pET15b-AlbA, an expression construct without His-tag, and pCS-pAlbA-ilux, a reporter vector containing the pAlbA promoter and the improved bacterial luciferase reporter gene cassette ILUX.[7] An over-night culture was grown in LB medium with kanamycin and ampicillin to inoculate three 50 mL cultures of BL21(DE3):pET15b-AlbA + pCS-pAlbA-ilux in LB medium with 50 µg/ml kanamycin and 100 µg/ml ampicillin. The cultures were grown to OD_{600} 0.4-0.6 and transferred to white, clear bottom 96-well plates (Corning Inc.). To 200 µL culture in each well, 1 µL DMSO with albicidin or one of its derivatives were added. Of albicidin derivatives, a 1.5 µM or 2.0 µM albicidin concentration was added to measure transcription activation. Sixty data points were measured on a TECAN Infinite 200 microplate reader, after orbital shaking with for 10 min, with an amplitude of 3 mm, and a wait time of one minute between each measurement. At each time point, the absorbance at 600 nm (9 mm bandwidth and 25 flashes) and the luminescence (500 ms integration time, no attenuation) were measured. Each assay was measured in triplicate at 30°C. The luminescence data was normalized to the OD before the background signal (wells with no albicidin added) was subtracted and the mean + standard deviation of each measurement point was calculated. Each measurement series was normalized to 2nd Gen albicidin **2** as a standard, yielding relative luminescence curves of which the maximum within the first four hours of measurement was extracted to compare the luminescence output for each compound.

2. Synthesis of albicidin derivatives

Albicidin coupling protocol A

HATU (1.3 eq.) was added to a solution of AB building block (1.3 eq.) and DIPEA (6 eq.) in anhydrous DMF (1 mL) and the resulting reaction mixture was stirred for 1 h at room temperature. Tetrapeptide[8] (**26,** 1.0 eq.) was added and the solution was stirred for 16 h at room temperature. After full conversion an aqueous solution of 3 M KOH (1 mL) was added and the reaction mixture was stirred for 30 min at room temperature. An aqueous solution of HCl (3 M, 1 mL) was added and the resulting suspension was freeze-dried. The crude material was dissolved in DMSO, centrifuged, and the supernatant purified by HPLC (PLRP-S column, CH_3CN in H_2O). The albicidin derivatives were obtained as solids after freeze-drying.

Synthesis of albicidin derivative (**4**)

Methyl 4-(4-(cyano)benzamido)benzoate (**4a**).

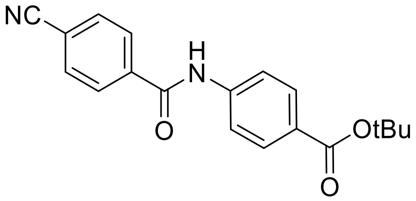

4-(Cyano)benzoic acid (**4a**, 5.00 g, 34.0 mmol, 1.00 eq.) was suspended in toluene (100 mL) and thionyl chloride (20.2 g, 12.3 mL, 131 mmol, 5.00 eq.) was added. The reaction mixture was heated to 80 °C and stirred overnight. The volatiles were removed under reduced pressure and the residue was taken up in dry THF (30 mL). To the solution was added *t*-butyl 4-aminobenzoate (**25a**, 6.57 g, 34.0 mmol, 1.00 eq.) and triethylamine (10.3 g, 14.2 mL, 102 mmol, 3.00 eq.) and the reaction mixture was stirred overnight at room temperature. The volatiles were evaporated under reduced pressure and the residue was washed with EtOAc. *t*-Butyl 4-(4-(cyano)benzamido)benzoate (**4b**, 10.9 g, 34.0 mmol, 100%) was obtained as a white solid.
^{1}H NMR (DMSO-d_6, 400 MHz): δ [ppm] = 10.78 (s, 1 H), 8.13 (d, J = 8.6 Hz, 2 H), 8.05 (d, J = 8.6 Hz, 2 H), 7.92 (s, 4 H), 1.55 (s, 9 H). **^{13}C NMR** (DMSO-d_6, 176 MHz): δ [ppm] = 165.0, 143.3, 139.1, 133.0, 130.4, 129.1, 127.0, 120.1, 118.7, 114.6, 80.6, 28.3. **HRMS** (ESI): m/z calculated for $C_{19}H_{19}N_2O_3$ $[M+H]^+$ 323.1390; found 323.1384.

4-(4-(Cyano)benzamido)benzoate (**4c**).

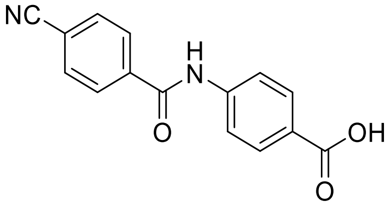

t-Butyl 4-(4-(cyano)benzamido)-benzoate (**4b**, 5.58 g, 17.3 mmol, 1.00 eq.) was dissolved in DCM/TFA (10:1, 33 mL) and the mixture was stirred for 4 h. The reaction mixture was coevaporated with MeOH (5 × 10 mL) under reduced pressure and washed with EtOAc and pentane. 4-(4-(cyano)benzamido)-benzoic acid (**4c**, 4.38 g, 16.4 mmol, 95%) was obtained as a white solid.

^{1}H NMR (DMSO-*d*$_6$, 400MHz): δ [ppm] = 12.79 (br. s, 1 H), 10.78 (s, 1 H), 8.13 (d, *J* = 8.4 Hz, 2 H), 8.04 (d, *J* = 8.4 Hz, 2 H) 7.98 – 7.90 (m, 4 H). **^{13}C NMR** (DMSO-*d*$_6$, 176 MHz): δ [ppm] =166.8, 164.6, 142.8, 138.6, 132.5, 130.2, 128.6, 125.9, 119.6, 118.2, 114.0. **HRMS** (ESI): *m/z* calculated for $C_{15}H_9N_2O_3$ [*M*-H]$^-$ 265.0619; found 265.0614.

Albicidin derivative (**4**)

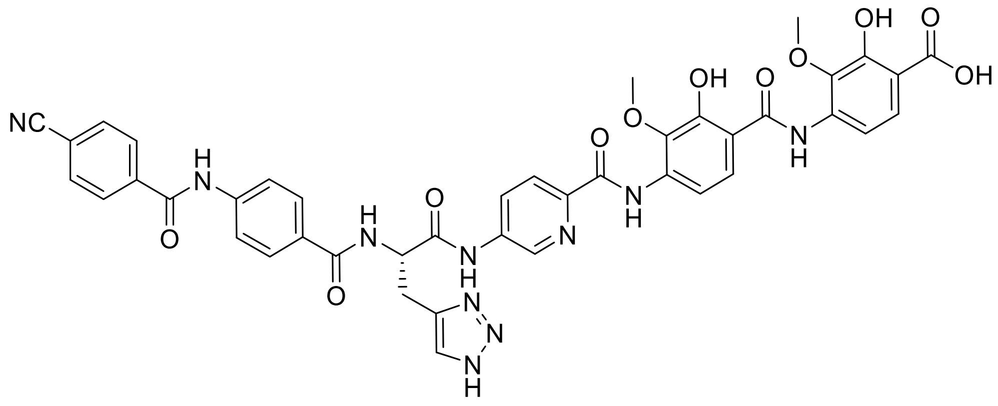

The synthesis of the product was conducted according to coupling protocol A. The albicidin derivative (**4**) was obtained as a colourless solid (22 mg, 37 µmol, 25% over two steps).

^{1}H NMR (DMSO-*d*$_6$, 700 MHz): δ [ppm] = 11.72 (s, 1 H), 11.13 (s, 1 H), 10.85 (s, 1 H), 10.71 (s, 1 H), 10.50 (s, 1 H), 8.98 (d, *J* = 2.5 Hz, 1 H), 8.83 (d, *J* = 6.9 Hz, 1 H), 8.34 (dd, *J* = 2.3, 8.6 Hz, 1 H), 8.21 (d, *J* = 8.5 Hz, 1 H), 8.15 – 8.10 (m, 3 H), 8.13 (d, *J* = 8.5 Hz, 2 H), 8.11 (d, J = 8.9 Hz, 1 H), 8.05 (d, *J* = 8.5 Hz, 2 H), 8.03 (d, *J* = 8.8 Hz, 1 H), 7.93 (d, *J* = 8.9 Hz, 2 H), 7.89 (d, *J* = 6.3 Hz, 2 H), 7.88 (d, J = 6.0 Hz, 1 H), 7.67 (s, 1 H), 7.60 (d, *J* = 8.8 Hz, 1 H), 4.95 (dd, 1 H), 3.93 (s, 3 H), 3.88 (s, 3 H), 3.34 (dd, *J* = 5.6, 14.6 Hz, 1 H), 3.27 (dd, *J* = 9.2, 14.6 Hz, 1 H). **^{13}C NMR** from HSQC-ed (DMSO-*d*$_6$), 176 MHz): δ [ppm] = 140.0, 140.0, 133.0, 133.0, 129.1, 129.0, 128.9, 127.8, 127.1, 126.1, 123.5, 120.0, 110.8, 110.9, 61.3, 60.7, 54.8. **HRMS** (ESI): *m/z* calculated for $C_{42}H_{35}N_{10}O_{11}$ [*M*+H]$^+$ 855.2481; found 855.2465, t_R = 8.20 min.

Synthesis of albicidin derivative (**5**)

Methyl 4-(4-(trifluoromethyl)benzamido)benzoate (**5b**).

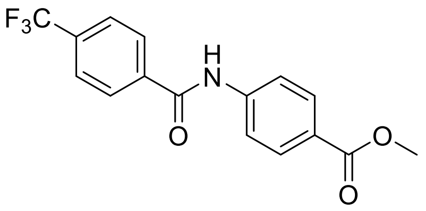

4-(Trifluoromethyl)benzoate (**5a**, 5.00 g, 26.3 mmol, 1.00 eq.) was suspended in toluene (165 mL) and thionyl chloride (9.54 mL, 15.6 g, 131 mmol, 5.00 eq.) was added. The reaction mixture was heated to 115 °C and stirred overnight. The volatiles were removed under reduced pressure and the residue was taken up in dry THF (30 mL). To the solution was added methyl 4-aminobenzoate (**25b**, 4.37 g, 28.9 mmol, 1.10 eq.) and triethylamine (5.85 g, 8.06 mL, 57.9 mmol, 2.2 eq.) and the reaction mixture was stirred overnight at room temperature. The reaction mixture was filtered, the precipitate was washed with M*t*BE and H_2O and dried on the filter. Methyl 4-(4-(trifluoromethyl)benzamido)benzoate (**5b**, 8.25 g, 25.5 mmol, 97%) was obtained as a white solid.

^{1}H NMR (DMSO-*d*$_6$, 400MHz): δ [ppm] = 10.90 (s, 1 H), 8.19 (d, *J* = 8.2 Hz, 2 H), 7.98 (s, 4 H), 7.93 (d, *J* = 8.2 Hz, 2 H), 3.85 (s, 1 H). **^{13}C NMR** (DMSO-*d*$_6$, 176 MHz): δ [ppm] = 143.3, 138.4, 135.3, 130.1, 128.8, 125.4, 124.6, 123.8, 119.8, 51.9. **HRMS** (ESI): *m/z* calculated for $C_{16}H_{12}F_3NO_3$ [*M*+H]$^+$ 324.0842; found 324.0841.

4-(4-(Trifluoromethyl)benzamido)benzoic acid (**5c**).

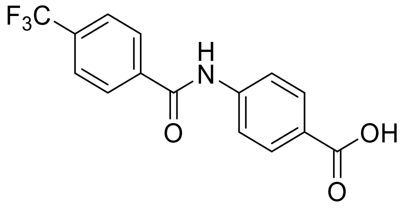

Methyl 4-(4-(trifluoromethyl)benzamido)-benzoate (**5b**, 5.00 g, 15.9 mmol, 1.00 eq.) was dissolved in THF (20 mL) and MeOH (20 mL). To the solution was added an aqueous solution of KOH (5 M, 20 mL) and the mixture was stirred overnight. The reaction mixture was concentrated under reduced pressure and the residue was treated with an aqueous solution of HCl (6 M, 20 mL). The precipitate was filtrated, washed by an aqueous solution of HCl (1 M) and dried. 4-(4-(Trifluoromethyl)benzamido)benzoate (**5c**, 4.92 g, 15.9 mmol, 100%) was obtained as a white solid.

^{1}H NMR (DMSO-*d*$_6$, 400MHz): δ [ppm] = 12.84 (br. S, 1 H), 10.75 (s, 1 H), 8.17 (d, *J* = 8.1 Hz, 2 H), 7.98 – 7.91 (m, 6 H). **^{13}C NMR** (DMSO-*d*$_6$, 176 MHz): δ [ppm] = 167.4, 165.3, 143.4, 138.9, 130.7, 129.2, 126.3, 125.9, 125.9, 120.1. **HRMS** (ESI): *m/z* calculated for $C_{15}H_9F_3NO_3$ [*M*-H]$^-$ 308.0540; found 308.0533.

Perchlorophenyl 4-(4-(trifluoromethyl)benzamido)benzoate (**5d**).

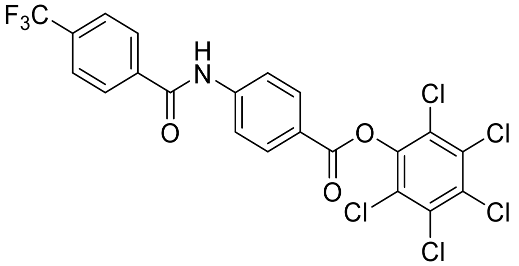

4-(4-(Trifluoromethyl)-benzamido)benzoate (**5c**, 2.00 g, 6.47 mmol, 1.00 eq.) was dissolved in dry DMF (20 mL) and pentachlorophenol (1.89 g, 7.11 mmol, 1.10 eq.), DMAP (79.0 mg, 0.647 mmol, 0.100 eq.) and EDC (1.49 g, 7.76 mmol, 1.20 eq.) were added and the reaction mixture was stirred overnight at room temperature. To the reaction mixture was added EtOAc (200 mL) and the organic phase was washed with an aqueous solution of HCl (1 M, 3 × 200 mL) and a saturated aqueous solution of NaCl (200 mL), dried over $MgSO_4$, filtered and concentrated under reduced pressure. Purification of the crude product by column chromatography on silica gel (SiO_2, *n*-hexane/EtOAc, 9:1–4:1) afforded the desired product **18** (3.03 g, 5.43 mmol, 84%) as a white solid.

^{1}H NMR (DMSO-*d*$_6$, 400MHz): δ [ppm] = 10.95 (s, 1 H), 8.23 (d, *J* = 9.0 Hz, 2 H), 8.20 (d, *J* = 8.3 Hz, 2 H), 8.10 (d, *J* = 9.0 Hz, 2 H), 7.95 (d, *J* = 8.5 Hz, 2 H). **^{13}C NMR** (DMSO-*d*$_6$, 176 MHz): δ [ppm] = 165.9, 165.0, 161.7, 145.1, 144.1, 138.1, 131.6, 131.3, 130.8, 128.8, 125.5, 125.4, 121.0, 120.1. **HRMS** (ESI): *m/z* calculated for $C_{21}H_{10}{}^{35}Cl_5F_3NO_3$ $[M+H]^+$ 555.9050; found 555.9037.

Albicidin derivative (**5**)

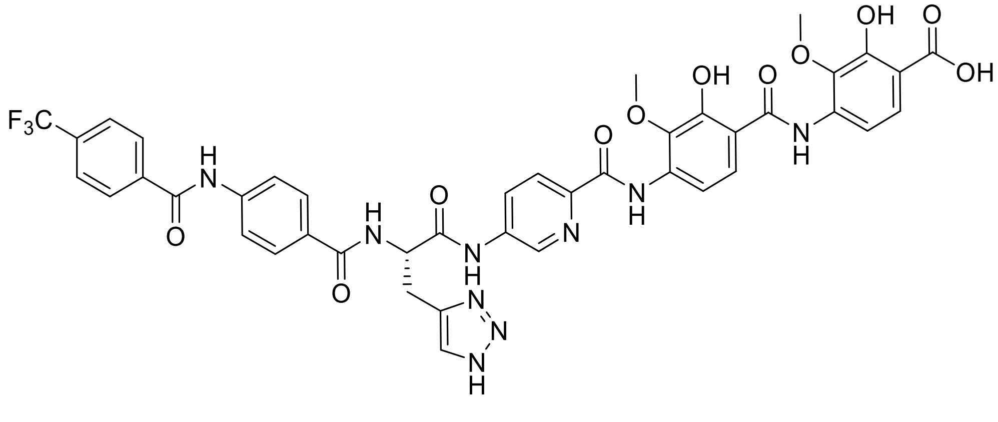

Perchlorophenyl 4-(4-(trifluoromethyl)benzamido)benzoate (**5d**, 69.6 mg, 125 µmol, 1.50 mmol) and Tetrapeptide (**26**, 60.0 mg, 83.2 µmol, 1.00 equiv.) were dissolved in dry DMF (1 mL) and TEA (42.1 mg, 58.0 µL, 416 µmol, 5.00 eq.) was added. The reaction mixture was stirred overnight at room temperature. To the reaction mixture was added 3 M KOH (1 mL) and the reaction mixture was stirred for 20 min. To the reaction mixture was added 3 M HCl (1 mL), diluted by H_2O and lyophilized. The resulting material

was dissolved in DMSO and purified by HPLC (PLRP-S, CH_3CN in H_2O). The albicidin product (**5**, 17.0 mg, 18.9 µmol, 23%) was obtained as white solid.

^{1}H NMR (500 MHz, DMSO-*d*$_6$) δ [ppm] = 11.73 (s, 1 H), 11.14 (s, 1 H), 10.87 (s, 1 H), 10.50 (s, 1 H), 8.98 (d, *J* = 2.5 Hz, 1 H), 8.84 (d, *J* = 7.5 Hz, 1 H), 8.35 (dd, *J* = 8.5, 1 H), 8.18 (d, *J* = 8.6 Hz, 2 H), 8.12 (d, *J* = 8.9 Hz, 1 H), 8.04 (d, *J* = 8.9 Hz, 1 H), 7.96 – 7.87 (m, 6 H), 7.71 (s, 1 H), 7.60 (d, *J* = 8.8 Hz, 1 H), 4.95 (q, *J* = 7.5 Hz, 1 H), 4.60 (s, 2 H), 3.93 (s, 3 H), 3.88 (s, 3 H), 3.37 – 3.25 (m, 2 H). **^{13}C NMR** from HSQC-ed (DMSO-*d*$_6$, 126 MHz): δ [ppm] = 140.0, 129.3, 128.9, 127.8, 127.0, 126.2, 125.9, 123.5, 119.9, 110.9, 110.7, 61.3, 60.6, 54.7, 27.6. **HRMS** (ESI): m/z calculated for $C_{42}H_{38}N_9O_{13}$: 876.2784, found: 876.2570, t_R = 8.99 min.

Synthesis of albicidin derivative (**6**)

Methyl 4-(5-methoxypicolinamido)benzoate (**6b**)

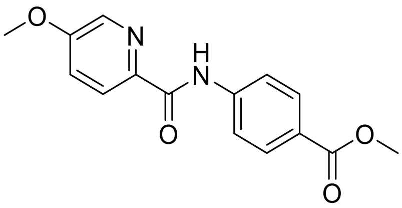

5-Methoxypicolinic acid (**6a**, 500 mg, 3.54 mmol, 1.00 eq.) was dissolved in $SOCl_2$ (5 mL). The reaction mixture was stirred at 100°C for 2 h, after which all volatiles were evaporated under reduced pressure. The residue was dissolved in of dry THF (5 mL) and added to a solution of methyl 4-aminobenzoate (**25b**, 590 mg, 3.90 mmol, 1.10 eq.) and DIPEA (1.85 mL, 1.37 g, 10.6 mmol, 3.00 eq.) in dry THF (5 mL). The reaction mixture was stirred overnight at room temperature, after which an aqueous solution of HCl (1 M, 10 mL) was added. The aqueous layer was separated and extracted with EtOAc (3 × 20 mL). The combined organic fractions were washed with an aqueous solution of $NaHCO_3$ (1 M, 20 mL) and brine (20 mL), dried over Na_2SO_4 and concentrated under reduced pressure. Purification of the crude product by flash column chromatography (SiO_2, *n*-hexane/EtOAc, 9:1 – 4:1) afforded methyl 4-(5-methoxypicolinamido)benzoate (**6b**, 550 mg, 2.01 mmol, 56%) as a white solid.

^{1}H NMR (DMSO-*d*$_6$, 400MHz): δ [ppm] = 10.52 (s, 1 H), 7.96 (d, *J* = 8.8 Hz, 2 H), 7.87 (d, *J* = 8.8 Hz, 2 H), 7.68 (t, *J* = 8.7 Hz, 1 H), 6.99 (dd, *J* = 12.5, 2.3 Hz, 1 H), 6.92 (dd, *J* = 8.7, 2.3 Hz, 1 H), 3.85 (s, 3 H), 3.84 (s, 3 H). **^{13}C NMR** (DMSO-*d*$_6$, 176 MHz): δ [ppm] = 166.3, 163.4, 163.2, 161.9, 159.9 143.9, 131.7, 130.7, 124.8, 119.6, 116.9, 111.1, 102.5, 56.5, 52.4. **HRMS** (ESI): *m/z* calculated for $C_{15}H_{15}N_2O_4$ $[M+H]^+$ 287.1026; found 287.1023.

4-(5-Methoxypicolinamido)benzoic acid (**6c**)

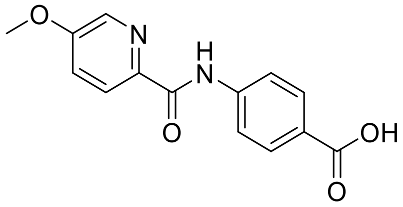

Methyl 4-(5-methoxypicolinamido)benzoate (**6b**, 500 mg, 2.01 mmol, 1.00 eq.) was dissolved in MeOH (10 mL), cooled to 0°C and an aqueous solution of KOH (4 M, 2 mL) was added. The reaction was stirred for 30 minutes, then quenched with an aqueous solution of HCl (6 M, 2 mL). The product precipitated as a white solid that was separated by centrifugation to yield of target compound (**6c**, 420 mg, 1.54 mmol, 84% yield).

^{1}H NMR (500 MHz, DMSO-d_6) δ [ppm] = 12.74 (s, 1 H), 10.74 (s, 1 H), 8.40 (d, J = 2.9 Hz, 1 H), 8.15 (d, J = 8.6 Hz, 1 H), 8.03 (d, J = 8.9 Hz, 2 H), 7.92 (d, J = 8.6 Hz, 1 H), 7.63 (dd, J = 8.8, 2.9 Hz, 1 H), 3.94 (s, 3 H). **^{13}C NMR** (DMSO-d_6, 176 MHz): δ [ppm] = 166.9, 162.7, 158.0, 142.6, 142.0, 136.6, 130.1, 125.7, 124.1, 121.1, 119.4, 56.0. **HRMS** (ESI): *m/z* calculated for $C_{14}H_{13}N_2O_4$ [*M*+H]$^+$ 273.0870; found 273.0862.

Albicidin derivative (**6**)

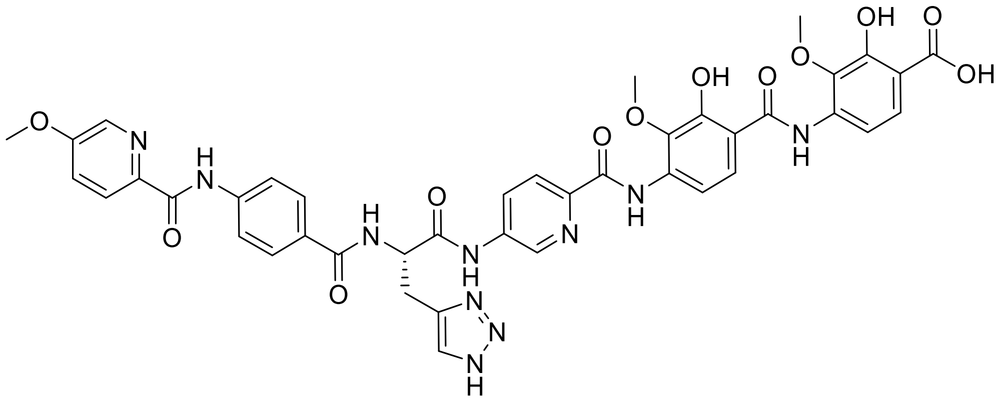

The synthesis of the product was conducted according to coupling protocol A. The albicidin derivative (**8**) was obtained as a colourless solid (8 mg, 17% over two steps). **^{1}H NMR** (DMSO-d_6, 700 MHz): δ [ppm] = 14.67 (s, 1 H), 11.75 (s, 1 H), 11.06 (s, 1 H), 10.88 (s, 1 H), 10.70 (s, 1 H), 10.50 (s, 1 H), 8.98 (d, J = 2.4 Hz, 1 H), 8.83 (d, J = 7.7 Hz, 1 H), 8.41 (d, J = 2.9 Hz, 1 H), 8.35 (dd, J = 8.6, 2.3 Hz, 2 H), 8.21 (d, J = 8.5 Hz, 1 H), 8.16 (d, J = 8.7 Hz, 1 H), 8.11 (d, J = 8.9 Hz, 1 H), 8.02 (d, J = 8.9 Hz, 2 H), 7.90 (d, J = 8.5 Hz, 2 H), 7.88 (d, J = 8.9 Hz, 1 H), 7.67 (s, 1 H), 7.65 (dd, J = 2.8, 8.8 Hz, 1 H), 7.56 (d, J = 9.1 Hz, 1 H), 4.94 (dd, J = 8.9 Hz, 1 H), 3.95 (s, 3 H), 3.91 (s, 3 H), 3.88 (s, 3 H). **^{13}C NMR** from HSQC-ed (DMSO-d_6, 176 MHz): δ [ppm] = 140.0, 137.1, 133.4, 128.8, 127.7, 126.8, 125.9, 124.5, 123.5, 121.6, 119.7, 110.6, 54.8, 56.5, 54.0. 60.5, 61.3. **HRMS** (ESI): m/z calculated for $C_{41}H_{37}N_{10}O_{12}$ [M+H]$^+$ 861.2587; found 861.2604, t_R = 8.29 min.

Synthesis of albicidin derivative (**7**)

Methyl 4-(5-fluoropicolinamido)benzoate (**7b**)

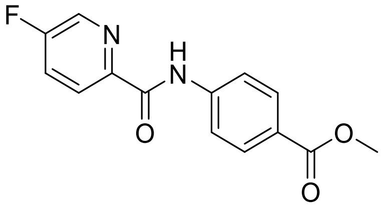

5-Fluoropicolinic acid (**7a**, 500 mg, 3.54 mmol, 1.00 eq.) was dissolved in $SOCl_2$ (2 mL). The reaction mixture was heated to reflux and stirred for 2 h. After that, all volatiles were evaporated. The resulting solid was dissolved in dry THF (5 mL) and added to a solution of methyl 4-aminobenzoate (**25b**, 590 mg, 3.91 mmol, 1.10 eq.) and DIPEA (500 µL) in dry THF (5 mL). The reaction was stirred overnight and quenched with an aqueous solution of HCl (1 M, 10 mL). The aqueous layer was separated and extracted with EtOAc (3 × 10 mL). The combined organic fractions were washed with an aqueous solution of 1 M $NaHCO_3$ (10 mL) and brine (10 mL), dried over anhydrous Na_2SO_4 and concentrated under reduced pressure. Purification of the crude product by column chromatography on silica gel (*n*-hexane/EtOAc, 9:1 – 4:1) afforded the methyl 4-(5-fluoropicolinamido)benzoate (**7b**, 550 mg, 2.01 mmol, 56%) as a white solid.

^{1}H NMR (500 MHz, DMSO-d_6) δ [ppm] = 10.92 (s, 1 H), 8.75 (d, *J* = 2.8 Hz, 1 H), 8.25 (dd, *J* = 8.8, 4.6 Hz, 1 H), 8.07 (d, *J* = 8.9 Hz, 2 H), 8.01 (dd, *J* = 2.7, 8.6 Hz, 1 H), 7.96 (d, *J* = 8.9 Hz, 2 H), 3.84 (s, 3 H). **^{13}C NMR** (DMSO-d_6, 176 MHz): δ [ppm] = 165.77, 162.23, 162.05, 159.65, 146.27, 142.75, 137.01, 136.76 (d), 130.02, 125.00, 124.94, 124.80, 124.61, 119.77, 51.92. **HRMS** (ESI): *m*/*z* calculated for $C_{14}H_{12}FN_2O_3$ $[M+H]^+$ 275.0826; found 275.0821.

4-(5-fluoropicolinamido)benzoate (**7c**)

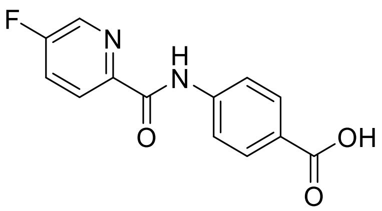

Methyl 4-(5-fluoropicolinamido)benzoate (**7b**, 500 mg, 1.82 mmol, 1.00 eq.) was dissolved in THF (10 mL), cooled to 0°C and an aqueous solution of KOH solution (4 M, 2 mL) was added. The reaction was stirred for 30 minutes and quenched with an aqueous solution of HCl (6 M, 2 mL). The product precipitated as a white solid that was separated by centrifugation to yield target compound (**7c**, 385 mg, 1.48 mmol, 81% yield).

^{1}H NMR (500 MHz, DMSO-d_6) δ [ppm] = 10.86 (s, 1 H), 8.75 (d, *J* = 2.8 Hz, 1 H), 8.26 (dd, *J* = 4.5, 8.7 Hz, 1 H), 8.03−7.97 (m, 3 H), 7.93 (d, *J* = 8.6 Hz, 2 H). **^{13}C NMR** (DMSO-d_6, 176 MHz): δ [ppm] = 167.1, 162.2, 161.9, 159.6, 146.4, 141.9, 137.0, 136.7, 130.1, 127.0, 125.0, 124.9, 124.9, 124.8, 119.6. **HRMS** (ESI): m/z calculated for $C_{13}H_{10}FN_2O_3$ $[M+H]^+$ 261.0670; found 261.0667.

Albicidin derivative (**7**)

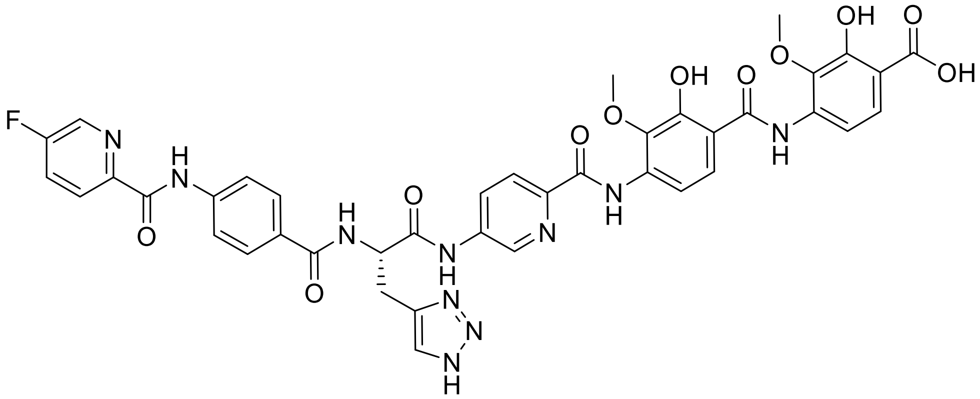

The synthesis of the product was conducted according to coupling protocol A. The albicidin derivative (**7**) was obtained as a colourless solid (10 mg, 21% over two steps).

^{1}H NMR (DMSO-d_6, 700 MHz): δ [ppm] = 14.67 (s, 1 H), 11.73 (s, 1 H), 11.64 (s, 1 H), 11.13 (s, 1 H), 10.89 (s, 1 H), 10.84 (s, 1 H), 10.50 (s, 1 H), 8.99 (d, *J* = 2.4 Hz, 1 H), 8.84 (d, *J* = 7.6 Hz, 1 H), 8.76 (d, *J* = 2.9 Hz, 1 H), 8.35 (dd, *J* = 8.6, 2.4 Hz, 1 H), 8.27 (dd, *J* = 8.7, 4.6 Hz, 1 H), 8.21 (d, *J* = 8.5 Hz, 1 H), 8.12 (d, *J* = 8.9 Hz, 1 H), 8.05 – 7.99 (m, 4 H), 7.92 (d, *J* = 8.8 Hz, 2 H), 7.89 (d, *J* = 8.9 Hz, 1 H), 7.68 (s, 1 H), 7.60 (d, *J* = 8.8 Hz, 1 H), 4.94 (dd, *J* = 7.5 Hz, 1 H), 3.92 (s, 3 H), 3.88 (s, 3 H), 3.28 (m, 2 H). **^{13}C NMR** from HSQC-ed (DMSO-d_6, 176 MHz): δ [ppm] = 140.0, 137.4, 137.3, 130.6, 128.8, 128.8, 127.7, 127.7, 127.0, 126.96, 126.1, 125.4, 125.4, 123.5, 123.4, 121.2, 120.0, 110.9, 110.8, 60.7, 63.7, 54.8. **HRMS** (ESI): m/z calculated for $C_{40}H_{34}FN_{10}O_{11}$ [*M*+H]$^+$ 849.2387; found 849.2375, t_R = 8.29 min.

Synthesis of albicidin derivative (**8**)

Methyl 4-(5-nitropicolinamido)benzoate (**8b**)

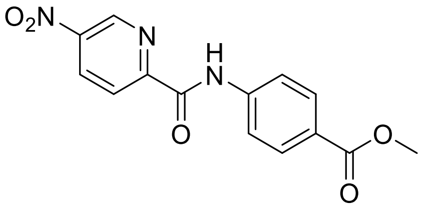

5-nitropicolinic acid (**8a**, 500 mg, 2.97 mmol, 1.00 eq.) was dissolved in $SOCl_2$ (5 mL). The reaction mixture was heated to reflux and stirred for 2 h. After that, all volatiles were removed by rotary evaporation. The resulting solid was dissolved in dry THF (5 mL) and added to a solution of of methyl 4-aminobenzoate (540 mg, 3.57 mmol, 1.20 eq.) and DIPEA (500 µL) in dry THF (5 mL). The reaction was stirred overnight and quenched with an aqueous solution of HCl (1 M, 10 mL). The aqueous layer was separated an extracted with EtOAc (3 × 10 mL). The combined organic fractions were washed with an aqueous solution of 1 M $NaHCO_3$ (10 mL) and brine (10 mL), dried over anhydrous Na_2SO_4 and concentrated under reduced pressure. Purification of the crude product by column chromatography on

silica gel (*n*-hexane/EtOAc, 9:1–2:1) afforded the methyl 4-(5-nitropicolinamido)benzoate (**8b**, 715 mg, 2.37 mmol, 80%) as a yellowish solid.

^{1}H NMR (500 MHz, DMSO-d_6) δ [ppm] = 11.19 (s, 1 H), 9.45 (d, *J* = 2.6, 1 H), 8.82 (dd, *J* = 8.6, 2.6 Hz, 1 H), 8.38 (d, *J* = 8.9 Hz, 2 H), 8.10 (d, *J* = 8.8 Hz, 2 H), 7.98 (d, *J* = 8.6 Hz, 2 H), 3.84 (s, 3 H). **HRMS** (ESI): *m*/*z* calculated for $C_{14}H_{12}N_3O_5$ [*M*+H]$^+$ 302.0771; found: 302.0772.

4-(5-nitropicolinamido)benzoate (**8c**)

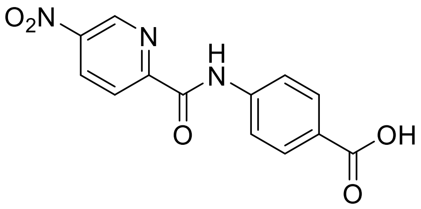

Methyl 4-(5-nitropicolinamido)benzoate (**8b**, 700 mg, 2.32 mmol, 1.00 eq.) was dissolved in THF (10 mL), cooled to 0°C and an aqueous solution of KOH (4 M, 2 mL) was added. The reaction was stirred for 30 minutes and quenched with an aqueous solution of HCl (6 M, 2 mL). The product precipitated as a white solid that was separated by centrifugation to yield target compound (**8c**, 580 mg, 2.02 mmol, 87%).

^{1}H NMR (500 MHz, DMSO-d_6) δ [ppm] = 12.79 (s, 1 H), 11.15 (s, 1 H), 9.46 (d, *J* = 2.2 Hz, 1 H), 8.84 (dd, *J* = 8.6, 2.6 Hz, 1 H), 8.41 (d, *J* = 8.5 Hz, 1 H), 8.07 (d, *J* = 8.7 Hz, 2 H), 7.97 (d, *J* = 8.6 Hz, 2 H). **HRMS** (ESI): *m*/*z* calculated for $C_{13}H_{10}N_3O_5$ [*M*+H]$^+$ 288.0615; found: 288.0616.

Albicidin derivative (**8**)

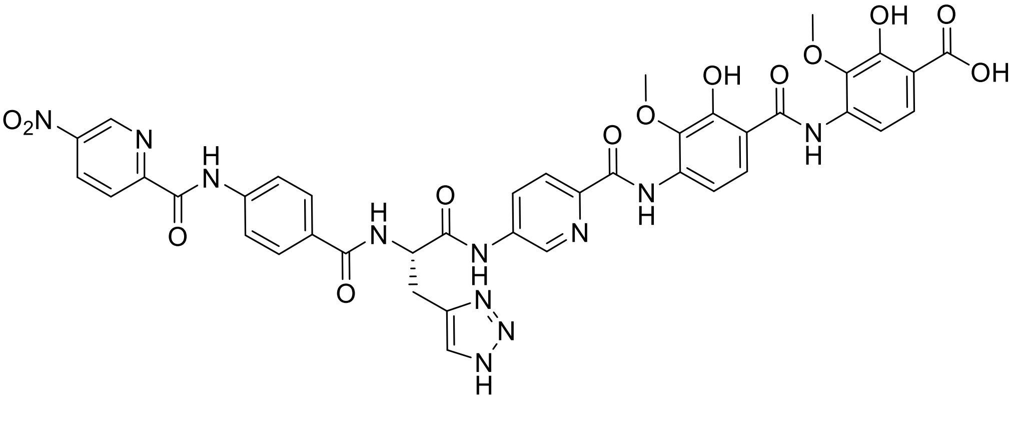

The synthesis of the product was conducted according to coupling protocol A. The albicidin derivative (**6**) was obtained as a colorless solid (12 mg, 25% over two steps).

^{1}H NMR (DMSO-d_6, 500MHz): δ [ppm] = 14.66 (s, 1 H), 11.74 (s, 1 H), 11.10 (s, 1 H), 11.04 (s, 1 H), 10.84 (s, 1 H), 10.49 (s, 1 H), 9.46 (d, *J* = 2.5 Hz, 1 H), 8.98 (d, *J* = 2.4 Hz, 1 H), 8.85 (dd, *J* = 8.6, 2.5 Hz, 2 H), 8.41 (d, *J* = 8.6 Hz, 1 H), 8.34 (dd, *J* = 8.8, 2.5 Hz, 1 H), 8.21 (d, *J* = 8.6 Hz, 1 H), 8.11 (d, *J* = 8.9 Hz, 1 H), 8.05 (d, *J* = 8.5 Hz, 2 H), 7.94 (m, 3 H), 7.88 (d, *J* = 8.9 Hz, 1 H), 7.67 (s, 1 H), 7.57 (d,

J = 8.8 Hz, 1 H), 4.95 (m, 1 H), 3.91 (s, 3 H), 3.88 (s, 3 H), 3.08-3.11 (m, 2 H) **^{13}C NMR** from HSQC-ed (DMSO-d_6, 176 MHz): δ [ppm] = 140.2, 134.3, 133.4, 128.8, 127.7, 127.0, 126.1, 123.9, 123.4, 120.3, 120.0, 110.8, 61.4, 60.0, 54.7, 28.2. **HRMS** (ESI): m/z calculated for $C_{40}H_{34}N_{11}O_{13}$ $[M+H]^+$ 876.2332; found 876.2315, t_R = 8.30 min.

Synthesis of albicidin derivative (**9**)

(*R*)-2-(4-Acetoxyphenoxy)propionic acid (**9b**)

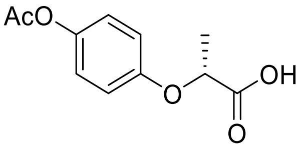

(*R*)-2-(4-Hydroxyphenoxy)propionic acid (**9a**, 3.00 g, 16.5 mmol, 1.00 Äq.) was dissolved in a mixture of Ac_2O (15.0 mL, 159 mmol, 9.64 eq.) and pyridine (15.0 mL, 186 mmol, 11.3 eq.) and stirred for 16 h at room temperature. The reaction mixture was cooled to 0 °C, water (50 mL) was added and the mixture was extracted with EtOAc (3 × 20 mL). The combined organic layers were washed with an aqueous solution of HCl (1 M, 3 × 20 mL), brine (20 mL) and dried over anhydrous Na_2SO_4. After filtration of the organic phase, the solvent was removed under reduced pressure by rotary evaporation. The crude product was purified by flash column chromatography (SiO_2, *n*-Hexan/EtOAc, 1:1). Acid (**9b**, 3.19 g, 14.2 mmol, 86%) was obtained as colourless solid.

^{1}H-NMR (400 MHz, DMSO-d_6): δ [ppm] = 13.02 (br. s, 1 H), 7.00 - 7.05 (m, 2 H), 6.85 - 6.90 (m, 2 H), 4.81 (q, J = 6.8 Hz, 1 H), 2.22 - 2.24 (m, 3 H), 1.50 (d, J = 6.8 Hz, 3 H). **^{13}C-NMR** (101 MHz, DMSO-d_6): δ [ppm] = 173.0, 169.4, 155.0, 144.1, 122.6, 115.3, 71.8, 20.7, 18.1. **HRMS** (ESI): *m/z* calculated for $C_{11}H_{12}O_5$ $[M-H]^-$ 223.0601, found: 223.0611.

Methyl (*R*)-4-(2-(4-acetoxyphenoxy)propanamido)benzoate (**9c**)

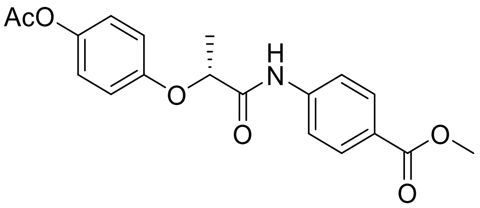

(*R*)-2-(4-Acetoxyphenoxy)propionic acid (**9b**, 100 mg, 446 µmol, 1.00 eq.) was dissolved in THF (5 mL) and EEDQ (165 mg, 669 µmol, 1.50 eq.) was added and the reaction mixture was stirred for 1 h at room temperature. A solution of methyl 4-aminobenzoate (101 mg, 669 µmol, 1.50 eq.) in THF (2 mL) was added and the reaction mixture was stirred for 16 h at room temperature. The solvent was removed by rotary evaporation, the residue was dissolved in EtOAc (20 mL) and the organic layer washed with an aqueous solution of HCl (1 M, 3 × 10 mL), brine (10 mL). The organic layer was dried over anhydrous Na_2SO_4, filtrated und the solvent was removed under reduced pressure by rotary evaporation. The crude

product was purified by flash column chromatography (SiO_2, *n*-Hexan/Ethylacetat, 8:2) and the product (**9c**, 120 mg, 337 µmol, 76%) was obtained as colorless solid.

^{1}H-NMR (400 MHz, DMSO-*d*$_6$): δ [ppm] = 10.49 (s, 1 H), 7.92 (d, *J* = 9.1 Hz, 2 H), 7.79 (d, *J* = 9.5 Hz, 2 H), 7.05 (d, *J* = 9.6 Hz, 2 H), 6.97 (d, *J* = 9.5 Hz, 2 H), 4.90 (q, *J* = 6.9 Hz, 1 H), 3.82 (s, 3 H), 2.22 (s, 3 H), 1.56 (d, *J* = 6.5 Hz, 3 H). **^{13}C-NMR** (101 MHz, DMSO-*d*$_6$): δ [ppm] = 170.5, 169.4, 165.7, 154.7, 144.4, 142.8, 130.2, 124.4, 122.7, 119.1, 115.7, 74.1, 51.9, 20.7, 18.5. **HRMS** (ESI): *m/z* calculated for $C_{19}H_{19}NO_6$ $[M+H]^+$ 358.1285, found: 358.1285.

(*R*)-4-(2-(4-phenoxy)propanamido)benzoic acid (**9d**)

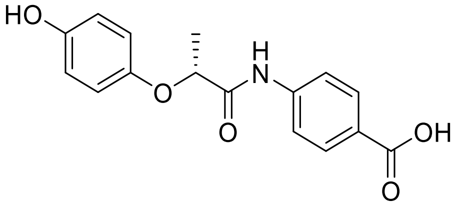

Methyl (*R*)-4-(2-(4-acetoxyphenoxy)propanamido)benzoate (**9c**, 350 mg, 979 µmol, 1.00 eq.) was dissolved in a mixture of MeOH and THF (1:1, 4 mL) and an aqueous solution of KOH (3 M, 4 mL) was added. The reaction mixture was stirred for 4 h at room temperature, after which an aqueous solution of HCl (3 M, 5 mL) was added. The formed precipitate was filtered and the product (**9d**, 230 mg, 763 µmol, 78%) was obtained as colorless solid.

^{1}H-NMR (400 MHz, DMSO-*d*$_6$): δ [ppm] = 12.75 (br. s., 1 H), 10.36 (s., 1 H), 9.02 (s., 1 H), 7.89 (d, *J* = 8.4 Hz, 2 H), 7.77 (q, *J* = 8.4 Hz, 2 H), 6.81 (q, *J* = 8.4 Hz, 2 H), 6.67 (d, *J* = 8.4 Hz, 2 H), 4.73 (q, *J* = 6.5 Hz, 1 H), 1.50 (d, *J* = 6.3 Hz, 3 H). **^{13}C-NMR** (101 MHz, DMSO-*d*$_6$): δ [ppm] = 171.0, 166.8, 151.9, 149.9, 142.5, 130.3, 125.5, 118.9, 116.6, 115.7, 74.9, 18.6. **HRMS** (ESI): *m/z* calculated for $C_{16}H_{15}NO_5$ $[M+H]^+$ 302.1023, found: 302.1014.

Perchlorophenyl (*R*)-4-(2-(4-phenoxy)propanamido)benzoate (**9e**)

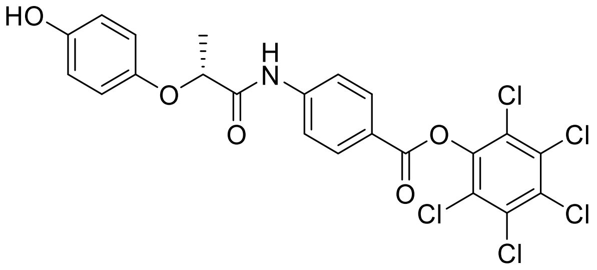

(*R*)-4-(2-(4-phenoxy)propanamido)benzoic acid (**9d**, 130 mg, 432 µmol, 1.00 eq.), pentachlorophenol (138 mg, 518 µmol, 1.20 eq.), EDC (124 mg, 647 µmol, 1.50 eq.) and DIPEA (139 µL, 1.08 mmol, 2.50 eq.) were dissolved in DMF (3 mL). To the reaction mixture was added EtOAc (20 mL) and the organic phase was washed with an aqueous solution of HCl (1 M, 3 × 20 mL) and a saturated aqueous solution of NaCl (20 mL), dried over $MgSO_4$, filtered and concentrated under reduced pressure. The crude product was purified by flash column chromatography (SiO_2, DCM/MeOH, 98:2) and the product (**9e**, 100 mg, 182 µmol, 42%) was obtained as colorless solid.

^{1}H-NMR (400 MHz, DMSO-d_6): δ [ppm] = 10.57 (s, 1 H), 9.03 (s, 1 H), 8.16 (d, 8.5 Hz, 2 H), 7.94 (d, J = 8.5 Hz, 2 H), 6.82 (d, J = 9.0 Hz, 2 H), 6.68 (d, 9.0 Hz, 2 H), 4.76 (d, J = 6.8 Hz, 1 H), 1.52 (d, J = 6.8 Hz, 3 H). **^{13}C-NMR** (101 MHz, DMSO-d_6): δ [ppm] = 171.4, 161.7, 151.9, 149.8, 144.7, 144.1, 131.6, 131.2, 130.7, 127.4, 120.7, 119.5, 116.7, 115.7, 75.0, 18.5. **HRMS** (ESI): *m/z* calculated for $C_{22}H_{14}Cl_5NO_5$ $[M+H]^+$ 549.9358, found 549.9355.

Albicidin derivative (**9**)

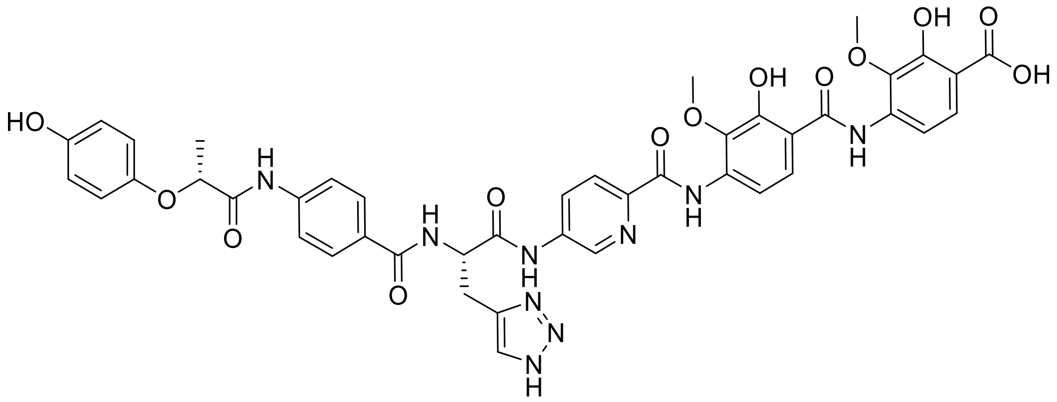

PCP-activated AB building block (**9e**, 74.4 mg, 135 µmol, 1.30 eq.) was dissolved in anhydrous DMF (2 mL) and a solution of tetrapeptide (**26**, 75.0 mg, 104 µmol, 1.00 eq.) and DIPEA (87 µL) in anhydrous DMF (1 mL) was added dropwise. After the reaction mixture was stirred at r.t for 16 h all volatiles were removed in vacuo and the residue was taken up in a mixture of THF (1 mL) and MeOH (1 mL), and an aqueous solution of KOH (3 M, 1 mL) was added dropwise. After 45 min of stirring, an aqueous solution of HCl (3 M, 1.1 mL) was added and the resulting suspension was evaporated under reduced pressure. The crude material was dissolved in DMSO, centrifuged, and the supernatant purified by HPLC (PLRP-S column, CH_3CN in H_2O). The title compound (**9**, 26 mg, 28% over two steps) was obtained as a yellow solid.

^{1}H-NMR (500 MHz, DMSO-d_6): δ [ppm] = 11.71 (br. s., 1 H), 11.58 (br. s, 1 H), 11.12 (s, 1 H), 10.83 (s, 1 H), 10.48 (s, 1 H), 10.26 (s, 1 H), 8.96 (d, J = 1.8 Hz, 1 H), 8.77 (d, J = 7.3 Hz, 1 H), 8.33 (dd, J = 8.6, 1.9 Hz, 1 H), 8.20 (d, J = 8.5 Hz, 1 H), 8.11 (d, J = 8.9 Hz, 1 H), 8.03 (d, J = 8.9 Hz, 1 H), 7.83 – 7.90 (m, 3 H), 7.75 (d, J = 8.7 Hz, 2 H), 7.69 (s, 1 H), 7.59 (d, J = 8.9 Hz, 1 H), 6.82 (d, J = 9.0 Hz, 2 H), 6.67 (d, J = 9.0 Hz, 2 H), 4.92 (dd, J = 7.5, 14.2 Hz, 1 H), 4.72 (q, J = 6.6 Hz, 1 H), 3.95 (s, 3 H), 3.86 (s, 3 H), 3.26 (dd, J = 5.5, 14.9 Hz, 1 H), 3.19 (dd, J = 8.7, 14.5 Hz, 1 H), 1.50 (d, J = 6.6 Hz, 3 H) **^{13}C NMR** from HSQC-ed (DMSO-d_6, 126 MHz): δ [ppm] = 172.6, 171.6, 171.3, 166.4, 164.0, 162.0, 154.9, 152.4, 150.5, 150.1, 143.9, 141.9, 140.0, 139.2, 138.3, 137.9, 136.7, 136.0, 129.1, 128.8, 127.7, 126.9, 126.0, 123.4, 119.3, 117.2, 116.2, 116.0, 111.0, 110.7, 109.4, 75.5, 61.3, 60.6, 54.7, 19.1. **HRMS** (ESI): m/z calculated for $C_{43}H_{39}N_9O_{13}$ $[M+H]^+$ 890.2734, found 890.2734, t_R = 7.78 min.

Synthesis of albicidin derivative (**10**)

4-(6-hydroxy-2-naphthamido)benzoic acid (**10b**).

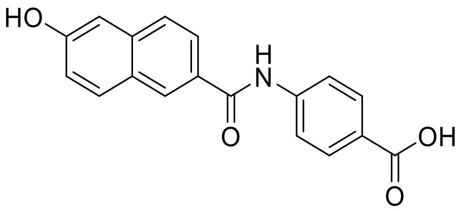

6-Hydroxy-2-naphthoic acid (**10a**, 1.00 g, 5.31 mmol, 1.25 eq.) was dissolved in $SOCl_2$ (5 mL) and the reaction mixture was heated to reflux and stirred for 2 h. The volatiles were evaporated under reduced pressure and the resulting solid was dissolved in dry THF (5 mL) and added to a solution of methyl 4-aminobenzoate (**25b**, 585 mg, 3.87 mmol, 1.00 eq.) and DIPEA (500 µL) in dry THF (5 mL). The reaction was stirred overnight and quenched with an aqueous solution of HCl (1 M, 10 mL). The aqueous layer was extracted with EtOAc (3 × 10 mL) and the combined organic layers were washed with an aqueous solution of 1 M $NaHCO_3$ (10 mL) and brine (10 mL), dried over anhydrous Na_2SO_4 and concentrated under reduced pressure. The crude product was dissolved in THF (10 mL), cooled to 0°C and an aqueous solution of KOH (4 M, 2 mL) was added. The reaction was stirred for 30 minutes and quenched with HCl (6 M, 2 mL). The product precipitated as a yellowish solid that was separated by centrifugation to yield of target compound (**10b**, 650 mg, 2.12 mmol, 44% yield).

^{1}H NMR (400 MHz, DMSO-*d*$_6$) δ [ppm] = 10.63 (s, 1 H), 8.52 (d, *J* = 1.9 Hz, 1 H), 8.00 – 7.93 (m, 7 H), 7.80 (d, *J* = 8.7 Hz, 1 H), 7.22 (s, 1 H). **^{13}C NMR** (101 MHz, DMSO) δ [ppm] = 167.0, 166.0, 165.7, 157.3, 143.6, 136.4, 130.8, 130.2, 129.4, 129.3, 128.8, 128.5, 128.3, 127.3, 126.5, 126.1, 125.2, 124.7, 122.4, 119.6, 119.5, 119.4, 108.7. **HRMS** (ESI): *m*/*z* calculated for $C_{18}H_{14}NO_4$ [*M*+H]$^+$ 308.0917; found 308.0919.

Albicidin derivative (**10**)

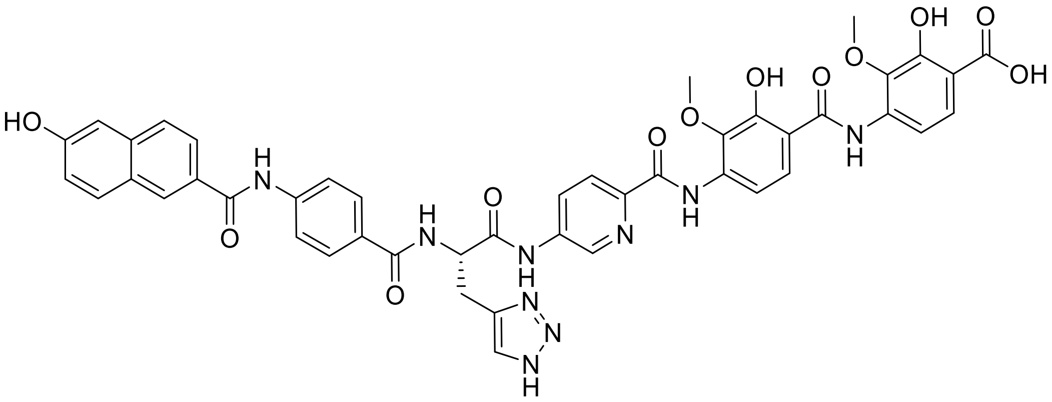

The synthesis of the product was conducted according to coupling protocol A. The albicidin derivative (**10**) was obtained as a colorless solid (8 mg, 16% over two steps).

^{1}H NMR (DMSO-*d*$_6$, 700 MHz): δ [ppm] = 11.72 (s, 1 H), 11.13 (s, 1 H), 10.85 (s, 1 H), 10.52 (s, 1 H), 10.50 (s, 1 H), 10.11 (s, 1 H), 8.98 (d, *J* = 2.3 Hz, 1 H), 8.81 (d, 1 H), 8.50 (s, 1 H), 8.35 (dd, *J* = 8.5, 2.4 Hz, 1 H), 8.22 (d, *J* = 8.6 Hz, 1 H), 8.12 (d, *J* = 8.9 Hz, 1 H), 8.03 (d, *J* = 8.8 Hz, 1 H), 7.96 – 7.90 (m, 6 H), 7.89 (d, *J* = 8.8 Hz, 1 H), 7.82 (d, *J* = 8.7 Hz, 1 H), 7.77 (s, 1 H), 7.60 (d, *J* = 8.8 Hz, 1 H), 7.23 – 7.18 (m, 2 H), 4.95 (dd, *J* = 7.6 Hz, 1 H), 3.90 (s, 3 H), 3.88 (s, 3 H) **^{13}C NMR** from HSQC-ed

(DMSO-d_6, 176 MHz): δ [ppm] = 140.0, 131.3, 128.8, 128.7, 127.6, 127.0, 126.6, 126.2, 125.1, 123.5, 120.1, 119.7, 110.7, 109.2, 63.8, 61.3, 60.7. **HRMS** (ESI): m/z calculated for $C_{45}H_{38}N_9O_{12}$ [M+H]$^+$ 896.2634; found 896.2643, t_R = 8.15 min.

Synthesis of albicidin derivative (**11**)

methyl 4-(quinoline-2-carboxamido)benzoate (**11b**).

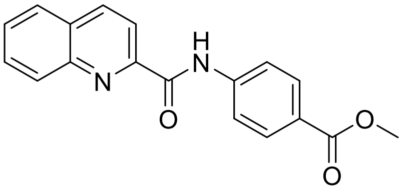

To a solution of quinoline-2-carboxylic acid (**11a**, 500 mg, 2.89 mmol, 1.00 eq.) in DMF (5 mL) was added HATU (1.32 g, 3.47 mmol, 1.20 eq.), HOBt (195 mg, 1.44 mmol, 0.50 eq.) and DIPEA (1.51 mL, 1.12 g, 8.66 mmol, 3.00 eq.). The reaction mixture was stirred for 1 h at room temperature after which methyl 4-aminobenzoate (524 mg, 3.46 mmol, 1.20 eq.) was added and the reaction mixture was stirred overnight at room temperature. The reaction mixture was diluted with EtOAc (50 mL) washed with a saturated aqueous solution of $NaHCO_3$ (3 × 50 mL) and brine (50 mL), dried over $MgSO_4$ and concentrated under reduced pressure by rotary evaporation. The crude product was purified flash column chromatography (SiO_2, hexane/ethyl acetate 8:2) to yield the title compound (**11b**, 554 mg, 1.80 mmol, 63%) as a colourless solid.

^{1}H NMR (400 MHz, DMSO-d_6): δ [ppm] = 11.05 (s, 1 H), 8.65 (d, J = 8.3 Hz, 1 H), 8.28 (d, J = 8.6 Hz, 1 H), 8.25 (d, J = 8.4 Hz, 1 H), 8.14 (d, J = 8.5 Hz, 2 H), 8.01 (d, J = 8.8 Hz, 2 H), 7.94 (ddd, J = 1.4, 7.0, 8.4 Hz, 1 H), 7.77 (ddd, J = 1.5, 7.0 Hz 1 H), 3.85 (s, 3 H). **HRMS** (ESI): m/z calculated for $C_{16}H_{13}N_2O_4$ [M+H]$^+$ 307.1078; found 307.1073.

4-(quinoline-2-carboxamido)benzoic acid (**11c**)

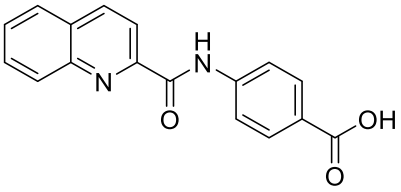

The methyl ester (**11b**, 100 mg, 0.325 mmol, 1.00 eq.) was dissolved in a mixture of THF/MeOH (1:1, 10 mL) and an aqueous solution of KOH (3 M, 5 mL) solution was added. The reaction mixture was stirred for 3 h, after which the organic solvent was removed under reduced pressure by rotary evaporation. The residue was treated with aqueous HCl (4 M, 5 mL) and the precipitated product (**11c**, 95.4 mg, 0.325 mmol, quant.) was filtrated and obtained as brownish solid.

¹H NMR (400 MHz, DMSO-d_6): δ [ppm] = 11.01 (s, 1 H), 8.65 (d, *J* = 8.3 Hz, 1 H), 8.30 – 8.24 (m, 2 H), 8.14 (d, *J* = 8.5 Hz, 2 H), 8.11 (d, *J* = 8.8 Hz, 2 H), 7.99 (ddd, *J* = 8.6 Hz, 1 H), 7.94 (ddd, *J* = 1.5, 7.0 Hz 1 H). **HRMS** (ESI): *m/z* calculated for $C_{16}H_{13}N_2O_4$ [*M*+H]⁺ 293.0921; found 293.0914.

Albicidin derivative (**11**)

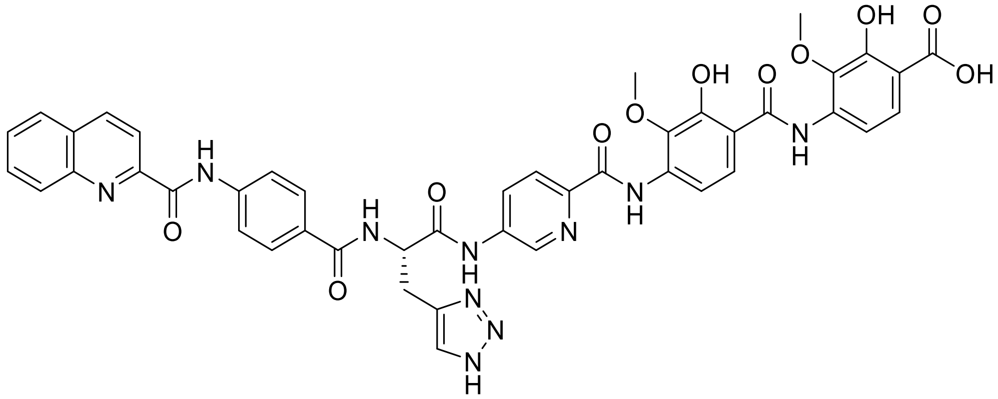

The synthesis of the product was conducted according to coupling protocol A. The albicidin derivative **11** (6 mg, 7% over two steps) was obtained as a colourless solid.

¹H NMR (DMSO-d_6, 700 MHz): δ [ppm] = 11.72 (s, 1 H), 11.59 (s, 1 H), 11.14 (s, 1 H), 10.98 (s, 1 H), 10.86 (s, 2 H), 10.50 (s, 1 H), 9.00 (d, *J* = 1.0 Hz, 2 H), 8.87 (d, *J* = 7.3 Hz, 1 H), 8.67 (d, *J* = 8.6 Hz, 1 H), 8.35 (dd, *J* = 8.6, 2.4 Hz, 1 H), 8.29 – 8.26 (m, 2 H), 8.22 (d, *J* = 8.6 Hz, 1 H), 8.15 (d, *J* = 7.9 Hz, 1 H), 8.12 (d, *J* = 8.9 Hz, 1 H), 8.10 (d, *J* = 8.6 Hz, 2 H), 8.04 (d, *J* = 8.9 Hz, 1 H), 7.97 (d, *J* = 8.6 Hz, 2 H), 7.94 (d, *J* = 7.6 Hz, 1 H), 7.89 (d, *J* = 8.9 Hz, 1 H), 7.78 (dd, *J* = 7.4, 14.7 Hz, 1 H), 7.73 (s, 1 H), 7.60 (d, *J* = 8.9 Hz, 1 H), 4.96 (dd, *J* = 7.5 Hz, 1 H), 3.93 (s, 3 H), 3.88 (s, 3 H), 3.35 (dd, *J* = 14.7, 5.7 Hz, 1 H), 3.28 (dd, *J* = 14.8, 9.2 Hz, 1 H). **¹³C NMR** from HSQC-ed (DMSO-d_6, 176 MHz): δ [ppm] =140.0, 138.8, 131.2, 129.8, 129.0, 128.9, 128.6, 127.8, 127.0, 126.1, 123.5, 120.0, 119.1, 110.9, 110.7, 61.3, 60.7, 54.9, 27.5. **HRMS** (ESI): m/z calculated for $C_{44}H_{37}N_{10}O_{11}$ [M+H]⁺ 881.2638; found 881.2642, t_R = 9.49 min.

Synthesis of albicidin derivative (**12**)

***tert*-butyl 4-(isoquinoline-3-carboxamido)benzoate** (**12b**).

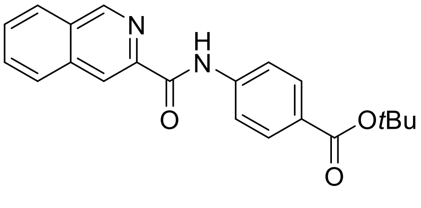

To a solution of isoquinoline-3-carboxylic acid (**12a**, 500 mg, 2.89 mmol, 1.00 eq.) in DMF (5 mL) was added HATU (1.43 g, 3.75 mmol, 1.30 eq.), and DIPEA (1.51 mL, 1.12 g, 8.66 mmol, 3.00 eq.). The reaction mixture was stirred for 1 h at room temperature after which *tert*-butyl 4-aminobenzoate (725 mg,

3.75 mmol, 1.30 eq.) was added and the reaction mixture was stirred overnight at room temperature. The reaction mixture was diluted with EtOAc (10 mL) and the precipitated product (**12b**, 534 mg, 1.53 mmol, 53%) was filtrated and obtained as a colourless solid.

^{1}H NMR (400 MHz, $CDCl_3$) δ [ppm] = 10.34 (s, 1 H), 9.15 (s, 1 H), 8.65 (s, 1 H), 8.01 (d, *J* = 8.1 Hz, 2 H), 7.98 – 7.95 (m, 3 H), 7.83 (d, *J* = 8.8 Hz, 2 H), 7.74 (ddd, *J* = 1.4, 7.2, 8.2 Hz, 1 H), 7.68 (ddd, *J* = 1.4, 8.1, 9.1 Hz, 1 H), 1.54 (s, 9 H). **^{13}C NMR** (101 MHz, $CDCl_3$): δ [ppm] = 165.4, 162.7, 151.0, 143.1, 141.7, 136.1, 131.4, 130.7, 129.9, 129.3, 128.3, 127.8, 127.5, 121.1, 118.8, 80.8, 28.3. **HRMS** (ESI): *m*/*z* calculated for $C_{16}H_{13}N_2O_4$ [*M*+H]$^+$ 349.1547; found 349.1538.

4-(isoquinoline-3-carboxamido)benzoic acid (**12c**)

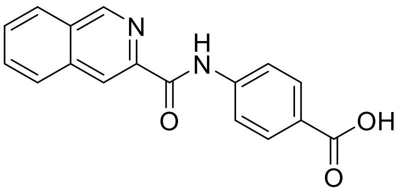

The *tert*-butyl ester (**12b**, 100 mg, 0.325 mmol, 1.00 eq.) was dissolved in a solution of HCl in dioxane (4 M, 10 mL) the reaction mixture was stirred for 4 h, after which the volatiles were removed under reduced pressure by rotary evaporation and the product (**12c**, 83.9 mg, 0.287 mmol, quant.) was obtained as white solid.

HRMS (ESI): *m*/*z* calculated for $C_{16}H_{13}N_2O_4$ [*M*+H]$^+$ 293.0920; found 293.0914.

Albicidin derivative (**12**)

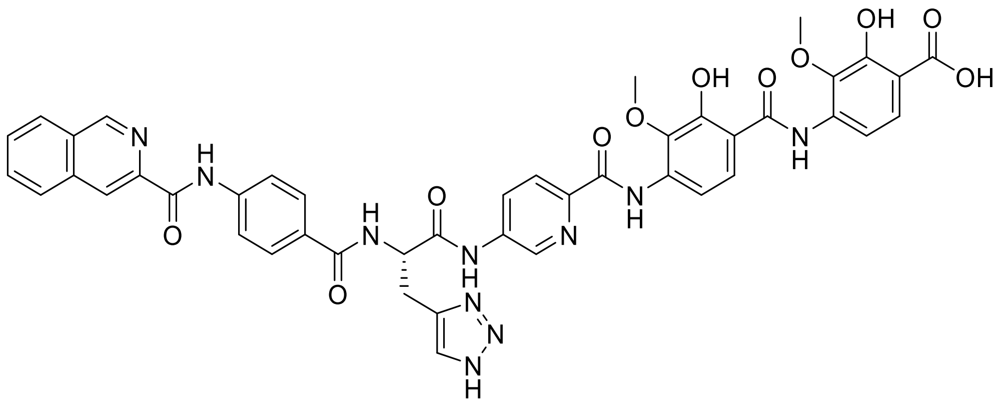

The synthesis of the product was conducted according to coupling protocol A. The albicidin derivative **12** (6 mg, 7% over two steps) was obtained as a colourless solid. **^{1}H NMR** (DMSO-d_6, 700 MHz): δ [ppm] = 11.73 (s, 1 H), 11.60 (s, 1 H), 11.14 (s, 1 H), 11.00 (s, 1 H), 10.86 (d, *J* = 7.2 Hz, 2 H), 10.50 (s, 1 H), 9.51 (s, 1 H), 8.99 (dd, *J* = 2.3 Hz, 1 H), 8.85 (d, *J* = 7.4 Hz, 1 H), 8.75 (s, 1 H), 8.35 (dd, *J* = 8.6, 2.4 Hz, 1 H), 8.33 (d, *J* = 8.7 Hz, 1 H), 8.28 (d, *J* = 7.9 Hz, 1 H), 8.22 (d, *J* = 8.6 Hz, 1 H), 8.12 – 8.10 (m, 3 H), 8.04 (d, *J* = 8.9 Hz, 1 H), 7.95 – 7.93 (m, 3 H), 7.89 – 7.87 (m, 2 H), 7.73 (s, 1 H), 7.60

(d, J = 8.8 Hz 1 H), 4.95 (dd, J = 7.5 Hz, 1 H), 3.92 (s, 3 H), 3.88 (s, 3 H), 3.35 (dd, J = 14.7, 5.7 Hz, 1 H), 3.28 (dd, J = 14.8, 9.2 Hz, 1 H). **^{13}C NMR** from HSQC-ed (DMSO-d_6, 176 MHz): δ [ppm] = 152.0, 139.9, 132.0, 130.1, 128.8, 128.6, 128.3, 126.9, 126.1, 119.9, 110.9, 110.7, 61.3, 60.7, 54.8, 27.7. **HRMS** (ESI): m/z calculated for $C_{44}H_{37}N_{10}O_{11}$ [M+H]$^+$ 881.2638; found 881.2636, t_R = 8.74 min.

Synthesis of Albicidin derivative (**13**)

4-(quinoline-3-carboxamido)benzoic acid (**13b**).

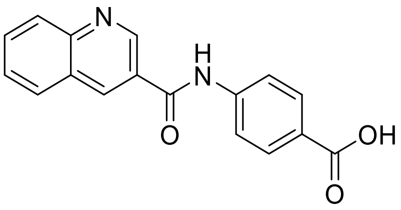

Quinoline-3-carboxylic acid (**13a**, 1.00 g, 5.77 mmol, 1.25 eq.) was dissolved in $SOCl_2$ (5 mL). The reaction mixture was heated to reflux and stirred for 2 h. The volatiles were evaporated and the resulting solid was dissolved in dry THF (5 mL) and added to a solution of methyl 4-aminobenzoate (670 mg, 4.70 mmol, 1.00 eq.) and DIPEA (500 µL) in dry THF (5 mL). The reaction was stirred overnight and quenched with an aqueous solution of HCl (1 M, 10 mL). The aqueous layer was extracted with EtOAc (3 × 10 mL) and the combined organic layers were washed with an aqueous solution of 1 M $NaHCO_3$ (10 mL) and brine (10 mL), dried over anhydrous Na_2SO_4, filtrated and concentrated under reduced pressure. The crude product was dissolved in THF (10 mL), cooled to 0°C and an aqueous solution of KOH (4 M, 2 mL) were added. The reaction was stirred for 30 minutes and quenched with an aqueous solution of HCl (6 M, 2 mL). The product precipitated as a yellowish solid that was separated by centrifugation to yield the target compound (**13b**, 800 mg 2.74 mmol, 47% yield).

^{1}H NMR (400 MHz, DMSO-d_6): δ [ppm] = 12.81 (s, 1 H), 10.91 (s, 1 H), 9.37 (d, J = 2.2 Hz, 1 H), 9.00 (d, J = 2.4 Hz, 1 H), 8.18 (d, J = 8.4, 2 H), 8.13 (d, J = 8.2 Hz, 1 H), 8.03 – 7.93 (m, 4 H), 7.92 (ddd, J = 8.5, 6.9, 1.5 Hz, 1 H), 7.75 (ddd, J = 8.1, 6.8, 1.2 Hz, 1 H). **^{13}C NMR** (101 MHz, DMSO-d_6): δ [ppm] = 166.9, 164.5, 149.03, 148.5, 143.0, 136.3, 131.6, 130.3, 129.3, 128.7, 127.6, 127.4, 126.4, 125.8, 119.5. **HRMS** (ESI): m/z calculated for $C_{17}H_{13}N_2O_4$ [M+H]$^+$ 293.0921; found 293.0922.

Albicidin derivative (**13**)

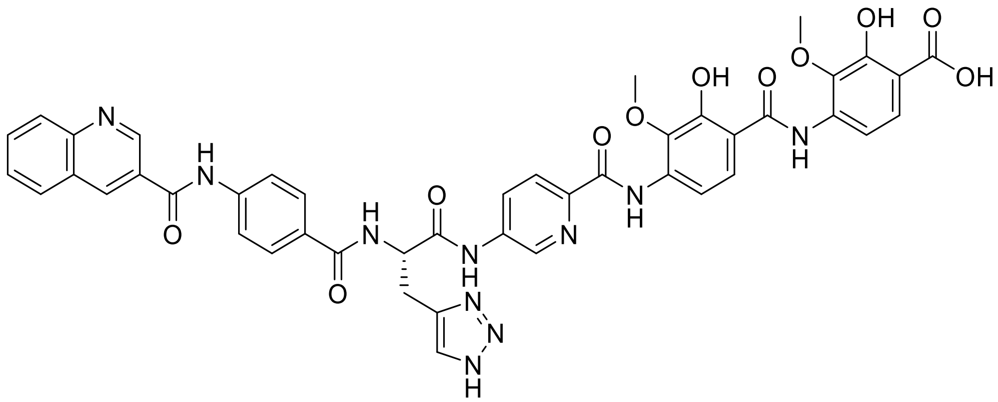

The synthesis of the product was conducted according to coupling protocol A. The albicidin derivative (**13**, 6 mg, 7% over two steps) was obtained as a colourless solid. **^{1}H NMR** (DMSO-d_6, 700 MHz): δ [ppm] = 11.73 (s, 1 H), 11.60 (s, 1 H), 11.14 (s, 1 H), 10.86 (s, 1 H), 10.85 (s, 1 H), 10.50 (s, 1 H), 9.39 (d, *J* = 2.2 Hz, 1 H), 9.01 (d, *J* = 2.3 Hz, 1 H), 8.98 (d, *J* = 2.3 Hz, 1 H), 8.85 (d, *J* = 7.4 Hz, 1 H), 8.35 (dd, J = 8.6, 2.4 Hz, 1 H), 8.22 (d, *J* = 8.6 Hz, 1 H), 8.18 (dd, *J* = 8.1, 1.4 Hz, 1 H), 8.14 (d, *J* = 8.4 Hz, 1 H), 8.12 (d, *J* = 8.9 Hz, 1 H), 8.04 (d, *J* = 8.9 Hz, 1 H), 7.98 – 7.94 (m, 4 H), 7.92 (ddd, *J* = 8.1, 6.8, 1.2 Hz, 1 H), 7.89 (d, *J* = 8.9 Hz, 1 H), 7.75 (ddd, *J* = 8.1, 6.8, 1.2 Hz, 1 H), 7.72 (s, 1 H), 7.66 – 7.59 (m, 2 H), 7.75 (ddd, *J* = 8.1, 7.5, 3.1 Hz, 1 H), 4.96 (dd, *J* = 7.5, 14.5 Hz, 1 H), 3.93 (s, 3 H), 3.88 (s, 3 H), 3.35 (dd, *J* = 14.7, 5.7 Hz, 1 H), 3.28 (dd, *J* = 14.8, 9.2 Hz, 1 H). **^{13}C NMR** from HSQC-ed (DMSO-d_6, 176 MHz): δ [ppm] = 149.5, 140.0, 136.7, 132.6, 132.0, 131.9, 129.6, 129.3, 129.2, 128.9, 128.1, 127.74, 127.0, 126.1, 123.5, 119.9, 110.9, 110.8, 61.3, 60.7, 54.8, 27.6. **HRMS** (ESI): m/z calculated for $C_{44}H_{37}N_{10}O_{11}$ $[M+H]^+$ 881.2638; found 881.2642, t_R = 8.07 min.

Synthesis of albicidin derivative (**14**)

methyl 4-(quinoline-6-carboxamido)benzoate (**14b**).

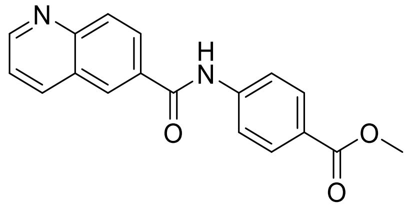

To a solution of quinoline-6-carboxylic acid (**14a**, 200 mg, 1.15 mmol, 1.00 eq.) in DMF (3 mL) was added HATU (571 mg, 1.50 mmol, 1.30 eq.), and DIPEA (605 mL, 448 mg, 3.46 mmol, 3.00 eq.). The reaction mixture was stirred for 1 h at room temperature after which methyl 4-aminobenzoate (227 mg, 1.50 mmol, 1.30 eq.) was added and the reaction mixture was stirred overnight at room temperature. The reaction mixture was diluted with EtOAc (30 mL) washed with a saturated aqueous solution of $NaHCO_3$ (3 × 30 mL) and brine (30 mL), dried over $MgSO_4$ and concentrated under reduced pressure by rotary evaporation. The crude product was purified flash column chromatography (SiO_2, hexane/ethyl acetate 8:2) to yield the title compound (**14b**, 208 mg, 0.678 mmol, 59%) as an off-white solid.

^{1}H NMR (400 MHz, DMSO-*d*$_6$): δ [ppm] = 10.84 (s, 1 H), 9.06 (dd, *J* = 1.8, 4.2 Hz, 1 H), 8.69 (d, *J* = 2.0 Hz, 1 H), 8.61 (dd, *J* = 1.3, 8.4 Hz, 1 H), 8.29 (dd, *J* = 2.3 Hz, 1 H), 8.00 (br. s, 4 H), 7.72 – 7.68 (m, 1 H), 3.86 (s, 3 H). **^{13}C NMR** (101 MHz, DMSO-*d*$_6$): δ [ppm] = 165.8, 165.5, 152.1, 143.5, 137.9, 132.6, 130.2, 128.8, 128.7, 128.4, 127.1, 124.5, 122.4, 119.6. **HRMS** (ESI): *m*/*z* calculated for $C_{16}H_{13}N_2O_4$ [*M*+H]$^+$ 307.1078; found 307.1076.

4-(quinoline-6-carboxamido)benzoic acid (**14c**)

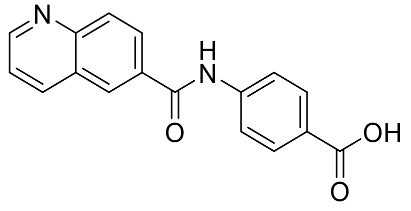

The methyl ester (**14a**, 100 mg, 0.325 mmol, 1.00 eq.) was dissolved in a mixture of THF/MeOH (1:1, 10 mL) and an aqueous solution of KOH (6 M, 5 mL) solution was added. The reaction mixture was stirred for 3 h, after which the organic solvent was removed under reduced pressure by rotary evaporation. The residue was treated with aqueous HCl (6 M, 5 mL) and the precipitated product (**14b**, 95.4 mg, 0.325 mmol, quant.) was filtrated and obtained as brownish solid.

^{1}H NMR (400 MHz, DMSO-*d*$_6$) δ [ppm] = 10.74 (s, 1 H), 9.30 (dd, *J* = 1.8, 4.3 Hz, 1 H), 9.07 (d, *J* = 1.9 Hz, 1 H), 8.95 (d, *J* = 1.4, 8.3 Hz, 1 H), 8.53 (dd, *J* = 1.9, 8.6 Hz, 1 H), 8.42 (d, *J* = 9.0 Hz, 1 H), 8.04 – 7.96 (m, 5 H). **^{13}C NMR** (101 MHz, DMSO-*d*$_6$): δ [ppm] = 167.4, 165.3, 149.2, 143.5, 134.6, 131.7, 130.8, 129.7, 128.1, 126.3, 124.6, 123.3, 120.1. **HRMS** (ESI): *m*/*z* calculated for $C_{17}H_{13}N_2O_4$ [*M*+H]$^+$ 293.0921; found 293.0918.

Albicidin derivative (**14**)

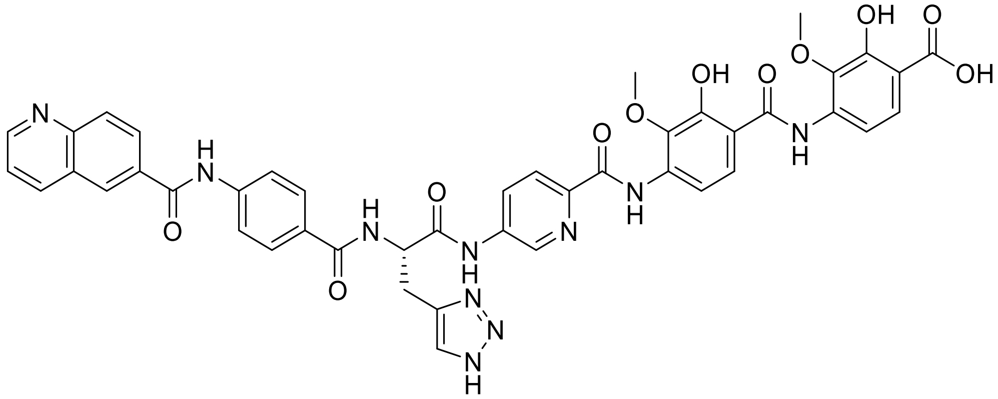

The synthesis of the product was conducted according to coupling protocol A. The albicidin derivative **14** (6 mg, 7% over two steps) was obtained as a colourless solid. **^{1}H NMR** (DMSO-d_6, 700 MHz): δ [ppm] = 11.72 (s, 1 H), 11.60 (s, 1 H), 11.14 (s, 1 H), 10.87 (d, *J* = 7.2 Hz, 1 H), 10.79 (s, 1 H), 10.50 (s, 1 H), 9.10 (d, *J* = 2.9 Hz, 1 H), 8.99 (d, *J* = 1.8 Hz, 1 H), 8.85 (d, *J* = 7.4 Hz, 1 H), 8.73 (s, 1 H), 8.68 (d, *J* = 7.9 Hz, 1 H), 8.36 – 8.33 (m, 2 H), 8.22 – 8.20 (m, 2 H), 8.12 (d, *J* = 8.5 Hz, 1 H), 8.04 (d, *J* = 8.9 Hz, 1 H), 7.95 (s, 4 H), 7.89 (d, *J* = 8.9 Hz, 1 H), 7.76 – 7.73 (m, 2 H), 7.60 (d, *J* = 9.0 Hz, 1 H), 4.96 (dd, *J* = 7.5 Hz, 1 H), 3.93 (s, 3 H), 3.89 (s, 3 H), 3.35 (dd, *J* = 14.7, 5.7 Hz, 1 H), 3.28 (dd, *J* = 14.8, 9.2 Hz, 1 H). **^{13}C NMR** from HSQC-ed (DMSO-d_6, 176 MHz): δ [ppm] = 152.1, 140.0, 129.3, 129.1, 128.9, 128.3, 127.0, 126.10, 123.5, 119.9, 110.9, 110.7, 61.3, 60.7, 54.8, 27.4 ppm. **HRMS** (ESI): m/z calculated for $C_{44}H_{37}N_{10}O_{11}$ $[M+H]^+$ 881.2638; found 881.2637, t_R = 7.67 min.

Synthesis of albicidin derivative (**15**)

methyl 4-(quinoxaline-6-carboxamido)benzoate (**15b**)

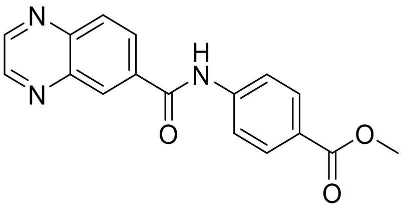

To a solution of quinoxaline-6-carboxylic acid (**15a**, 500 mg, 2.87 mmol, 1.00 eq.) in DMF (5 mL) was added HATU (1.64 g, 4.31 mmol, 1.50 eq.), HOBt (194 mg, 1.44 mmol, 0.50 eq.) and DIPEA (1.48 mL, 1.11 g, 8.61 mmol, 3.00 eq.). The reaction mixture was stirred for 1 h at room temperature after which methyl 4-aminobenzoate (524 mg, 3.46 mmol, 1.20 eq.) was added and the reaction mixture was stirred overnight at room temperature. The reaction mixture was diluted with EtOAc (50 mL) washed with a saturated aqueous solution of $NaHCO_3$ (3 × 50 mL) and brine (50 mL), dried over $MgSO_4$ and concentrated under reduced pressure by rotary evaporation. The crude product was purified flash column chromatography (SiO_2, hexane/ethyl acetate 1:1) to yield the title compound (**15b**, 778 mg, 2.53 mmol, 88%) as a colourless solid.

^{1}H NMR (400 MHz, DMSO-d_6): δ [ppm] = 10.95 (s, 1 H), 9.06 – 9.04 (m, 2 H), 9.31 (s, 1 H), 8.75 (d, *J* = 1.8 Hz, 1 H), 8.33 (dd, *J* = 1.9 8.7 Hz, 1 H), 8.23 (d, *J* = 8.6 Hz, 1 H), 7.98 (s, 4 H), 3.84 (s, 3 H). **HRMS** (ESI): *m*/*z* calculated for $C_{16}H_{13}N_2O_4$ [*M*+H]$^+$ 308.1029; found 308.1033.

4-(quinoxaline-6-carboxamido)benzoic acid (**15c**)

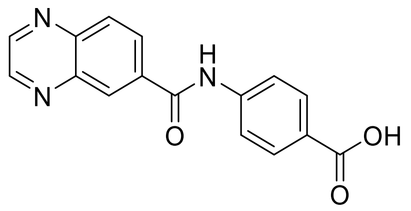

The methyl ester (**15b**, 374 mg, 1.22 mmol, 1.00 eq.) was dissolved in a mixture of THF/MeOH (1:1, 10 mL) and an aqueous solution of KOH (5 M, 5 mL) solution was added. The reaction mixture was stirred for 3 h, after which the organic solvent was removed under reduced pressure by rotary evaporation. The residue was treated with aqueous HCl (6 M, 5 mL) and the precipitated product (**15c**, 358 mg, 1.22 mmol, quant.) was filtrated and obtained as off-white solid.

^{1}H NMR (400 MHz, DMSO-d_6): δ [ppm] = 10.98 (s, 1 H), 9.09 (d, *J* = 1.8 Hz, 1 H), 9.07 (d, *J* = 1.8 Hz, 1 H), 9.06 (s, 1 H), 8.80 (d, *J* = 1.8 Hz, 1 H), 8.38 (d, J = 2.0, 8.8 Hz, 1 H), 8.25 (d, *J* = 8.8 Hz, 1 H), 7.99 (m, 4 H). **HRMS** (ESI): *m*/*z* calculated for $C_{16}H_{13}N_2O_4$ [*M*+H]$^+$ 294.0873; found 294.0876.

Albicidin derivative (**15**)

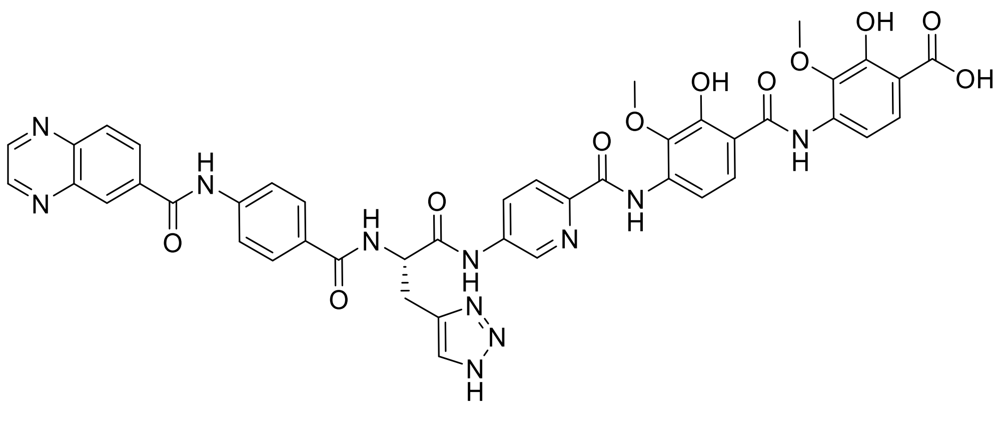

The synthesis of the product was conducted according to coupling protocol A. The albicidin derivative **15** (6 mg, 7% over two steps) was obtained as a colourless solid.

^{1}H NMR (DMSO-d_6, 700 MHz): δ [ppm] = 11.73 (s, 1 H), 11.60 (s, 1 H), 11.14 (s, 1 H), 10.86 (d, *J* = 7.2 Hz, 2 H), 10.50 (s, 1 H), 9.10 (d, *J* = 1.7 Hz, 1 H), 9.08 (d, *J* = 1.7 Hz, 1 H), 8.99 (d, 2.2 Hz, 1 H), 8.85 (d, *J* = 7.4 Hz, 1 H), 8.80 (d, 1.8 Hz, 1 H), 8.38 (dd, *J* = 8.6, 1.9 Hz, 1 H), 8.35 (dd, *J* = 8.6, 2.3 Hz, 1 H), 8.26 (d, *J* = 8.7 Hz, 1 H), 8.22 (d, *J* = 8.6 Hz, 1 H), 8.12 (d, *J* = 8.9 Hz, 1 H), 8.04 (d, *J* = 8.9 Hz, 1 H), 7.98 – 7.94 (m, 4 H), 7.73 (b, 1 H), 7.60 (d, 8.8 Hz, 1 H), 4.96 (dd, *J* = 7.5 Hz, 1 H), 3.93 (s, 3 H), 3.88 (s, 3 H), 3.35 (dd, *J* = 14.7, 5.7 Hz, 1 H), 3.28 (dd, *J* = 14.8, 9.2 Hz, 1 H). **^{13}C NMR** from HSQC-ed

(DMSO-d6, 700 MHz): δ [ppm] 147.7, 140.0, 129.9, 129.4, 129.4, 128.9, 127.8, 127.0, 126.1, 123.5, 120.0.87, 110.9, 110.7, 61.2, 60.7, 54.8, 27.5. **HRMS** (ESI): m/z calculated for $C_{43}H_{36}N_{11}O_{11}$ [*M*+H]+ 882.2590; found 882.2588, t_R = 7.71 min.

Synthesis of albicidin derivative (**16**)

Albicidin derivative (**16**)

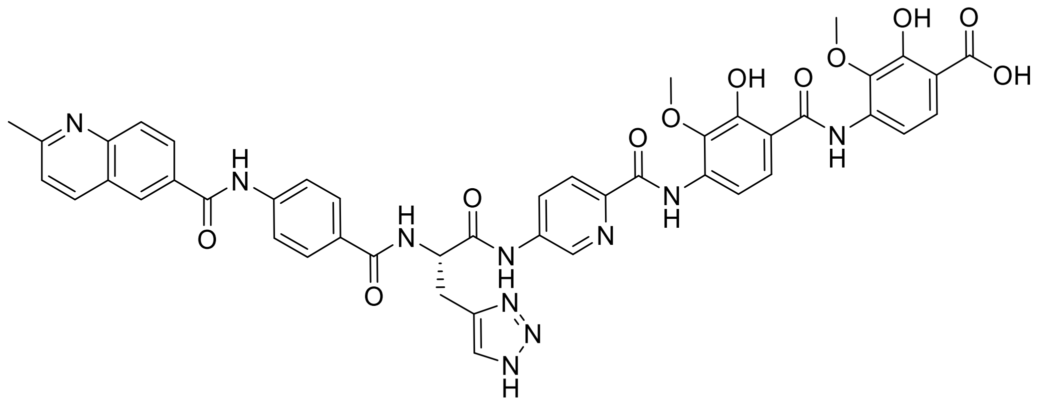

The synthesis of albicidin derivative **16** was reported in ref.[9]

Synthesis of albicidin derivative (**17**)

Methyl 4-(4-quinolone-3-carboxamido)benzoate (**17b**)

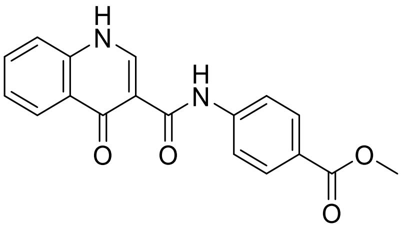

To a solution of 4-quinolone-3-carboxylic acid (**17a**, 2.00 g, 10.6 mmol, 1.00 eq.) in DMF (10 mL) was added HATU (6.03 g, 15.9 mmol, 1.50 eq.), and DIPEA (5.94 mL, 4.10 g, 31.7 mmol, 3.00 eq.). The reaction mixture was stirred for 1 h at room temperature after which methyl 4-aminobenzoate (2.40 g, 15.9 mmol, 1.50 eq.) was added and the reaction mixture was stirred overnight at room temperature. The reaction mixture was diluted with EtOAc (50 mL) washed with an aqueous solution of HCl (1 M, 50 mL). The precipitated product was filtrated from the organic layer and the title compound (**17b**, 1.83 mg, 5.68 mmol, 54%) was obtained as yellowish solid.

^{1}H NMR (400 MHz, DMSO-d_6) δ [ppm] = 12.85 (s, 1 H), 8.90 (s, 1 H), 8.34 (dd, *J* = 1.2, 8.2 Hz, 1 H), 7.97 (d, *J* = 8.7 Hz, 2 H), 7.87 (d, *J* = 8.7 Hz, 2 H) 7.83 (ddd, *J* = 1.3, 5.5, 7.0 Hz, 1 H), 7.76 (d, *J* = 7.8 Hz, 1 H). **^{13}C NMR** (101 MHz, DMSO-d_6) δ [ppm] = 176.8, 166.3, 163.8, 145.1, 143.6, 139.8, 133.6, 131.0, 126.4, 125.9, 125.9, 124.5, 119.9, 119.5, 110.6, 52.4. **HRMS** (ESI): *m*/*z* calculated for $C_{16}H_{13}N_2O_4$ [*M*+H]+ 323.1026; found 323.1024.

4-(4-quinolone-3-carboxamido)benzoic acid (**17c**)

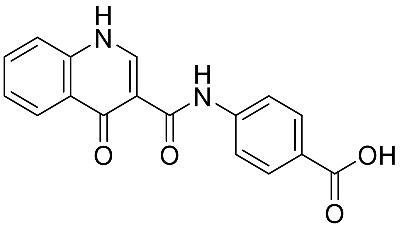

The methyl ester (**17b**, 37.6 mg, 0.112 mmol, 1.00 eq.) was dissolved in a mixture of THF/MeOH (1:1, 10 mL) and an aqueous solution of KOH (3 M, 5 mL) solution was added. The reaction mixture was stirred for 3 h, after which the organic solvent was removed under reduced pressure by rotary evaporation. The residue was treated with aqueous HCl (4 M, 5 mL) and the precipitated product (**17c**, 21.1 mg, 68.5 µmol, 61%) was filtrated and obtained as yellowish solid.

^{1}H NMR (400 MHz, DMSO-d_6) δ [ppm] = 13.29 (d, J = 6.1 Hz, 1 H), 12.80 (s, 1 H), 8.88 (d, J = 6.8 Hz, 1 H), 8.34 (d, J = 7.8 Hz, 1 H), 7.47 (d, J = 8.6 Hz, 1 H), 7.96 (d, J = 7.3 Hz, 2 H) 7.88 – 7.89 (m, 4 H). 7.55 (dd, J = 1.63, 6.4, 8.1 Hz, 1 H). **^{13}C NMR** (101 MHz, DMSO-d_6) δ [ppm] = 176.9, 167.4, 163.7, 144.7, 143.3, 139.6, 133.6, 131.1, 126.4, 125.9, 125.7, 119.7, 119.4, 110.7. **HRMS** (ESI): m/z calculated for $C_{16}H_{13}N_2O_4$ [M+H]$^+$ 309.0870; found 309.0872.

Albicidin derivative (**17**)

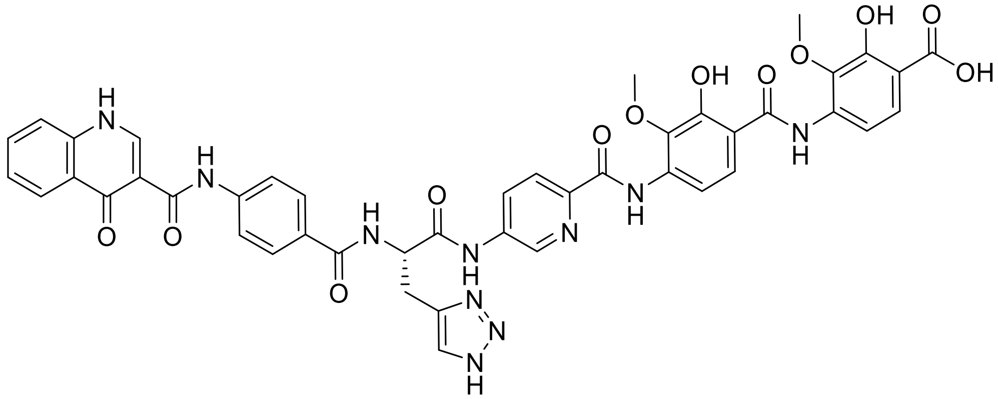

The synthesis of the product was conducted according to coupling protocol A. The albicidin derivative **17** (6 mg, 7% over two steps) was obtained as a colourless solid.

^{1}H NMR (DMSO-d_6, 700 MHz): δ [ppm] = 14.67 (s, 1 H), 13.10 (s, 1 H), 12.74 (s, 1 H), 11.76 (s, 1 H), 11.11 (s, 1 H), 10.86 (s, 1 H), 10.50 (s, 1 H), 8.98 (d, J = 1.9 Hz, 1 H), 8.91 (d, J = 6.3 Hz, 1 H), 8.83 (d, J = 5.6 Hz, 1 H), 8.35 (d, J = 8.2 Hz, 1 H), 8.21 (d, J = 8.6 Hz, 1 H), 8.11 (d, J = 8.1, 1 H), 8.00 (d, 1 H), 7.94 (d, J = 8.4 Hz, 2 H), 7.88 (d, J = 8.4 Hz, 1 H), 7.85 (d, J = 8.6 Hz, 2 H), 7.84 (d, J = 7.4 Hz, 1 H), 7.78 (d, J = 8.3 Hz, 1 H), 7.67 (b, 1 H), 7.60 – 7.54 (m, 2 H), 4.95 (dd, J = 7.5 Hz, 1 H), 3.92 (s, 3 H), 3.88 (s, 3 H), 3.30 – 3.25 (m, 2 H). **^{13}C NMR** from HSQC-ed (DMSO-d_6, 176 MHz): δ [ppm] = 144.8, 140.1, 133.6, 133.5, 129.2, 127.7, 127.0, 126.0, 125.9, 123.4, 119.7 119.2, 110.7, 110.6, 60.8, 54.7, 40.4, 34.7, 27.8 ppm. **HRMS** (ESI): m/z calculated for $C_{44}H_{37}N_{10}O_{12}$ [M+H]$^+$ 897.2509; found 897.2563, t_R = 7.98 min.

Synthesis of albicidin derivative (**18**)

***t*-butyl 4-(1-ethyl-7-methyl-4-oxo-1,4-dihydro-1,8-naphthyridine-3-carboxamido)benzoate** (**18b**)

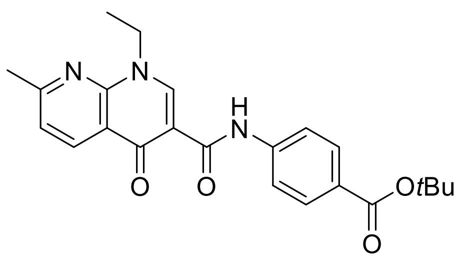

To a solution of nalidixic acid (**18a**, 1.00 g, 4.31 mmol, 1.00 eq.) in DMF (10 mL) was added HATU (2.46 g, 6.46 mmol, 1.50 eq.). The reaction mixture was stirred for 1 h at room temperature after which *tert*-butyl 4-aminobenzoate (**25a**, 1.25 g, 6.46 mmol, 1.50 eq.) and DIPEA (3.00 mL, 2.23 g, 17.2 mmol, 4.00 eq.) was added and the reaction mixture was stirred overnight at room temperature. The reaction mixture was diluted with EtOAc (50 mL) washed with an aqueous solution of HCl (1 M, 3 × 50 mL) and brine (50 mL). The organic layer was dried over Na_2SO_4, filtrated and the organic solvent was removed under reduced pressure by rotary evaporation. The crude product was purified by flash column chromatography (SiO_2, n-hexane/EtOAc, 7:1) and the title compound (**18b**, 1.55 mg, 3.80 mmol, 88%) was obtained as white solid.

^{1}H NMR (500 MHz, DMSO-d_6): δ [ppm] = 12.58 (s, 1 H), 9.21 (s, 1 H), 8.70 (d, *J* = 7.8 Hz, 2 H), 7.97 (d, *J* = 8.6 Hz, 2 H), 7.90 (d, *J* = 8.6 Hz, 2 H), 7.62 (d, *J* = 8.2 Hz, 1 H), 4.70 (q, *J* = 7.3 Hz, 2 H), 2.76 (s, 3 H), 1.61 (s, 9 H), 1.49 (t, *J* = 7.1 Hz, 3 H). **^{13}C NMR** (101 MHz, DMSO-d_6) δ [ppm] = 177.2, 164.9, 158.4, 156.1, 152.1, 142.1, 130.5, 127.8, 125.5, 125.4, 123.8, 123.6, 122.5, 122.4, 120.8, 111.0, 110.2, 110.0. **HRMS** (ESI): *m*/*z* calculated for $C_{23}H_{26}N_3O_4$ $[M+H]^+$ 408.1918; found 408.1907.

4-(1-ethyl-7-methyl-4-oxo-1,4-dihydro-1,8-naphthyridine-3-carboxamido)benzoic acid (**18c**)

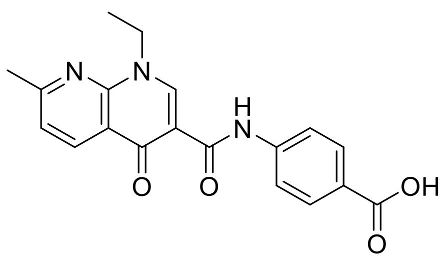

The *tert*-butyl ester (**18b**, 150 mg, 0.368 mmol, 1.00 eq.) was dissolved in a mixture of TFA/H_2O/TIS (95:2.5:2.5, 9 mL) and the reaction mixture was stirred for 2.5 h at room temperature, after which Et_2O was added and the precipitated product was centrifuged. The supernatant was decanted and the residue was lyophilized. The title compound (**18c**, 110 mg, 0.313 mmol, 85%) was obtained as white solid.

^{1}H NMR (500 MHz, DMSO-d_6): δ [ppm] = 10.99 (s, 1 H), 8.00 (d, *J* = 8.2 Hz, 2 H), 7.97 – 7.93 (m, 3 H), 7.84 (ddd, *J* = 3.0, 8.3 Hz, 1 H), 7.77 (dd, *J* = 3.0, 8.4 Hz, 1 H), 7.02 (s, 1 H). **^{13}C NMR** (101 MHz, DMSO-d_6): δ [ppm] = 177.2, 167.2, 159.2, 158, 8, 158.7, 158.4, 156.1, 152.1, 142.0, 130.8, 127.3, 123.8, 123.6, 122.4, 122.3, 120.8, 119.4, 117.1, 114.8, 111.0, 110.2, 110.0, 60.2, 21.2, 14.5. **HRMS** (ESI): *m*/*z* calculated for $C_{19}H_{18}N_3O_4$ $[M+H]^+$ 352.1292; found 352.1287.

Albicidin derivative (**18**)

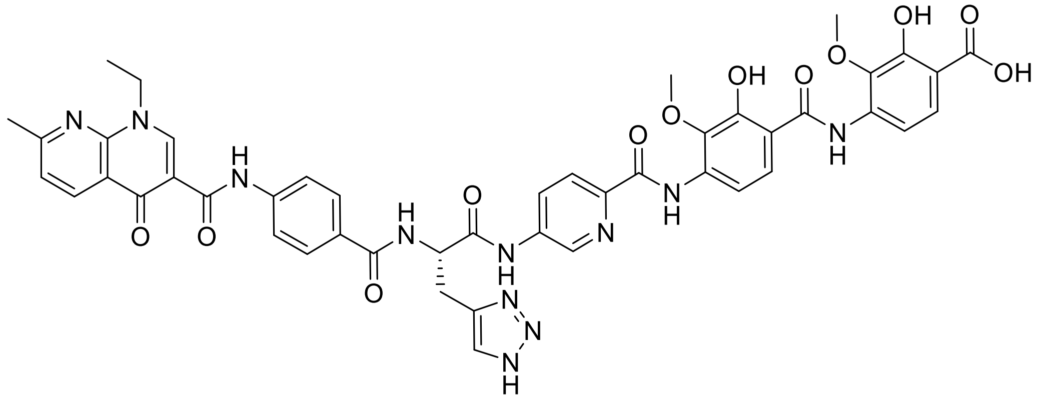

The synthesis of the product was conducted according to coupling protocol A. The albicidin derivative **18** (9.7 mg, 9% over two steps) was obtained as a colourless solid. **^{1}H NMR** (DMSO-*d*$_6$, 500 MHz): δ [ppm] = 12.46 (s, 1 H), 11.78 (s, 1 H), 10.99 (s, 1 H), 10.88 (s, 1 H), 10.49 (s, 1 H), 9.16 (s, 1 H), 8.98 (s, 1 H), 8.84 (d, *J* = 7.4 Hz, 1 H), 8.64 (d, *J* = 4.9 Hz, 1 H), 8.35 (d, *J* = 8.6 Hz, 1 H), 8.22 (d, *J* = 8.6 Hz, 1 H), 8.10 (d, *J* = 8.1 Hz, 1 H), 7.98 – 7.80 (m, 5H), 7.66 (s, 1 H), 7.56 (d, *J* = 8.6 Hz, 1 H), 4.95 (dd, *J* = 7.5 Hz, 1 H), 4.65 (q, 2 H), 3.90 (s, 3 H), 3.88 (s, 3 H), 2.70 (s, 3 H), 1.44 (t, 3 H). **^{13}C NMR** from HSQC-ed (DMSO-*d*$_6$, 176 MHz): δ [ppm] = 140.0, 136.4, 129.2, 127.8, 126.9, 123.5, 122.6, 119.3, 109.9, 110.3, 61.3, 60.7, 46.8, 25.4, 15.4. **HRMS** (ESI): *m/z* calculated for $C_{44}H_{37}N_{10}O_{11}$ [*M*+H]$^+$ 940.3009; found 940.3004, t_R = 9.24 min.

Synthesis of albicidin derivative (**19**)

methyl 4-(5-ethyl-8-oxo-5,8-dihydro-[1,3]dioxolo[4,5-g]quinoline-7-carboxamido)benzoate (**19b**)

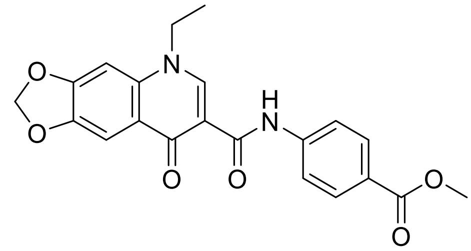

To a solution of 5-ethyl-8-oxo-5,8-dihydro-[1,3]dioxolo[4,5-g]quinoline-7-carboxylic acid (**19a**, 2.00 g, 7.66 mmol, 1.00 eq.) in DMF (20 mL) was added HATU (4.37 g, 11.5 mmol, 1.50 eq.), and DIPEA (3.97 mL, 2.97 g, 23.0 mmol, 3.00 eq.). The reaction mixture was stirred for 1 h at room temperature after which methyl 4-aminobenzoate (1.74 g, 11.5 mmol, 1.50 eq.) was added and the reaction mixture was stirred overnight at room temperature. The reaction mixture was diluted with EtOAc (200 mL) washed with a saturated aqueous solution of $NaHCO_3$ (3 × 200 mL) and brine (200 mL), dried over $MgSO_4$ and concentrated under reduced pressure by rotary evaporation. The crude product was purified flash column chromatography (SiO_2, hexane/ethyl acetate 8:2) to yield the title compound (**19b**, 2.74 mg, 1.80 mmol, 63%) as a colourless solid.

^{1}H NMR (400 MHz, $CDCl_3$) δ [ppm] = 12.63 (s, 1 H), 8.68 (br. s, 1 H), 7.96 (d, *J* = 8.5 Hz, 2 H), 7.80 (s, 1 H), 7.77 (d, *J* = 8.6 Hz, 2 H), 6.87 (s, 1 H), 6.09 (s, 2 H), 6.62 (dd, *J* = 8.6, 2.2 Hz, 1 H), 4.23 (q, *J* = 7.1 Hz, 2 H), 3.84 (s, 3 H), 1.51 (t, J = 7.3 Hz, 3 H). **^{13}C NMR** (101 MHz, $CDCl_3$) δ [ppm] = 166.9, 145.9, 136.2, 130.7, 119.6, 104.3, 102.7, 95.2, 51.9, 50.0, 14.6. **HRMS** (ESI): *m*/*z* calculated for $C_{16}H_{13}N_2O_4$ [*M*+H]$^+$ 395.1238; found 395.1228.

4-(5-ethyl-8-oxo-5,8-dihydro-[1,3]dioxolo[4,5-g]quinoline-7-carboxamido)benzoic acid (**19c**)

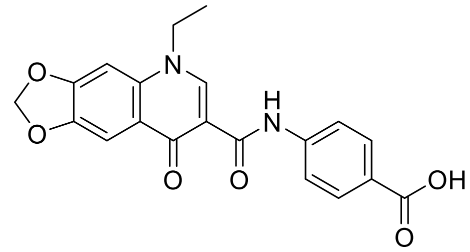

The methyl ester (**19b**, 1.00 g, 2.53 mmol, 1.00 eq.) was dissolved in a mixture of THF/MeOH (1:1, 40 mL) and an aqueous solution of KOH (5 M, 5 mL) solution was added. The reaction mixture was stirred for 3 h, after which the organic solvent was removed under reduced pressure by rotary evaporation. The residue was treated with an aqueous solution of HCl (6 M, 5 mL) and the precipitated product (**19c**, 964 mg, 2.53 mmol, quant.) was filtrated and obtained as brownish solid.

HRMS (ESI): *m*/*z* calculated for $C_{16}H_{13}N_2O_4$ [*M*+H]$^+$ 381.1081; found 381.1079.

Albicidin derivative (**19**)

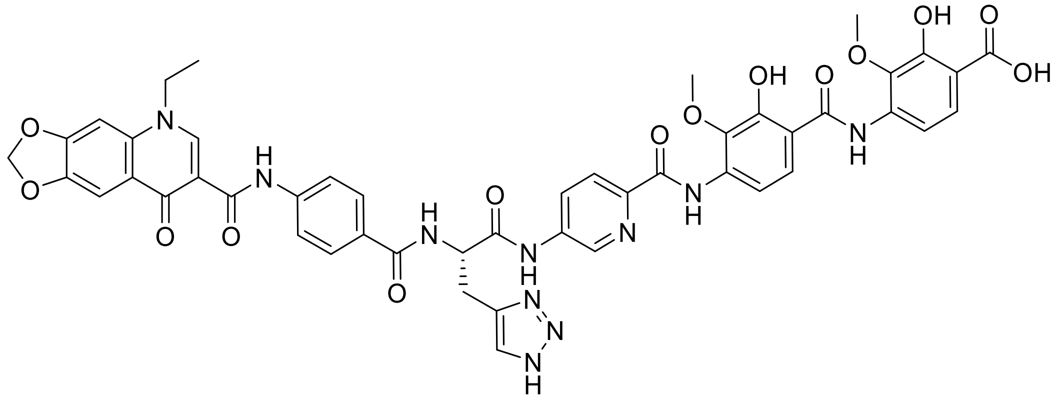

The synthesis of the product was conducted according to coupling protocol A. The albicidin derivative **19** (18 mg, 21% over two steps) was obtained as a colourless solid. **^{1}H NMR** from HSQC-ed (DMSO-*d*$_6$, 700 MHz): δ [ppm] = 14.66 (s, 1 H), 11.87 (s, 1 H), 11.72 (s, 1 H), 11.13 (s, 1 H), 10.84 (d, *J* = 7.2 Hz, 2 H), 10.50 (s, 1 H), 8.98 (d, *J* = 2.1 Hz, 1 H), 8.90 (s, 1 H), 8.82 (d, *J* = 4.9 Hz, 1 H), 8.34 (dd, *J* = 8.6, 2.1 Hz, 1 H), 8.21 (d, *J* = 8.6 Hz, 1 H), 8.12 (d, *J* = 8.9, 1 H), 8.03 (d, *J* = 8.8, 1 H), 7.92 (d, *J* = 8.7 Hz, 2 H), 7.88 (d, *J* = 8.9 Hz, 1 H), 7.83 (d, *J* = 8.7 Hz, 2 H), 7.72 (s, 1 H), 7.67 (b, 1 H), 7.60 – 7.59 (m, 2 H2 H), 6.28 (s, 2 H), 4.94 (dd, *J* = 7.5 Hz, 1 H), 4.53 (q, *J* = 7.2 Hz, 2 H), 3.92 (s, 3 H), 3.88 (s, 3 H),

3.27 (dd, J = 14.7, 5.7 Hz, 1 H), 3.28 (dd, J = 14.8, 9.2 Hz, 1 H), 1.40 (t, J = 7.2 Hz, 3 H). **^{13}C NMR** from HSQC-ed (DMSO-d_6, 176 MHz): δ [ppm] = 146.8, 139.5, 129.3, 127.6, 127.1, 126.1, 123.5, 119.1, 110.7, 110.5, 103.0, 102.8, 97.4, 61.3, 60.7, 54.7, 49.7, 15.2. **HRMS** (ESI): m/z calculated for $C_{47}H_{41}N_{10}O_{14}$ $[M+H]^+$ 969.2786; found 969.2798, t_R = 8.45 min.

Synthesis of albicidin derivative (**20**)

***tert*-butyl 4-(6-fluoro-4-oxo-4H-chromene-2-carboxamido)benzoate** (**20b**)

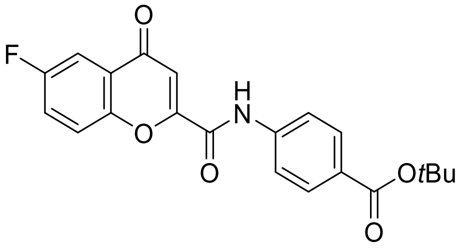

4-(6-fluoro-4-oxo-4H-chromene-2-carboxilic acid (**20a**, 1.00 g, 4.83 mmol, 1.00 eq.) was dissolved in *n*-hexane (10 mL) and PCl_5 (550 mg, 2.64 mmol, 1.10 eq.) was added. The reaction mixture was stirred 1 h at 70°C. The volatiles were removed under reduced pressure by rotary evaporation and the residue was dissolved in dry THF (10 mL), *tert*-butyl 4-aminobenzoate (10.7 g, 5.53 mmol, 1.15 eq.) and triethylamine (2.01 mL, 1.46 g, 14.4 mmol, 3 eq.) were added and the reaction mixture was stirred at room temperature overnight. The reaction mixture was diluted by 50 mL EtOAc and the organic layer was washed with an aqueous solution of HCl (1 M, 2 × 50 mL) and brine (50 mL) and dried over Na_2SO_4 and filtrated. The organic solvent was removed under reduced pressure by rotary evaporation and the crude product was purified by flash column chromatography (SiO_2, n-hexane/EtOAc, 6:1). The title compound (**20b**, 740 mg, 1.93 mmol, 40%) was obtained as yellowish solid.

^{1}H NMR (500 MHz, DMSO-d_6): δ [ppm] = 11.08 (s, 1 H), 8.01 – 7.95 (m, 5 H), 7.85 (ddd, J = 3.0, 8.3 Hz, 1 H), 7.77 (dd, J = 3.0, 8.4 Hz, 1 H), 7.02 (s, 1 H), 1.56 (s, 9 H). **^{13}C NMR** (101 MHz, DMSO-d_6): δ [ppm] = 177.2, 164.9, 158.4, 156.1, 152.1, 142.1, 130.5, 127.8, 125.5, 125.4, 123.8, 123.6, 122.5, 122.5, 122.4, 120.8, 111.0, 110.2, 110.0. **HRMS** (ESI): *m*/*z* calculated for $C_{21}H_{18}FNO_5$ $[M+H]^+$ 384.1241; found 384.1236.

4-(6-fluoro-4-oxo-4H-chromene-2-carboxamido)benzoic acid (**20c**)

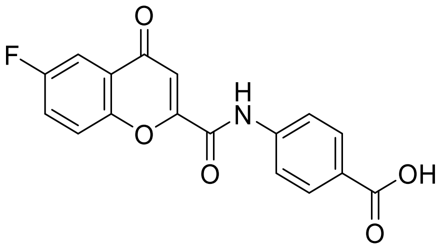

The *tert*-butyl ester (**20b**, 300 mg, 0.783 mmol, 1 eq.) was dissolved in a mixture of TFA and CH_2Cl_2 (1:1, 10 mL) and the reaction mixture was stirred for 2 h at room temperature. After completion of the reaction the volatiles were removed under reduced pressure by rotary evaporation and the residue was lyophilized. The title compound (**20c**, 235 mg, 718 mmol, 92%) was obtained as yellowish solid.

^{1}H NMR (500 MHz, DMSO-d_6): δ [ppm] = 10.99 (s, 1 H), 8.00 (d, *J* = 8.2 Hz, 2 H), 7.97 – 7.93 (m, 3 H), 7.84 (ddd, *J* = 3.0, 8.3 Hz, 1 H), 7.77 (dd, *J* = 3.0, 8.4 Hz, 1 H), 7.02 (s, 1 H). **^{13}C NMR** (101 MHz, DMSO-d_6): δ [ppm] = 177.2, 167,2, 159.2, 158, 8, 158.7, 158.4, 156.1, 152.1, 142.0, 130,.8, 127.3, 123.8, 123.6, 122.4, 122.3, 120.8, 119.4, 117.1, 114.8, 111.0, 110.2, 110.0. **HRMS** (ESI): *m*/*z* calculated for $C_{17}H_{11}N_2O_5$ [*M*+H]$^+$ 328.0616; found 328.0614.

Albicidin derivative (**20**)

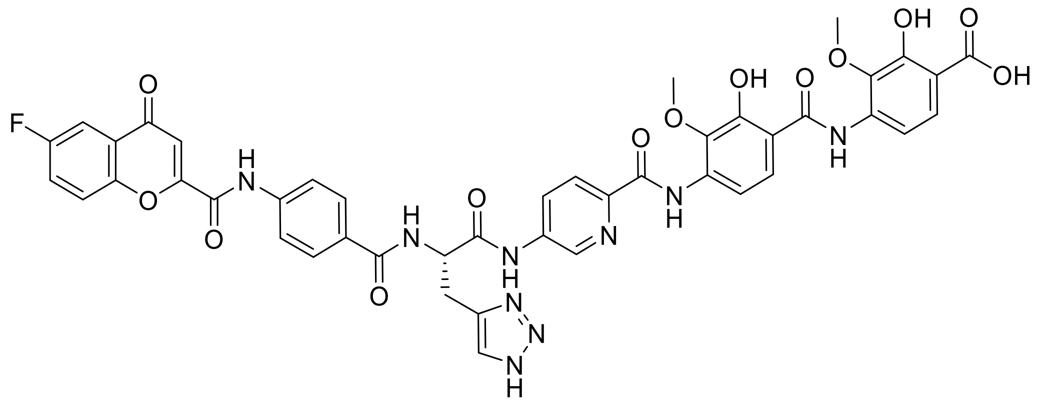

The synthesis of the product was conducted according to coupling protocol A. The albicidin derivative (**20**, 10.3 mg, 12%, over two steps) was obtained as a colourless solid. **^{1}H NMR** (DMSO-d_6, 700 MHz): δ [ppm] = 11.73 (s, 1 H), 11.10 (s, 1 H), 10.97 (s, 1 H), 10.89 (s, 1 H), 10.49 (s, 1 H), 8.98 (s, 1 H), 8.89 (d, *J* = 6.0 Hz, 1 H), 8.85 (d, *J* = 7.4 Hz, 1 H), 8.34 (dd, *J* = 8.6, 2.4 Hz, 1 H), 8.20 (d, *J* = 8.6 Hz, 1 H), 8.10 (d, *J* = 8.1, 1 H), 8.02 – 7.91 (m, 5H), 7.90 – 7.81 (m, 2 H), 7.78 (d, *J* = 8.1, Hz, 1 H), 7.68 (s, 1 H), 7.58 (d, *J* = 8.1, 1 H), 7.02, (s, 1 H), 4.96 (dd, *J* = 7.5 Hz, 1 H), 3.91 (s, 3 H), 3.87 (s, 3 H), 3.21 – 3.06 (m, 2 H). **^{13}C NMR** from HSQC-ed (DMSO-d_6, 176 MHz): δ [ppm] = 140.0, 128.9, 127.9, 126.9, 126.2, 123.7, 123.4, 121.2, 120.9, 119.9, 11.0, 110.9, 110.8, 110.2, 61.3, 60.7, 54.8, 27.9. **HRMS** (ESI): *m/z* calculated for $C_{44}H_{37}N_{10}O_{11}$ [*M*+H]$^+$ 916.2333; found 916.2321. t_R = 8.60 min.

Synthesis of albicidin derivative (**21**)

Methyl 4-(6-hydroxy-1H-indole-2-carboxamido)benzoate (**21b**)

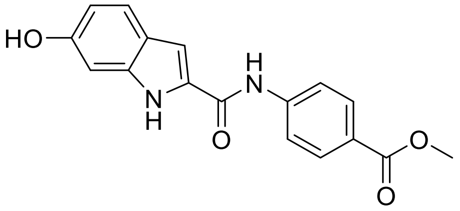

To a solution of 6-hydroxy-1H-indole-2-carboxylic acid (**21a**, 450 mg, 2.56 mmol, 1.00 eq.) in THF (5 mL) oxalyl chloride (651 mg, 440 µL, 5.12 mmol, 2.00 eq.) was added. The reaction mixture was stirred at room temperature for 2 h. The volatiles were evaporated to yield 6-hydroxy-1H-indole-2-carbonyl chloride (500 mg, 2.56 mmol, quant.) as a yellowish solid that was used without further purification.

Methyl 4-aminobenzoate (**25b**, 310 mg, 2.40 mmol, 1.00 eq.) was dissolved in dry THF (5 mL) and DIPEA (300 µL) was added. The solution was cooled to 0°C and of the acid chloride (**x**, 500 mg, 2.56 mmol, 1.25 eq.) in dry THF (5 mL) was added. The reaction was stirred overnight and quenched with an aqueous solution of HCl (1 M, 10 mL). The aqueous layer was extracted with EtOAc (3 × 10 mL) and the combined organic layers were washed with 1 M $NaHCO_3$ (10 mL) and brine (10 mL), dried over anhydrous Na_2SO_4, filtrated and concentrated under reduced pressure. The crude product was purified using C18 reverse phase chromatography (MeCN/H2O 5% to 100% in 30 min) to yield methyl 4-(6-hydroxy-1H-indole-2-carboxamido)benzoate (**21b**, 300 mg, 0.97 mmol, 38%) as off-white solid.

^{1}H NMR (500 MHz, DMSO-d_6): δ [ppm] = 11.37 (d, J = 2.3 Hz, 1 H), 10.31 (s, 1 H), 9.31 (s, 1 H), 7.96 (s, 4 H), 7.47 (d, J = 8.6 Hz, 1 H), 7.38 (d, J = 2.3 Hz, 1 H), 6.82 (d, J = 2.2 Hz, 1 H), 6.62 (dd, J = 8.6, 2.2 Hz, 1 H), 3.84 (s, 3 H). **HRMS** (ESI): m/z calculated for $C_{16}H_{13}N_2O_4$ $[M+H]^+$ 311.1026; found 311.1024.

4-(6-hydroxy-1H-indole-2-carboxamido)benzoic acid (**21c**)

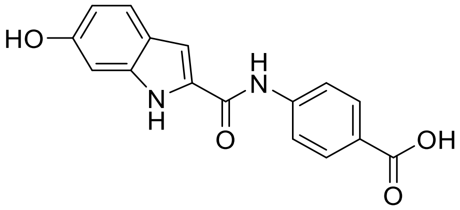

Methyl ester (**21b**, 300 mg, 0.965 mmol, 1.00 eq.) was dissolved in THF (3 mL), cooled to 0°C and an aqueous solution of KOH (4 M, 1 mL) was added. The reaction was stirred for 30 minutes and quenched with an aqueous solution of HCl (6 M, 1 mL). The product precipitated as a brownish solid that was separated by centrifugation to yield target compound (**21c**, 240 mg, 0.811 mmol, 84% yield).

^{1}H NMR (500 MHz, DMSO-d_6): δ [ppm] = 12.70 (s, 1 H), 11.40 (d, J = 2.3 Hz, 1 H), 10.32 (s, 1 H), 9.31 (s, 1 H), 7.94 (s, 4 H), 7.47 (d, J = 8.6 Hz, 1 H), 7.38 (d, J = 2.3 Hz, 1 H), 6.82 (d, J = 2.2 Hz, 1 H), 6.62 (dd, J = 8.6, 2.2 Hz, 1 H). **^{13}C NMR** (126 MHz, DMSO-d_6): δ [ppm] = 167.5, 160.5, 155.9, 139.0, 130.8, 129.9, 125.5, 123.0, 121.0, 119.5, 112.3, 105.7, 96.9. **HRMS** (ESI): m/z calculated for $C_{16}H_{13}N_2O_4$ $[M+H]^+$ 297.0870; found 297.0865.

Albicidin derivative (**21**)

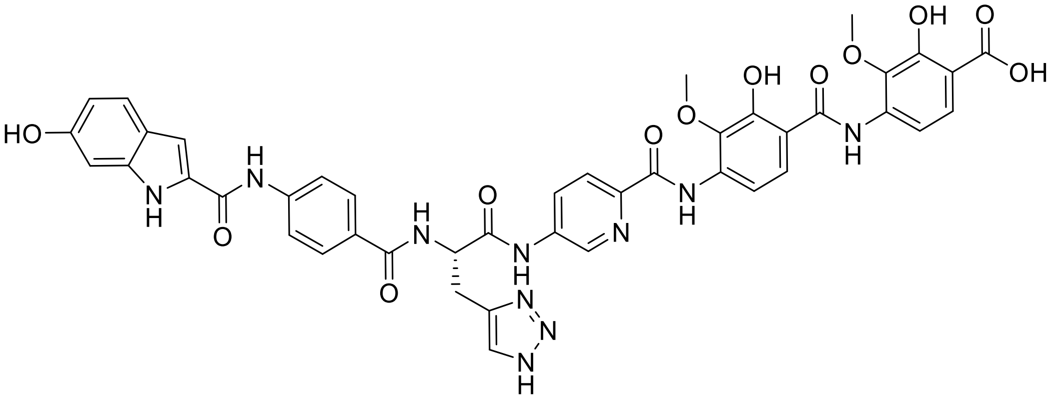

The synthesis of the product was conducted according to coupling protocol A. The albicidin derivative was obtained as a colourless solid (**21**, 10 mg, 16% over two steps).

^{1}H NMR (DMSO-d_6, 700 MHz): δ [ppm] = 11.38 (s, 1 H) 10.85 (s, 1 H), 10.48 (s, 1 H), 10.26 (s, 1 H), 9.32 (s, 1 H), 8.97 (d, *J* = 2.5 Hz, 1 H), 8.79 (d, *J* = 7.6 Hz, 1 H), 8.35 (dd, *J* = 8.5, 2.4 Hz, 1 H), 8.21 (d, *J* = 8.5 Hz, 1 H), 8.02 (m, 1 H), 7.96 (s, 2 H), 7.95 – 7.86 (m, 4 H), 7.84 (d, *J* = 7.9 Hz, 2 H), 7.72 (m, 2 H), 7.54 (s, 1 H), 7.47 (d, *J* = 8.6 Hz, 1 H), 7.40 (d, *J* = 8.8 Hz, 1 H), 7.37 (d, *J* = 2.4 Hz, 1 H), 7.29 – 7.25 (m, 1 H), 7.24 – 7.19 (m, 2 H), 6.82 (d, *J* = 2.1 Hz, 1 H), 6.62 (dd, *J* = 8.6, 2.2 Hz, 1 H), 6.56 (s, 1 H), 4.94 (dd, *J* = 7.6, 14.3 Hz, 1 H), 3.89 (s, 3 H), 3.86 (s, 3 H). **^{13}C NMR** from HSQC-ed (DMSO-d_6, 176 MHz): δ [ppm] = 162.7, 140.0, 129.0, 128.8, 127.9, 127.6, 126.2, 124.9, 123.3, 123.0, 119.4, 112.2, 108.3, 105.3, 96.9, 61.0, 59.9, 54.7. **HRMS** (ESI): m/z calculated for $C_{43}H_{37}N_{10}O_{12}$ $[M+H]^+$ 885.2587; found 885.2590, t_R = 7.50 min

Synthesis of albicidin derivative (**22**)

methyl 4-(benzo[d]thiazole-6-carboxamido)benzoate (**22b**)

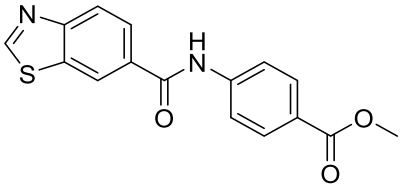

To a solution of benzo[d]thiazole-6-carboxylic acid (**22a**, 100 mg, 558 mmol, 1.30 eq.) in DMF (5 mL) was added HATU (276 mg, 0.725 mmol, 1.30 eq.), and DIPEA (0.260 mL, 193 mg, 1.67 mmol, 3.00 eq.). The reaction mixture was stirred for 1 h at room temperature after which methyl 4-aminobenzoate (**25b**, 110 mg, 0.725 mmol, 1.30 eq.) was added and the reaction mixture was stirred overnight at room temperature. The reaction mixture was diluted with EtOAc (50 mL) washed with a saturated aqueous solution of $NaHCO_3$ (3 × 50 mL) and brine (50 mL), dried over $MgSO_4$ and concentrated under reduced pressure by rotary evaporation. The crude product was purified flash column chromatography (SiO_2, hexane/ethyl acetate 8:2) to yield the title compound (**22b**, 103 mg, 0.331 mmol, 59%) as a brownish solid.

^{1}H NMR (400 MHz, DMSO-d_6): δ [ppm] = 10.72 (s, 1 H), 9.59 (s, 1 H), 8.81 (d, *J* = 1.7 Hz, 1 H), 8.24 (d, *J* = 8.7 Hz, 1 H), 8.12 (dd, *J* = 1.6, 8.7 Hz, 1 H), 7.98 (br. s, 4 H), 3.85 (s, 3 H). **^{13}C NMR** (101 MHz, DMSO-d_6): δ [ppm] = 165.8, 165.5, 159.4, 155.0, 143.6, 133.7, 131.7, 130.1, 125.9, 124.4, 122.8, 119.6, 51.9. **HRMS** (ESI): *m*/*z* calculated for $C_{16}H_{13}N_2O_4$ [*M*+H]$^+$ 313.0641; found 313.0631.

4-(benzo[d]thiazole-6-carboxamido)benzoic acid (**22c**)

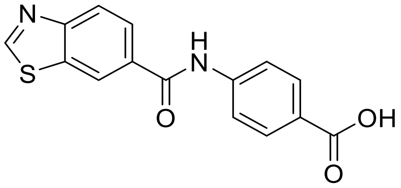

The methyl ester (**22b**, 45.3 mg, 0.145 mmol, 1.00 eq.) was dissolved in a mixture of THF/MeOH (1:1, 10 mL) and an aqueous solution of KOH (3 M, 5 mL) solution was added. The reaction mixture was stirred for 3 h, after which the organic solvent was removed under reduced pressure by rotary evaporation. The residue was treated with an aqueous solution of HCl (4 M, 5 mL) and the precipitated product (**22c**, 43.3 mg, 0.145 mmol, quant.) was filtrated and obtained as brownish solid.

^{1}H NMR (400 MHz, DMSO-d_6): δ [ppm] = 10.77 (s, 1 H), 9.59 (s, 1 H), 8.85 (d, *J* = 1.7 Hz, 1 H), 8.23 (d, *J* = 8.7 Hz, 1 H), 8.14 (dd, *J* = 1.6, 8.7 Hz, 1 H), 7.97 (br. s, 4 H). **^{13}C NMR** (101 MHz, DMSO-d_6): δ [ppm] = 166.9, 165.4, 159.9, 154.9, 143.3, 133.6, 131.7, 130.2, 125.9, 125.6, 122.8, 119.5. **HRMS** (ESI): *m*/*z* calculated for $C_{16}H_{13}N_2O_4$ [*M*+H]$^+$ 299.0485; found 299.0492.

Albicidin derivative (**22**)

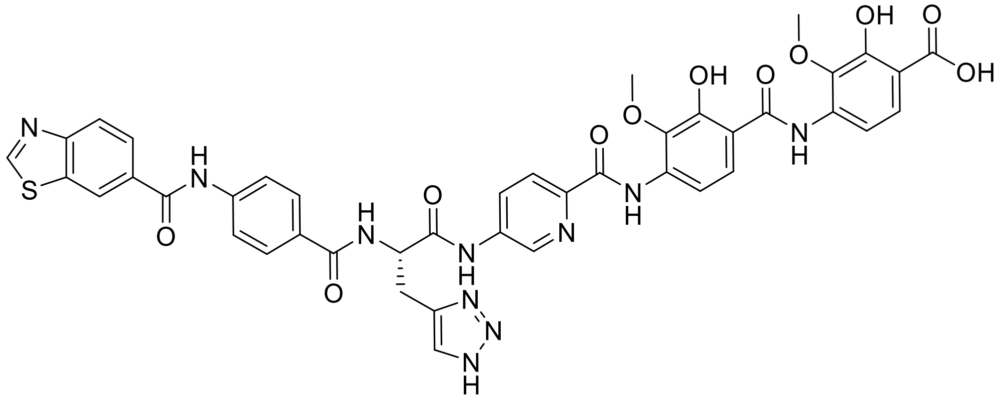

The synthesis of the product was conducted according to coupling protocol A. The albicidin derivative **22** (6 mg, 7% over two steps) was obtained as a colourless solid.

^{1}H NMR (DMSO-d_6, 700 MHz): δ [ppm] = 11.73 (s, 1 H), 11.60 (s, 1 H), 11.14 (s, 1 H), 10.86 (d, *J* = 7.2 Hz, 2 H), 10.65 (s, 1 H), 10.50 (s, 1 H), 9.60 (s, 1 H), 8.98 (d, *J* = 2.0 Hz, H), 8.83 (d, *J* = 1.5 Hz, 1 H), 8.35 (dd, *J* = 8.6, 2.4 Hz, 1 H), 8.24 (d, *J* = 8.9 Hz, 1 H), 8.22 (d, *J* = 8.6 Hz, 1 H), 8.14 (dd, *J* = 8.5, 1.7 Hz, 1 H), 8.12 (d, *J* = 8.9, 1 H), 8.04 (d, *J* = 8.9 Hz, 1 H), 7.93 (s, 4 H), 7.89 (d, *J* = 8.9 Hz, 1 H), 7.72 (s,

1 H), 7.60 (d, J = 8.7, 1 H), 4.96 (dd, J = 7.5 Hz, 1 H), 3.93 (s, 3 H), 3.88 (s, 3 H), 3.35 (dd, J = 14.7, 5.7 Hz, 1 H), 3.28 (dd, J = 14.8, 9.2 Hz, 1 H). **^{13}C NMR** from HSQC-ed (DMSO-d_6, 176 MHz): δ [ppm] = 159.9, 139.8, 128.8, 127.7, 127.0, 126.3, 126.2, 123.2, 123.1, 119.9, 110.9, 110.7, 61.3, 60.7, 54.8, 27.7. **HRMS** (ESI): *m/z* calculated for $C_{42}H_{35}N_{10}O_{11}S$ $[M+H]^+$ 887.2192; found 887.2198, t_R = 7.65 min.

Synthesis of albicidin derivative (**23**)

Methyl 4-(1H-benzo[d]imidazole-6-carboxamido)benzoate (**23b**)

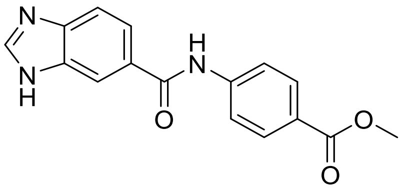

To a suspension of 1H-benzo[d]imidazole-6-carboxylic acid (**23a**, 1.00 g, 6.16 mmol, 1.30 eq.) in toluene (50 mL) was added $SOCl_2$ (1.72 mL, 2.82 g, 23.7 mmol, 5.00 eq.) and the reaction mixture was stirred at 80°C overnight. The volatiles were removed under reduced pressure by rotary evaporation and the residue was dissolved in THF (20 mL). To this solution was added methyl 4-aminobenzoate (717 mg, 4.74 mmol, 1.00 eq.) and triethylamine (1.98 mL, 1.44 g, 14.2 mmol, 3.00 eq.) and the reaction mixture was stirred overnight at room temperature. The reaction mixture was diluted with EtOAc (50 mL) washed with a saturated aqueous solution of $NaHCO_3$ (3 × 50 mL) and brine (50 mL), dried over $MgSO_4$ and concentrated under reduced pressure by rotary evaporation. The crude product was purified flash column chromatography (SiO_2, hexane/ethyl acetate 1:2) to yield the title compound (**23b**, 726 mg, 2.46 mmol, 52%) as a colourless solid.

^{1}H NMR (400 MHz, DMSO-d_6): δ [ppm] = 10.57 (s, 1 H), 8.46 (s, 1 H), 8.34 (d, J = 1.7 Hz, 1 H), 8.02 – 7.95 (m, 4 H), 7.89 (dd, J = 1.7, 8.7 Hz, 1 H), 7.72 (d, J = 8.3 Hz, 1 H), 3.85 (s, 3 H). **^{13}C NMR** (101 MHz, DMSO-d_6): δ [ppm] = 166.3, 165.9, 144.2, 144.0, 130.1, 128.3, 124.0, 122.2, 119.5, 51.8. **HRMS** (ESI): *m/z* calculated for $C_{16}H_{13}N_2O_4$ $[M+H]^+$ 296.1030; found 296.1022.

4-(1H-benzo[d]imidazole-6-carboxamido)benzoic acid (**23c**)

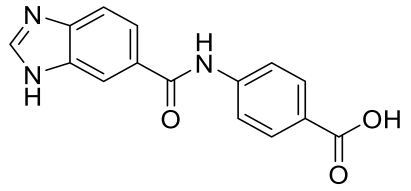

The methyl ester (**23b**, 201 mg, 0.680 mmol, 1.00 eq.) was dissolved in a mixture of THF/MeOH (1:1, 10 mL) and an aqueous solution of KOH (3 M, 5 mL) solution was added. The reaction mixture was stirred for 3 h, after which the organic solvent was removed under reduced pressure by rotary evaporation. The residue was purified by automated reverse-phase chromatography (Biotage C18, CH_3CN/H_2O 20% → 50%) and the product (**23c**, 128 mg, 0.456 mmol, 67%) was obtained as white solid.

^{1}H NMR (400 MHz, DMSO-*d*$_6$): δ [ppm] = 11.00 (s, 1 H), 9.73 (s, 1 H), 8.56 (d, *J* = 1.7 Hz, 1 H), 8.21 (dd, *J* = 1.7, 8.7 Hz, 1 H), 7.89 (m, 5 H). **^{13}C NMR** (101 MHz, DMSO-*d*$_6$): δ [ppm] = 167.2, 166.5, 142.3, 133.7, 131.0, 130.9, 128.2, 126.5, 125.7, 119.7, 116.1, 115.2, 114.6. **HRMS** (ESI): *m*/*z* calculated for $C_{16}H_{13}N_2O_4$ [*M*+H]$^+$ 282.0873; found 282.0874.

Albicidin derivative (**23**)

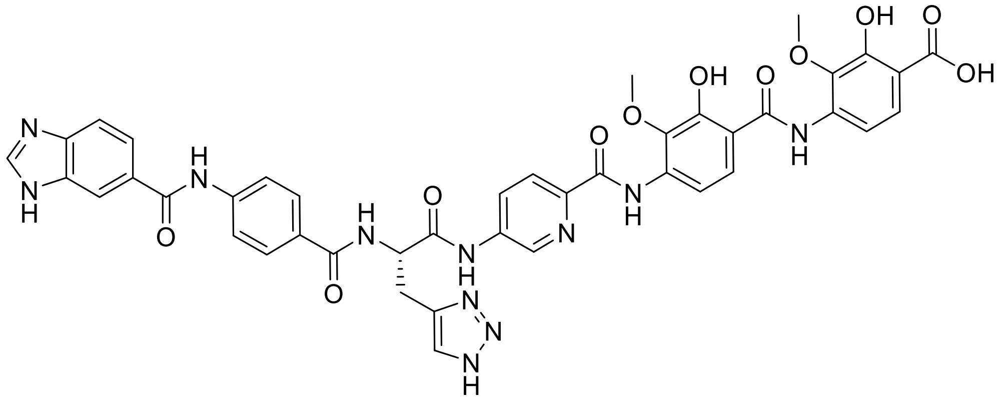

The synthesis of the product was conducted according to coupling protocol A. The albicidin derivative **23** (6 mg, 7% over two steps) was obtained as a colourless solid.

^{1}H NMR (DMSO-*d*$_6$, 700 MHz): δ [ppm] = 11.73 (s, 1 H), 11.60 (s, 1 H), 11.14 (s, 1 H), 10.86 (d, *J* = 7.2 Hz, 2 H), 10.63 (s, 1 H),10.50 (s, 1 H), 9.21 (s, 1 H), 8.98 (d, *J* = 2.2 Hz, 2 H), 8.83 (d, *J* = 7.4 Hz, 1 H), 8.44 (s, 1 H), 8.35 (dd, *J* = 8.6, 2.4 Hz, 1 H), 8.22 (d, *J* = 8.6 Hz, 1 H), 8.12 (d, *J* = 8.9 Hz, 1 H), 8.07 (d, *J* = 8.3 Hz, 1 H), 8.04 (d, *J* = 8.9 Hz, 1 H), 7.93 (s, 4 H), 7.89 (d, *J* = 8.9 Hz, 1 H), 7.72 (s, 1 H), 7.60 (d, *J* = 8.9 Hz, 1 H), 4.96 (dd, *J* = 7.5 Hz, 1 H), 3.93 (s, 3 H), 3.88 (s, 3 H), 3.35 (dd, *J* = 14.7, 5.7 Hz, 1 H), 3.28 (dd, *J* = 14.8, 9.2 Hz, 1 H). **^{13}C NMR** from HSQC-ed (DMSO-*d*$_6$, 176 MHz): δ [ppm] = 140.0, 128.9, 127.8, 127.0, 126.2, 124.7, 123.5, 119.9, 110.9, 110.7, 61.3, 60.7, 54.8, 27.7. **HRMS** (ESI): m/z calculated for $C_{42}H_{36}N_{11}O_{11}$ [*M*+H]$^+$ 870.2591; found 870.2596, t_R = 6.69 min.

Synthesis of albicidin derivative (**24**)

Methyl 4-(1H-benzo[d][1,2,3]triazole-6-carboxamido)benzoate (**24b**)

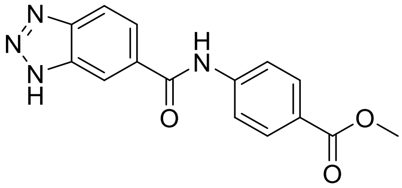

To a solution of 1H-benzo[d][1,2,3]triazole-6-carboxylic acid (**24a**, 1.00 g, 6.13 mmol, 1.30 eq.) in toluene (50 mL) was added $SOCl_2$ (1.71 mL, 2.80 g, 23.6 mmol, 5.00 eq.) and the reaction mixture was stirred at 80°C overnight after which methyl 4-aminobenzoate (713 mg, 4.72 mmol, 1.00 eq.) and triethylamine (1.97 mL, 1.43 g, 14.1 mmol, 3.00 eq.) and the reaction mixture was stirred overnight at room temperature. The reaction mixture was diluted with EtOAc (50 mL) washed with a saturated aqueous solution of $NaHCO_3$ (3 × 50 mL) and brine (50 mL), dried over $MgSO_4$ and concentrated under reduced pressure by rotary evaporation. The crude product was purified flash column chromatography (SiO_2, hexane/ethyl acetate 1:1) to yield the title compound (**24b**, 651 mg, 2.20 mmol, 36%) as a colourless solid.

^{1}H NMR (400 MHz, DMSO-d_6): δ [ppm] = 10.72 (s, 1 H), 8.67 (s, 1 H), 8.07 – 8.01 (m, 2 H), 7.99 (br. s, 4 H), 3.85 (s, 3 H). **^{13}C NMR** (101 MHz, DMSO-d_6): δ [ppm] = 165.8, 165.6, 143.6, 130.1, 124.4, 119.6, 51.9. **HRMS** (ESI): *m/z* calculated for $C_{16}H_{13}N_2O_4$ [M+H]$^+$ 297.0982; found 297.0978.

4-(1H-benzo[d][1,2,3]triazole-6-carboxamido)benzoic acid (**24c**)

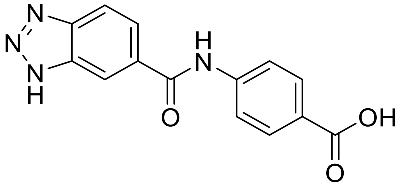

The methyl ester (**24b**, 622 mg, 2.10 mmol, 1.00 eq.) was dissolved in a mixture of THF/MeOH (1:1, 10 mL) and an aqueous solution of NaOH (5 M, 5 mL) solution was added. The reaction mixture was stirred for 3 h, after which the organic solvent was removed under reduced pressure by rotary evaporation. The residue was treated with an aqueous solution of HCl (6 M, 5 mL) and the precipitated product (**24c**, 593 g, 2.10 mmol, quant.) was filtrated and obtained as brownish solid.

^{1}H NMR (400 MHz, DMSO-d_6): δ [ppm] = 10.71 (s, 1 H), 8.67 (s, 1 H), 8.00 – 7.99 (m, 2 H), 7.96 (br. s, 4 H). **^{13}C NMR** (101 MHz, DMSO-d_6): δ [ppm] = 165.8, 165.6, 143.6, 130.1, 124.4, 119.6, 51.9. **HRMS** (ESI): *m/z* calculated for $C_{16}H_{13}N_2O_4$ [M+H]$^+$ 283.0826; found 283.0819.

Albicidin derivative (**24**)

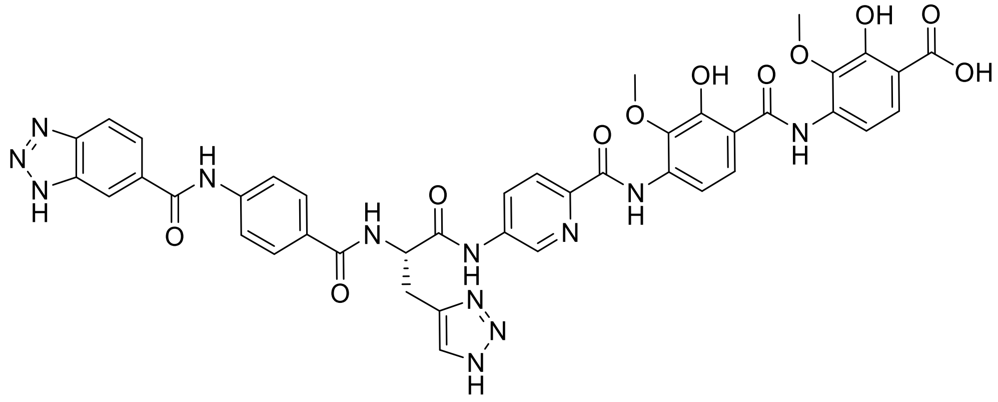

The synthesis of the product was conducted according to coupling protocol A. The albicidin derivative **24** (6 mg, 7% over two steps) was obtained as a colourless solid.

^{1}H NMR (DMSO-d_6, 700 MHz): δ [ppm] = 11.73 (s, 1 H), 11.60 (s, 1 H), 11.14 (s, 1 H), 10.86 (d, J = 7.2 Hz, 2 H), 10.66 (s, 1 H), 10.50 (s, 1 H), 9.00 (d, J = 2.3 Hz, 1 H), 8.83 (d, J = 7.4 Hz, 1 H), 8.70 (b, 1 H), 8.35 (dd, J = 8.6, 2.4 Hz, 1 H), 8.22 (d, J = 8.6 Hz, 1 H), 8.12 (d, J = 9.0 Hz, 1 H), 8.08 – 8.01 (m, 3 H), 7.94 (s, 4 H), 7.89 (d, J = 8.9 Hz, 1 H), 7.72 (s, 1 H), 7.60 (d, J = 8.8 Hz, 1 H), 4.96 (dd, J = 7.5 Hz, 1 H), 3.93 (s, 3 H), 3.88 (s, 3 H), 3.35 (dd, J = 14.7, 5.7 Hz, 1 H), 3.28 (dd, J = 14.8, 9.2 Hz, 1 H). **^{13}C NMR** from HSQC-ed (DMSO-d_6, 176 MHz): δ [ppm] = 139.5, 128.8, 127.8, 127.1, 126.2, 123.4, 119.9, 110.9, 110.7, 61.3, 60.7, 54.8, 27.7 ppm. **HRMS** (ESI): m/z calculated for $C_{41}H_{35}N_{12}O_{11}$ $[M+H]^+$ 871.2621; found 871.2535, t_R = 7.16 min.

Spectra

Albicidin derivative 4

^{1}H-NMR 700 MHz

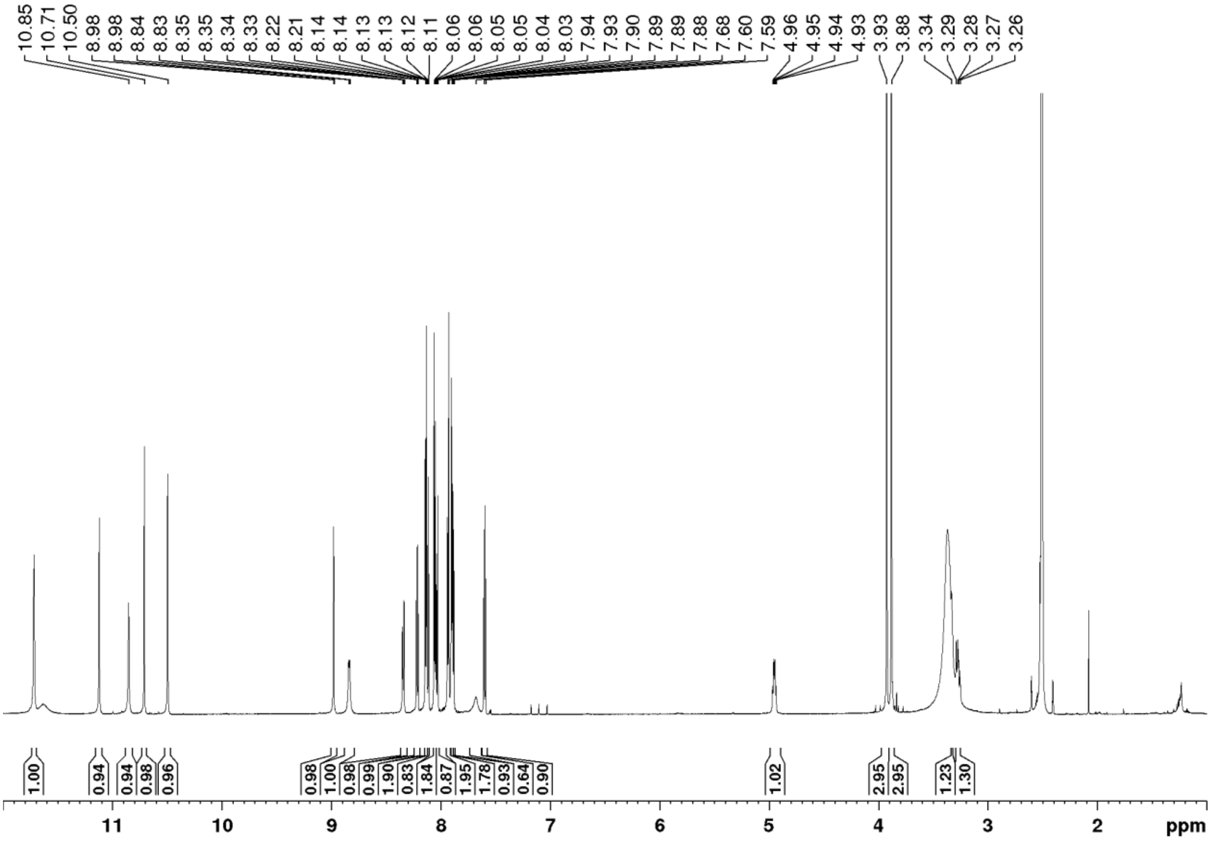

H,H-COSY 700 MHz

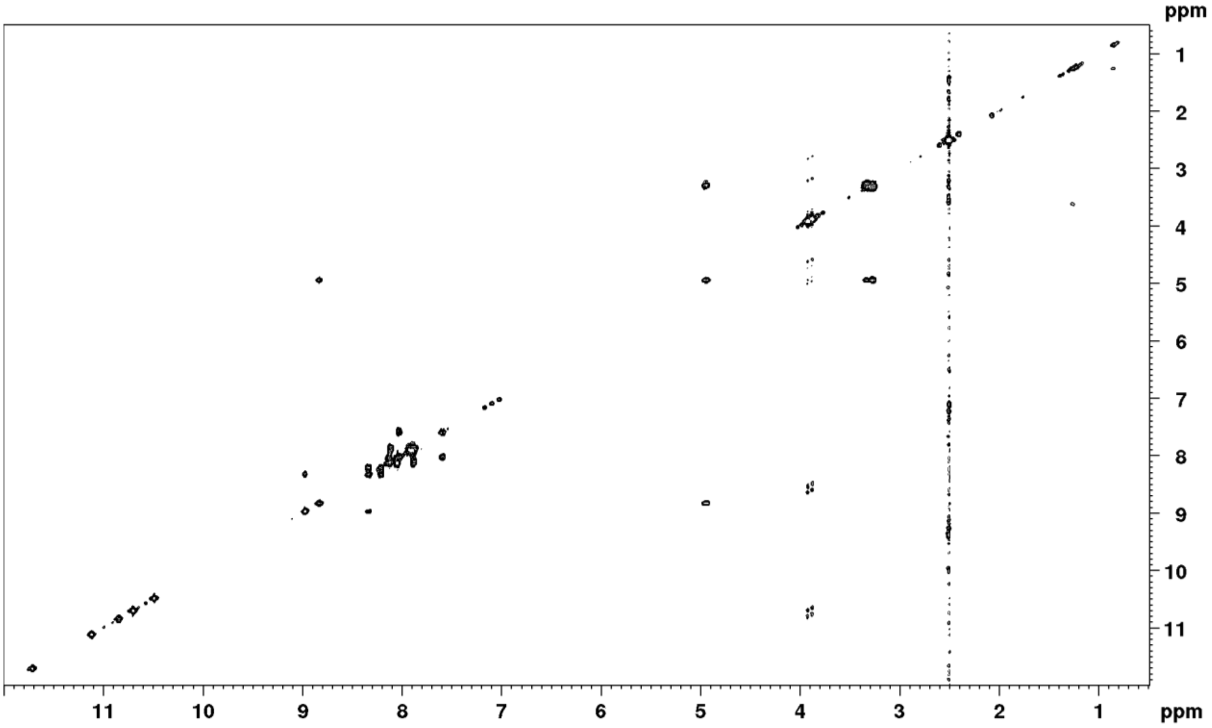

H,C-HSQC – 700 MHz

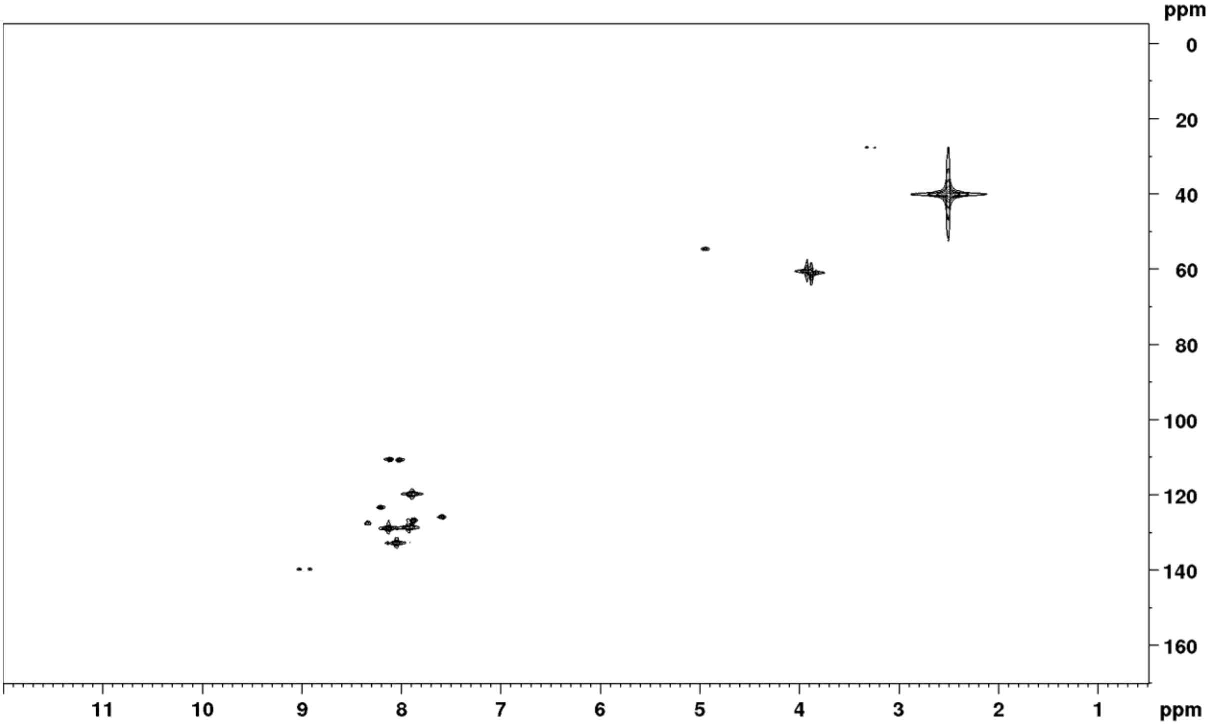

Albicidin derivative 5

^{1}H-NMR 700 MHz

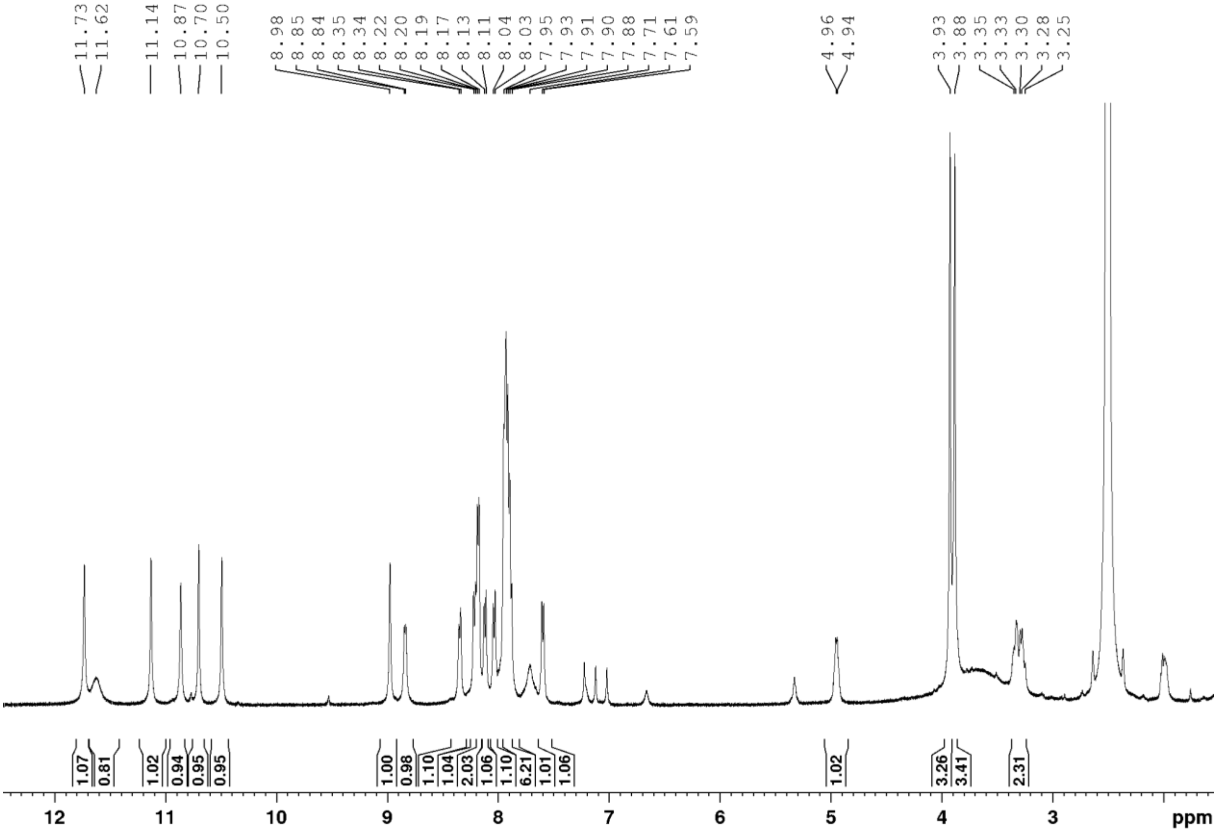

H,H-COSY 700 MHz

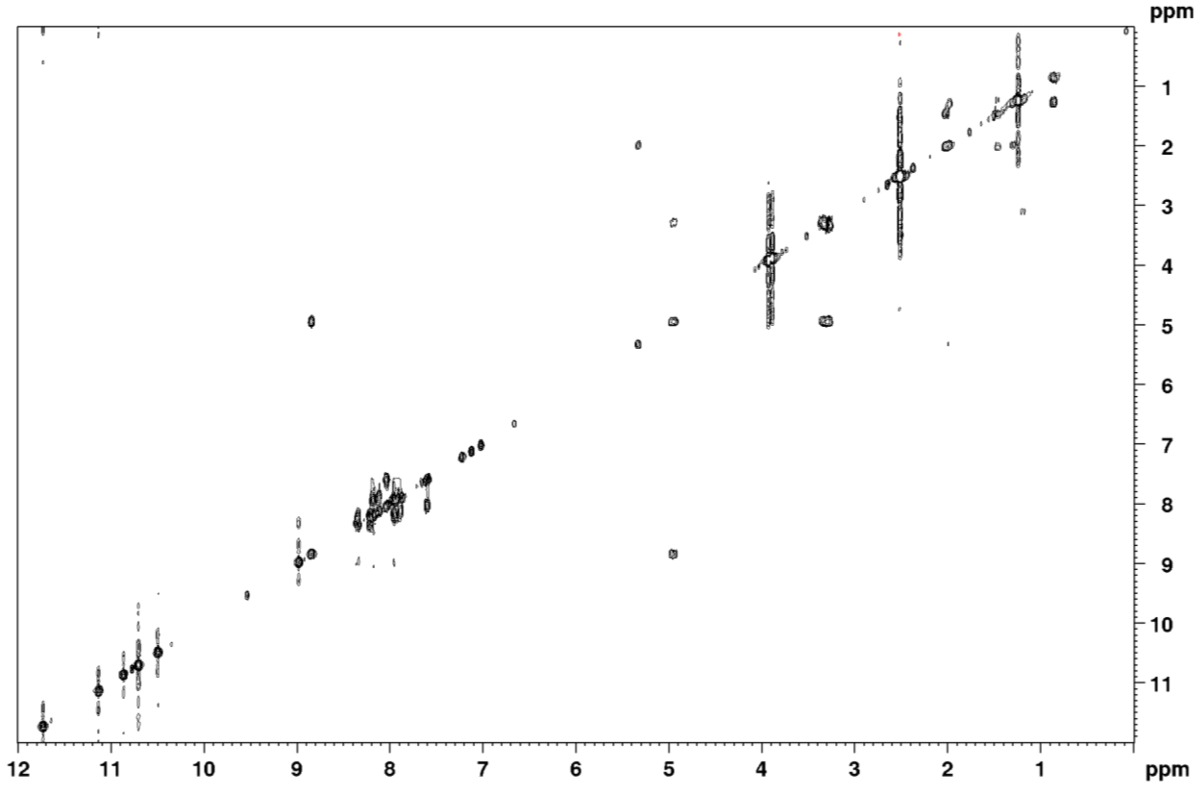

H,C-HMQC – 700 MHz

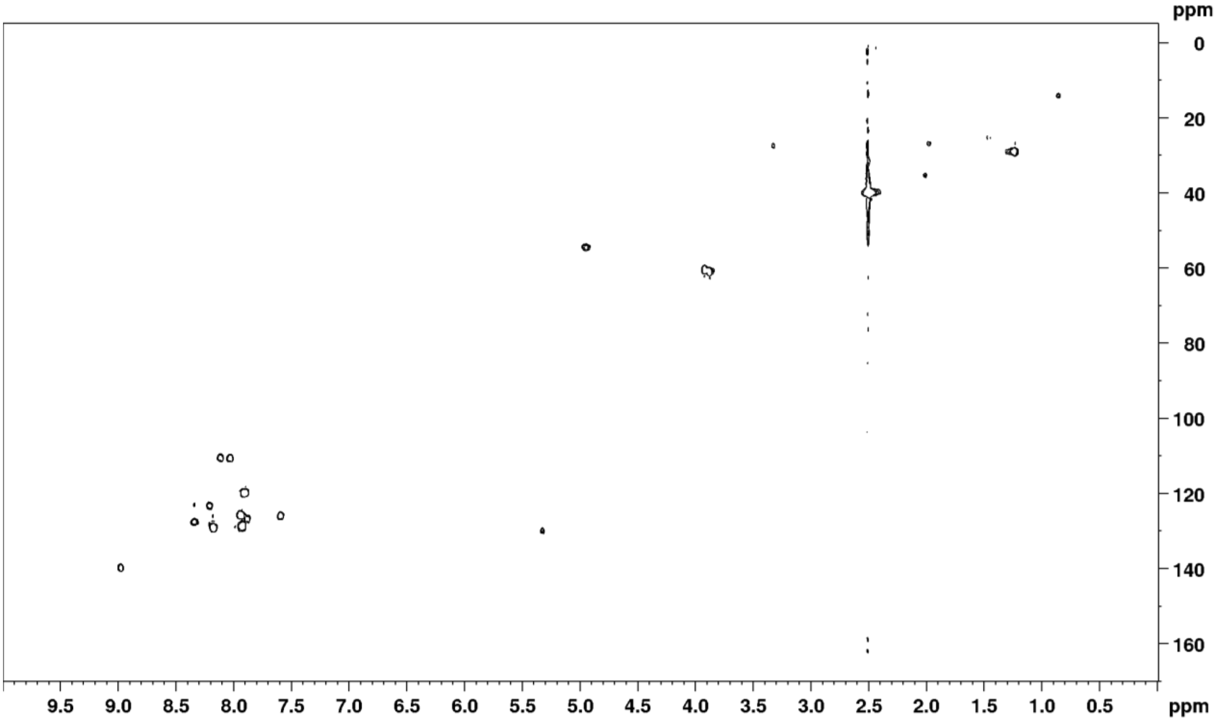

H,C-HMBC – 700 MHz

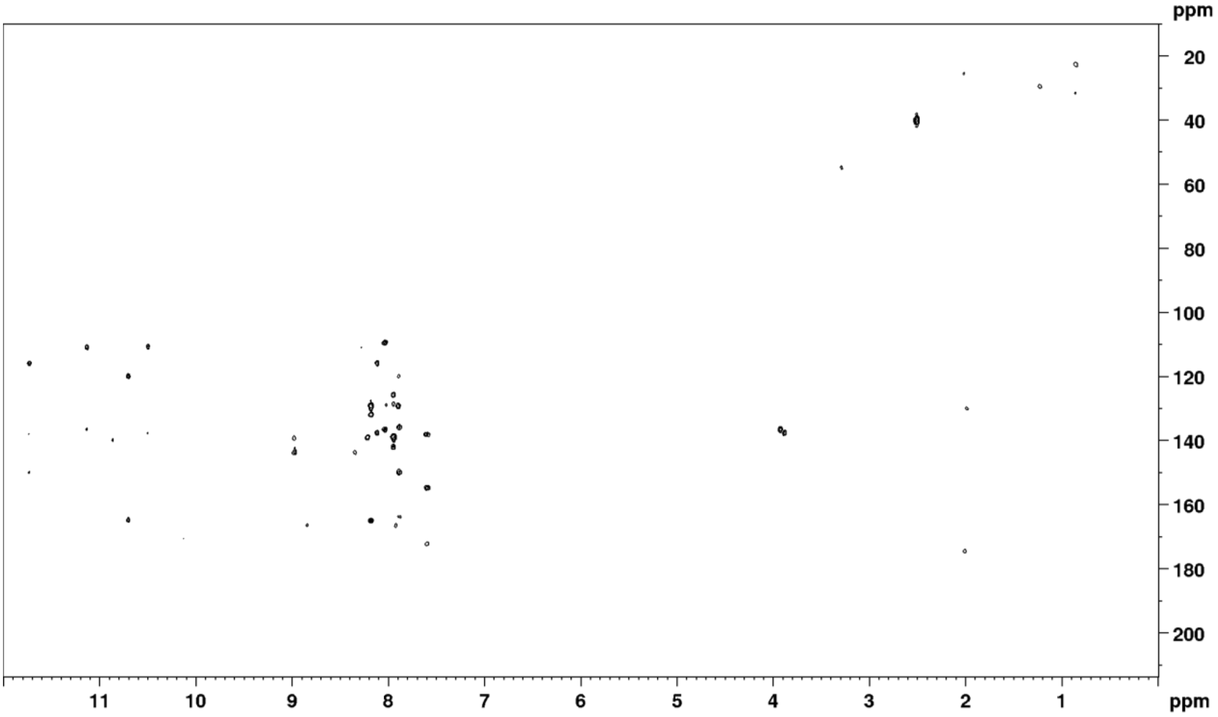

Albicidin derivative 6

^{1}H-NMR 700 MHz

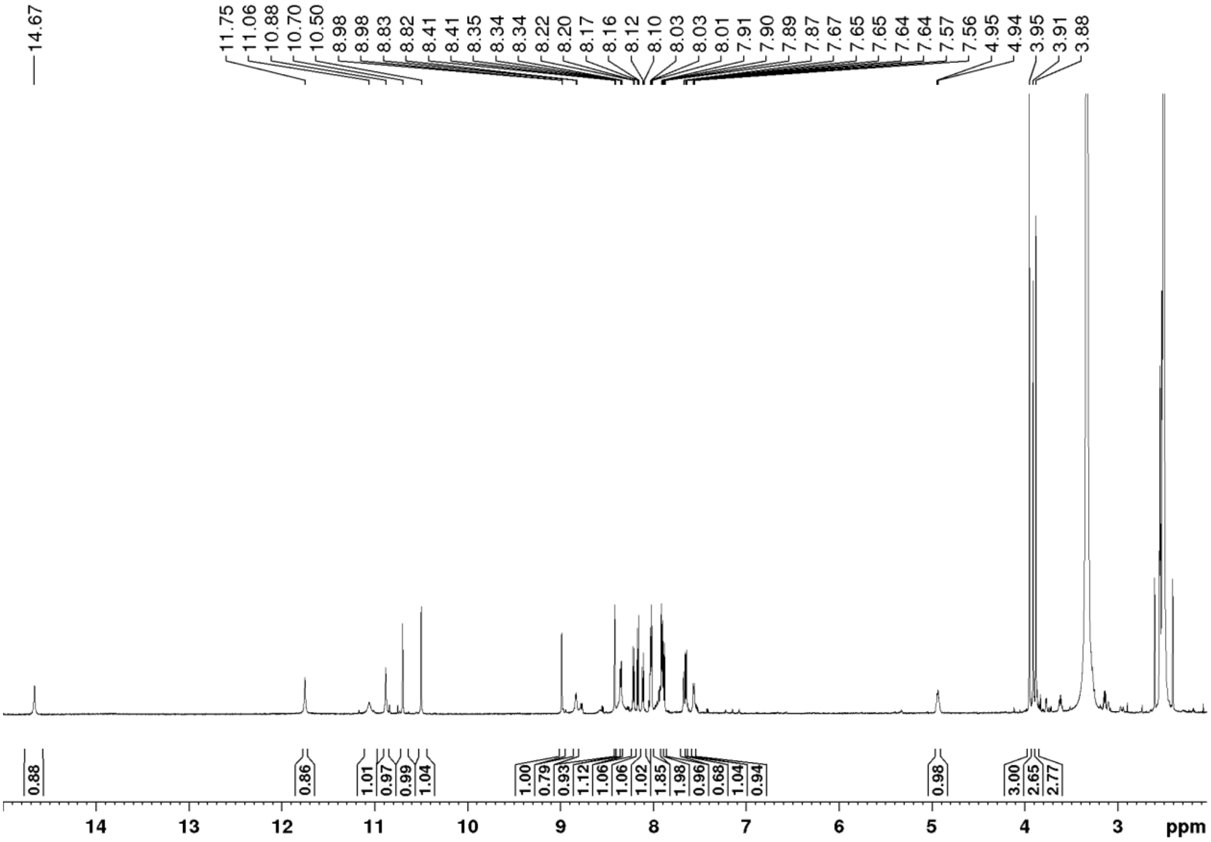

H,H-COSY 700 MHz

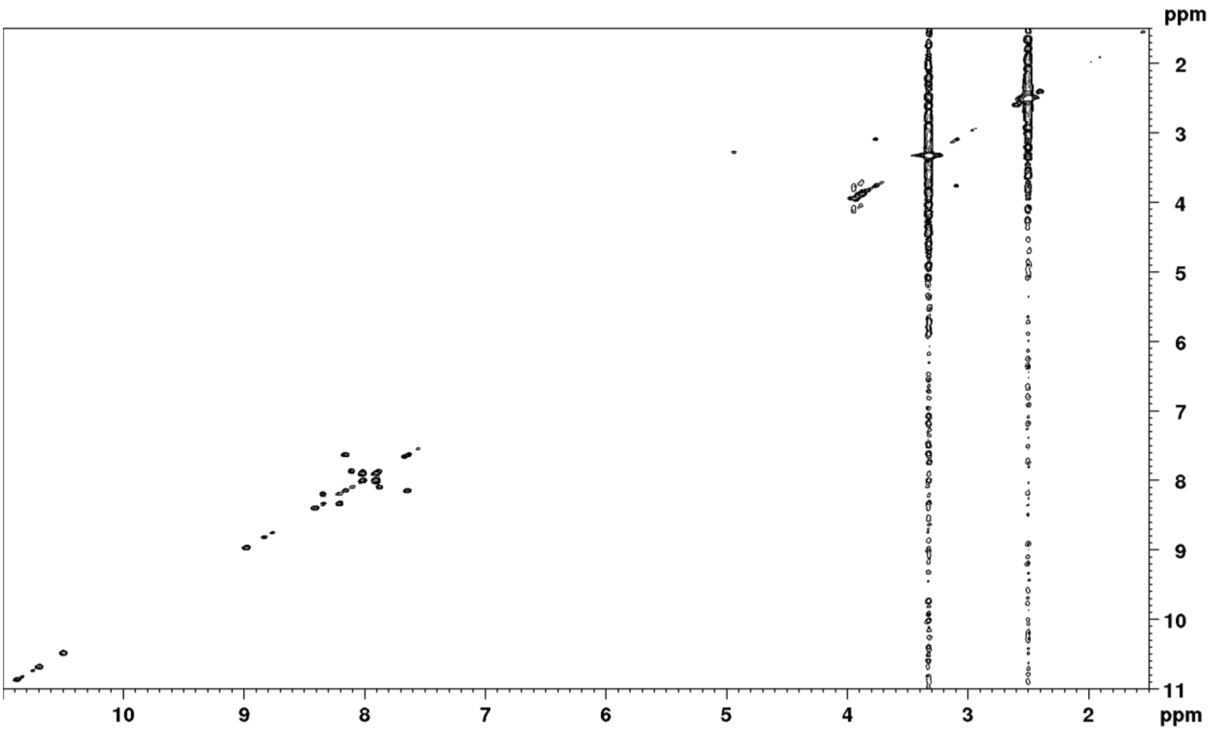

H,C-HSQC – 700 MHz

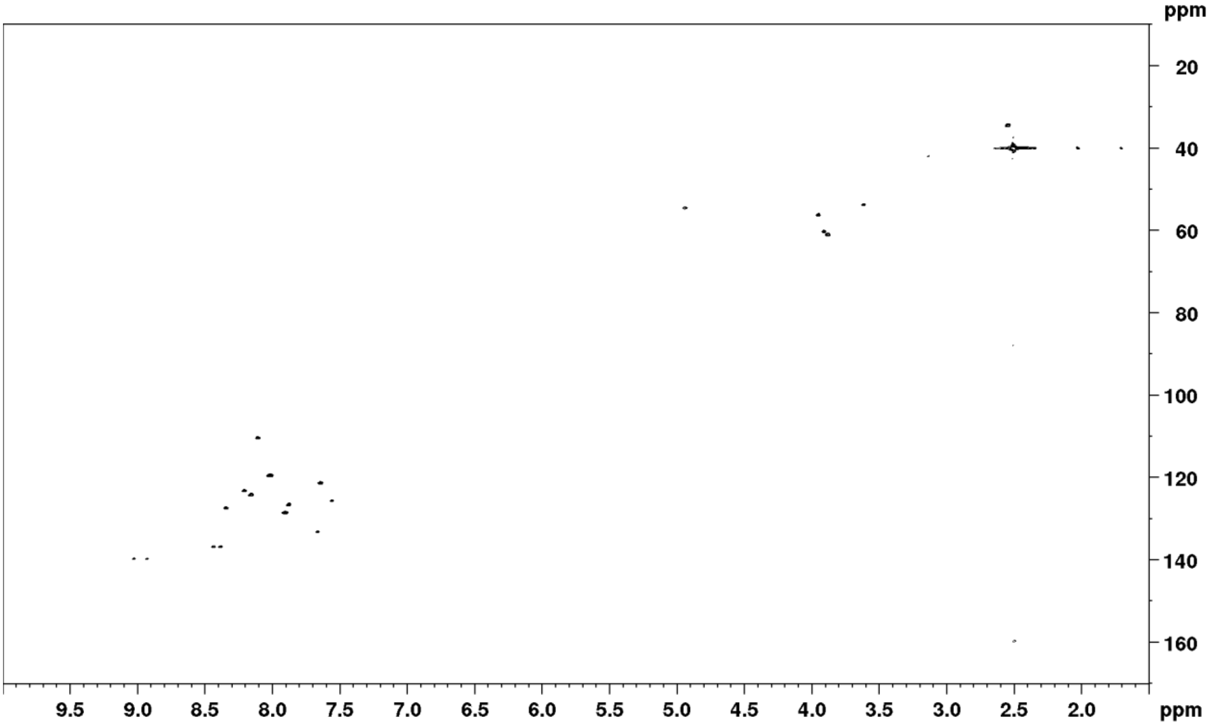

Albicidin derivative 7

^{1}H-NMR 700 MHz

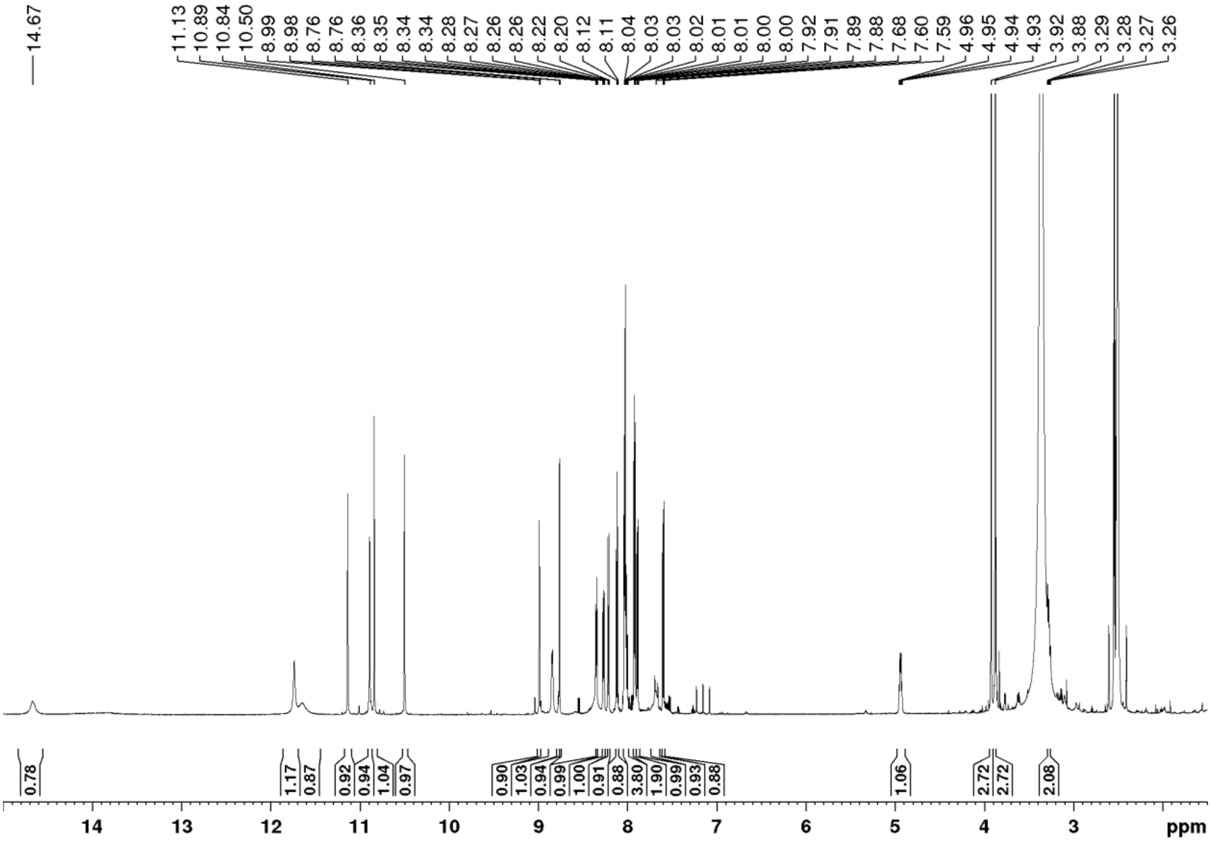

H,H-COSY 700 MHz

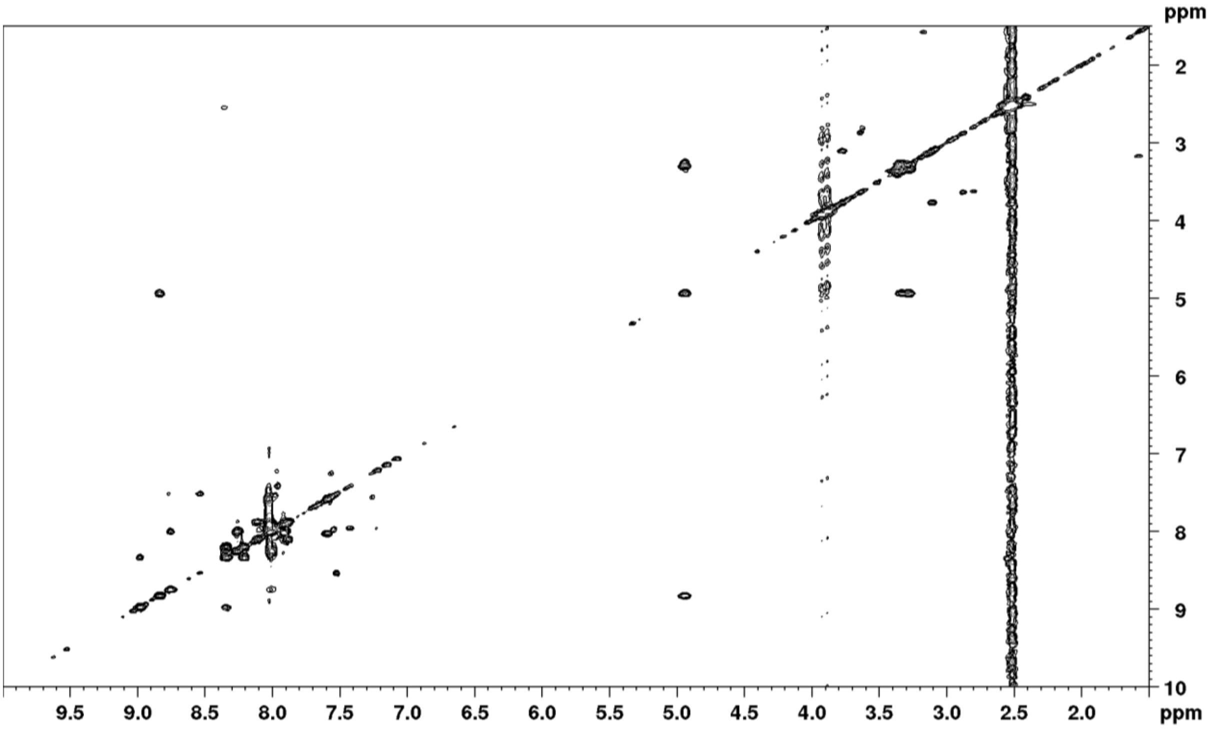

H,C-HSQC – 700 MHz

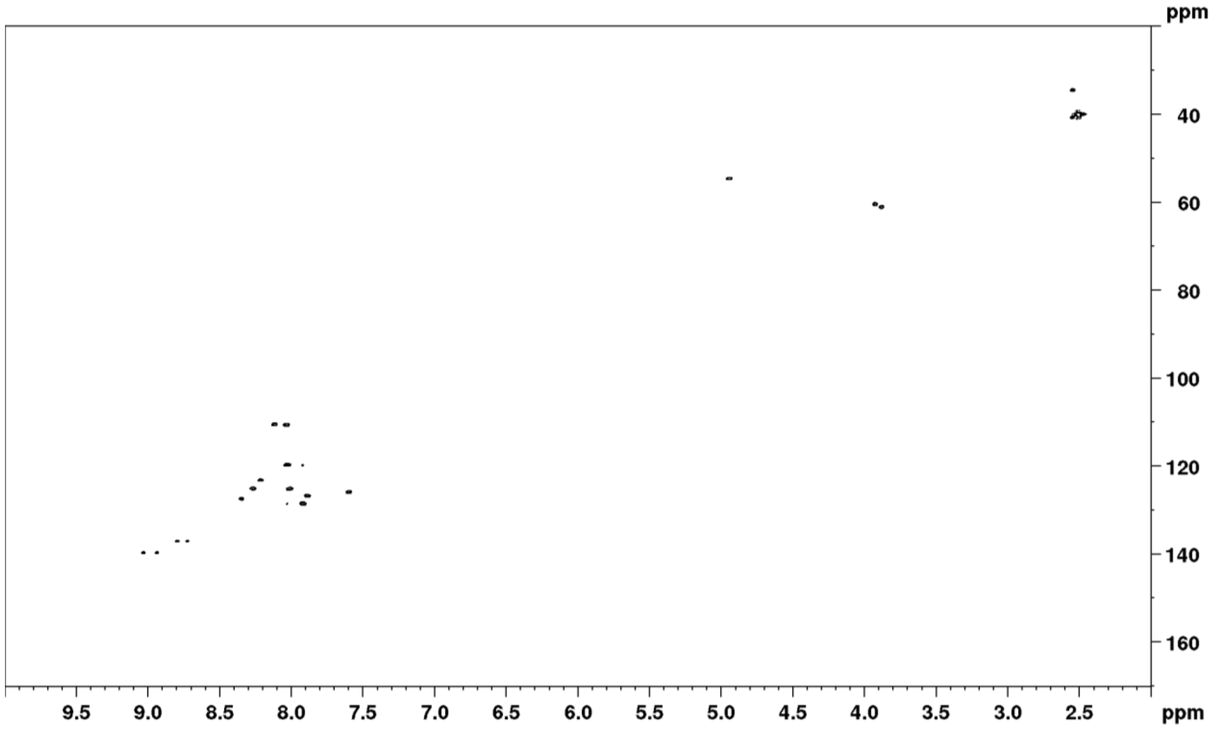
ppm
40
60
80
100
120
140
160
9.5
9.0
8.5
8.0
7.5
7.0
6.5
6.0
5.5
5.0
4.5
4.0
3.5
3.0
2.5
ppm

Albicidin derivative 8

^{1}H-NMR 700 MHz

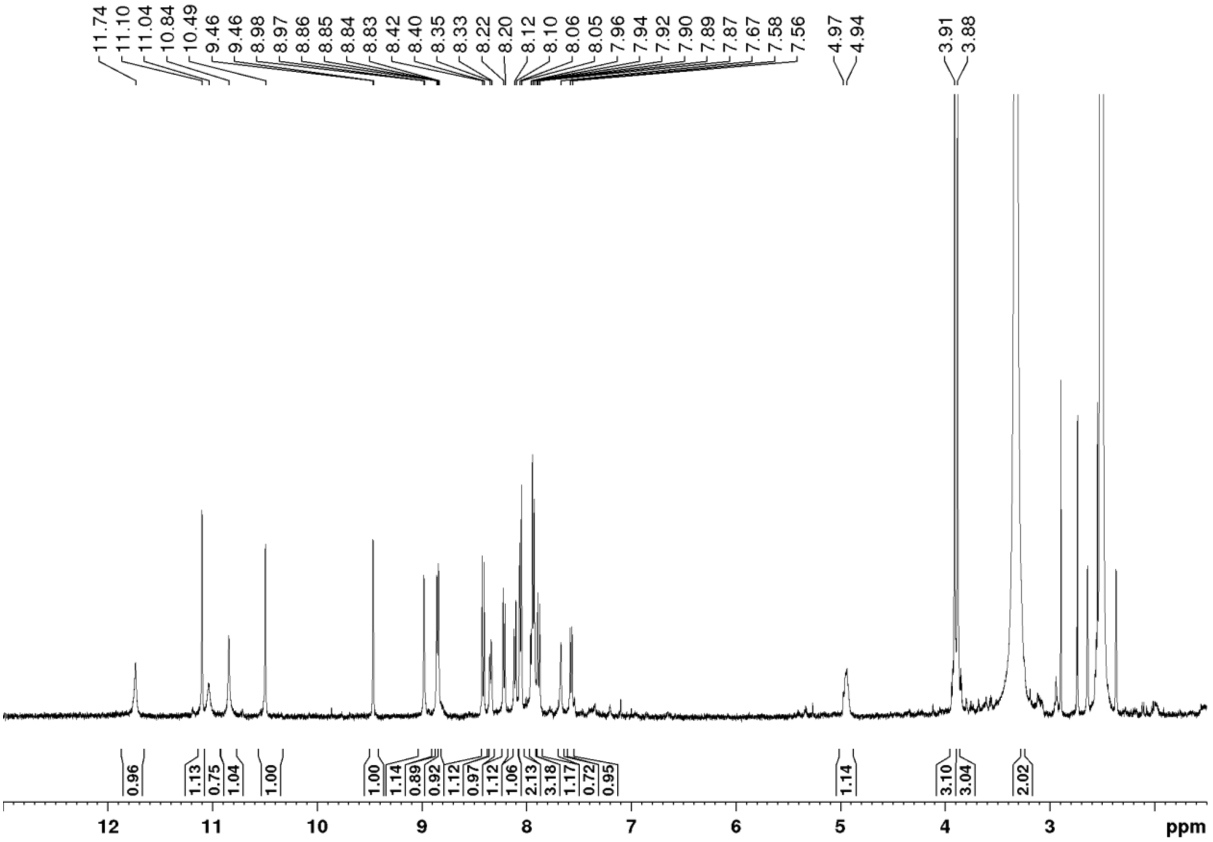

H,H-COSY 700 MHz

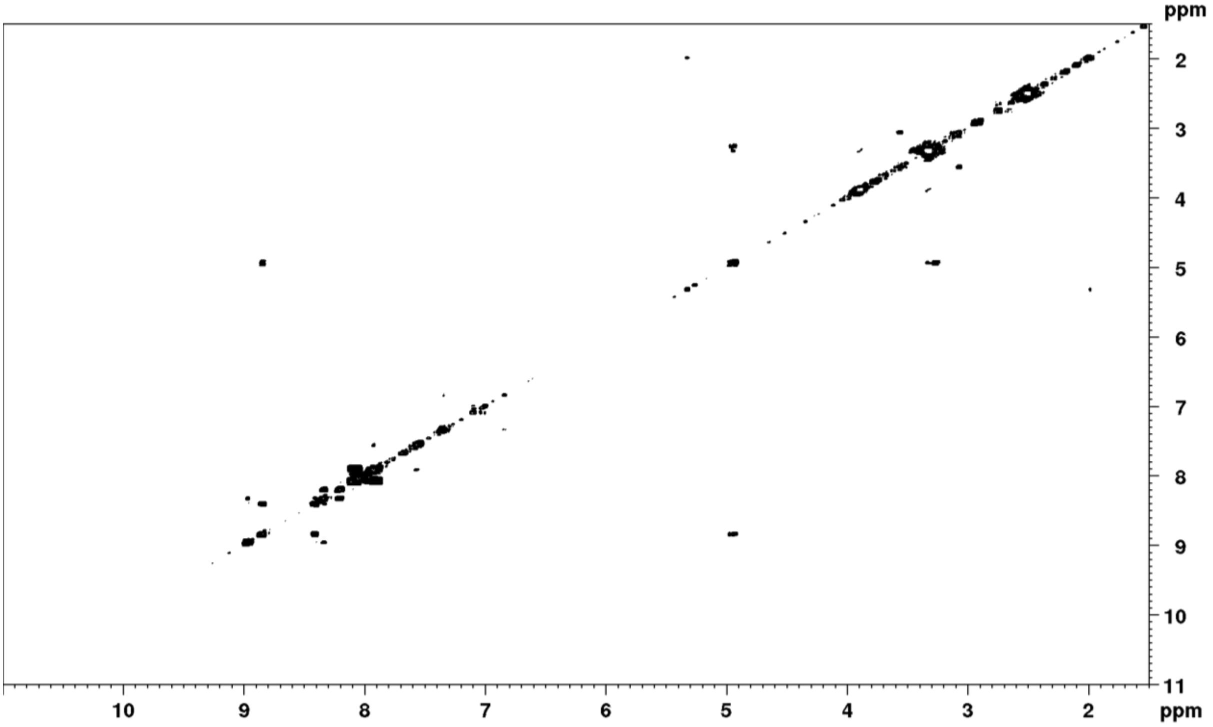

H,C-HSQC – 700 MHz

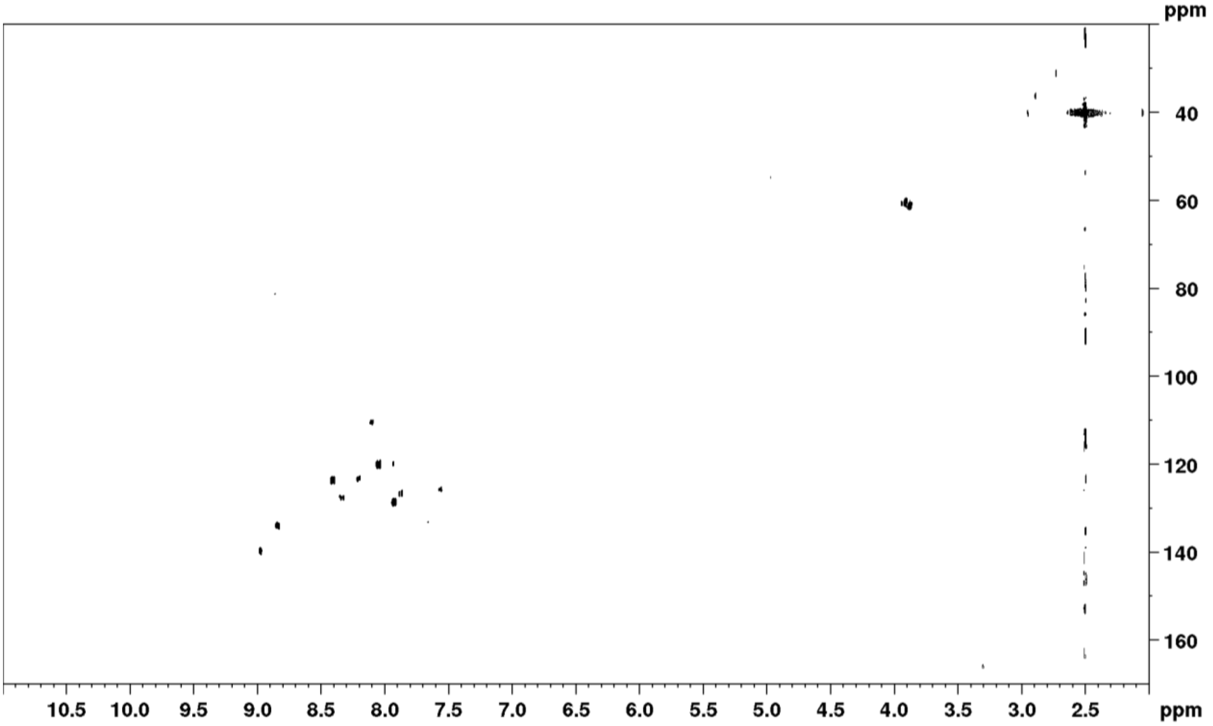

Albicidin derivative 9

^{1}H-NMR 700 MHz

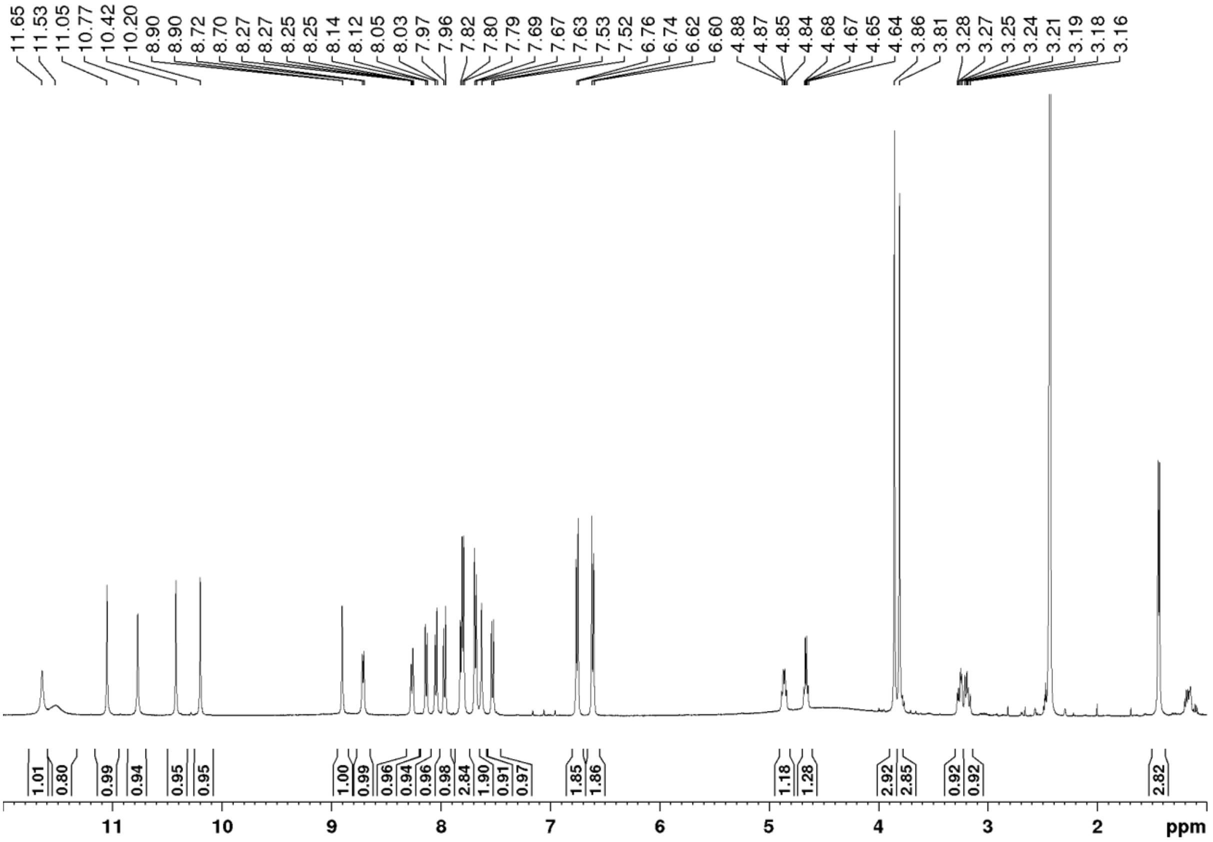

H,H-COSY 700 MHz

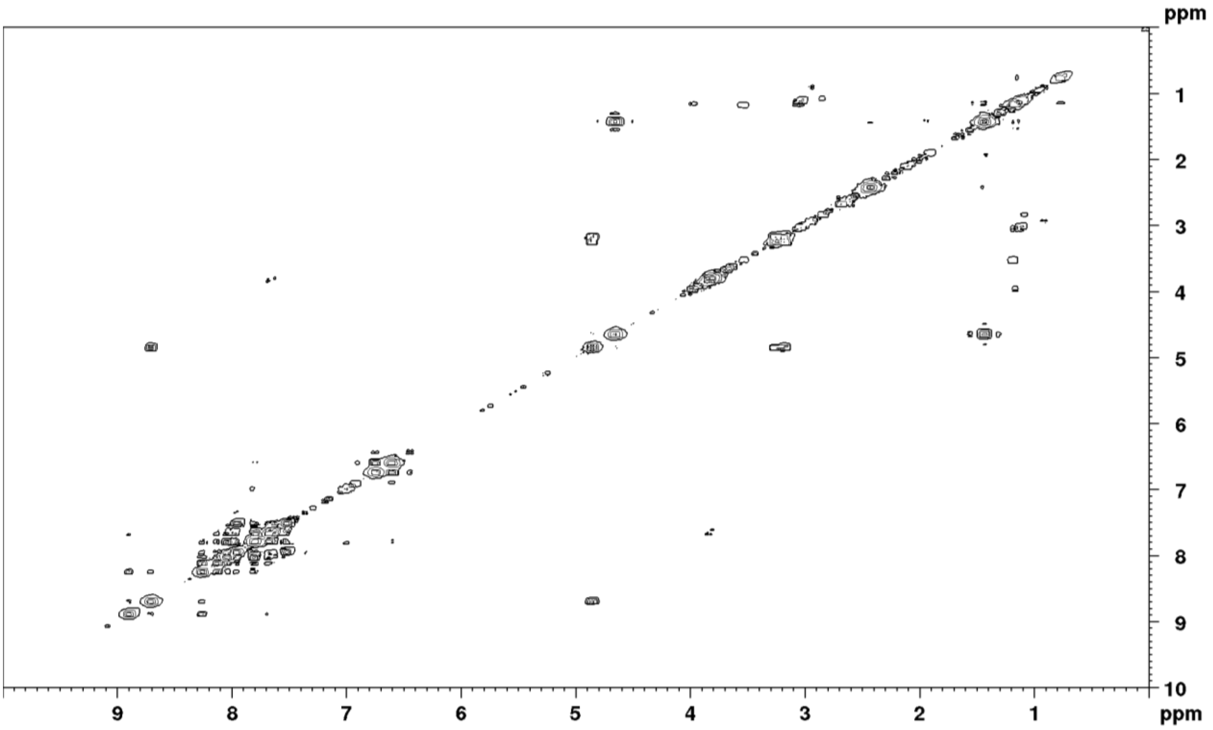

H,C-HSQC – 700 MHz

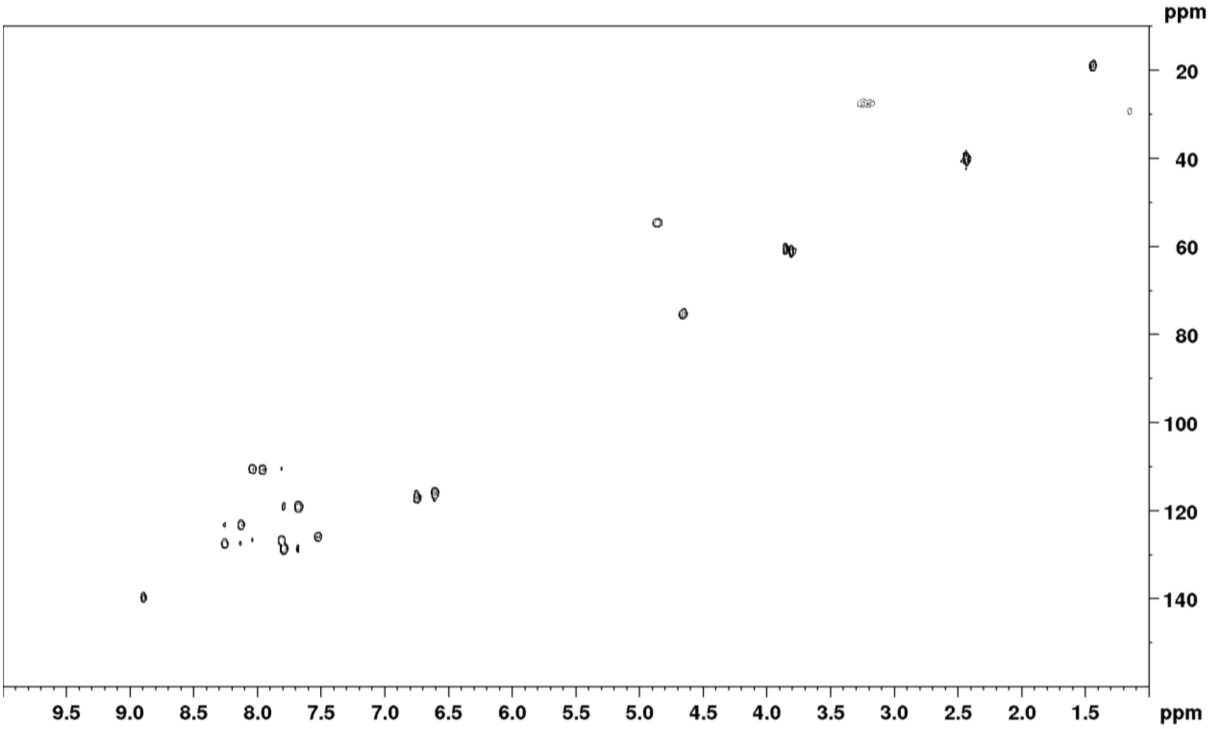

Albicidin derivative 10

^{1}H-NMR 700 MHz

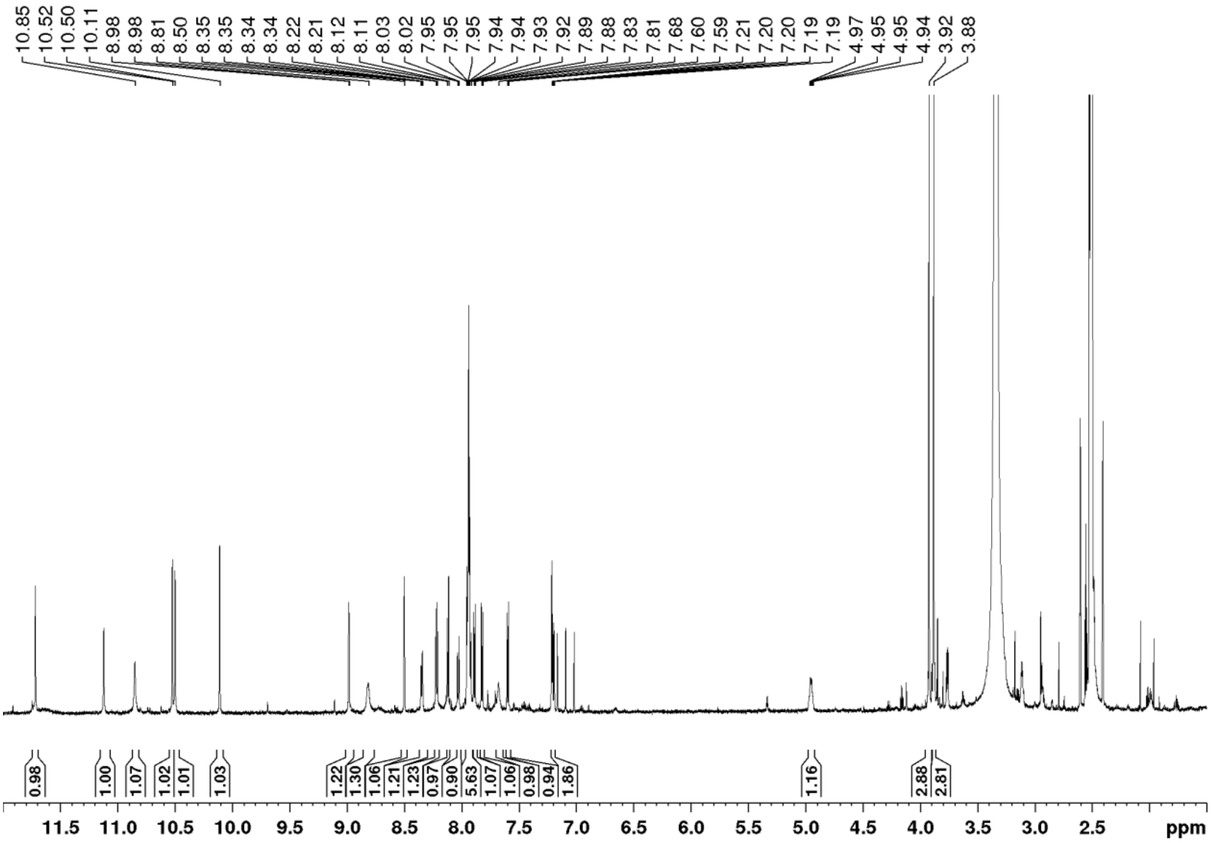

H,H-COSY 700 MHz

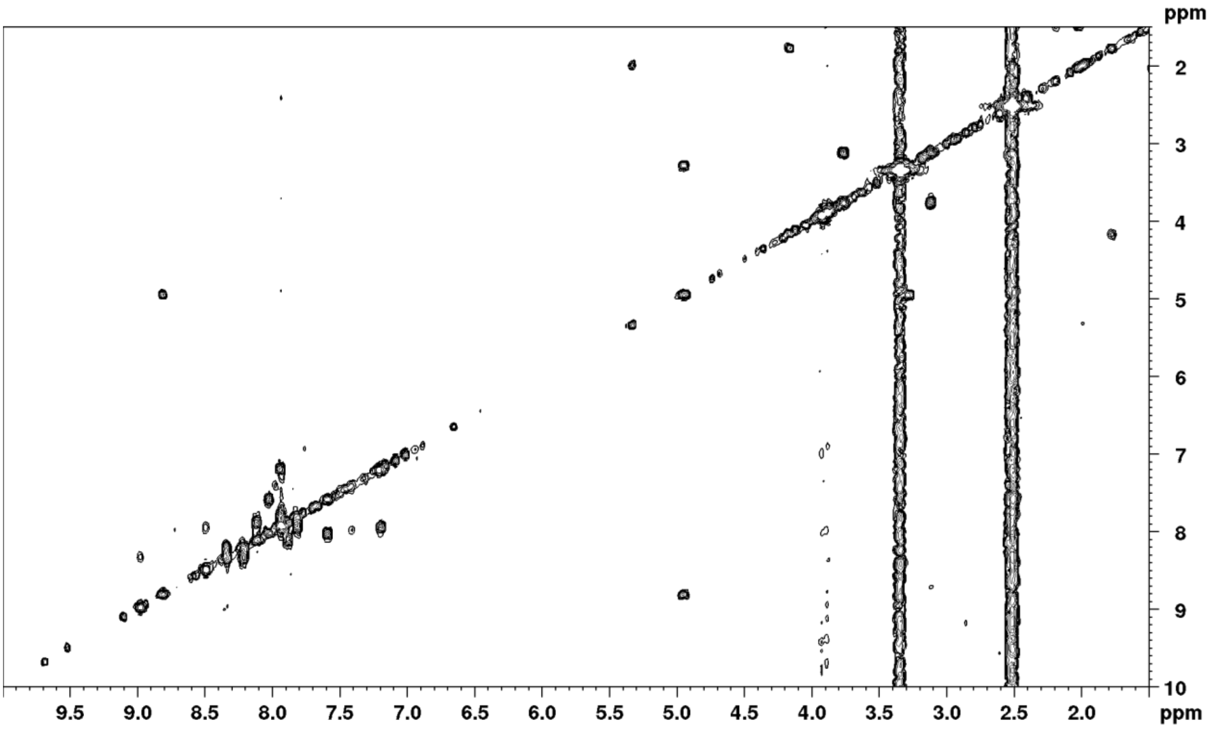

H,C-HSQC – 700 MHz

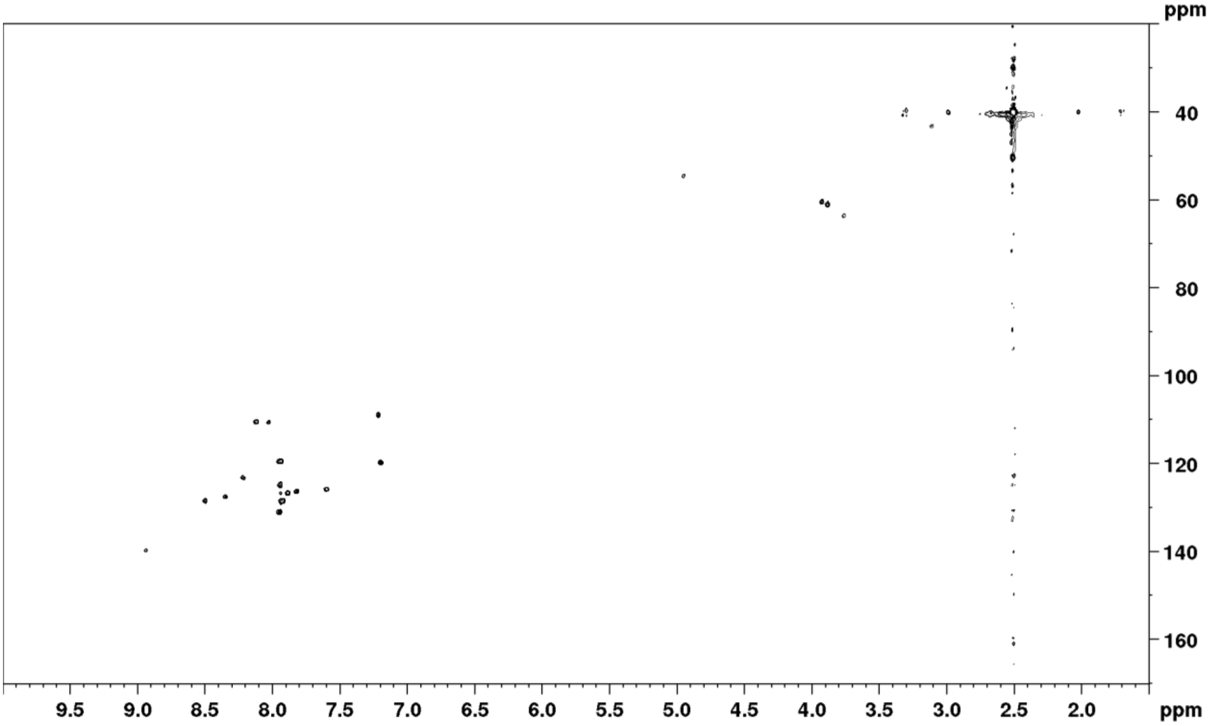

Albicidin derivative 11

^{1}H-NMR 700 MHz

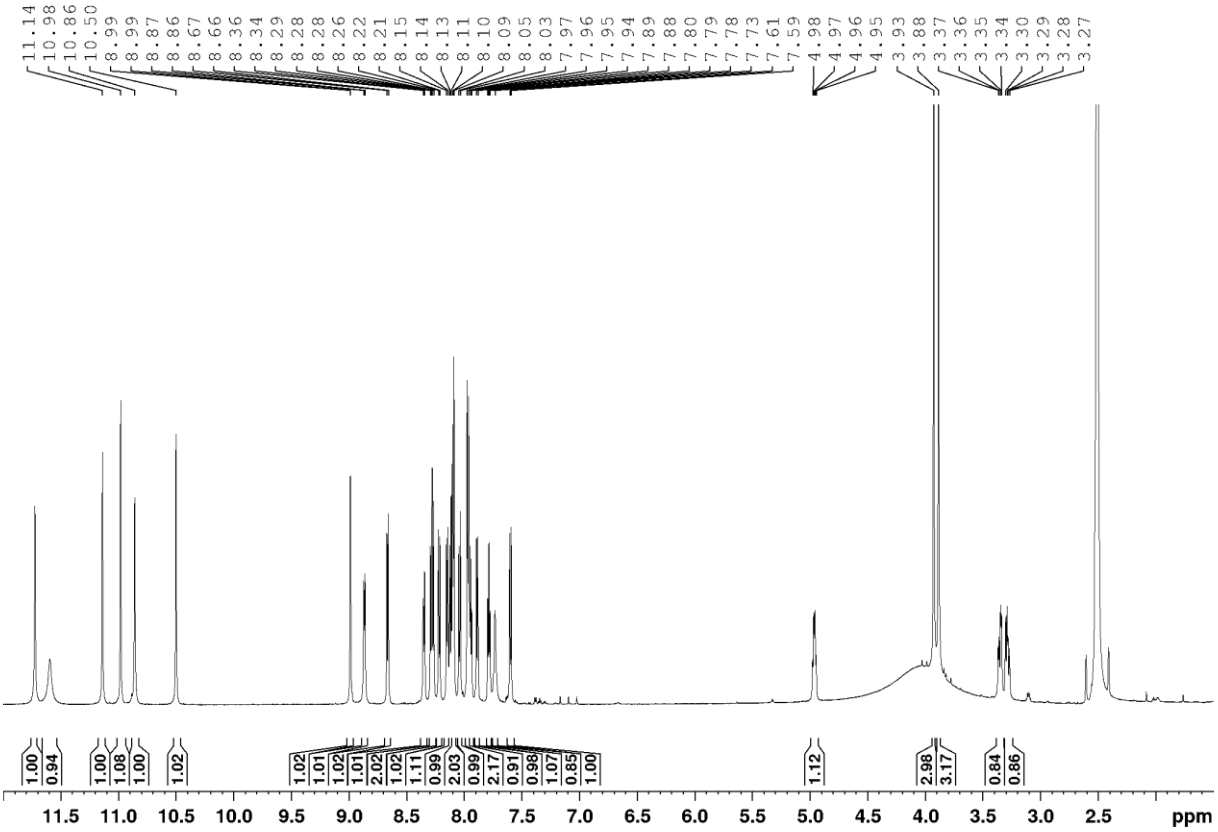

H,H-COSY 700 MHz

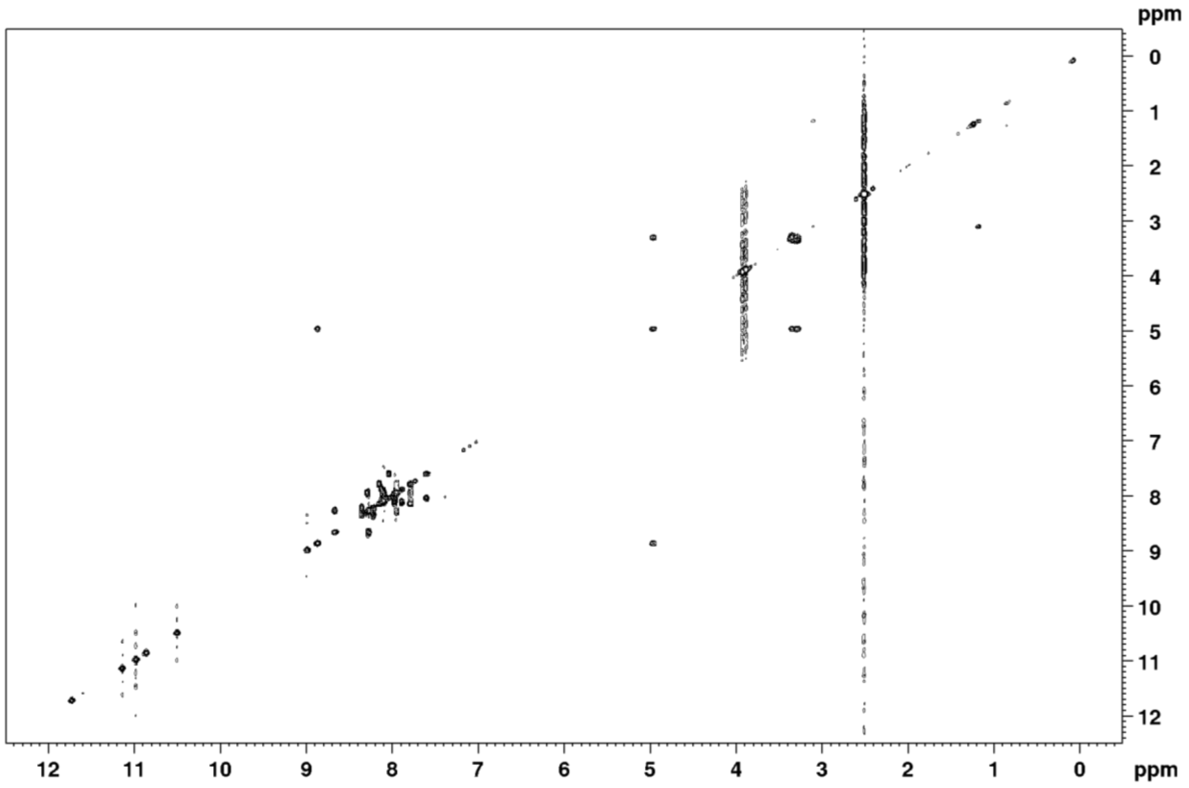

H,C-HSQC – multiplicity edited – 700 MHz

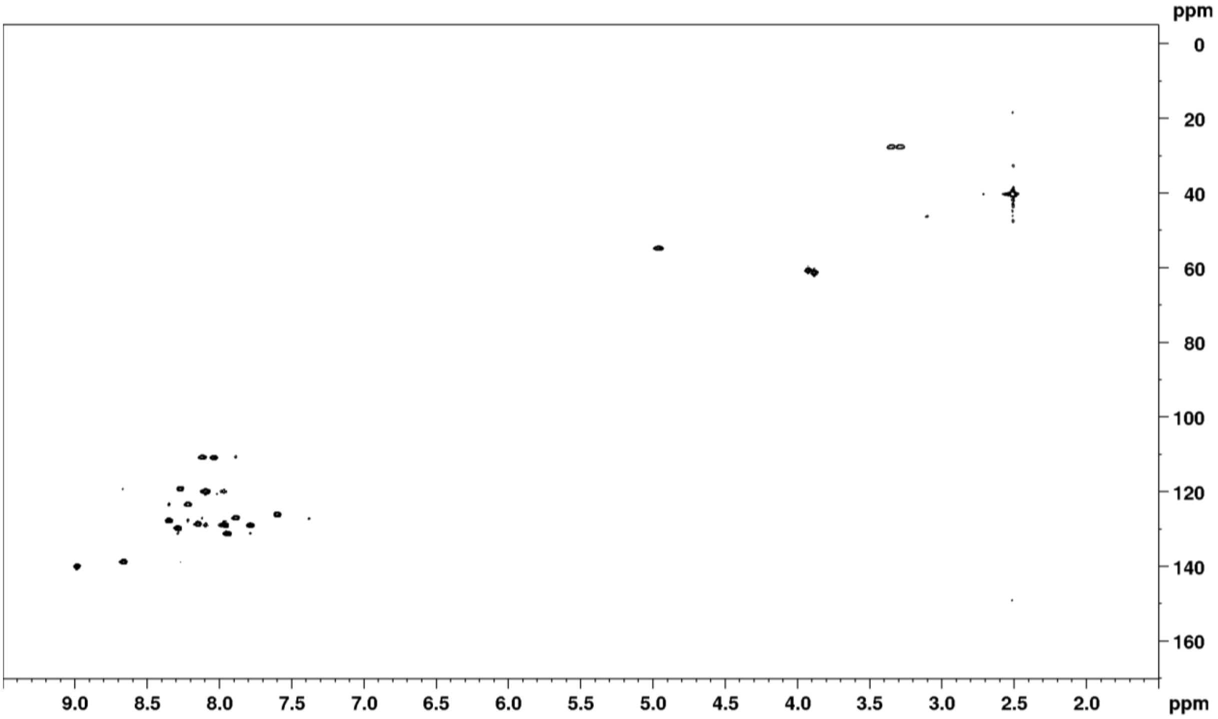

H,C-HMBC – 700 MHz

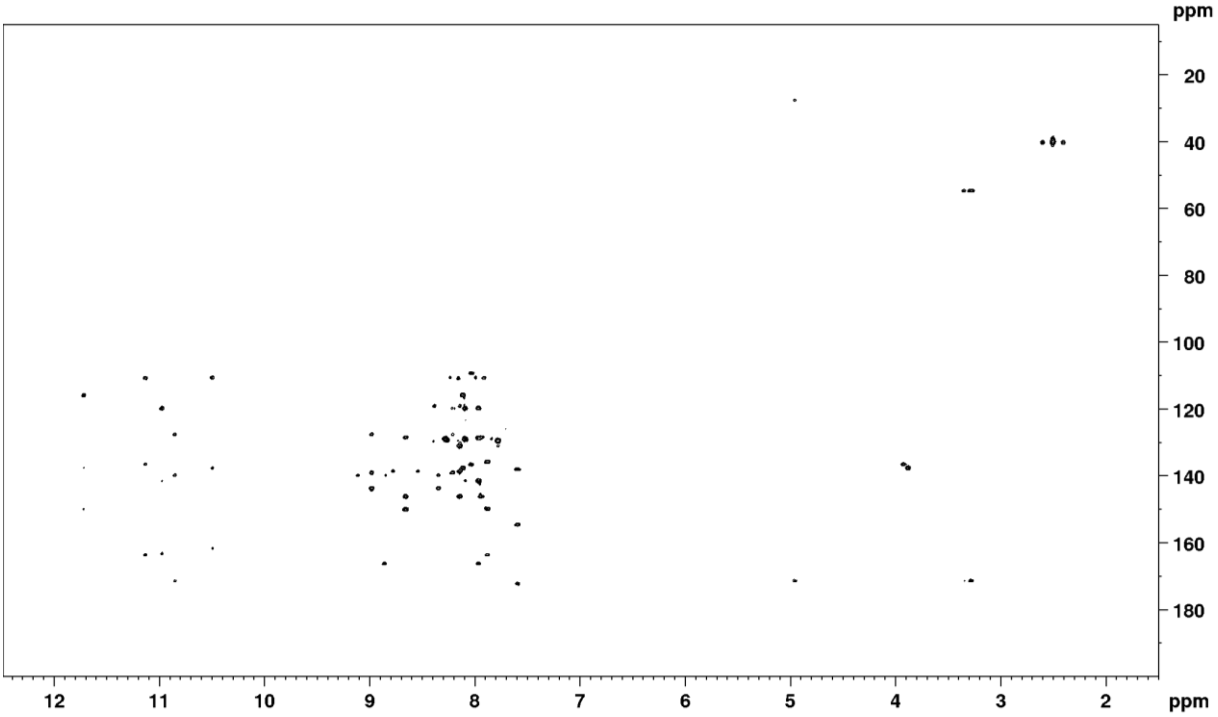

Albicidin derivative 12

^{1}H-NMR 700 MHz

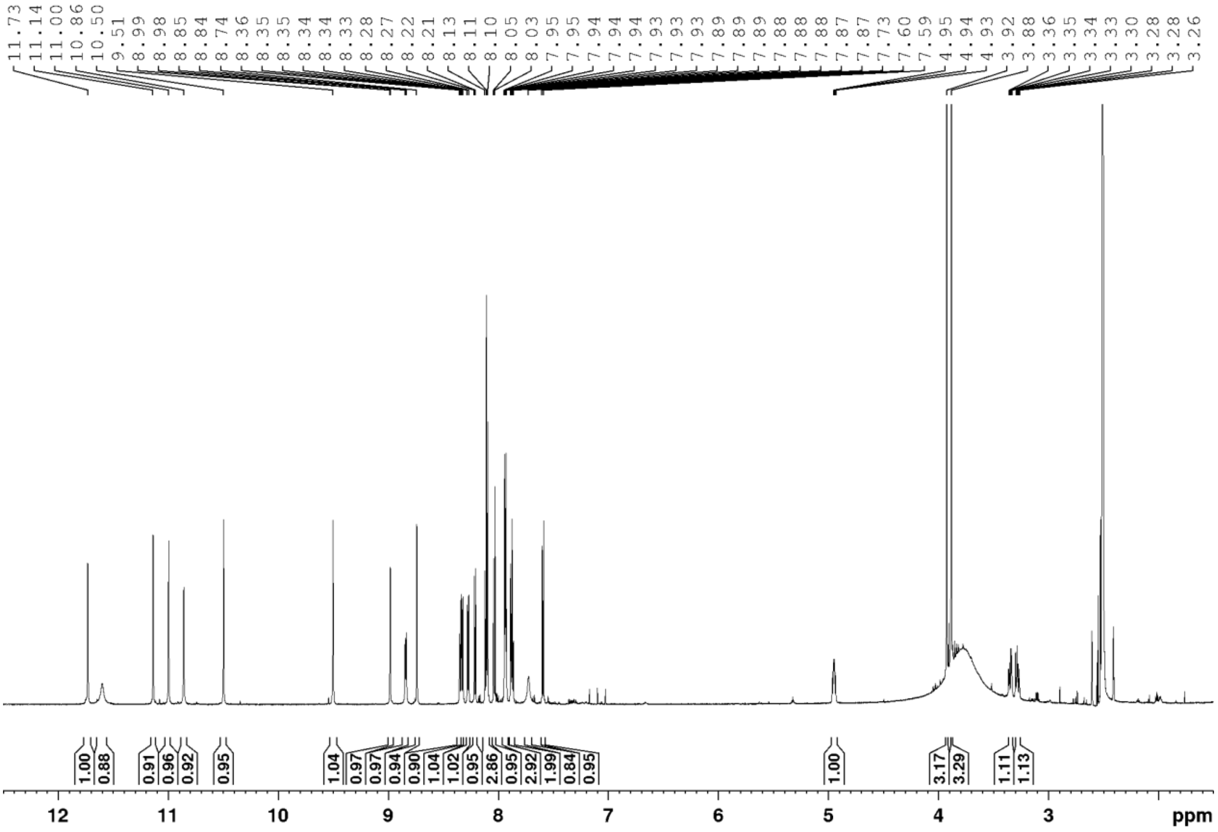

H,H-COSY 700 MHz

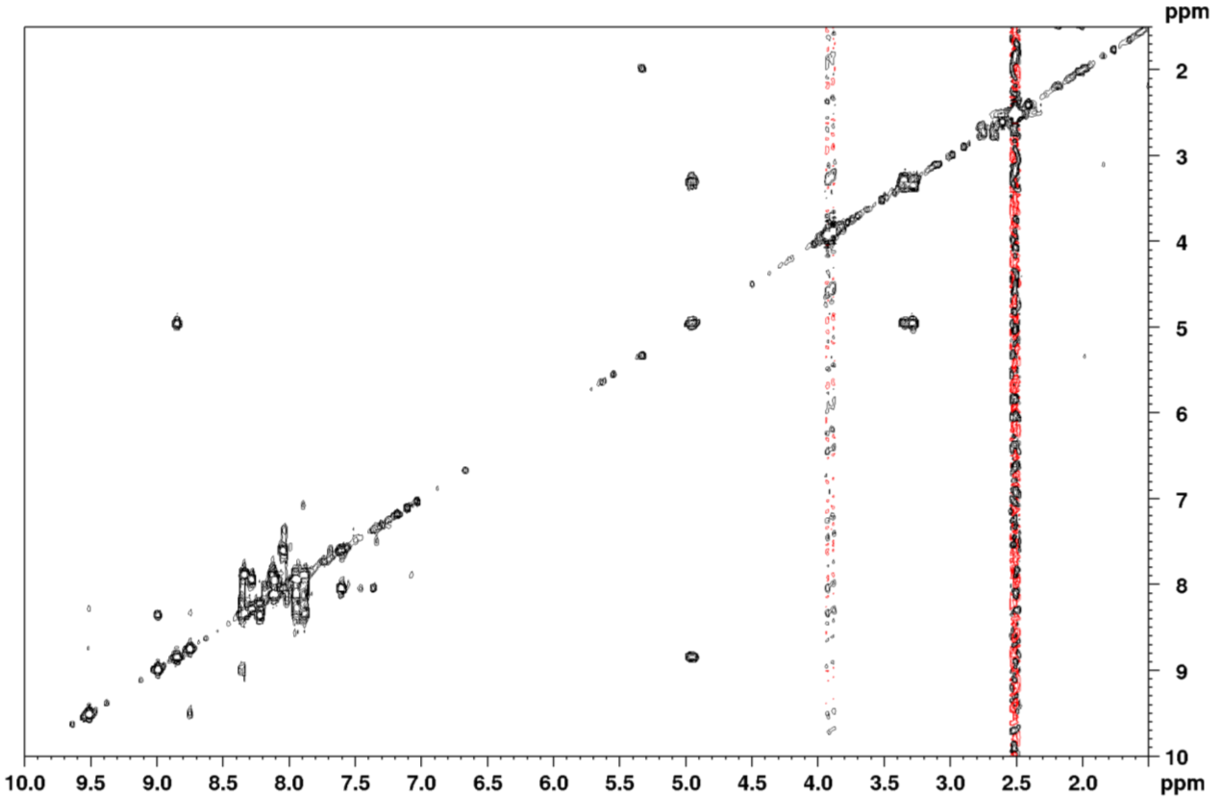

H,C-HMQC – 700 MHz

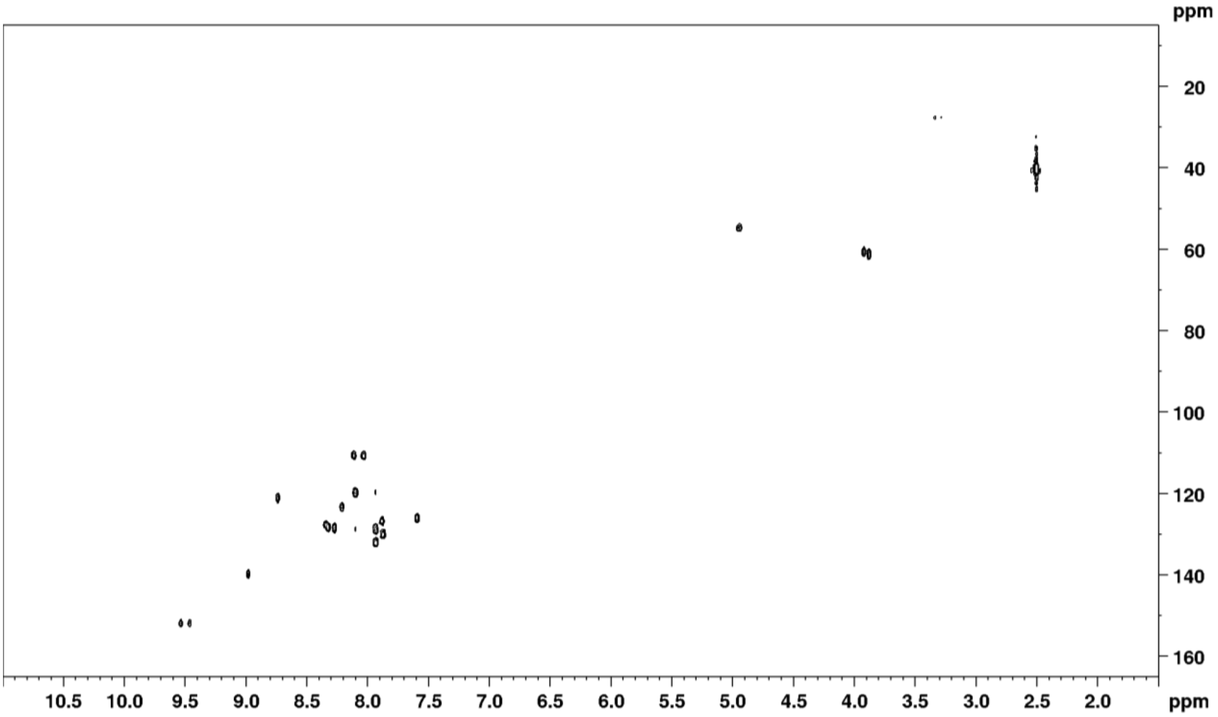

H,C-HMBC – 700 MHz

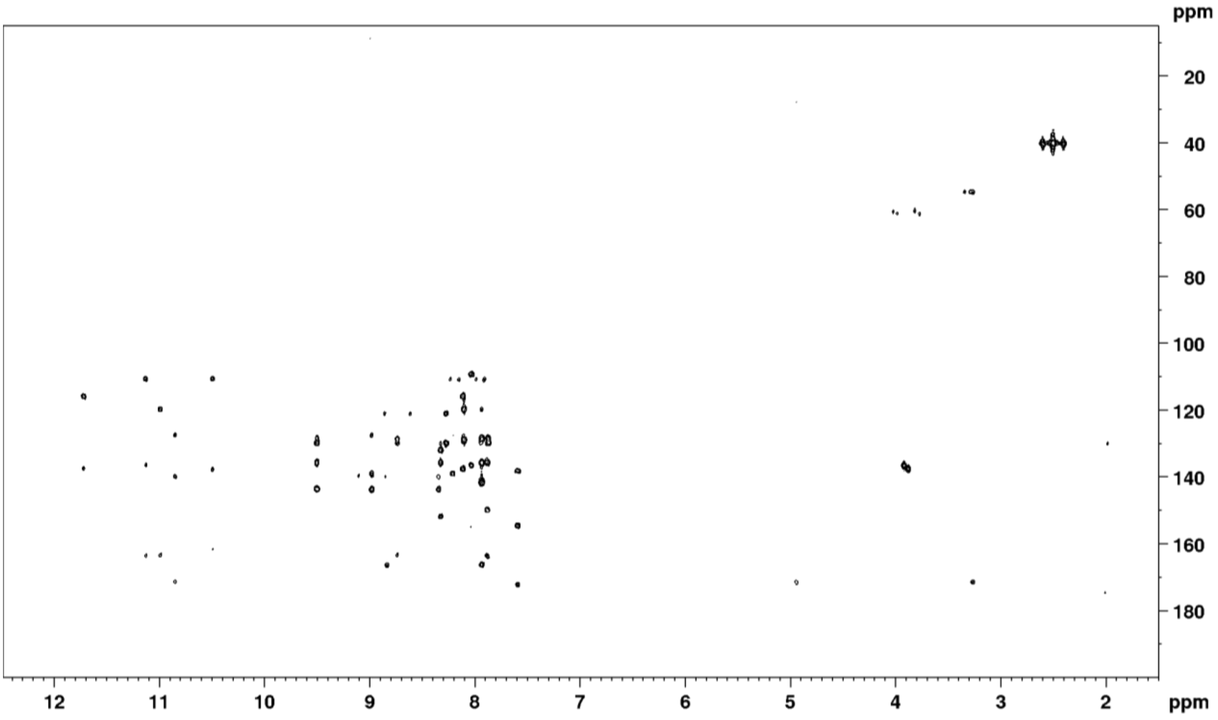

Albicidin derivative 13

^{1}H-NMR 700 MHz

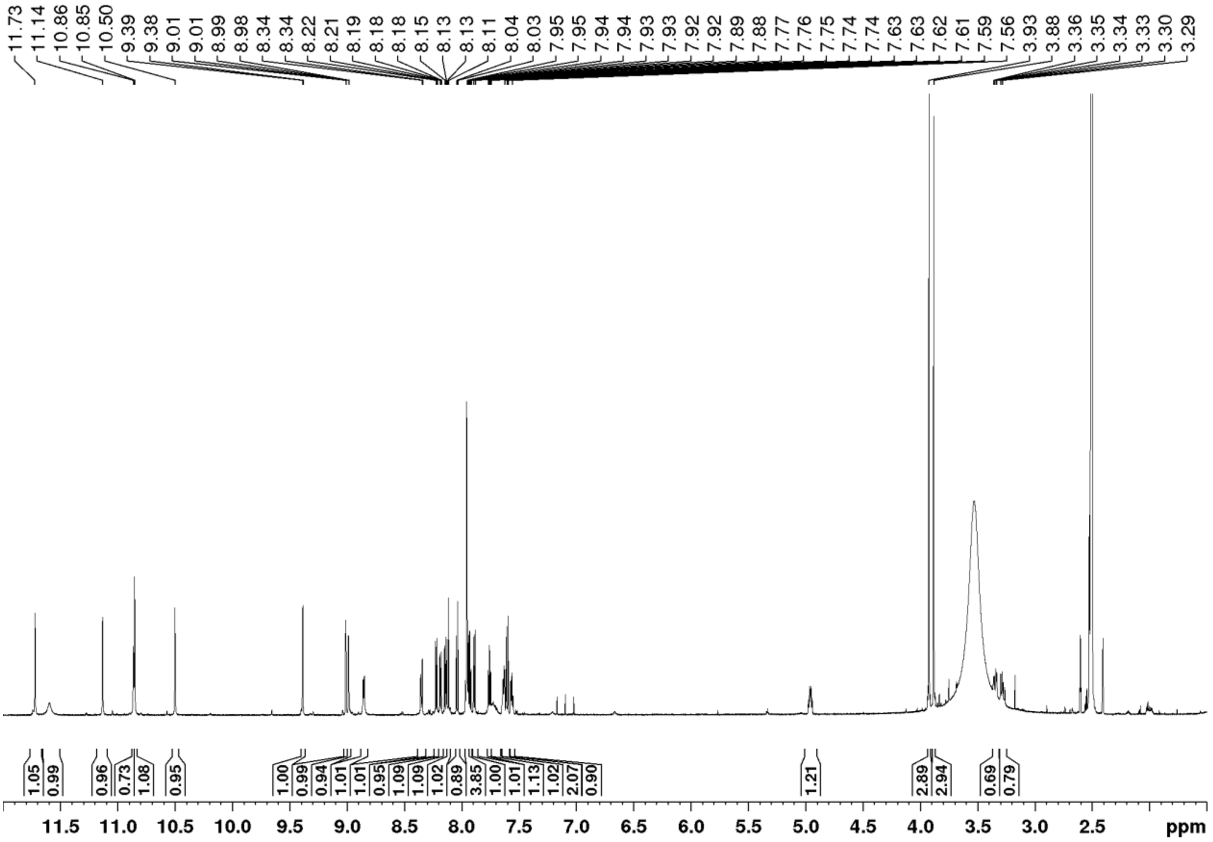

H,H-COSY 700 MHz

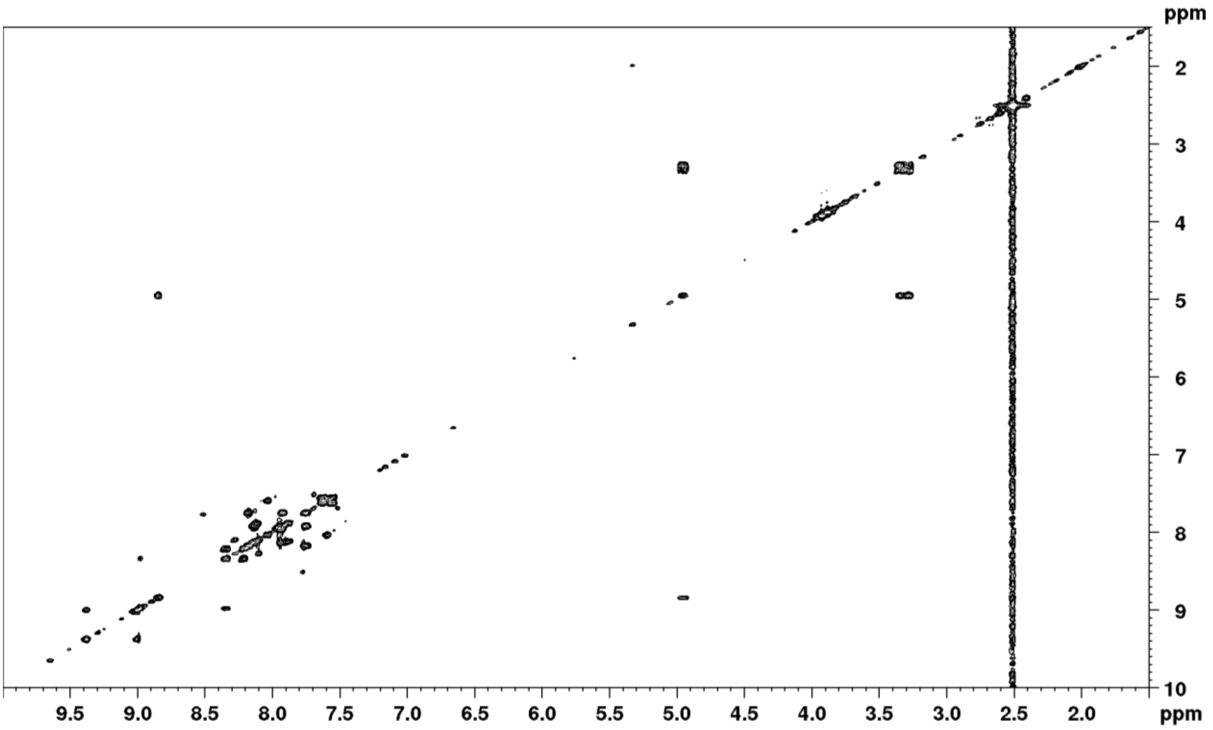

H,C-HSQC – 700 MHz

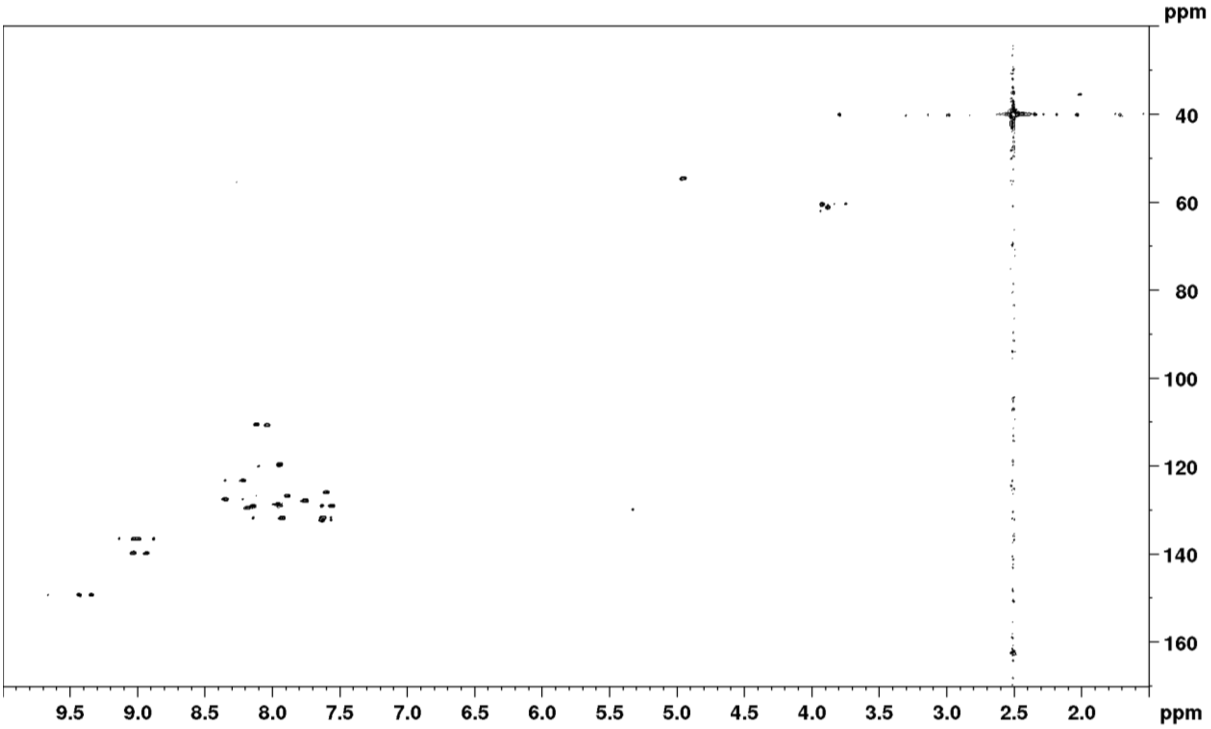

Albicidin derivative 14

^{1}H-NMR 700 MHz

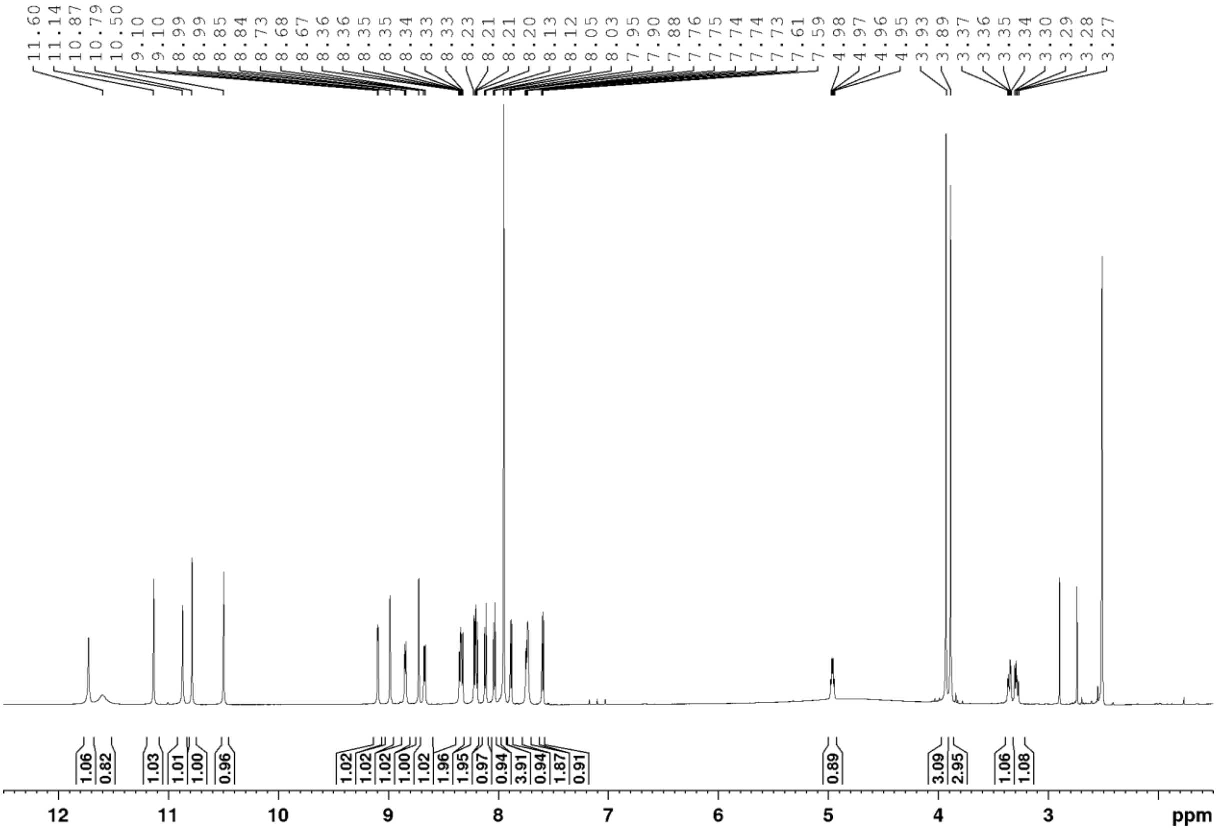

H,H-COSY 700 MHz

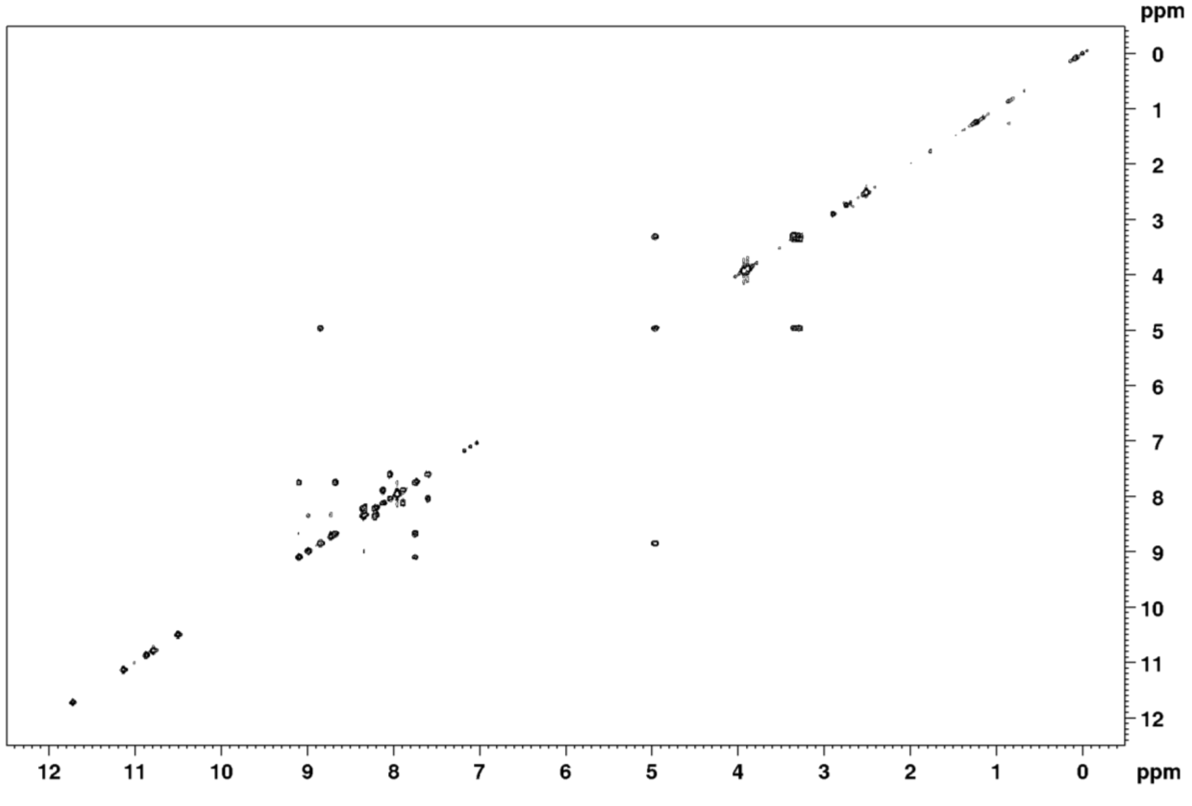

H,C-HMQC – 700 MHz

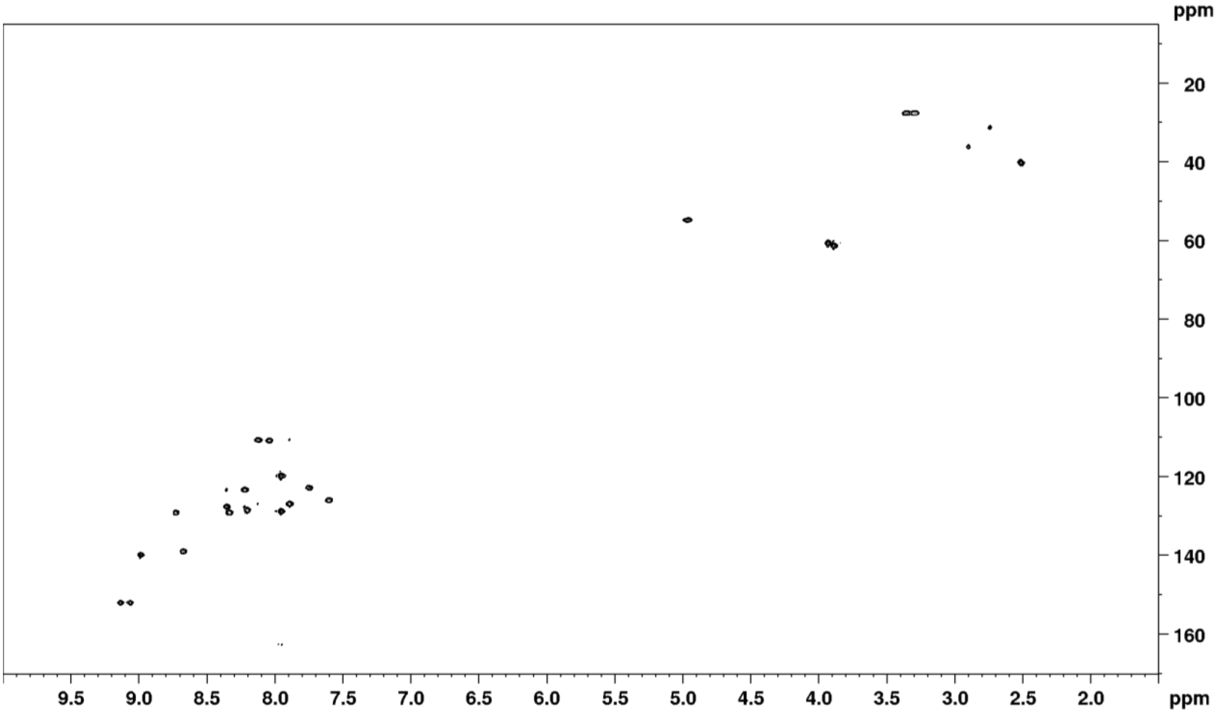

H,C-HMBC – 700 MHz

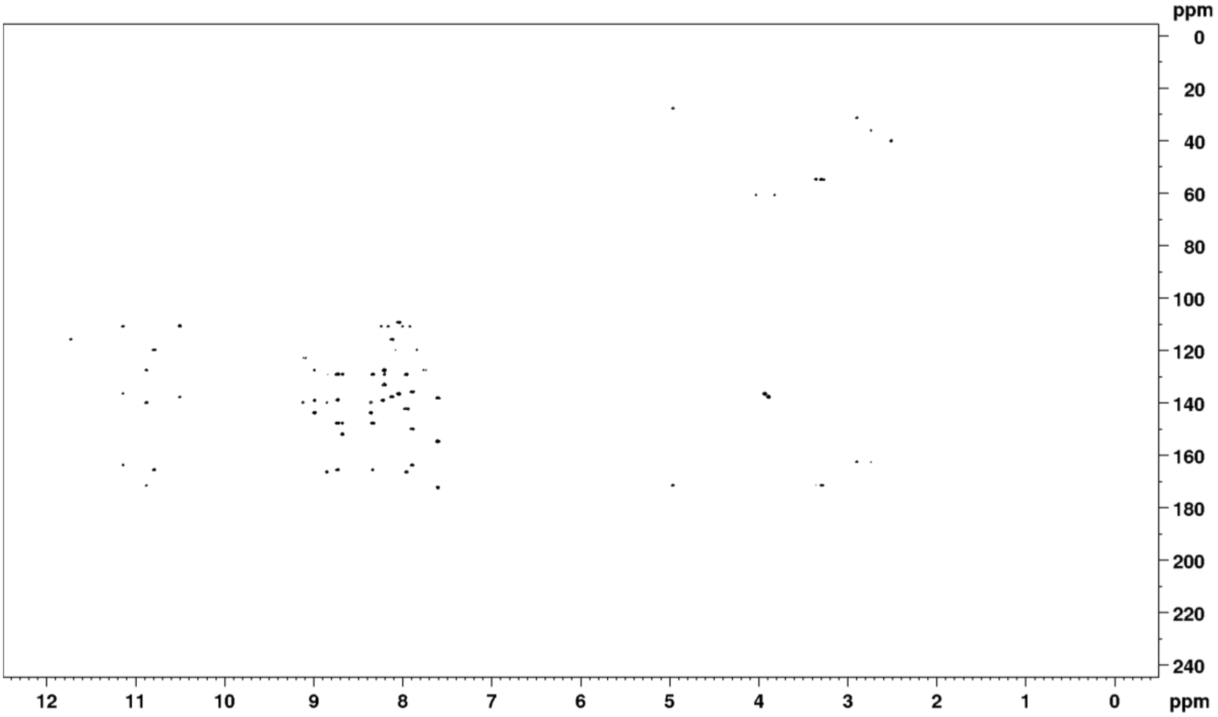

Albicidin derivative 15

^{1}H-NMR 700 MHz

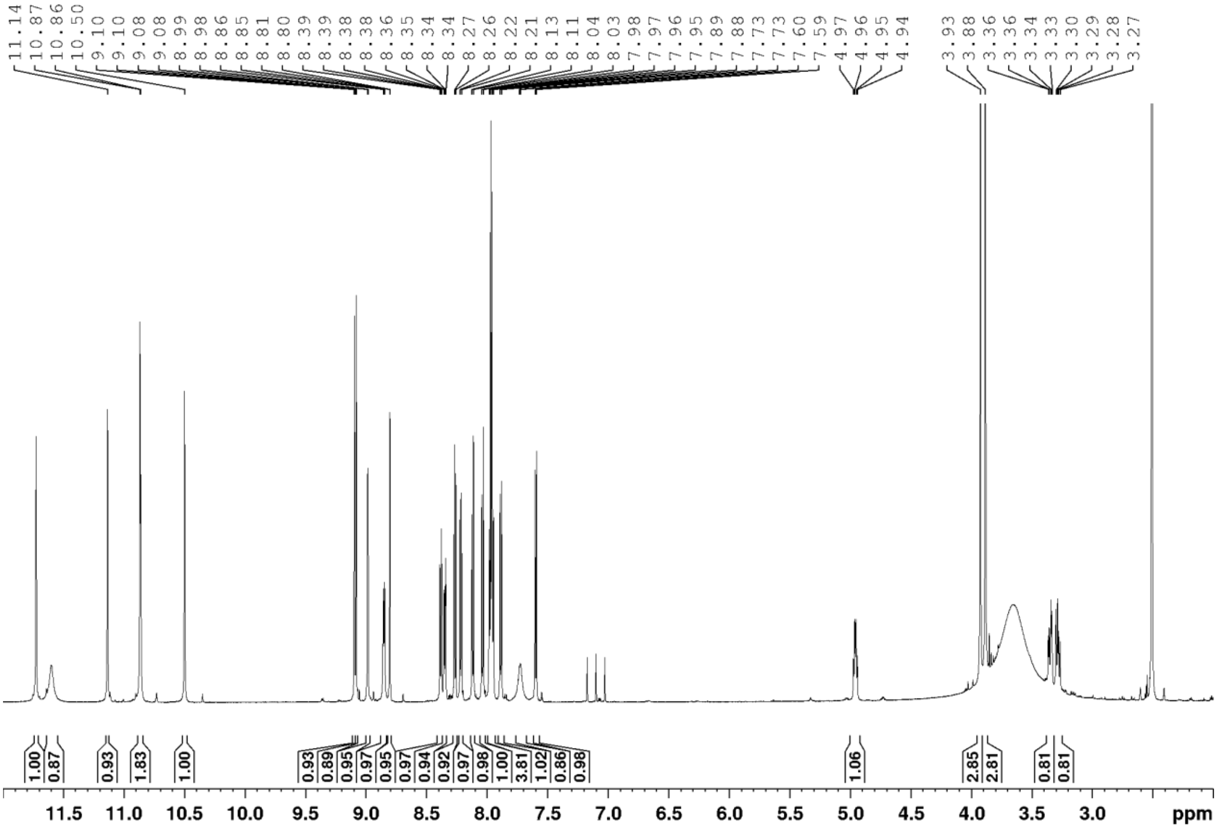

H,H-COSY 700 MHz

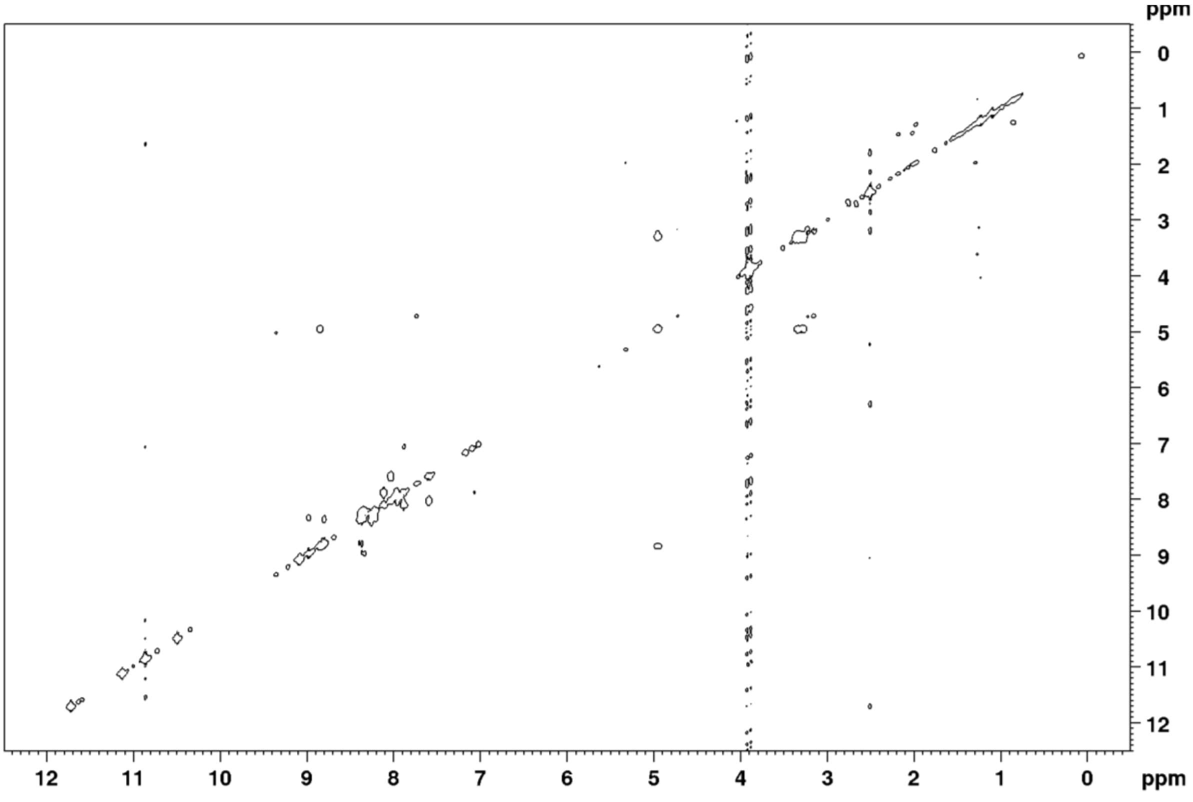

H,C-HMQC – 700 MHz

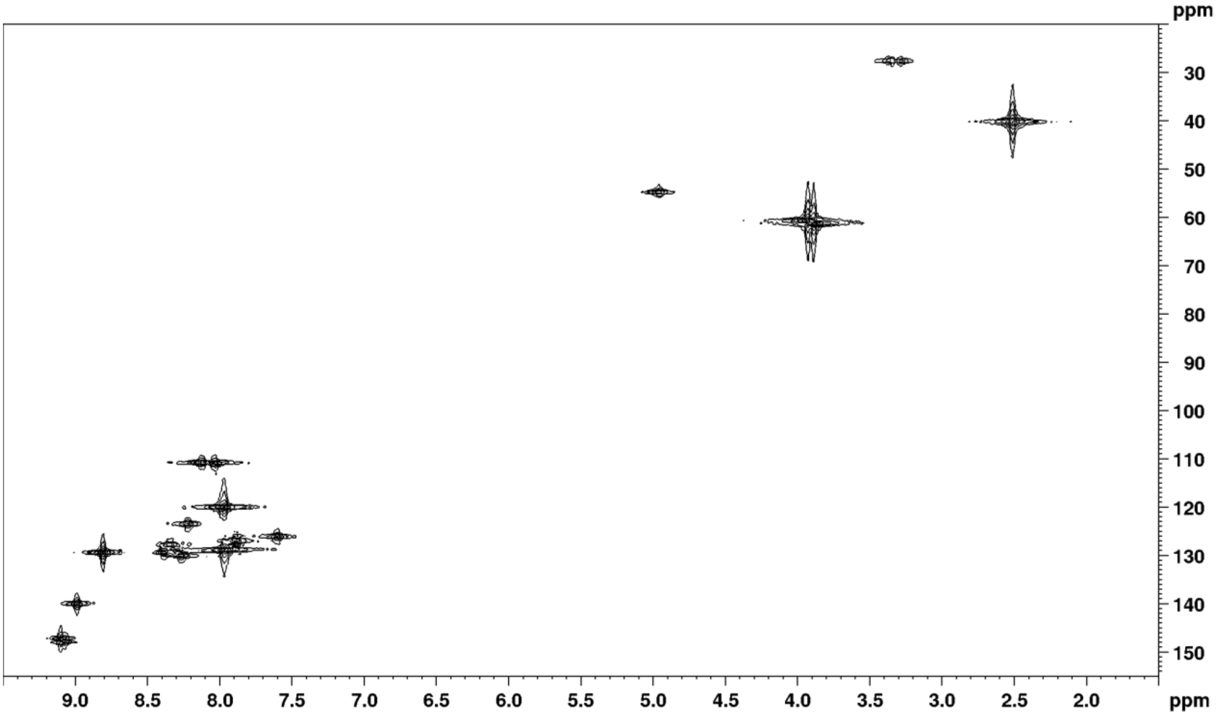

H,C-HMBC – 700 MHz

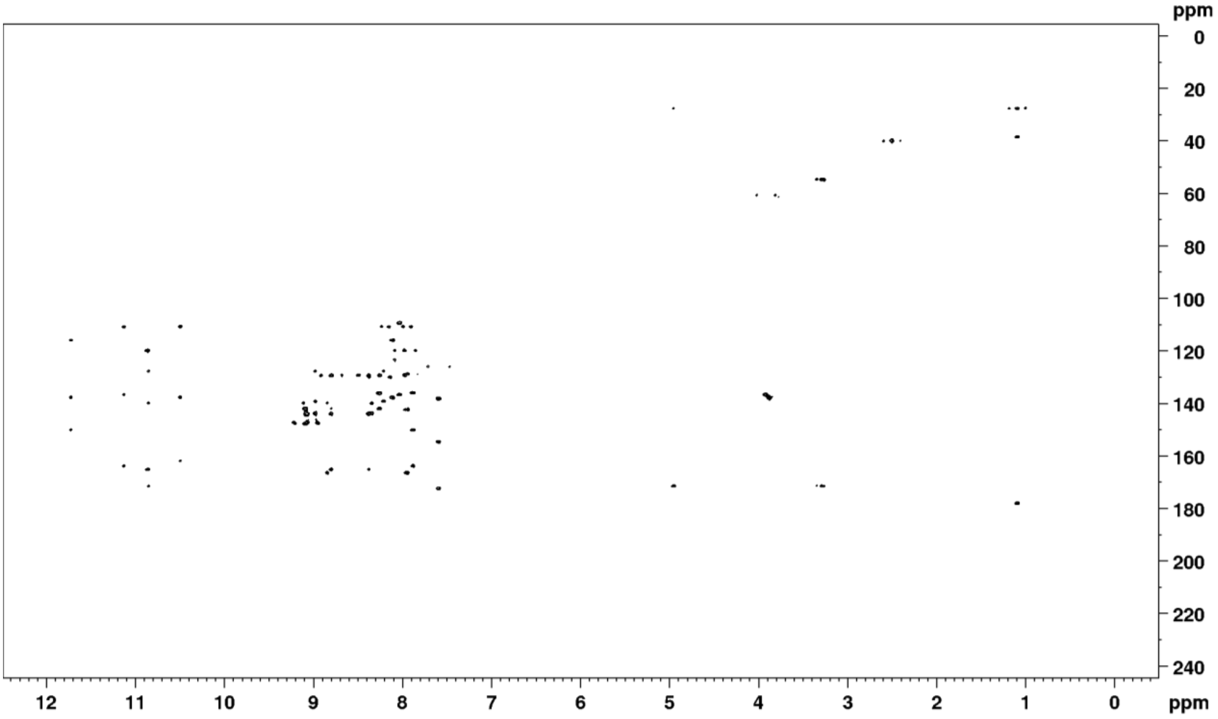

Albicidin derivative 17

^{1}H-NMR 700 MHz

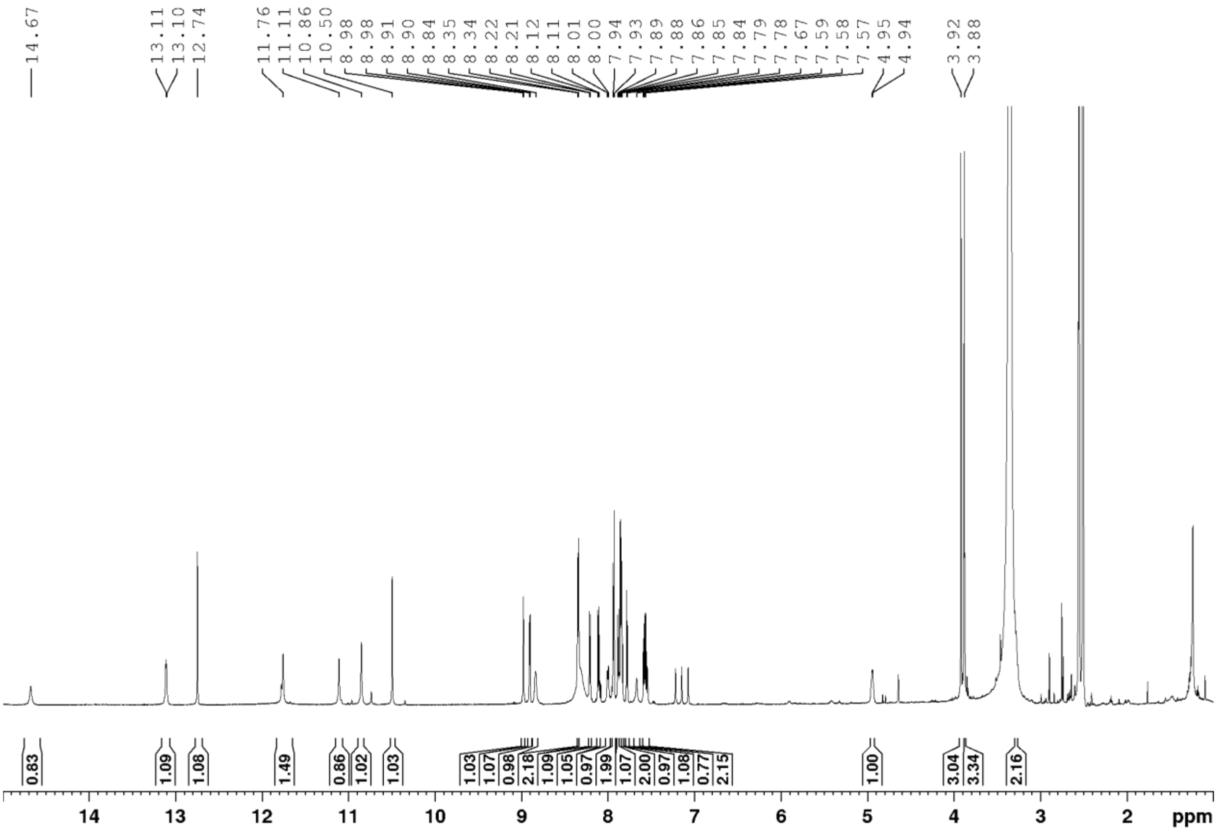

H,H-COSY 700 MHz

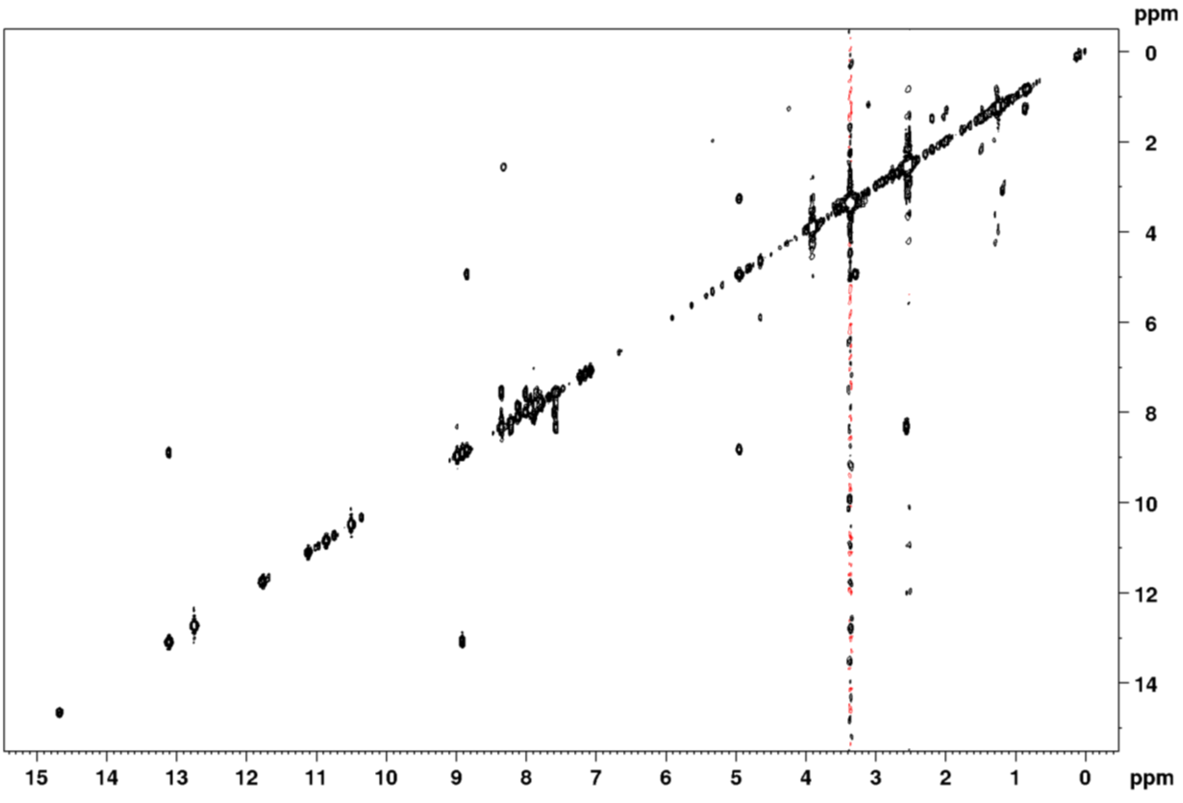

H,C-HMQC – 700 MHz

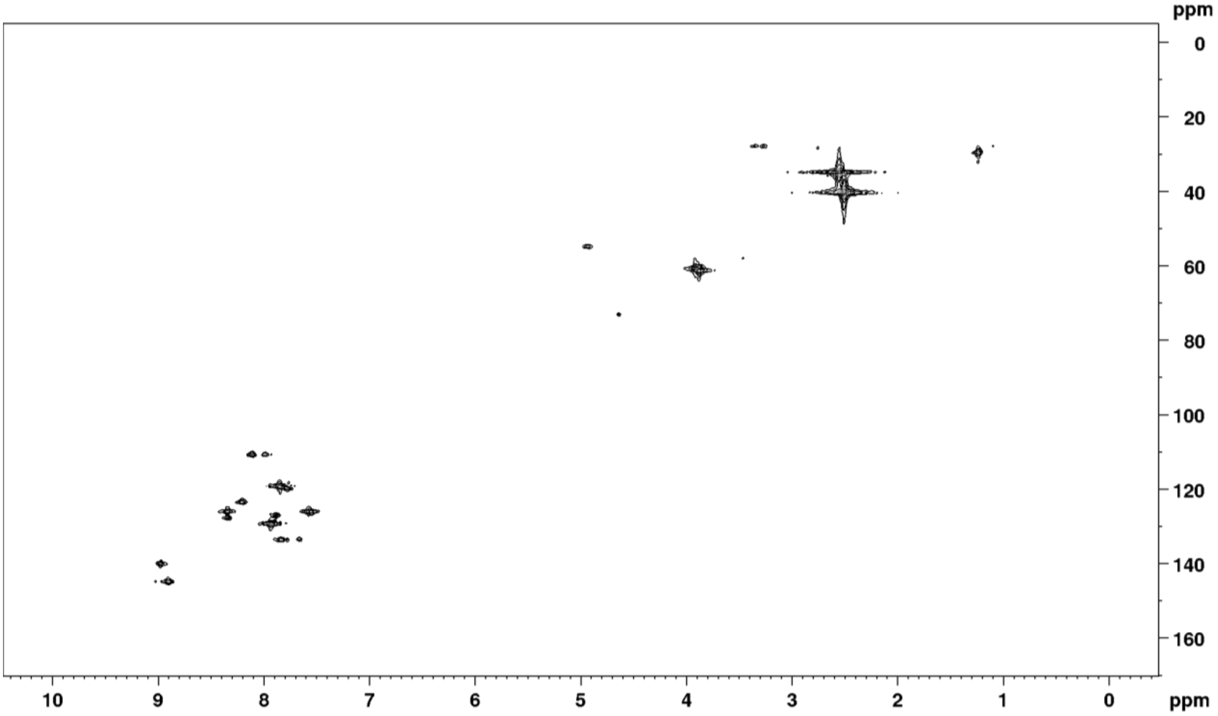

H,C-HMBC – 700 MHz

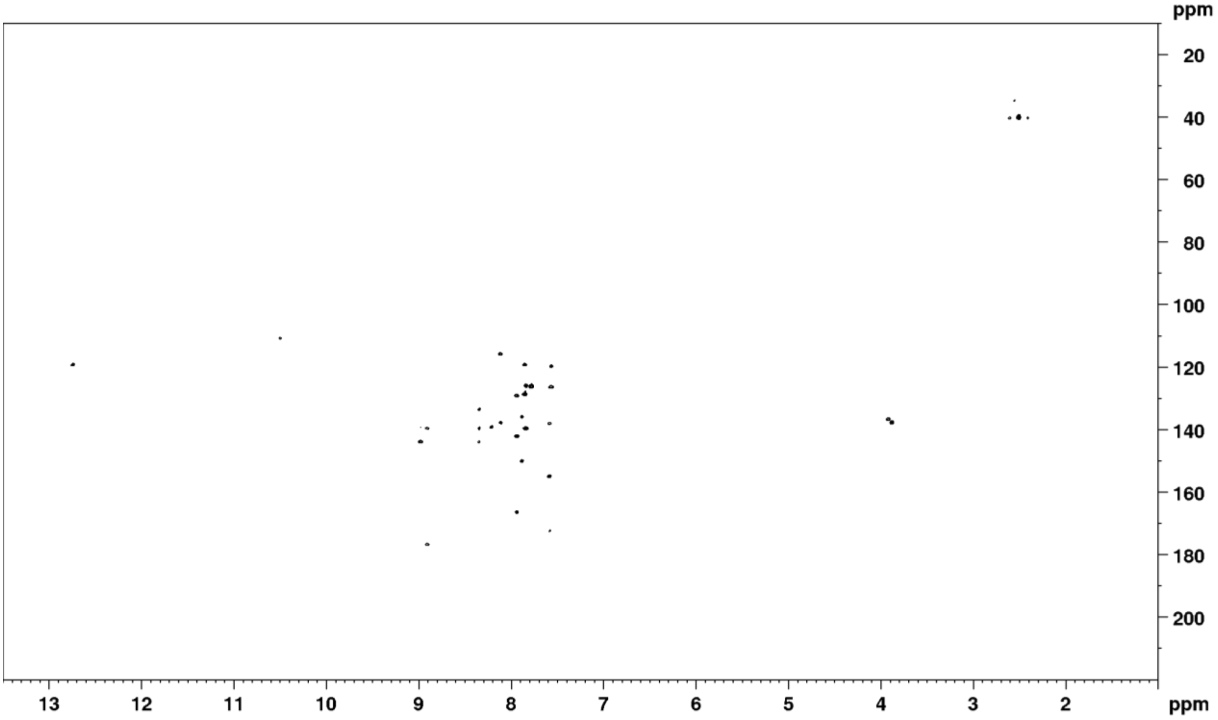

Albicidin derivative 18

^{1}H-NMR 700 MHz

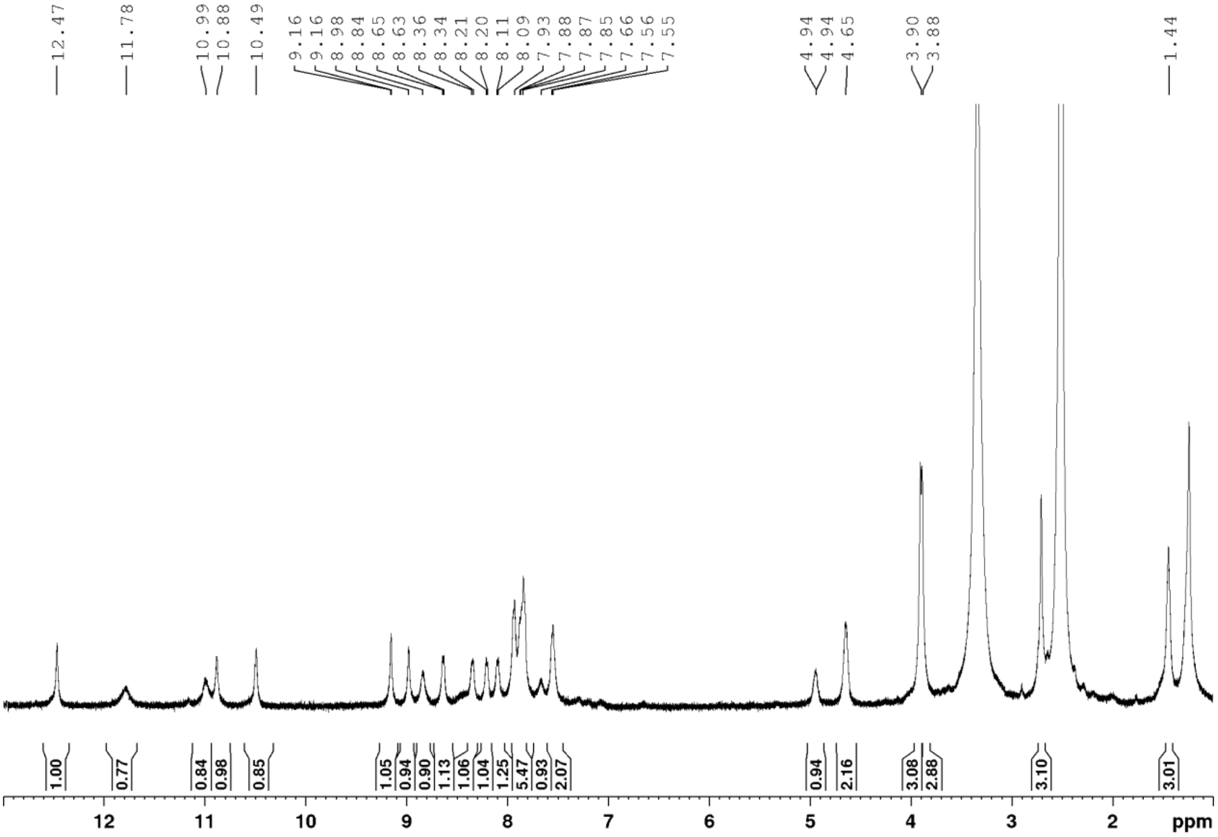

H,H-COSY 700 MHz

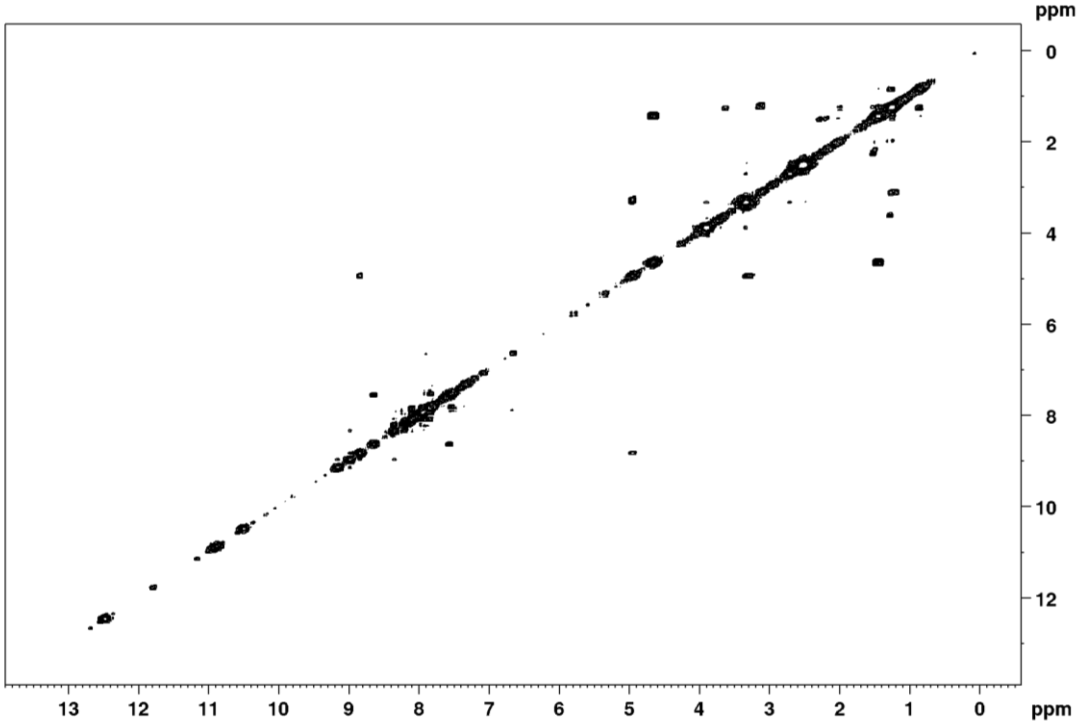

H,C-HMQC – 700 MHz

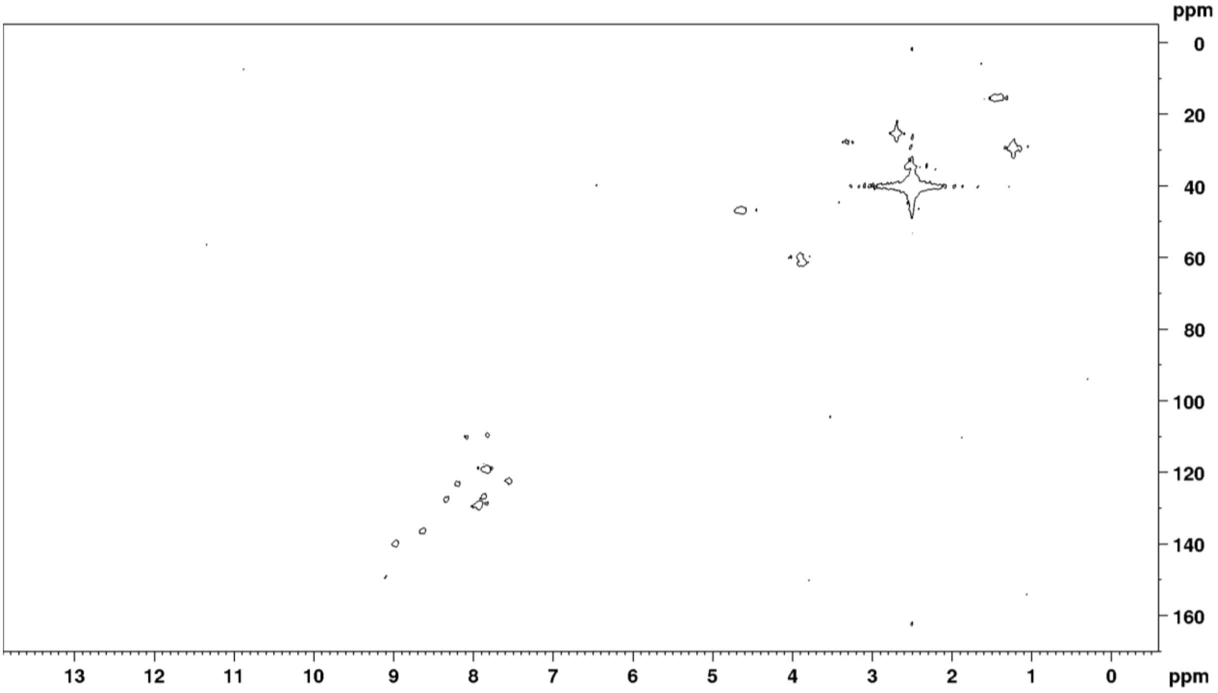

H,C-HMBC – 700 MHz

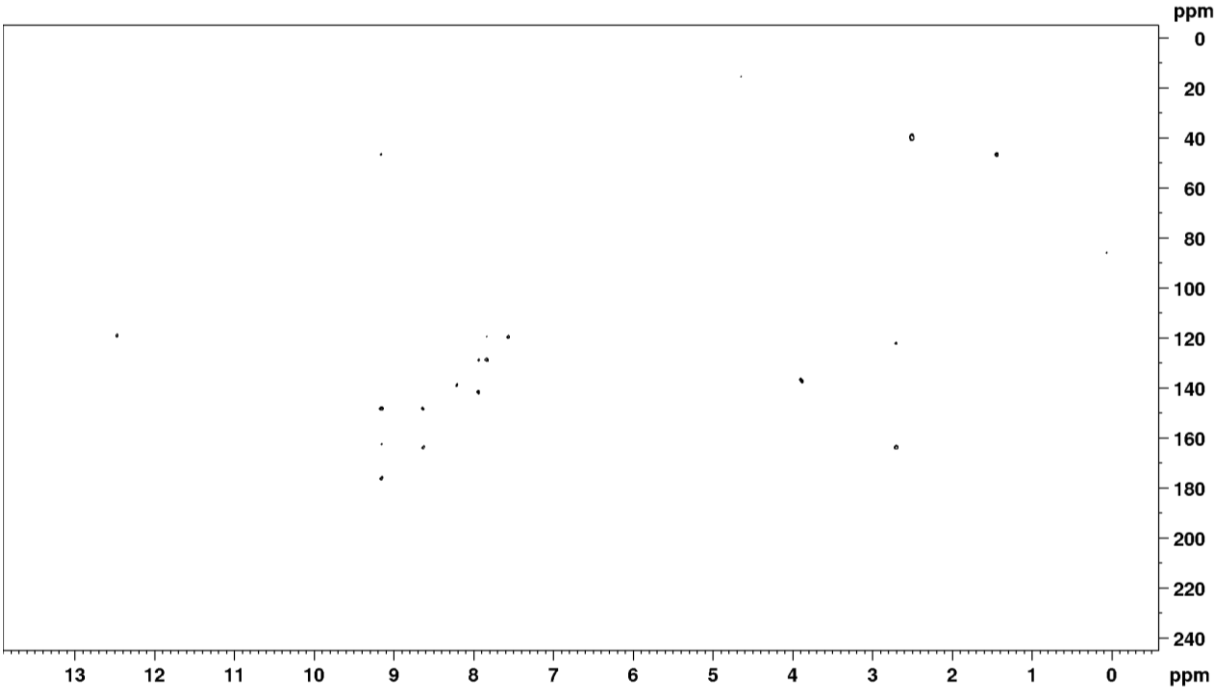

Albicidin derivative 19

^{1}H-NMR 700 MHz

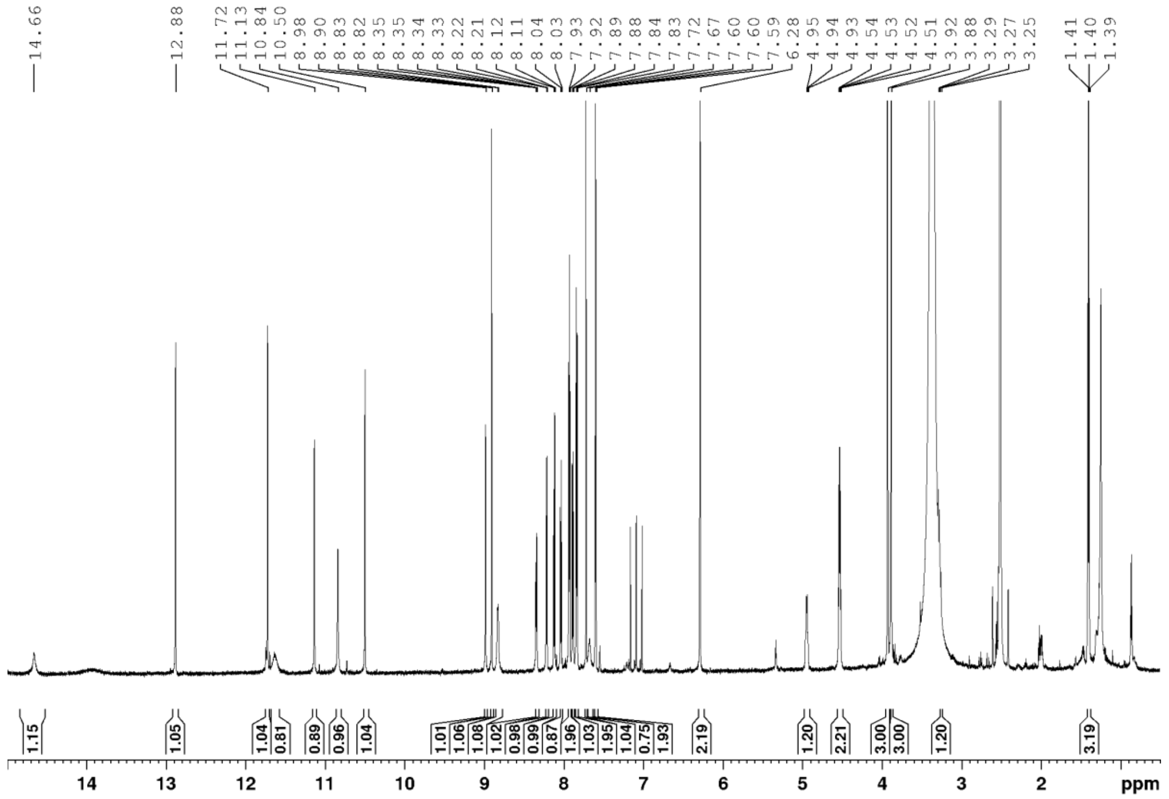

H,H-COSY 700 MHz

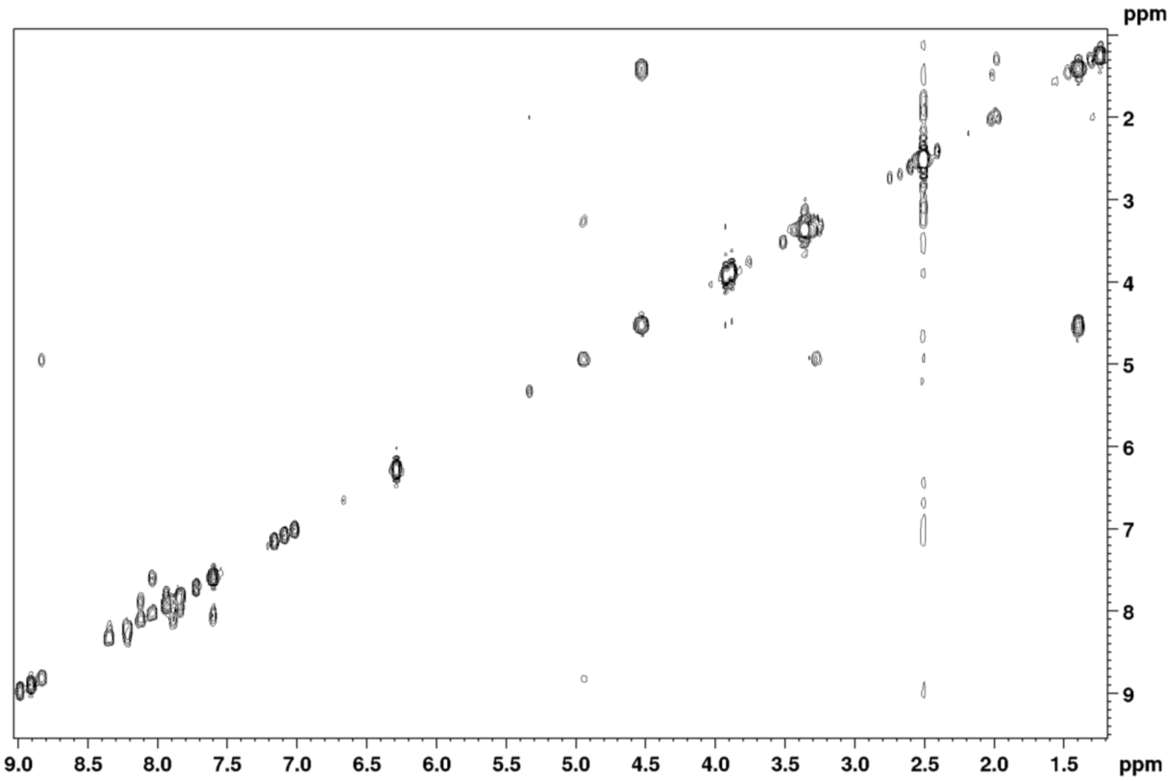

H,C-HSQC – multiplicity edited – 700 MHz

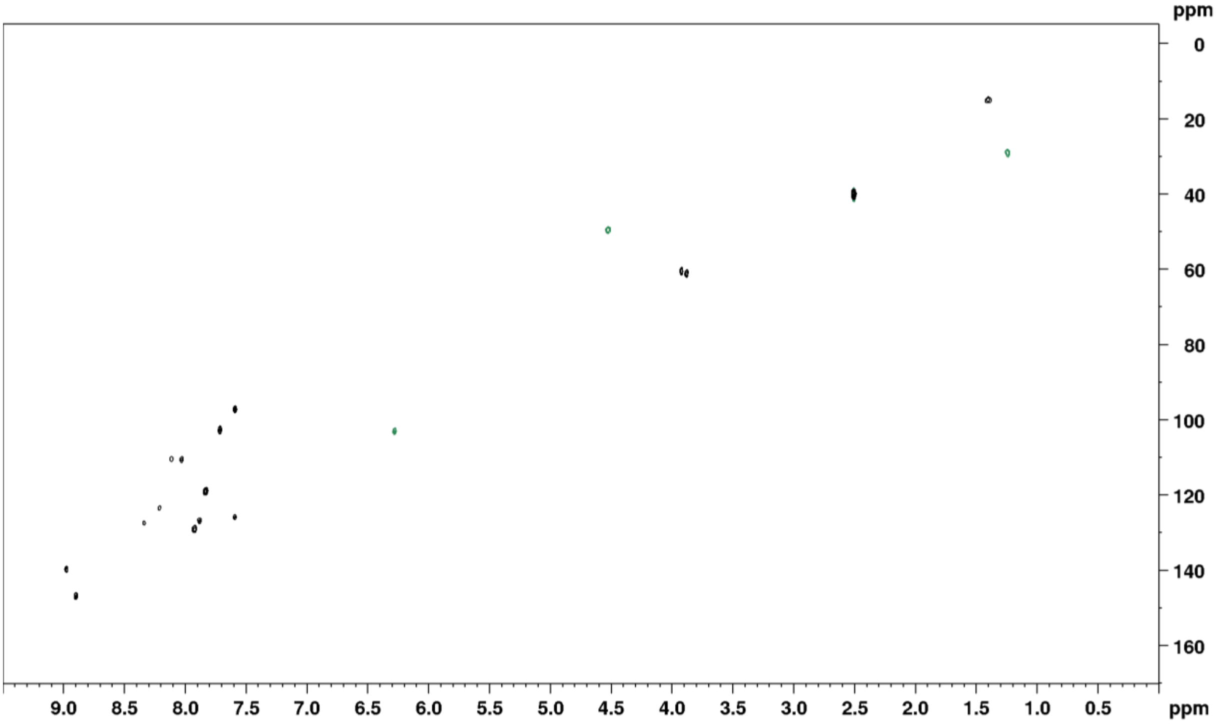

H,C-HMBC – 700 MHz

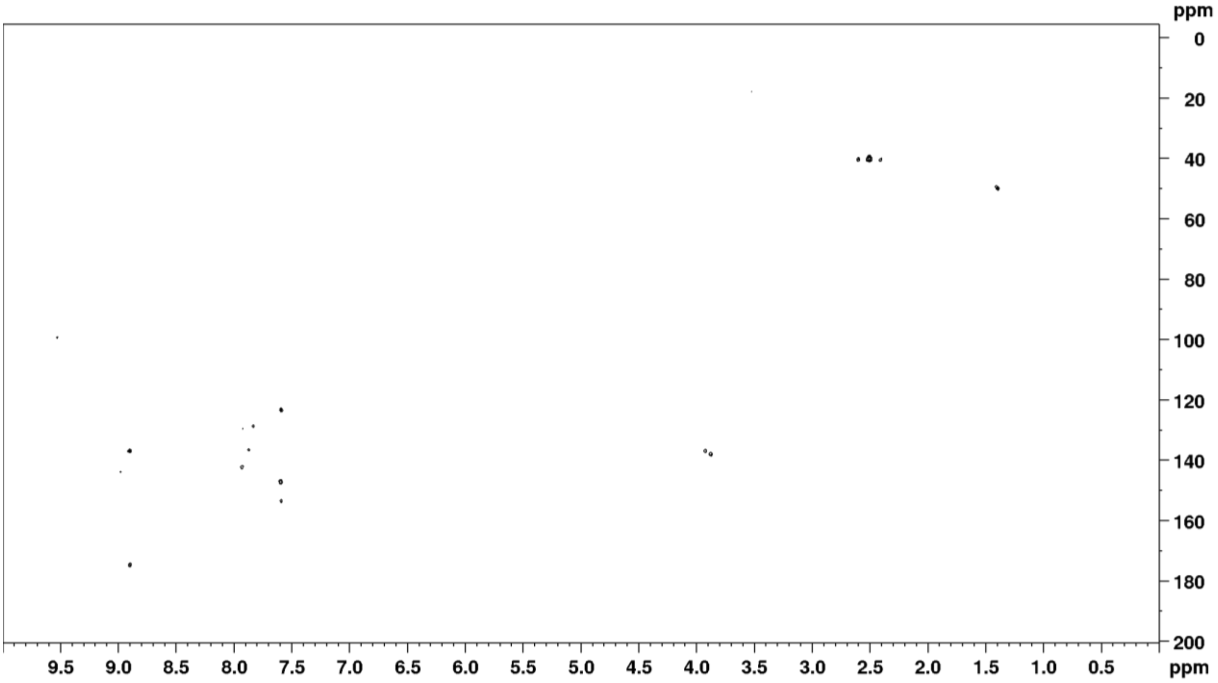

Albicidin derivative 20

^{1}H-NMR 500 MHz

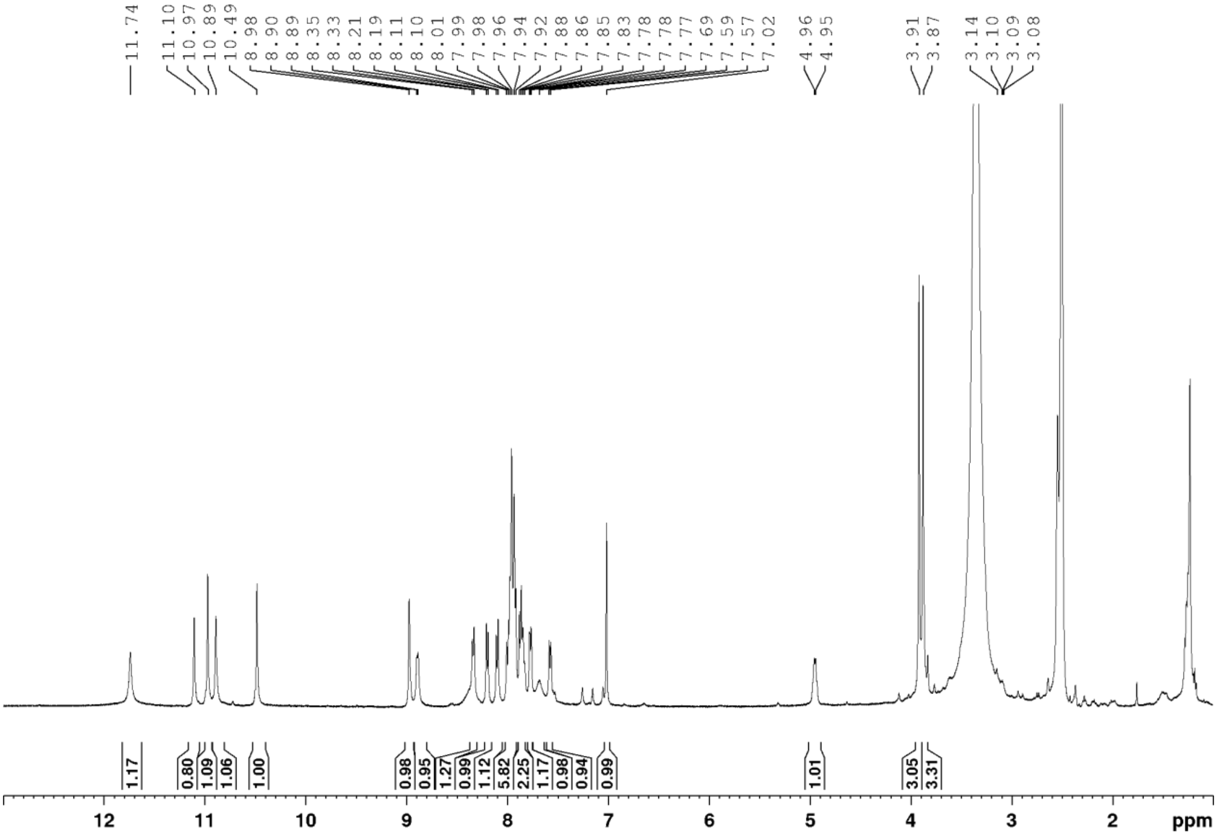

H,H-COSY 500 MHz

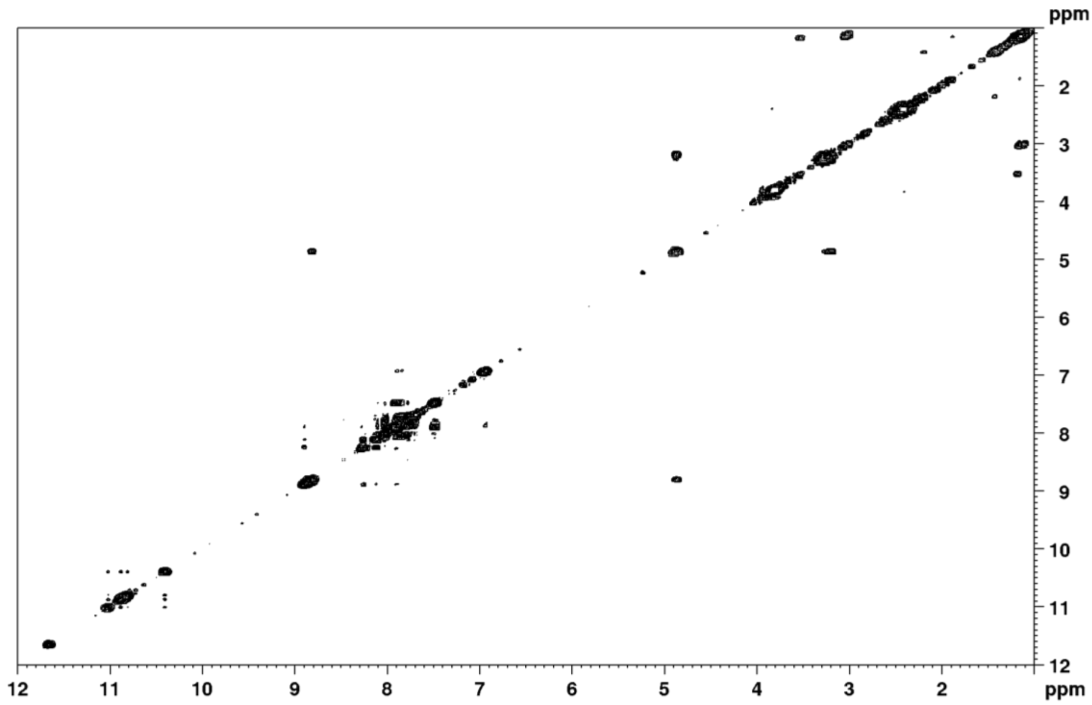

H,C-HMQC – 500 MHz

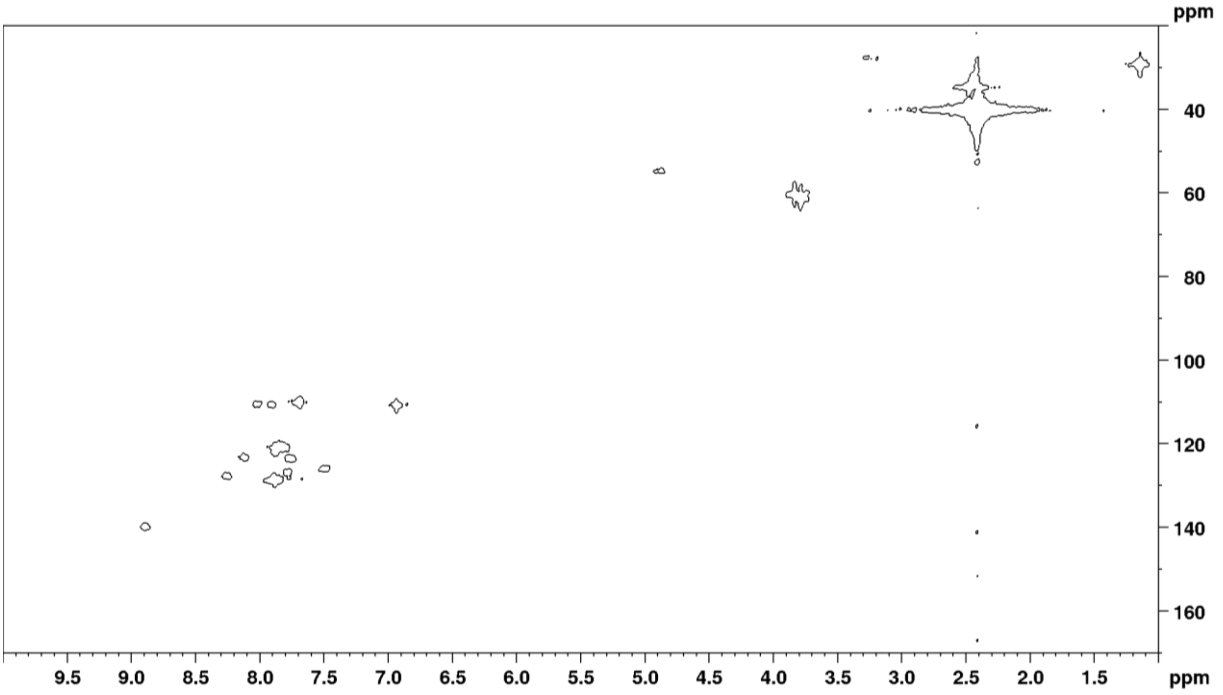

H,C-HMBC – 500 MHz

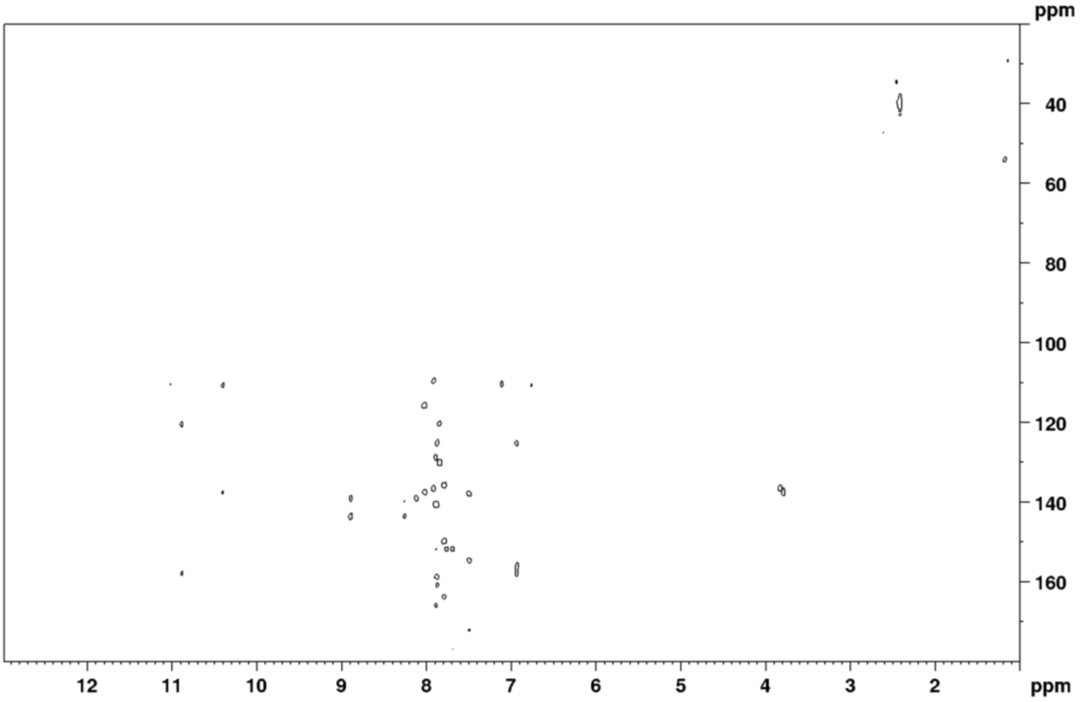

Albicidin derivative 21

^{1}H-NMR 700 MHz

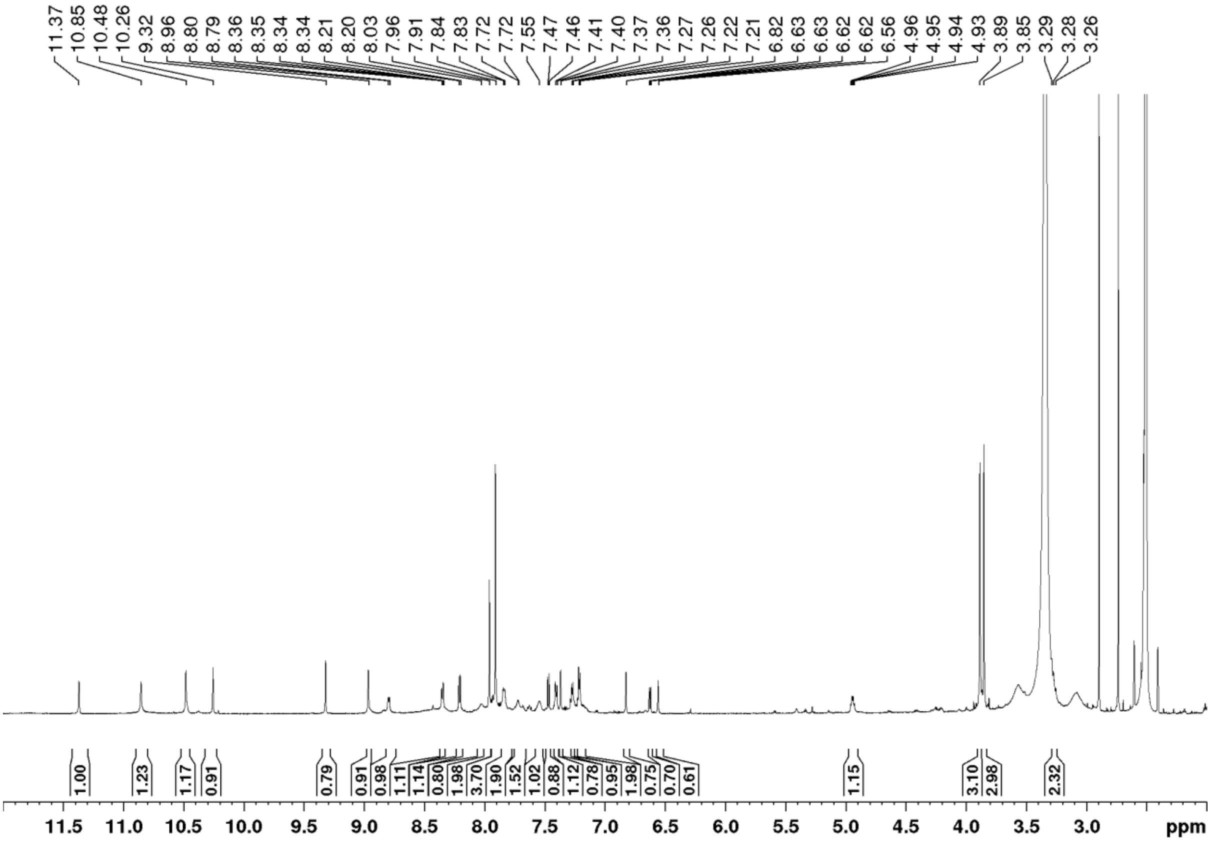

H,H-COSY 700 MHz

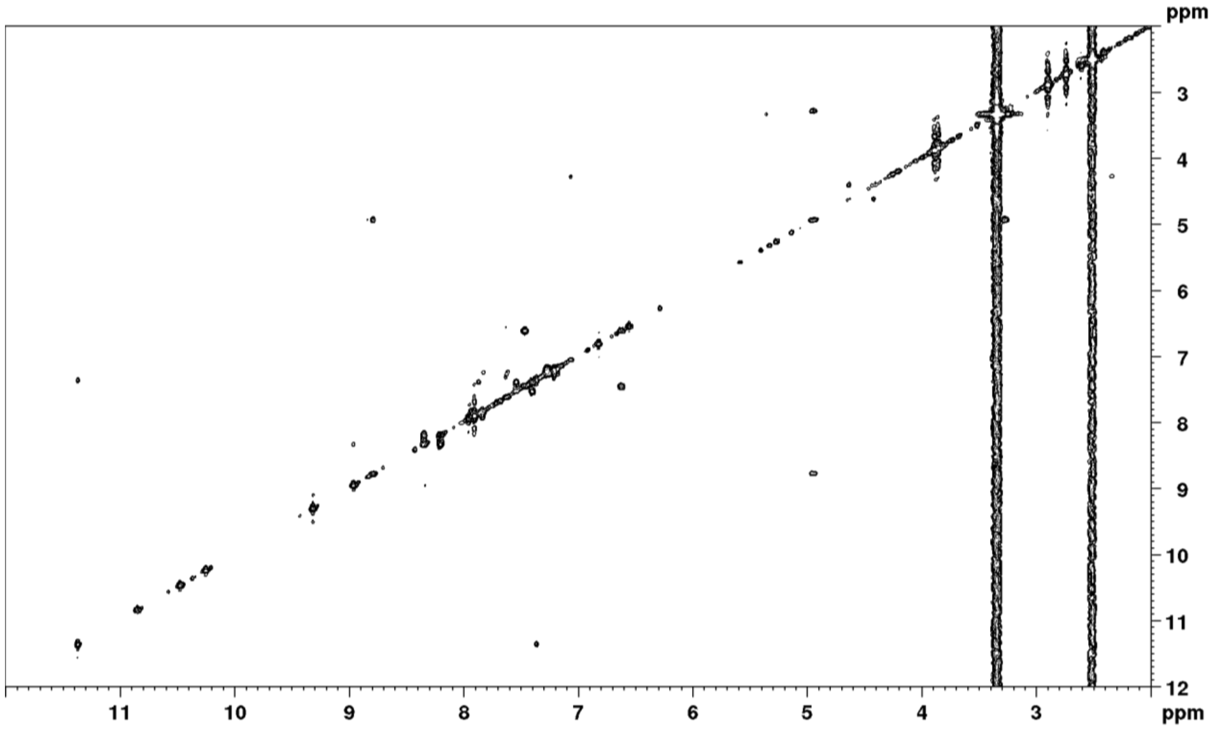

H,C-HSQC – 700 MHz

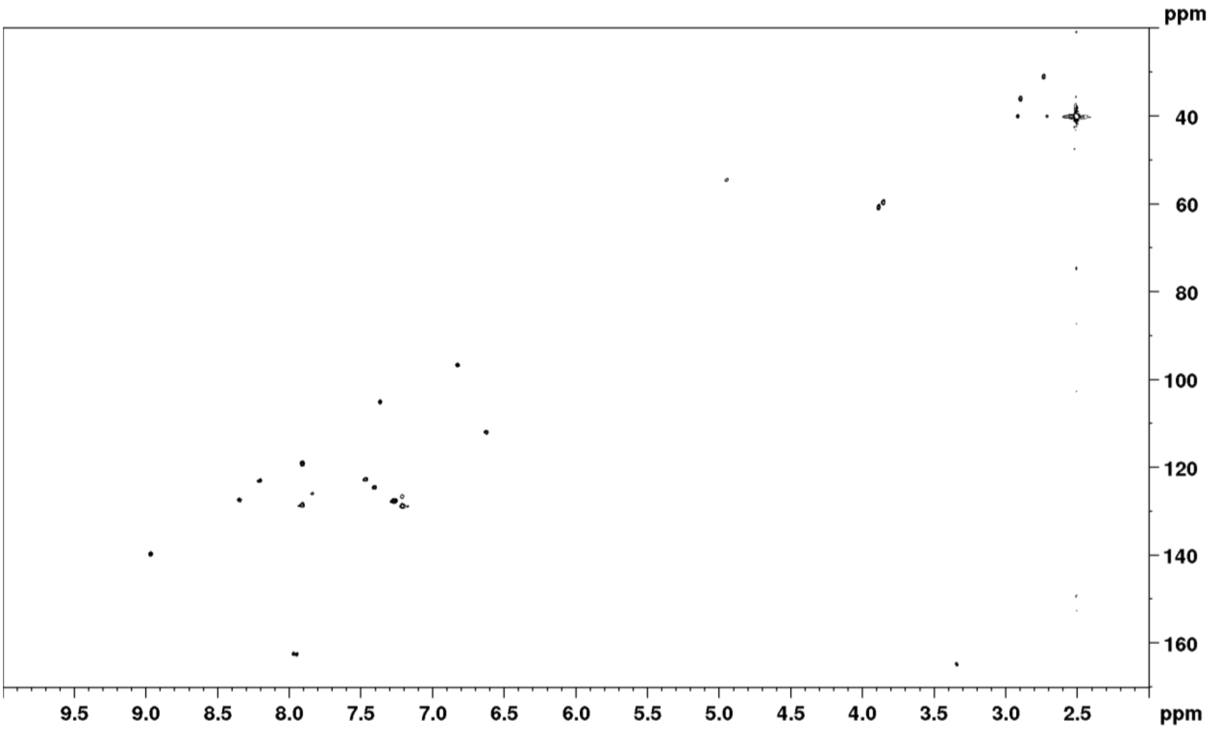

Albicidin derivative 22

^{1}H-NMR 700 MHz

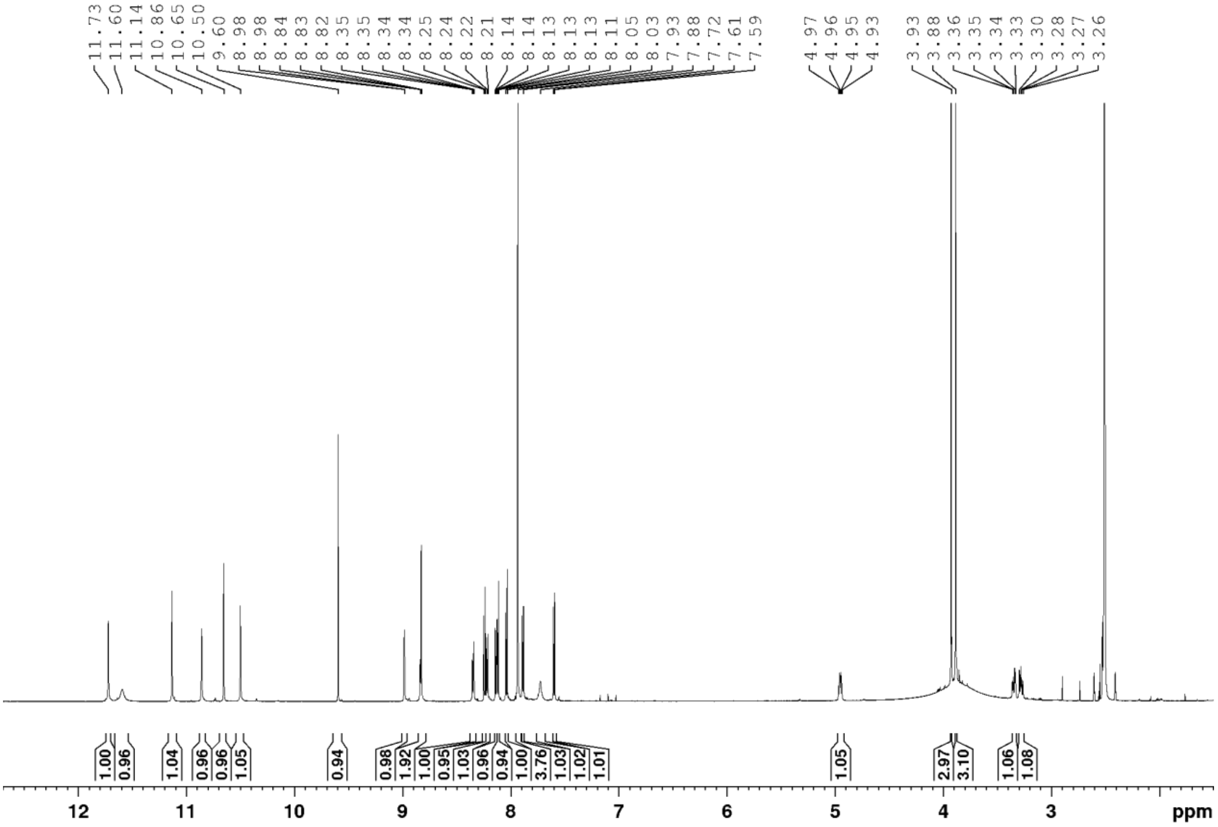

H,H-COSY 700 MHz

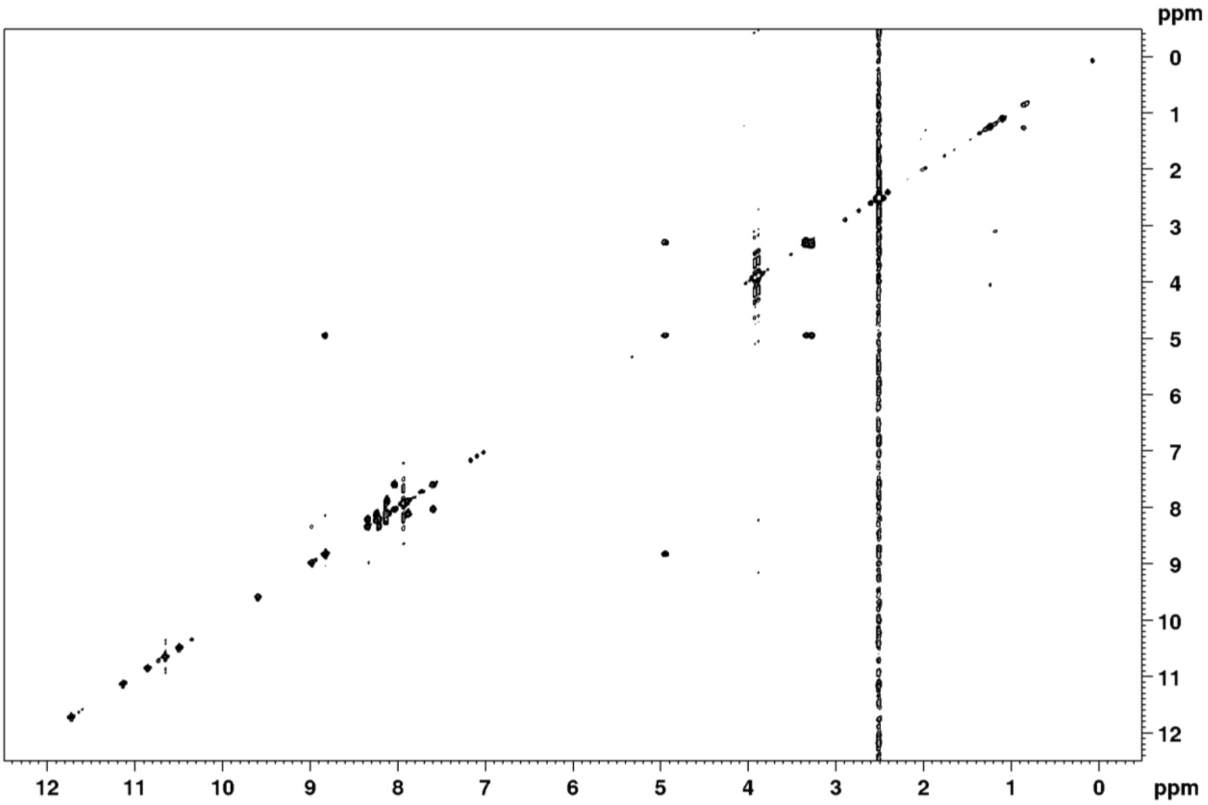

H,C-HSQC – 700 MHz

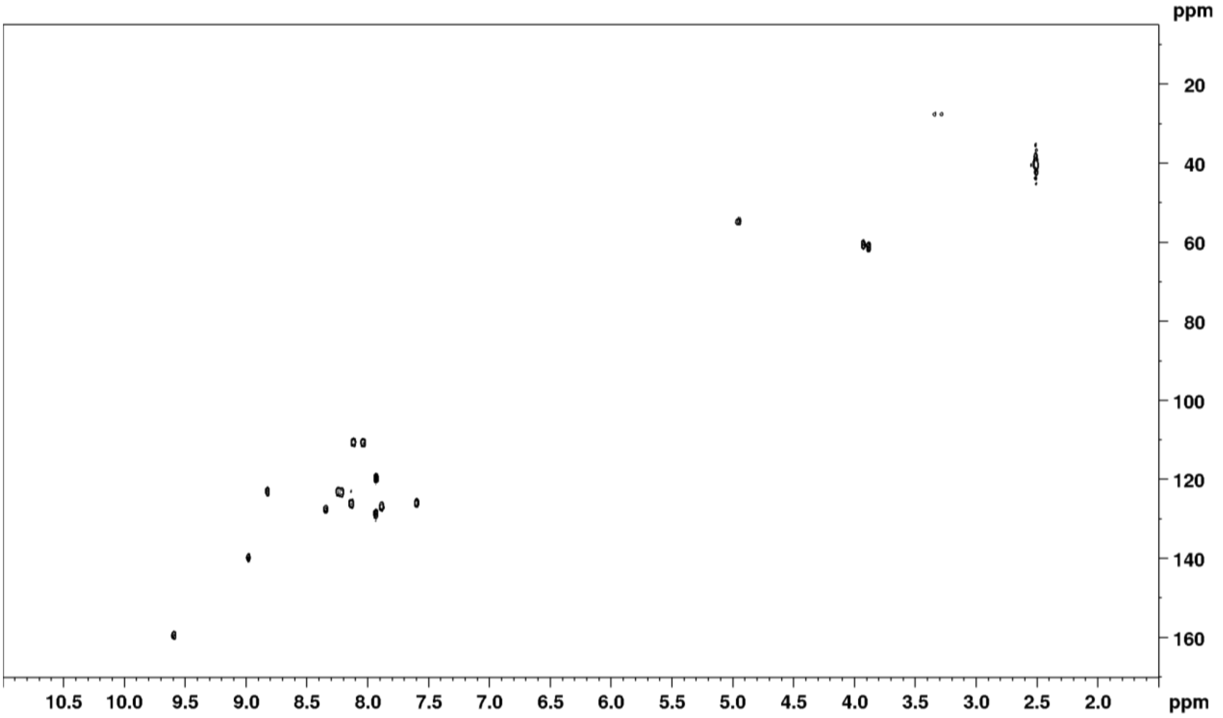

H,C-HMBC – 700 MHz

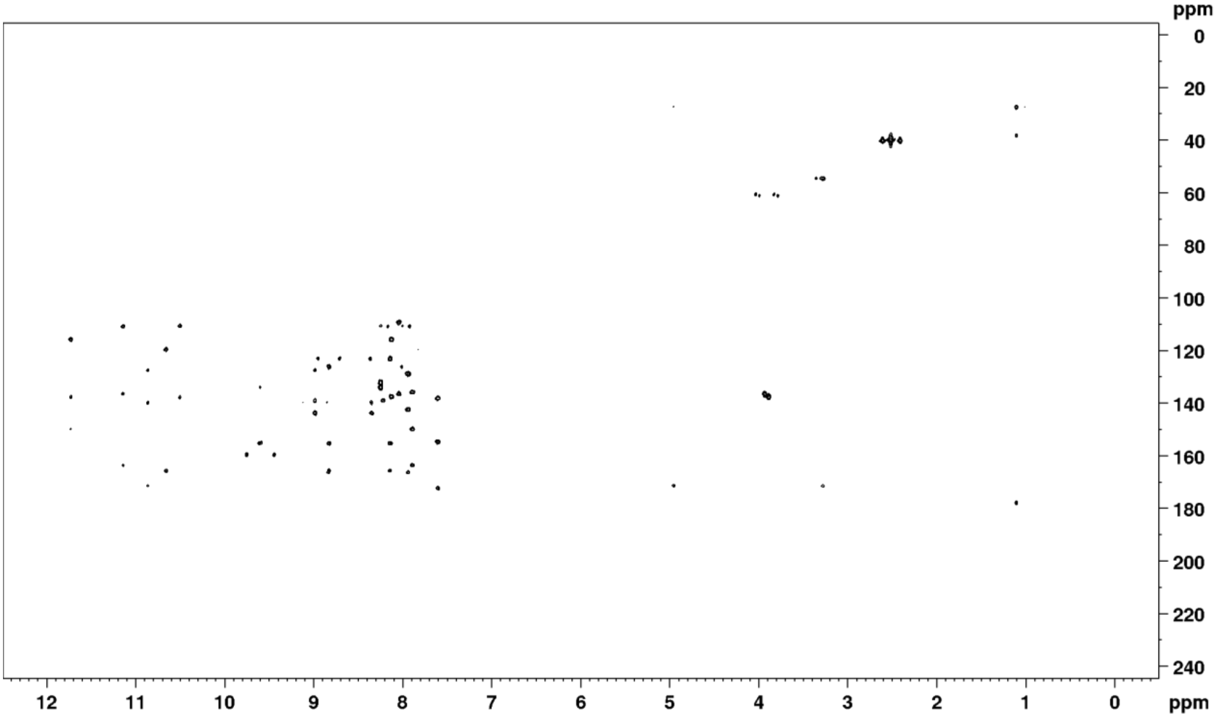

Albicidin derivative 23

^{1}H-NMR 700 MHz

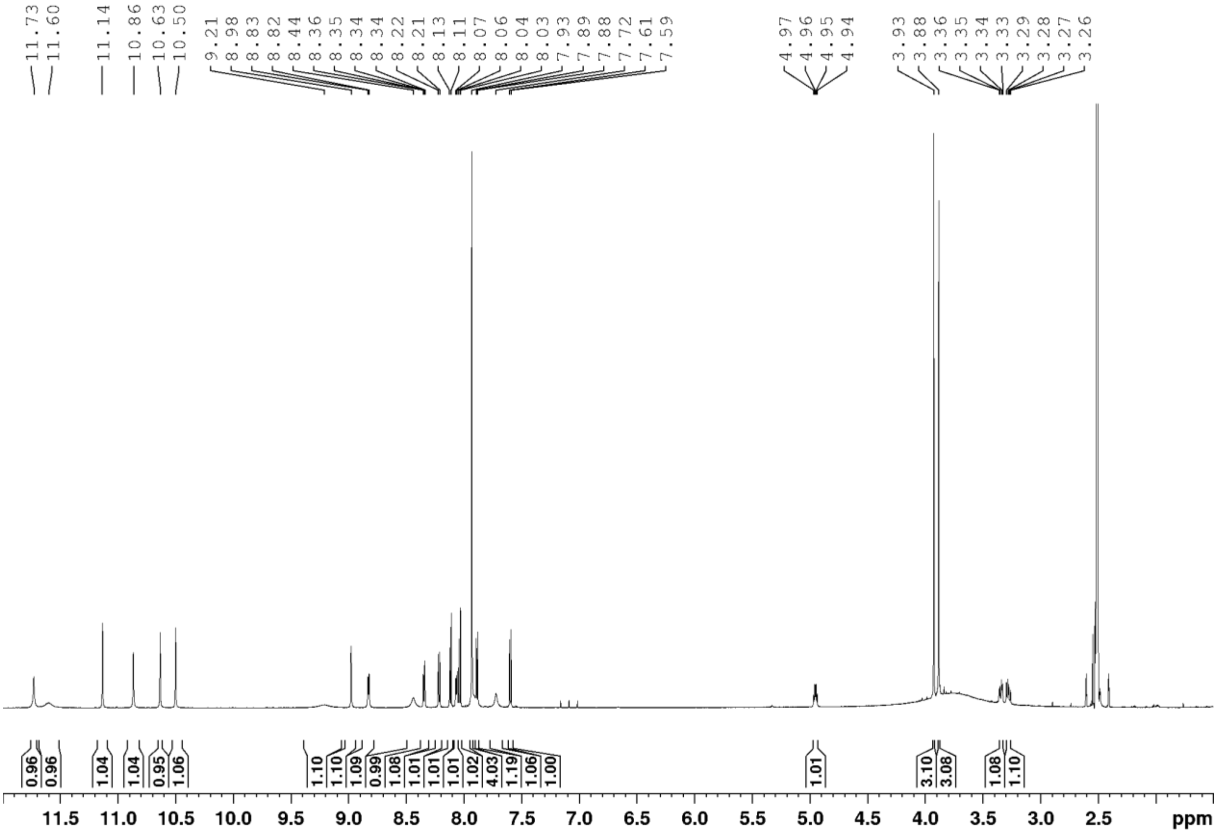

H,H-COSY 700 MHz

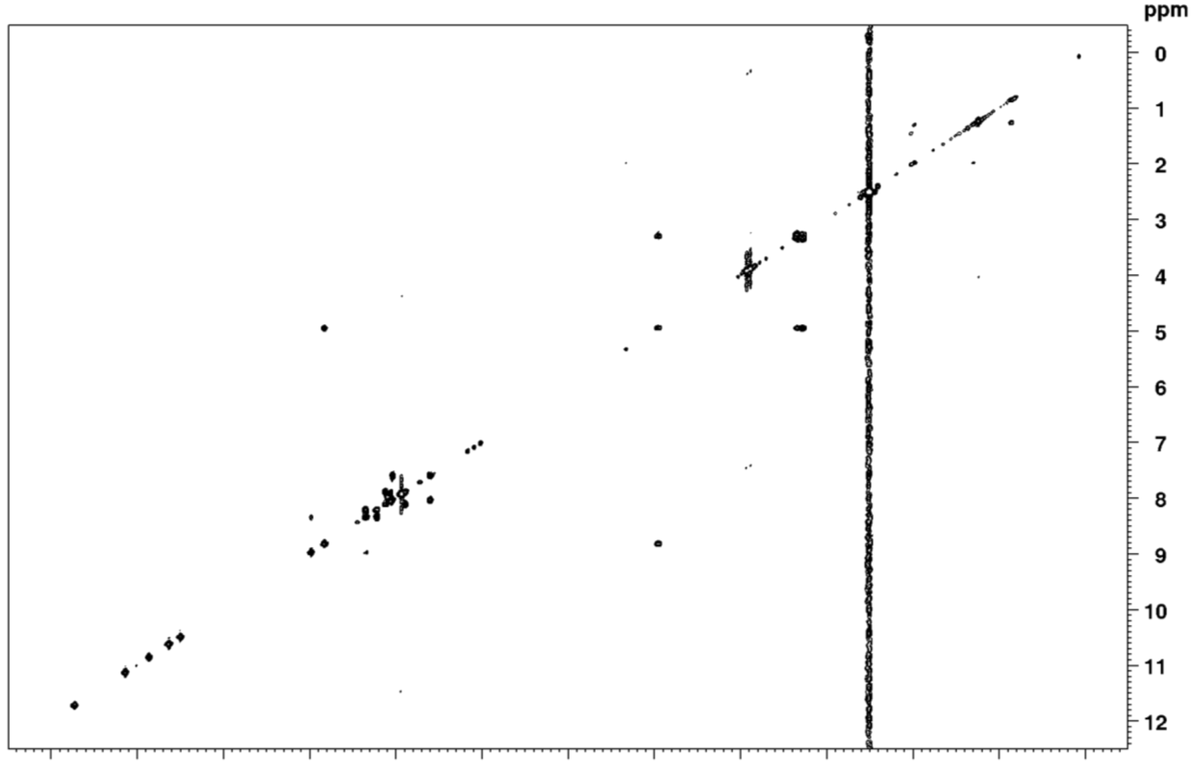

H,C-HMQC – 700 MHz

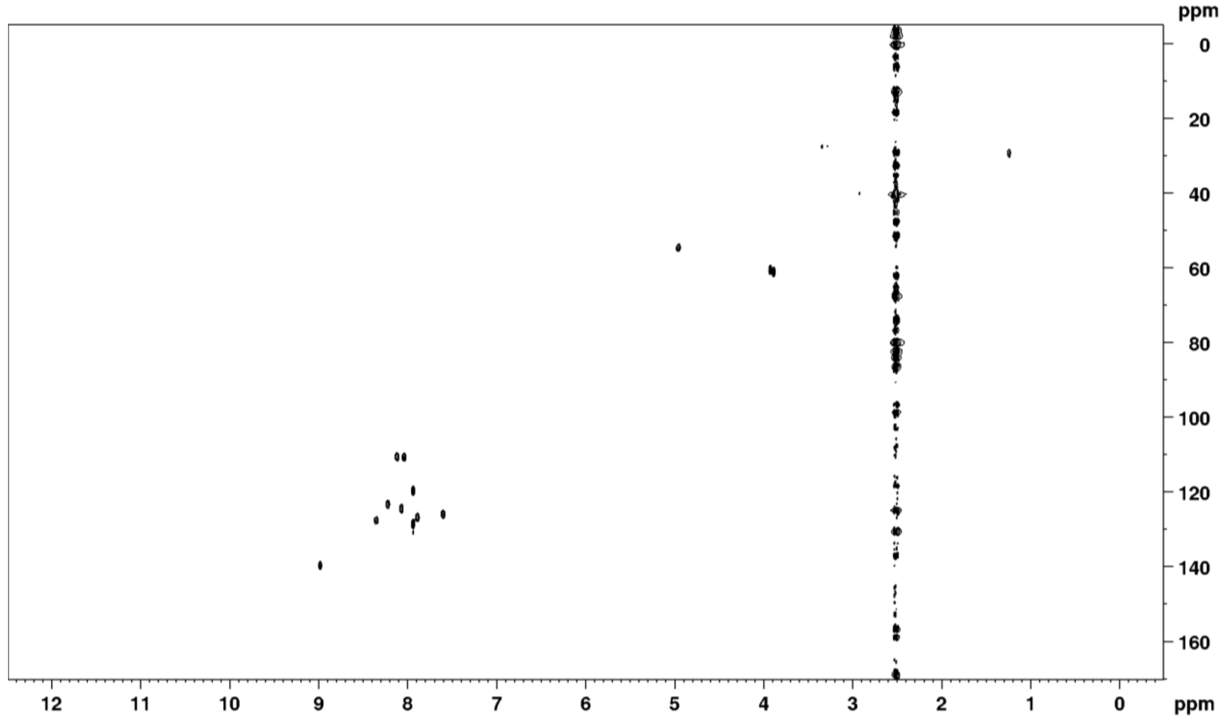

H,C-HMBC – 700 MHz

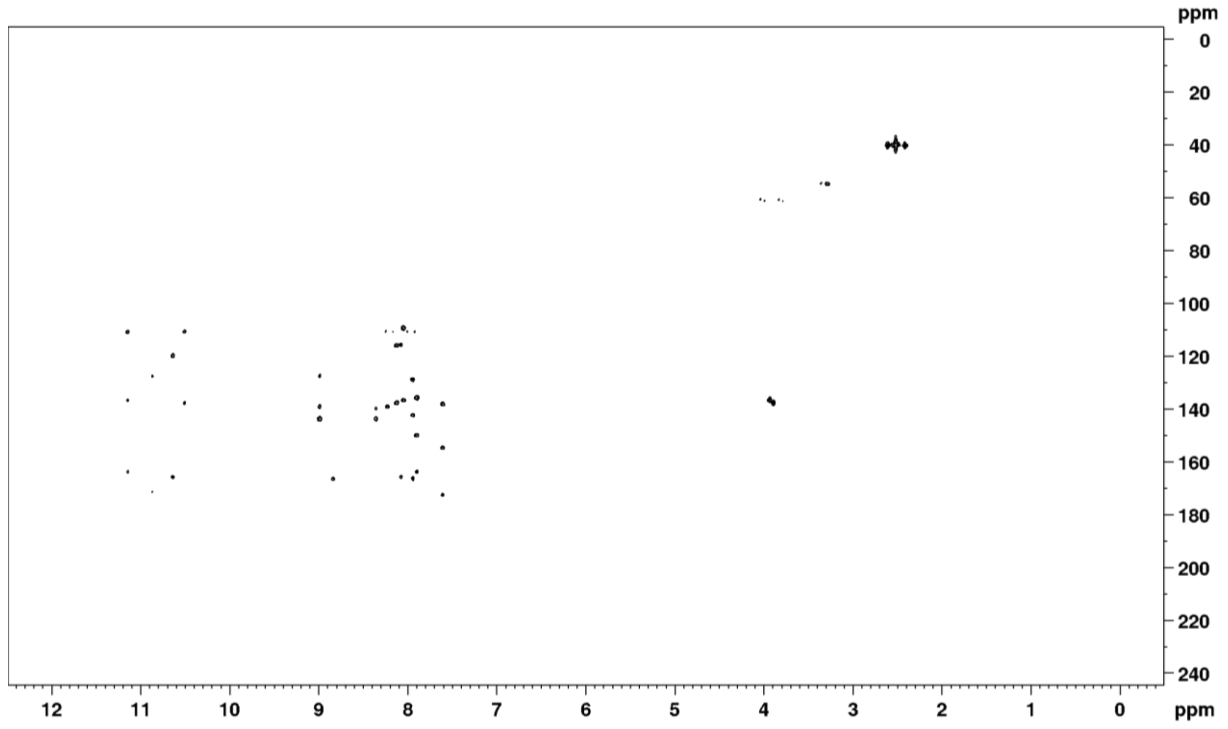

Albicidin derivative 24

^{1}H-NMR 700 MHz

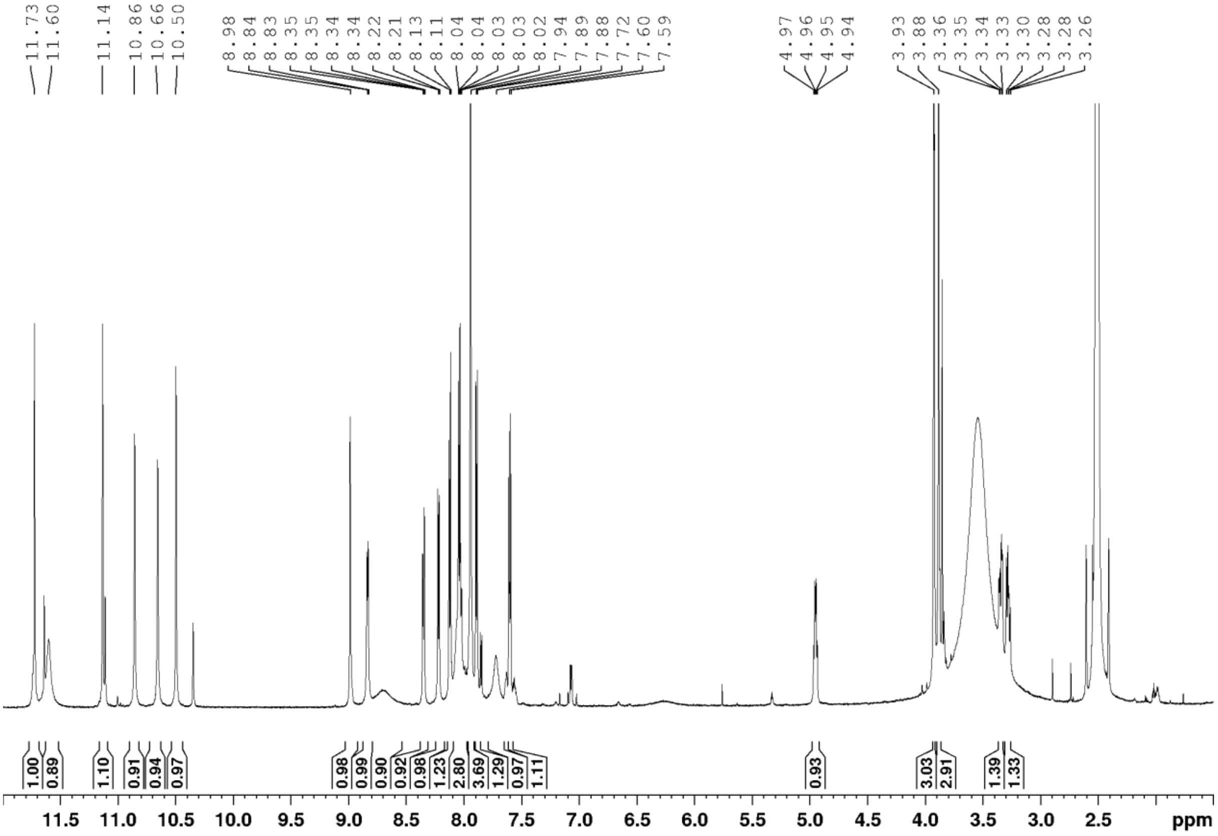

H,H-COSY 700 MHz

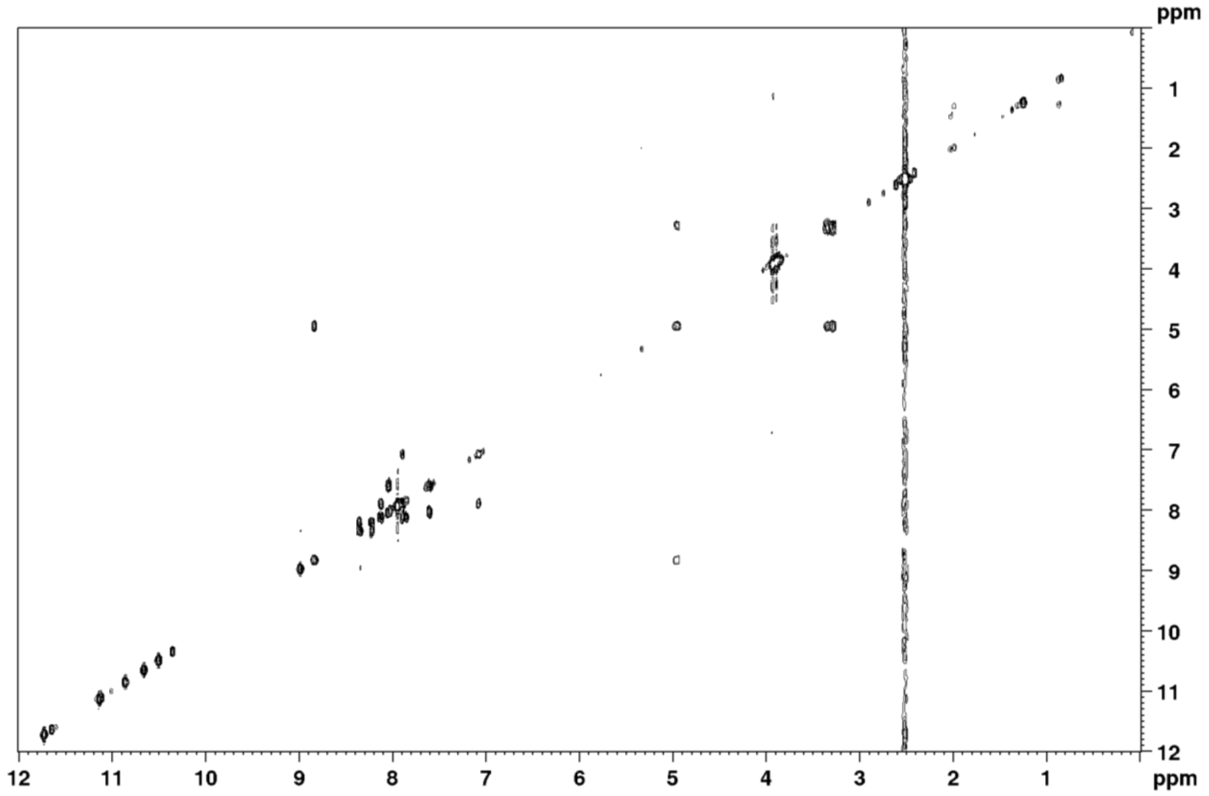

H,C-HMQC – 700 MHz

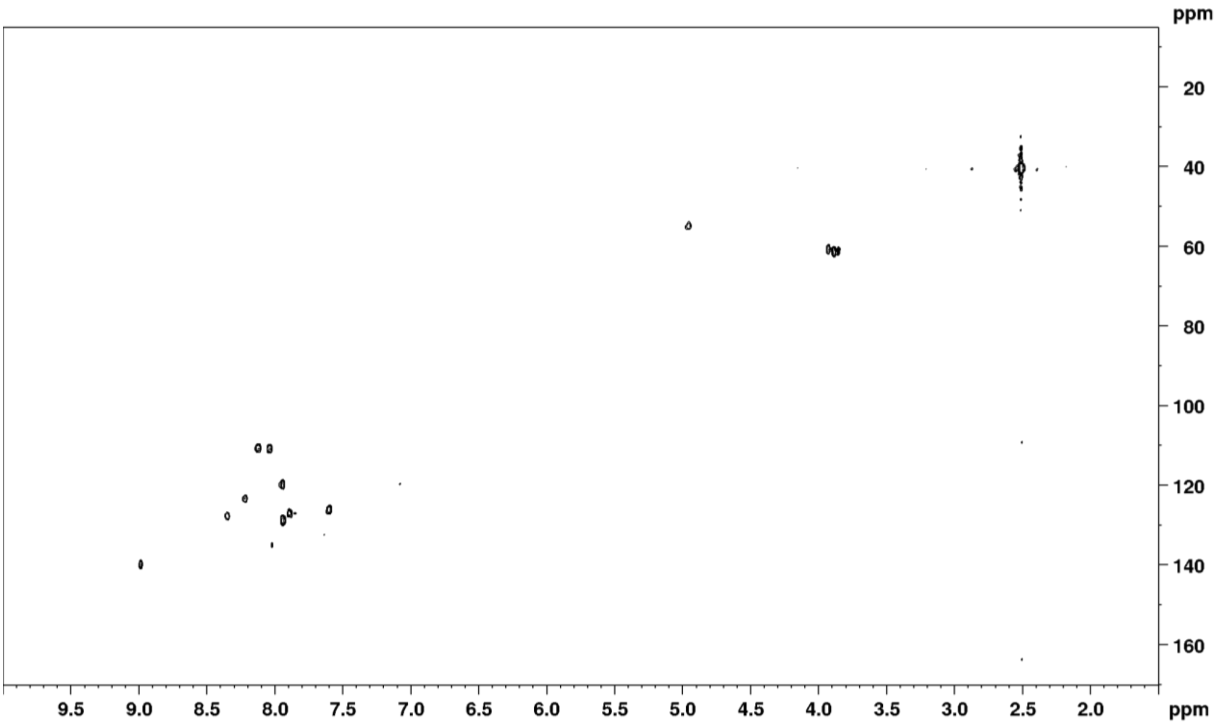

H,C-HMBC – 700 MHz

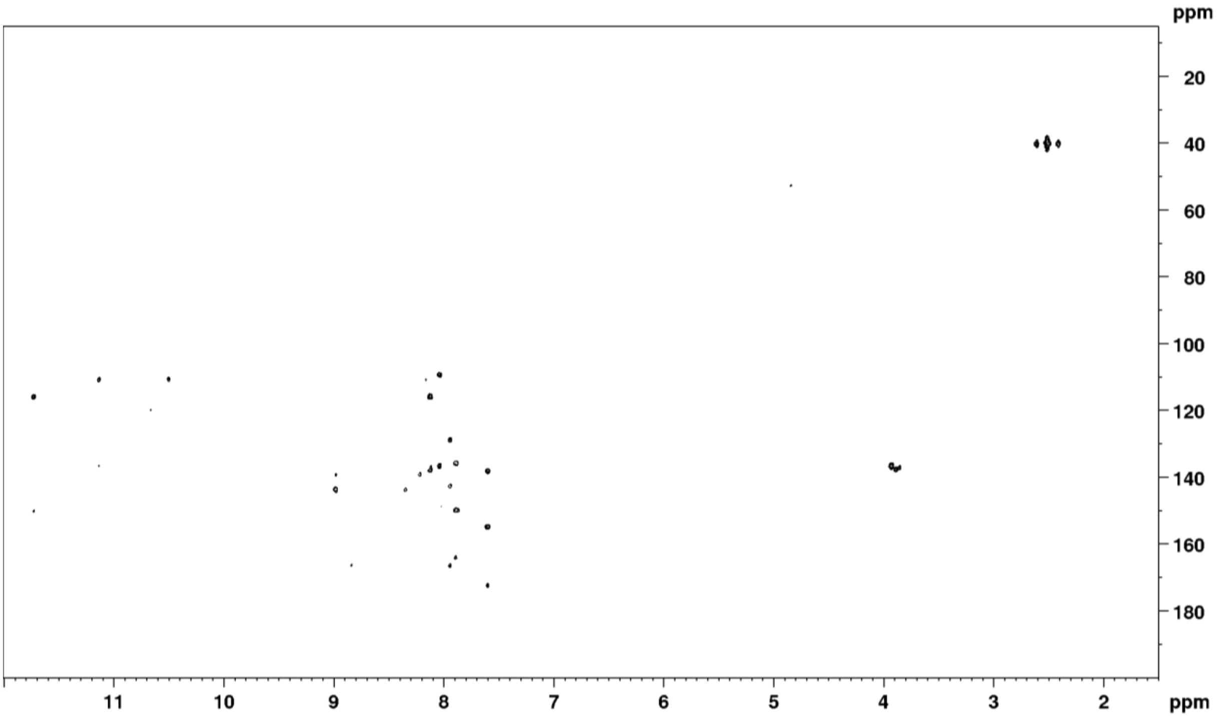

4. References

[1] M. P. Weinstein, *Methods for Dilution Antimicrobial Susceptibility Tests for Bacteria That Grow Aerobically*, CLSI, **2018**.

[2] J. Schindelin, I. Arganda-Carreras, E. Frise, V. Kaynig, M. Longair, T. Pietzsch, S. Preibisch, C. Rueden, S. Saalfeld, B. Schmid, J.-Y. Tinevez, D. J. White, V. Hartenstein, K. Eliceiri, P. Tomancak, A. Cardona, *Nat Methods* **2012**, *9*, 676–682.

[3] I. Behroz, P. Durkin, S. Grätz, M. Seidel, L. Rostock, M. Spinczyk, J. B. Weston, R. D. Süssmuth, *Chemistry – A European Journal* **2019**, *25*, 16538–16543.

[4] S. Kosol, L. Rostock, J. Barsig, T. Tabarelli, K. Hommernick, M. Kulike, T. Eulberg, M. Seidel, I. Behroz, L. Kleebauer, S. Grätz, A. Mainz, R. D. Süssmuth, *Chemical Science* **2023**, *14*, 5069–5078.

[5] L. Rostock, R. Driller, S. Grätz, D. Kerwat, L. von Eckardstein, D. Petras, M. Kunert, C. Alings, F.-J. Schmitt, T. Friedrich, M. C. Wahl, B. Loll, A. Mainz, R. D. Süssmuth, *Nature Communications* **2018**, *9*, 3095.

[6] E. Gasteiger, C. Hoogland, A. Gattiker, S. Duvaud, M. R. Wilkins, R. D. Appel, A. Bairoch, in *The Proteomics Protocols Handbook*, Humana Press, Totowa, NJ, **2005**, pp. 571–607.

[7] C. Gregor, K. C. Gwosch, S. J. Sahl, S. W. Hell, *Proceedings of the National Academy of Sciences* **2018**, *115*, 962–967.

[8] L. Zborovsky, L. Kleebauer, M. Seidel, A. Kostenko, L. von Eckardstein, F. Otto Gombert, J. Weston, R. D. Süssmuth, *Chemical Science* **2021**, *12*, 14606–14617.

[9] E. Michalczyk, K. Hommernick, I. Behroz, M. Kulike, Z. Pakosz-Stępień, L. Mazurek, M. Seidel, M. Kunert, K. Santos, H. von Moeller, B. Loll, J. B. Weston, A. Mainz, J. G. Heddle, R. D. Süssmuth, D. Ghilarov, *Nat Catal* **2023**, *6*, 52–67.
